# ﻿Matrix-based key to the click beetle genera of Canada and USA with a summary of habitat use (Coleoptera, Elateridae)

**DOI:** 10.3897/zookeys.1200.119315

**Published:** 2024-05-07

**Authors:** Hume B. Douglas, Frank E. Etzler, Paul J. Johnson, H.E. James Hammond

**Affiliations:** 1 Agriculture and Agri-Food Canada, Canadian National Collection of Insects, Arachnids and Nematodes, 960 Carling Avenue, Ottawa, Ontario, K1A 0C6, Canada Agriculture and Agri-Food Canada, Canadian National Collection of Insects, Arachnids and Nematodes Ottawa Canada; 2 Montana Department of Agriculture, 302 N. Roberts St., Helena, MT, 59601, USA Montana Department of Agriculture Helena United States of America; 3 Insect Biodiversity Lab, Box 2100A, 1030 N. Campus Drive, South Dakota State University, Brookings, South Dakota 57007, USA South Dakota State University Brookings United States of America; 4 Natural Resources Canada, Canadian Forest Service, Northern Forestry Centre, 5320 – 122 Street NW, Edmonton, Alberta, T6H 3S5 Canada Natural Resources Canada, Canadian Forest Service, Northern Forestry Centre Edmonton Canada

**Keywords:** Click beetle, crop pests, digital identification tools, interactive key, invasive alien species, key, LUCID, matrix key, wireworms

## Abstract

The Elateridae, or click beetles are abundant and diverse in most terrestrial ecosystems in North America, acting as plant pests and filling many other ecological roles. The 112 genera of Elateridae Leach, 1815, or click beetles, known from Canada and USA are included in a first comprehensive digital interactive key to adults. A link to an online peer-reviewed LUCID key to elaterid genera and downloadable LUCID files are provided. Diagnostic morphological summaries using information from the 61 characters and 158 character states of the matrix key are presented for all genera. A table summarizes current understanding of habitat use by all elaterid genera in Canada and USA from literature, collections, citizen science, and our own observations. Diversity of elaterid genera was high throughout warm and cool temperate regions, especially in mountainous areas and mesic woodlands. Larvae of most genera were associated with soil, litter and decaying wood.

## ﻿Introduction

Click beetles (Coleoptera: Elateridae) are common as adults and larvae in terrestrial ecosystems throughout the world. Adults are recognizable by the presence of a thoracic clicking mechanism combined with non-clubbed antennae and a labrum that is separated from the head capsule by a suture. The dorsoventrally compressed to nearly cylindrical larvae of Elateridae are present in soils and dead wood, with larvae of plant-damaging species known as wireworms. There are more than 400 valid elaterid genera worldwide, with 112 of these known from USA and Canada (Table 1), comprising nearly 1000 species (Johnson 2002). Phylogenetic analyses have prompted transfers of several genera from other beetle families into Elateridae (Kundrata and Bocak 2011; Kusy et al. 2018), and also demonstrate widespread homoplasy in morphological characters (Kundrata et al. 2018) making some elaterid genera (e.g., *Megapenthes* Kiesenwetter, 1858, see Douglas et al. 2021) difficult to define morphologically.

**Table 1.** Genera of Elateridae of Canada and USA with distribution and larval microhabitat.

Genus	Ecosystem/region	Habitat	Larval substrate	Source
*Acteniceromorphus* Kishii, 1955	Cool mesic, montane	Woodland, ecotones (edge habitats)	Soil, litter	18, 22, 27, 31, 32, 33
*Actenicerus* Kiesenwetter, 1858	Cool wetland, mesic prairie	Riparian meadow, woodland, wetland ecotones	Soil: organic	12, 16, 22, 33
*Aeolus* Eschscholtz, 1829	Warm temperate, not dry	Xeric to riparian meadow; suburban, agricultural	Soil, ant and termite nests	0, 3, 6, 9, 21, 29, 30, 33, 35
*Agriotes* Eschscholtz, 1829	Widespread, mesic	Meadow, woodland, desert, ecotones	Soil	0, 19, 21, 27, 28, 29, 30, 31, 36
*Agrypnus* Eschscholtz, 1829	Warm temperate	Xeric to mesic meadow, dunes, woodland, suburban	Soil, wood: subcortical	0, 20, 22, 24, 33
*Alaus* Eschscholtz, 1829	Temperate to warm temperate	Woodland, suburban	Wood	0, 5, 20, 22
*Ampedus* Dejean, 1833	Mesic temperate, montane	Woodland, savannah	Wood, litter	0, 12, 18, 19, 27, 29, 32, 33
*Anchastus* LeConte, 1853	Temperate	Woodland, desert steppe, suburban	Soil; wood: subcortical	1, 20, 22, 24, 33
*Anostirus* Thomson, 1859	Cool temperate	Woodland, embankments, savannah	Soil	12, 20, 22
*Anthracalaus* Fairmaire, 1888	Arizona	Desert riparian woodland, scrub	Unknown	22
*Anthracopteryx* Horn, 1891	Colorado and Wyoming	Mountain foothills; short grassland	Soil	9
*Aphricus* LeConte, 1853	Warm desert	Desert, chaparral	Unknown	22
*Aplastus* LeConte, 1859	Warm, dry	Scrub, savannah	Unknown	22, 33
*Aptopus* Eschscholtz, 1829	Warm desert	Scrubland, woodland	Soil	20, 22
*Ascoliocerus* Mequignon, 1930	Cool temperate	Subalpine meadow, tundra, riparian	Soil	9
*Athoplastus* Johnson & Etzler, 2018	Cool western, humid	Woodland, savannah	Litter	15
*Athous* Eschscholtz, 1829	Widespread	Woodland, savannah, meadow	Wood: subcortical, soil, litter,	12, 16, 18, 27, 38
*Barrelater* Johnson, 2014	Great Basin	Shrub-steppe, dunes	Sandy soils	23
*Beckerus* Johnson, 2008	Boreal/montane	Woodland, springs, bogs	Moss mats, litter	9, 16, 33
*Berninelsonius* Leseigneur, 1970	Boreal/montane	Meadow, riparian	Soil	22
*Billbrownia* Johnson, 2023	Cool to warm temperate	Woodland, savannah	Wood: subcortical	22, 33
*Bladus* LeConte, 1861	Warm temperate	Riparian woodland	Unknown	22
*Blauta* LeConte, 1853	Mesic warm temperate	Woodland, scrub, suburban	Unknown	22
*Campylomorphus* Jacquelin du Val, 1860	Western mountains	Woodland ecotone	Wood, litter	12, 22
*Cardiophorus* Eschscholtz, 1829	Temperate/tropical	Meadow, dune, woodland, alpine	Sandy soil, decayed wood	0, 14, 22
*Chalcolepidius* Eschscholtz, 1829	Warm temperate	Woodland, desert scrub	Wood	20, 22
*Corymbitodes* Buysson, 1904	Cool temperate	Woodland, mesic meadow	Soil	20, 22, 28, 33
*Ctenicera* Latreille, 1829	Boreal	Woodland, savannah	Soil	12, 16, 22, 32
*Dalopius* Eschscholtz, 1829	Temperate, mesic	Mesic woodland, meadow	Soil	0, 1, 12, 27, 29, 31, 35
*Danosoma* Thomson, 1859	Cool temperate	Woodland, savannah	Wood, subcortical	19, 20, 22
*Deilelater* Costa, 1975	Subtropical	Woodland, desert shrub	Soil, wood	22, 33
*Denticollis* Piller and Mitterpacher, 1783	Boreal	Woodland	Wood, litter	16, 18, 19, 22
*Deronocus* Johnson, 1997	California	Dry woodland, savannah, chaparral	Probably soil	22, 33
*Desolakerrus* Stibick, 1978	Montane	Desert, riparian (intermittent)	Soil	9
*Diacanthous* Reitter, 1905	Boreal	Woodland	Wood	9, 16, 18
*Dicrepidius* Eschscholtz, 1829	Temperate/tropical	Woodland, savannah, scrub, suburban	Wood	0, 3, 4, 7, 20, 22
*Diplostethus* Schwarz, 1907	Warm	Woodland, scrub, desert	Soil, wood	0, 20, 22, 24
*Dipropus* Germar, 1839	Temperate/tropical	Woodland, scrub, suburban	Wood, soil	0, 3, 4, 7, 20, 22
*Dixicollis* Johnson, 2021	Southeastern USA	Woodland	Soil	16, 22
*Dolerosomus* Motschulsky, 1859	Temperate	Montane woodland, hilltop meadow?	Unknown	20, 22, 33
*Drapetes* Dejean, 1821	Warm Temperate	Woodland, savannah	Wood, subcortical	2, 20, 22, 33
*Eanus* LeConte, 1861	Boreal and cool montane	Woodland, peatland, subalpine	Litter, mosses, soil	1, 9, 16 18, 19, 26, 33
*Elater* Linnaeus, 1758	Warm Temperate	Woodland, savannah, suburban	Tree cavities	0, 20, 22, 28, 34
*Elathous* Reitter, 1890	Widespread	Woodland, savannah	Soil, wood,	12, 16, 20, 22, 33, 37, 38
*Esthesopus* Eschscholtz, 1829	Warm Temperate/tropical	Desert, dune, woodland, savannah	Soil	20, 22, 24, 33
*Euplastius* Schwarz, 1903	Warm temperate	Woodland, meadow	Soil	22
*Euthysanius* LeConte, 1853	California	Scrub, savannah	Unknown	22
*Fleutiauxellus* Mequignon, 1930	Subarctic/boreal, eastern and western montane	Coastal meadow, montane meadow, riparian	Soil	9
*Floridelater* Douglas, 2017	Subtropical	Coastal dune	Soil	20, 22
*Gambrinus* LeConte, 1853	Widespread	Woodland, savannah, mesic meadow	Soil, litter, wood	12, 16, 22, 27, 31, 33
*Glyphonyx* Candèze, 1863	Mesic warm temperate	Woodland, meadow, suburban	Soil	0, 8, 10, 22
*Hadromorphus* Motschulsky, 1859	Cool temperate; lower montane	Woodland, mesic meadow, shrub steppe	Soil	16, 22, 29, 33, 35
*Hemicrepidius* Germar, 1839	Widespread	Woodland, savannah, meadow, coastal beach, montane, agricultural	Soil, litter, wood,	5, 16, 21, 26, 28, 29, 30, 33
*Heteroderes* Latreille, 1834	Warm	Dry meadow, woodland, desert, suburban	Soil	11, 22, 30, 33
*Horistonotus* Candèze, 1860	Temperate/tropical	Desert, woodland, meadow	Soil	0, 20, 22, 24
*Hypnoidus* Dillwyn, 1829	Cool temperate-boreal, alpine	Meadow, riparian, woodland, agricultural	Soil	12, 16, 18, 21, 22, 28, 29, 32
*Hypoganus* Kiesenwetter, 1858	Temperate, humid	Woodland, savannah	Wood: subcortical	8, 9, 10, 11, 36, 37
*Hypolithus* Eschscholtz, 1829	Cool temperate to subarctic	Marine shoreline	Soil	9, 12
*Idolus* Desbrochers des Loges, 1875	Cool temperate	Montane and hilltop meadow, woodland (*I.debilis*)	Soil, wood, litter/moss	0, 18, 20, 22, 33
*Ignelater* Costa, 1975	Subtropical	Woodland, scrub, suburban	Wood, litter, soil	20, 22, 33
*Lacon* Laporte, 1838	Widespread	Woodland, savannah	Wood: subcortical	0, 20, 22
*Laneganus* Johnson, 2021	Temperate, humid	Woodland, savannah	Wood	16, 20, 22,
*Lanelater* Arnett, 1952	Warm	Desert steppe, woodland, marine beach	Soil	13, 20, 22, 33
*Leptoschema* Horn, 1884	California	Woodland, savannah	Unknown	22
*Ligmargus* Stibick, 1976	Boreal/Montane	Riparian, alpine meadow	Soil	9
*Limonius* Eschscholtz, 1829	Widespread	Meadow, woodland, subalpine, marine dune	Soil, moss, litter	16, 21, 28, 29, 30, 31, 35
*Liotrichus* Kiesenwetter, 1858	Cool temperate, moist	Woodland	Soil, litter	12, 22
*Margaiostus* Stibick, 1978	Boreal/montane	Subalpine meadow, woodland	Soil	9, 27, 28
*Megapenthes* Kiesenwetter, 1858	Widespread	Woodland, desert	Soil, wood	0, 18, 19, 20, 22
*Melanactes* LeConte, 1853	Warm temperate	Woodland, savannah	Wood: subcortical	16, 22, 33
*Melanotus* Eschscholtz, 1829	widespread, tropical to cool temperate	Woodland, meadow, suburban, agricultural	Soil, wood	0, 27, 28, 29, 30, 31, 35
*Meristhus* Candèze, 1857	Warm temperate	Riparian	Sandy soil	20, 22, 33
*Metanomus* Buysson, 1887	Boreal	Woodland	Wood, litter	18, 20, 22, 31, 33
*Microhypnus* Kishii, 1976	Cool temperate to boreal	Riparian (lake and stream shores)	Soil	22
*Migiwa* Kishii, 1966	Temperate, dry	Meadow slopes, riparian	Soil	1, 9
*Monocrepidius* Eschscholtz, 1829	Tropical to warm temperate	Meadow, woodland, desert, suburban, agricultural	Soil	0, 10, 21, 22, 30
*Negastrius* Thomson, 1859	Cool temperate	Riparian, wetland, marine shoreline	Sand, gravel	0, 12, 20, 22
*Neohypdonus* Stibick, 1971	Cool temperate	Riparian, tundra, woodland	Soil	20, 22, 27, 31
*Neopristilophus* Buysson, 1894	Temperate, humid	Woodland	Wood	20, 22, 33
*Nitidolimonius* Johnson, 2008	Boreal/montane	Woodland, savannah, meadow	Litter, wood, mosses/soil	16, 18, 19, 27, 31, 33
*Octinodes* Candèze, 1863	Warm, dry	Savannah, scrub	Unknown	22, 33
*Oedostethus* LeConte, 1853	Cool temperate	Riparian, woodland, mesic meadow	Soil	1, 20, 22
*Oestodes* LeConte, 1853	Cool	Riparian, sandy meadow, saline mesic meadow	Soil	1, 20, 22
*Oistus* Candèze, 1857	Temperate west, mesic	Woodland, savannah	Wood	22, 26
*Orthostethus* Lacordaire, 1857	Warm	Woodland, dunes	Wood, soil	0, 20, 22
*Oxygonus* LeConte, 1863	Temperate, humid	Woodland, savannah, riparian	Soil	20, 22, 27, 33
*Paracardiophorus* Schwarz, 1895	Cool temperate, subalpine	Riparian, coastal and inland dunes, eroding banks	Soil: sandy	14, 22, 25
*Paractenicera* Johnson, 2008	Temperate, humid	Woodland	Unknown	22, 27
*Paradonus* Stibick, 1971	Cool temperate	Riparian, stream and lake shores	Soil	20, 22
*Parallelostethus* Schwarz, 1907	Warm, mesic	Woodland, savannah, suburban	Wood, Tree cavities	0, 22, 33
*Perissarthron* Hyslop, 1917	South Central U.S.A.	Riparian woodland, savannah	Unknown	22, 33
*Pheletes* Kiesenwetter, 1858	Cool temperate west	Woodland, savannah	Soil, wood	12
*Pherhimius* Fleutiaux, 1942	Warm humid	Woodland, savannah	Wood, soil	0, 20, 22
*Physorhinus* Germar, 1840	Subtropical/tropical, desert riparian woodland	Woodland, scrub	Wood	20, 22, 33
*Pityobius* LeConte, 1853	Warm temperate, east and west	Woodland, savannah	Wood, subcortical	0, 9, 33
*Proludius* Lane, 1971	Temperate, humid	Woodland, montane meadow	Soil	16, 20, 22
*Prosternon* Latreille, 1834	Cool temperate	Woodland, montane meadow	Soil, litter	12, 16, 22, 27, 28
*Pseudanostirus* Dolin, 1964	Temperate-boreal	Woodland, savannah	Soil, wood, litter	16, 18, 27, 28, 31, 33
*Pyrophorus* Billberg, 1820	Tropical, not established	Woodland, savannah	Soil, wood, litter	20, 22, 33
*Rismethus* Fleutiaux, 1947	Warm	Riparian	Riparian gravel	20, 22
*Scaptolenus* LeConte, 1853	Warm, seasonally dry	Dry meadow, savannah, desert steppe	Soil	22, 33
*Selatosomus* Stephens, 1830	Cool temperate, subalpine	Woodland, mesic meadow	Soil, moss/litter	12, 16 21, 27, 28, 29, 32, 31, 33, 35
*Selonodon* Latreille, 1834	Warm	Meadow, woodland, savannah	Soil	22, 33
*Sericus* Eschscholtz, 1829	Cool temperate	Woodland, peatland, mesic meadow	Soil, peat, litter, wood	0, 12, 22, 19
*Setasomus* Gurjeva, 1985	Cool temperate	Woodland, savannah	Litter, wood	16, 18, 22, 33
*Stropenron* Johnson, 2021	Temperate, humid	Woodland, savannah	Litter, wood	16, 18, 27, 31, 27, 33
*Sylvanelater* Johnson, 2008	Cool temperate	Woodland, meadow, savannah, suburban	Soil	16, 18, 27, 28, 29, 33
*Tesolasomus* Johnson, 2021	Cool temperate	Woodland, meadow, montane steppe	Soil	16, 20, 22, 33
*Tetralimonius* Etzler, 2019	Temperate east and west	Woodland	Unknown	
*Vesperelater* Costa, 1975	Riparian in desert woodland, savannah	woodland, savannah, scrub	Unknown	22
*Vittathous* Johnson, 2021	Warm temperate	Woodland, mesic meadow	Soil, litter	22, 33
*Zorochros* Thomson, 1859	Cool temperate	Riparian	Sand, gravel	12, 20, 22

Key to sources: 0, Becker and Dogger (1991); 1, Brooks (1961); 2, Burakowski (1973); 3, Casari (1996); 4, Casari (2002); 5, Casari (2003); 6, Casari (2006); 7, Casari and Biffi (2012); 8, Cheshire and Jones (1988); 9, CNC specimens; 10, Deen and Cuthbert (1955); 11, Dobrovsky (1954); 12, Dolin (1978); 13, Donlan et al. (2004); 14, Douglas (2003); 15, Etzler and Johnson (2018); 16, Glen (1950); 18, Hammond (1997); 19, Hammond et al. (2017); 20, HD experience; 21, Hoernemann et al. (2001); 22, iNaturalist (2023); 23, Johnson (2014); 24, Johnston et al. (2023); 25, Lanchester and Lane (1972); 26 Lane (1972); 27) Levesque and Levesque (1980); 28) Levesque and Levesque (1993); 29, Milosavljevic et al. (2016); 30, Morrill (1978); 31, Nol et al. (2006); 32, Papp (1978); 33, PJJ experience; 34, Svensson et al. (2004); 35 Toba and Campbell (1992); 36, Traugott et al. (2014); 37 Van Dyke (1932); 38, Webster et al. (2012). Litter here also includes decayed wood fragments.

Elateridae are hard-bodied (although the abdomen is soft in some), mostly elongate beetles with a pro-mesothoracic clicking mechanism. The peg-like prosternal process fits into the mesothoracic intercoxal cavity but is not fixed there. Additionally, the labrum is exposed, antennae are not clubbed, and each leg has five tarsomeres. Elateridae can mostly be recognized and identified to genera by adult external morphology, although some generic concepts need further analysis. Elaterid genera are distinguished primarily by characters of the head, thorax, and legs. Identification usually begins by examining the frons, pronotum, mesosternum, mesothoracic cavities, elytra, and tarsi to form an overall impression of a specimen’s morphology. Northern North America is one of few world regions which are covered by recent comprehensive keys to elaterid genera as adults (Arnett 1968; Johnson 2002). Larvae are also treated by partial keys to genera (Becker and Dogger 1991; Johnson 1993). Prior keys to adults are dichotomous and require readers to interpret difficult characters early in the specimen identification process. Digital matrix keys have improved ease of identification in other beetle groups (e.g., Hulcr and Smith 2010; Staines 2012), and we hope this approach will also work for Elateridae.

Digital matrix keys can facilitate taxonomic identifications by allowing flexible identification pathways, allowing users to select the characters that they can observe and interpret most easily. This can reduce the number of difficult character interpretations and allow for identification using fewer characters. We used a wide selection of mainly external morphological characters to generate a key to genera in the LUCID platform (Identic Ltd. 2020) to facilitate identification of adult Elateridae.

## ﻿Materials and methods

Specimens used to generate the key were examined from the Canadian National Collection of Insects, Arachnids and Nematodes (CNC, Ottawa, Canada), and the South Dakota State University Insect Collection. These specimens were previously identified to species by E.C. Becker, W.J. Brown, M.C. Lane and the authors using a wide variety of literature and often comparison to type specimens. Specimen characters were photographed using a Leica M205c stereoscope and multifocus images assembled using Leica LAS 4.8 software. Habitus photos were taken using a Leica M80 dissecting scope with Leica EC3 camera attachment. Subsequent images were then rendered using CombineZ stacking software and GIMP v. 2.10.34 photo editor. The key and informal taxon descriptions were built using LUCID 3.6 software.

Most diagnostic characters were modified from keys by Stibick (1976; 1978; 1990), and Johnson (2002). Some, including presence of microserration of edges of the pronotum, abdominal ventrites and paramere setal counts, were adapted from Douglas (2011). Others were diagnostic characters for genera described from Douglas (2017), Etzler (2019), Etzler and Johnson (2018), Fuller (1994), and Johnson (2021). We also developed several new characters here for pronotal setal vestiture direction, and microserration of the edges of the mesosternal cavity. The key includes some aedeagal characters because these are useful and readily observable in standard dissection of males and in specimens with extended aedeagi. Other genitalic characters including those from pregenital segments and internal genitalia were not included. Character definitions were edited for objectivity and tested by multiple users to improve diagnostic effectiveness.

Measurements: body length was measured from the anterior edge of the head capsule to the apex of the closed elytra; antennomeres were measured along their dorsal edges as the entire distance between the two adjacent antennomeres; pronotum length was measured at the midline, and width at the widest part. Informal descriptions and diagnostic summaries were generated in LUCID from morphological data used to encode genera and groups of species, together with LUCID’s natural language description template tool.

We encoded specimens from more than 400 species directly into LUCID. One to 38 species were encoded per genus representing most or all species in most genera. Taxon sampling and testing were most extensive for diagnostically problematic or morphologically divergent genera. The morphologically diverse genus *Athous* Eschscholtz, 1829 was divided into species groups defined by Becker (1979). A further 14 genera were subdivided in the key matrix to accommodate morphological divergence in diagnostic characters and improve the genus-level diagnostic efficacy.

Distributional and habitat associations of Elateridae were compiled (Table 1) for the areas where they have been observed at scales including general bioregion, habitat, and larval substrate. Sources include published literature (including larval ecology inferences for the same genus from other world regions); museum specimen labels; records from research grade iNaturalist (2023) observations reviewed by HD; and our own field notes and observations. Data entered in each cell was designed for maximum specificity, so that cell contents within a column may require reader interpretation prior to comparison. Meadow includes all non-wetland herbaceous habitats such as cropland, abandoned cropland, native meadow, and grassland. This summary includes both tested knowledge and preliminary hypotheses in need of further study.

## ﻿Result and discussion

### ﻿Key to adults

The interactive LUCID key and accompanying diagnostic summaries include multiple routes to identification of one or both sexes of many species at the following internet URL: https://keys.lucidcentral.org/keys/v4/nearctic_elateridae/.

In more than 95% of cases, specimens could be accurately identified to genus via the key using morphology alone. Our testing did not lead to any incorrect identifications. However, some specimens of 11 genera were resolved only as belonging to one of two or three genera based on morphology. Here some *Margaiostus* Stibick, 1978 specimens were not separated from *Hypnoidus* Dillwyn, 1829; *Paractenicerafulvipes* (Bland, 1863) and *Proludiussilvaticus* (Van Dyke, 1932) were not separated from *Acteniceromorphus* Kishii; *Selatosomusnigricans* (Fall, 1910) was not separated from *Hypoganus* Kiesenwetter, 1858; *Setasomusaratus* (LeConte, 1853) was not separated from *Selatosomus* Stephens, 1830 or *Tesolasomus* Johnson, 2021; *Setasomusnitidulus* (LeConte, 1853) and *S.rufopleuralis* (Fall, 1933) were not separated from *Billbrownia* Johnson, 2023; some *Blauta* LeConte specimens were not separated from *Dipropus* Germar, 1839. In some cases, these could be discriminated by entering specimen collection locality data. We believe incompletely resolved identifications result from incomplete generic concepts needing further revisions in the Hypnoidinae, and parts of the Dendrometrinae, and Elaterinae. Diagnostic data from the key are also available as an LIF3 file (Suppl. material 1), and as a comma separated values file (Suppl. material 2).

Elaterid genera in the study area have mostly been diagnosed and defined in the taxonomic literature based on morphological similarity but the monophyly of most has not been tested phylogenetically. Some generic concepts do not match those applied to other parts of the world fauna, nor do they include useful larval characters. For example, North American *Athous*, *Ctenicera* Latreille, 1829, and *Megapenthes* include species matching multiple Palaearctic genera. Our goal was to provide a diagnostic tool to match all species to their current taxonomic placement. Further future changes are expected.

### ﻿Using the key

Instructions for using this key mostly match instructions from other recent LUCID keys (e.g., Brust et al. 2020; Klimov et al. 2016). We generally recommend using the LUCID wand tool to select characters for identification, plus the geographic characters at the bottom. Experienced elaterid identifiers may instead wish to focus efforts on observed distinctive characteristics of the specimen. The key is only reliable for identification of Elateridae from Canada and USA because the morphologies of species of these genera occurring elsewhere were not considered.

### ﻿Ecological insights

Looking at regional diversity of elaterid genera from the online key, and the summary genus distributions, habitat, and microhabitat (Table 1) use allows preliminary insights into regional elaterid ecology. We found that some northern areas like Ontario (72 of 143 genera), New York (75 genera), or British Columbia (76 genera) have similar or higher numbers of genera than some southern areas like Florida (43 genera), Texas (44 genera), or even mountainous Arizona and New Mexico (51) genera. This shows that generic level diversity is not necessarily higher in unglaciated warm temperate areas than in once glaciated cool temperate areas. However, California (88 genera) did have the highest diversity, perhaps because of its diverse habitat types. Surprisingly, even this most diverse state was home to only ~ 60% of genera. The most northern regions like Alaska (37 genera) and Northwest Territories (32) genera had the lowest diversity, with a number of specialized northern genera. Overall, latitude was not a primary driver of elaterid genus-level diversity, except in subarctic and arctic areas.

By habitat type, most genera (89 of 143) were found, at least in part, in woodland. Some 36–38 genera were known from grassland. Many species were not restricted to either, with 30–32 genera inhabiting both woodland and grassland, and a further 31 genera known from intermediate habitats including scrub and savannah. At least three genera were known from peatlands. We expect genera with wood-dwelling larvae (and litter-dwelling larvae) to inhabit woody habitats, while those with soil dwelling larvae could inhabit both woody and herbaceous habitats. This hypothesis is consistent with the finding that only four genera were restricted to grassland (i.e., herbaceous) habitats.

By larval substrate type, most genera (80 of 143) were associated with soil and litter (including sand and gravel) and 40 with wood (partially decayed). Of these, 52 were associated with soil and litter but not wood, and 19 were associated with wood but not soil or litter. Plant pest genera include those known only from soil and genera with species known from both soil and wood. A further 21–23 genera had larvae known from both wood and soil or litter. These came from a combination of genera with species with different habitat specializations and from individual species with larva in both in decayed wood and soil or litter.

### ﻿Informal descriptions and diagnostic summaries

Condensed informal descriptions and diagnostic summaries are provided for each genus or partial genus below. Summaries are condensed sets of characters meant to diagnose each from all other genera in this text. The informal descriptions give a wider morphological summary of a genus or partial genus, but do not repeat common characteristics of the subfamily or tribe (Table 2) to which it belongs. Where a character state is attributed to most members of a higher-level taxon, only alternate character states are reported for genera within that group. Expanded informal descriptions are provided in Suppl. material 3, and in the online key. Supporting figures can be found in the online lucid key. Informal descriptions and summaries are also provided for local members of subfamilies and tribes including two or more genera in the study area.

**Table 2.** Summary of the classification used for genera of Elateridae found in Canada and USA.

Subfamily	Tribe	Genus
Lissominae		*Drapetes* Dejean, 1821
Oestodinae		*Bladus* LeConte, 1861
Oestodinae		*Oestodes* LeConte, 1853
Elaterinae	Melanotini	*Melanotus* Eschscholtz, 1829
Elaterinae	Dicrepidiini	*Blauta* LeConte, 1853
Elaterinae	Dicrepidiini	*Dicrepidius* Eschscholtz, 1829
Elaterinae	Dicrepidiini	*Dipropus* Germar, 1839
Elaterinae	Ampedini	*Ampedus* Dejean, 1833
Elaterinae	Physorhinini	*Anchastus* LeConte, 1853
Elaterinae	Physorhinini	*Physorhinus* Germar, 1840
Elaterinae	Megapenthini	*Megapenthes* Kiesenwetter, 1858
Elaterinae	Aplastini	*Aplastus* LeConte, 1859
Elaterinae	Aplastini	*Euthysanius* LeConte, 1853
Elaterinae	Aplastini	*Octinodes* Candèze, 1863
Elaterinae	Cebrionini	*Scaptolenus* LeConte, 1853
Elaterinae	Cebrionini	*Selonodon* Latreille, 1834
Elaterinae	Elaterini	*Campylomorphus* Jacquelin du Val, 1860
Elaterinae	Elaterini	*Diplostethus* Schwarz, 1907
Elaterinae	Elaterini	*Dolerosomus* Motschulsky, 1859
Elaterinae	Elaterini	*Elater* Linnaeus, 1758
Elaterinae	Elaterini	*Orthostethus* Lacordaire, 1857
Elaterinae	Elaterini	*Parallelostethus* Schwarz, 1907
Elaterinae	Elaterini	*Sericus* Eschscholtz, 1829
Elaterinae	Pomachiliini	*Idolus* Desbrochers, 1875
Elaterinae	Pomachiliini	*Leptoschema* Horn, 1885
Elaterinae	Agriotini	*Agriotes* Eschscholtz, 1829
Elaterinae	Agriotini	*Dalopius* Eschscholtz, 1829
Elaterinae	Synaptini	*Glyphonyx* Candèze, 1863
Pityobiinae		*Pityobius* LeConte, 1853
Agrypninae	Pseudomelanactini	*Anthracalaus* Fairmaire, 1888
Agrypninae	Pseudomelanactini	*Lanelater* Arnett, 1952
Agrypninae	Agrypnini	*Agrypnus* Eschscholtz, 1829
Agrypninae	Agrypnini	*Danosoma* Thompson, 1859
Agrypninae	Agrypnini	*Lacon* Laporte, 1838
Agrypninae	Agrypnini	*Meristhus* Candèze, 1857
Agrypninae	Agrypnini	*Rismethus* Fleutiaux, 1947
Agrypninae	Hemirhipini	*Alaus* Eschscholtz, 1829
Agrypninae	Hemirhipini	*Chalcolepidius* Eschscholtz, 1829
Agrypninae	Hemirhipini	*Pherhimius* Fleutiaux, 1942
Agrypninae	Pyrophorini	*Deilelater* Costa, 1975
Agrypninae	Pyrophorini	*Ignelater* Costa, 1975
Agrypninae	Pyrophorini	*Pyrophorus* Billberg, 1820
Agrypninae	Pyrophorini	*Vesperelater* Costa, 1975
Agrypninae	Oophorini	*Aeolus* Eschscholtz, 1829
Agrypninae	Oophorini	*Deronocus* Johnson, 1995
Agrypninae	Oophorini	*Heteroderes* Latreille, 1834
Agrypninae	Oophorini	*Monocrepidius* Eschscholtz, 1829
Hypnoidinae		*Ascoliocerus* Méquignon, 1930
Hypnoidinae		*Berninelsonius* Leseigneur, 1970
Hypnoidinae		*Desolakerrus* Stibick, 1978
Hypnoidinae		*Hypnoidus* Dillwyn, 1829
Hypnoidinae		*Hypolithus* Eschscholtz, 1829
Hypnoidinae		*Ligmargus* Stibick, 1976
Hypnoidinae		*Margaiostus* Stibick, 1978
Negastriinae		*Fleutiauxellus* Méquignon, 1930
Negastriinae		*Microhypnus* Kishii, 1976
Negastriinae		*Migiwa* Kishii, 1966
Negastriinae		*Negastrius* Thomson, 1859
Negastriinae		*Neohypdonus* Stibick, 1971
Negastriinae		*Oedostethus* LeConte, 1853
Negastriinae		*Paradonus* Stibick, 1971
Negastriinae		*Zorochros* Thomson, 1859
Cardiophorinae		*Aphricus* LeConte, 1853
Cardiophorinae		*Aptopus* Eschscholtz, 1829
Cardiophorinae		*Cardiophorus* Eschscholtz, 1829
Cardiophorinae		*Esthesopus* Eschscholtz, 1829
Cardiophorinae		*Floridelater* Douglas, 2017
Cardiophorinae		*Horistonotus* Candèze, 1860
Cardiophorinae		*Paracardiophorus* Schwarz, 1895
Dendrometrinae	Oxynopterini	*Melanactes* LeConte, 1853
Dendrometrinae	Oxynopterini	*Oistus* Candèze, 1857
Dendrometrinae	Oxynopterini	*Perissarthron* Hyslop, 1917
Dendrometrinae	Prosternini	*Acteniceromorphus* Kishii, 1955
Dendrometrinae	Prosternini	*Actenicerus* Kiesenwetter, 1858
Dendrometrinae	Prosternini	*Anostirus* C.G. Thomson, 1859
Dendrometrinae	Prosternini	*Anthracopteryx* Horn, 1891
Dendrometrinae	Prosternini	*Athoplastus* Johnson & Etzler, 2018
Dendrometrinae	Prosternini	*Beckerus* Johnson, 2008
Dendrometrinae	Prosternini	*Billbrownia* Johnson, 2023
Dendrometrinae	Prosternini	*Corymbitodes* Buysson, 1904
Dendrometrinae	Prosternini	*Ctenicera* Latreille, 1829
Dendrometrinae	Prosternini	*Dixicollis* Johnson, 2021
Dendrometrinae	Prosternini	*Eanus* LeConte, 1861
Dendrometrinae	Prosternini	*Hadromorphus* Motschulsky, 1859
Dendrometrinae	Prosternini	*Hypoganus* Kiesenwetter, 1858
Dendrometrinae	Prosternini	*Laneganus* Johnson, 2001
Dendrometrinae	Prosternini	*Liotrichus* Kiesenwetter, 1858
Dendrometrinae	Prosternini	*Metanomus* Buysson, 1887
Dendrometrinae	Prosternini	*Neopristilophus* Buysson, 1894
Dendrometrinae	Prosternini	*Nitidolimonius* Johnson, 2008
Dendrometrinae	Prosternini	*Oxygonus* LeConte, 1863
Dendrometrinae	Prosternini	*Paractenicera* Johnson, 2008
Dendrometrinae	Prosternini	*Proludius* Lane, 1971
Dendrometrinae	Prosternini	*Prosternon* Latreille, 1834
Dendrometrinae	Prosternini	*Pseudanostirus* Dolin, 1964
Dendrometrinae	Prosternini	*Selatosomus* Stephens, 1830
Dendrometrinae	Prosternini	*Setasomus* Gurjeva, 1985
Dendrometrinae	Prosternini	*Stropenron* Johnson, 2021
Dendrometrinae	Prosternini	*Sylvanelater* Johnson, 2008
Dendrometrinae	Prosternini	*Tesolasomus* Johnson, 2021
Dendrometrinae	Dendrometrini	*Athous* Eschscholtz, 1829
Dendrometrinae	Dendrometrini	*Barrelater* Johnson, 2014
Dendrometrinae	Dendrometrini	*Denticollis* Piller & Mitterpacher, 1783
Dendrometrinae	Dendrometrini	*Diacanthous* Reitter, 1852
Dendrometrinae	Dendrometrini	*Elathous* Reitter, 1890
Dendrometrinae	Dendrometrini	*Euplastius* Reitter, 1890
Dendrometrinae	Dendrometrini	*Gambrinus* LeConte, 1853
Dendrometrinae	Dendrometrini	*Hemicrepidius* Germar, 1839
Dendrometrinae	Dendrometrini	*Limonius* Eschscholtz, 1829
Dendrometrinae	Dendrometrini	*Pheletes* Kiesenwetter, 1858
Dendrometrinae	Dendrometrini	*Tetralimonius* Etzler, 2019
Dendrometrinae	Dendrometrini	*Vittathous* Johnson, 2021

In some cases, these treatments may be more useful than established diagnoses and descriptions because they include standardized information for a broader set of diagnostic characters. However, their direct utility is mostly limited to Canada and USA because only species from there were used to characterize the genera. This means some generic concepts differ from those applied elsewhere in the world (e.g., *Athous*, *Ctenicera*, *Megapenthes*), and that some morphological variability within widely distributed genera were not included (e.g., the larger-bodied Neotropical species of *Monocrepidius* Eschscholtz, 1829 with spinose elytral apices, Marinho et al. 2023).

**Subfamily Lissominae**

**Genus *Drapetes* Dejean, 1821**

**Habitus.** Body length 2–6 mm. Scale-like setae absent. ***Head*.** Supra-antennal carinae variable; hypognathous; frons without triangular depression. Antennae with 11 antennomeres, not pectinate, sensory elements beginning on antennomere IV. ***Prothorax*.** Pronotum with dorsal punctures uniform sized, simple, without tubercles between punctures; pronotal lateral carinae complete, meeting anterior edge of prothorax at ~ 90° in lateral view, not serrate; bioluminescent spots absent; hind angle carinae present (single); posterior edge of pronotum without sublateral notches, crenellations absent; pronotosternal sutures excavate (able to contain antennae). Prosternum with sides straight at midlength; prosternal process not curved upward ≥ 40° in lateral view. ***Mesothorax*.** Mesocoxal cavities open to mesepimeron only; mesoventral cavity without serration along sides. Scutellar shield (scutellum hereafter) with anterior edge not concave, posterior edge pointed (acuminate). Elytra. Anterior edge straight to arcuate near humeri in dorsal view, integument marked with spots or bands in most; some with pattern from differences in setal color; setal vestiture sparse on disk in most, apex not spinose. ***Legs*.** Metacoxal plate reaching side; tarsal pads or membranous lobes present on multiple tarsomeres, (I, II, III, and IV or II, III, and IV); claws without setae, appendiculate, or bifid. ***Ventrites***. Sides not microserrate. ***Aedeagus*.** Parameres with articulation at base, apical lateral expansions absent.

**Summary.** Pronotosternal sutures excavate, multiple tarsomeres lobed. Small, distinctive Elateridae, but sometimes mistaken for other beetle families.

**Subfamily Oestodinae**

**Habitus.** Body length 4–9 mm. Scale-like setae absent. ***Head*.** Supra-antennal carinae fading on frons or absent; hypognathous; frons without triangular depression. Antennae with 11 antennomeres, not pectinate. ***Prothorax*.** Pronotum punctures uniform sized; without tubercles between punctures; lateral carinae complete, meeting anterior edge of prothorax at ~ 90° in lateral view, not serrate; bioluminescent spots absent; posterior edge with crenellations absent. Prosternum with sides straight or concave at midlength; prosternal process curved upward > 40° in lateral view. ***Mesothorax*.** Mesocoxal cavities open; mesoventral cavity without serration along sides. Scutellum with anterior edge not concave. Elytra. Anterior edge straight to arcuate near humeri in dorsal view, integument unmarked with spots or transverse bands, elytral apex not spinose. ***Legs*.** Metacoxal plate reaching side, plate without elongation in mesal half; tarsal pads absent; claws without setae, apex simple. **Ventrites**. Sides not microserrate. ***Aedeagus*.** Parameres articulated at base.

**Genus *Bladus* LeConte, 1861**

**Habitus.** Body length 4–6 mm. ***Head*.** Supra-antennal carinae fading on frons or absent; frons without triangular depression. Antennae with sensory elements beginning on antennomere IV. ***Prothorax*.** Pronotum with some punctures umbilicate; hind angle carinae absent; posterior edge of pronotum without sublateral notches; pronotosternal sutures closed, hypomeral bead present; hypomeron with posterior edge near hind angle without concavity. Prosternum with sides straight at midlength. ***Mesothorax*.** Scutellum with posterior edge rounded (arcuate). Elytra. Striae present; setal vestiture even and mainly parallel. ***Aedeagus*.** Parameres without apical lateral expansions.

**Summary.** Hypognathous, supra-antennal carinae absent or fading on frons, sensory elements beginning on antennomere IV (apical sensorium with short erect setae), prosternal sides straight, prosternal process ascendent, posterior edge of scutellum rounded.

**Genus *Oestodes* LeConte, 1853**

**Habitus.** Body length 4–9 mm. ***Head*.** Supra-antennal carinae fading on frons (not reaching another structure). Antennae with sensory elements beginning on antennomere III. ***Prothorax*.** Pronotum with dorsal punctures all simple; hind angle carinae present (single); posterior edge of pronotum with sublateral notches present; pronotosternal sutures open or closed; hypomera with arcuate concavity on posterior edge near hind angle. Prosternum with sides concave at midlength. ***Mesothorax*.** Scutellum rounded posterad or pointed (acuminate). Elytra. Setal vestiture sparse on disk or, if present, even and mainly parallel. ***Aedeagus*.** Parameres apical lateral expansions present, without setae or each paramere with three or more setae.

**Summary.** Hypognathous, supra-antennal carinae fading on frons, pronotum with sublateral notches, prosternal sides concave at midlength, prosternal process ascendant.

**Subfamily Elaterinae**

**Habitus.** Body length 3–35 mm. Scale-like setae absent. ***Prothorax*.** Pronotum without tubercles between punctures, lateral carinae not serrate; crenellations absent, bioluminescent spots absent. Prosternum with sides concave at midlength in most. ***Mesothorax*.** Mesocoxal cavities not closed. Elytra. Setal vestiture even and mainly parallel, apex not spinose. ***Legs*.** Tarsal pads or membranous lobes absent, present on tarsomere III only, or present tarsomeres II and III; claws without setae, apex simple or with 3 or more points. **Ventrites**. Sides not microserrate. ***Aedeagus*.** Parameres with articulation at base, apical lateral expansions present or absent.

This group is defined based on evidence from larvae and DNA. It is difficult to define based on adult morphology.

**Tribe Melanotini**

**Genus *Melanotus* Eschscholtz, 1829**

**Habitus.** Body length 3–30 mm. ***Head*.** Supra-antennal carinae joining medially (forming shelf); nasale (head capsule below edge of frontal carina) with outline concave in lateral view; frons without triangular depression; gena not broadened anteriorly below eye, not extending spine-like anterior to basal tubercle of mandible (ventral condyle). Antennae not pectinate, sensory elements beginning on antennomere IV. ***Prothorax*.** Pronotum with some punctures umbilicate; pronotal lateral carinae complete, meeting anterior edge of prothorax at ~ 90° in lateral view; hind angle carinae present (one or two carinae); posterior edge of pronotum with sublateral notches present; pronotosternal sutures open, hypomeral bead present; hypomera with arcuate or angulate concavity on posterior edge near hind angle. Prosternum with sides concave at midlength; prosternal process not curved upward ≥ 40° in lateral view. ***Mesothorax*.** Mesocoxal cavities not closed; mesoventral cavity without serration along sides. Scutellum with anterior edge not concave, posterior edge rounded (arcuate) or bilobed (concave at midline). Elytra. Striae present; anterior edge straight to arcuate or sinuate (recurved) near humeri in dorsal view, integument unmarked; without pattern from differences in setal color; setal vestiture even and mainly parallel. ***Legs*.** Metacoxal plate without elongation in mesal half; tarsal pads absent, tarsal claws with three or more points. ***Aedeagus*.** Each paramere with three or more setae.

**Summary.** Tarsal claws each with six or more points per side, pronotal lateral carina reaching anterior edge of pronotum.

**Tribe Dicrepidiini**

**Habitus.** Body length 4–18 mm. ***Head*.** Supra-antennal carinae joining medially (forming shelf); hypognathous; frons without triangular depression; gena not broadened anteriorly below eye, not extending spine-like anterior to basal tubercle of mandible (ventral condyle). ***Prothorax*.** Pronotum with dorsal punctures uniform sized, some or all punctures umbilicate, lateral carinae hidden anteriorly in dorsal view, meeting anterior edge of prothorax at ~ 90° in lateral view; hind angle carinae present (single); pronotosternal sutures open; hypomera with arcuate concavity on posterior edge near hind angle. Prosternum with sides concave at midlength. ***Mesothorax*.** Mesocoxal cavities open; mesoventral cavity without serration along sides. Scutellum with anterior edge not concave. Elytra. Striae present; anterior edge straight to arcuate near humeri in dorsal view, integument unmarked; without pattern from differences in setal color; setal vestiture even and mainly parallel. ***Legs*.** Tarsal pads or membranous lobes present on multiple tarsomeres, (II and III), tarsal claws simple. ***Aedeagus*.** Parameres with apical lateral expansions present; each paramere with three or more setae.

**Genus *Blauta* LeConte, 1853**

**Habitus.** Body length 4–18 mm. ***Head*.** Nasale (head capsule below edge of frontal carina) with outline concave in lateral view; hypognathous. Antennae not pectinate, sensory elements beginning on antennomere III or IV. ***Prothorax*.** Pronotal lateral carinae complete or incomplete anteriorly; posterior edge of pronotum with sublateral notches in some; prosternal process not curved upward ≥ 40° in lateral view. ***Mesothorax*.** Mesocoxal cavities open, scutellum with posterior edge rounded (arcuate). ***Legs*.** Metacoxal plate without elongation in mesal half.

**Summary.** Nasale without dorsally convergent carinae, hypognathous, pronotal sculpture rugose, pronotosternal sutures open, tarsomeres II and III lobed. Incompletely separated from *Dipropus* here.

**Genus *Dicrepidius* Eschscholtz, 1829**

**Habitus.** Body length 10–15 mm. ***Head*.** Nasale (head capsule below edge of frontal carina) with outline not concave in lateral view, nasale with two dorsally convergent carinae. Antennae pectinate or not, sensory elements beginning on antennomere III. ***Prothorax*.** Pronotal lateral carinae complete; posterior edge of pronotum with sublateral notches present, hypomeral bead present; prosternal process not curved upward ≥ 40° in lateral view. ***Mesothorax*.** Mesocoxal cavities open, scutellum rounded posterad. ***Legs*.** Metacoxal plate without elongation in mesal half.

**Summary.** With dorsally convergent carinae above labrum.

**Genus *Dipropus* Germar, 1839**

**Habitus.** Body length 6–18 mm. ***Head*.** Nasale (head capsule below edge of frontal carina) with outline concave in lateral view; hypognathous. Antennae not pectinate, sensory elements beginning on antennomere III. ***Prothorax*.** Pronotal lateral carinae complete; posterior edge of pronotum with sublateral notches present, hypomeral bead present. ***Mesothorax*.** Mesocoxal cavities open, scutellum rounded posterad or pointed (acuminate).

**Summary.** Nasale without dorsally convergent carinae, hypognathous, pronotal sculpture fine to moderately rugose, pronotosternal sutures open, tarsomeres II and III lobed. Incompletely separated from *Blauta* here.

**Tribe Ampedini**

**Genus *Ampedus* Dejean, 1833**

**Habitus.** Body length 3–18 mm. ***Head*.** Supra-antennal carinae complete across frons in most, but narrowly separated from labrum; hypognathous in most; frons without triangular depression; gena not broadened anteriorly below eye, not extending spine-like anterior to basal tubercle of mandible (ventral condyle). Antennae not pectinate, sensory elements beginning on antennomere IV. ***Prothorax*.** Pronotum with dorsal punctures uniform sized; pronotal lateral carinae complete, meeting anterior edge of prothorax at ~ 90° in lateral view; hind angle carinae present (one or two carinae); posterior edge of pronotum without sublateral notches; pronotosternal sutures open, hypomeral bead present; hypomera with arcuate concavity on posterior edge near hind angle. Prosternum with sides straight or concave at midlength. ***Mesothorax*.** Mesocoxal cavities open; mesoventral cavity without serration along sides. Scutellum with anterior edge not concave, posterior edge pointed (acuminate). Elytra. Striae present; anterior edge straight to arcuate near humeri in dorsal view, integument marked with spots or transverse bands, or unmarked; without pattern from differences in setal color; setal vestiture even and mainly parallel. ***Legs*.** Metacoxal plate elongate in mesal half, plate reaching side; tarsal pads absent, claws simple. ***Aedeagus*.** Parameres with apical lateral expansions present; each paramere with three or more setae.

**Summary.** Supra-antennal carina complete, antennae not pectinate, pronotosternal sutures open, mesoventral cavity non-serrate, mesocoxal cavities open, metacoxal plates elongate, tarsi simple.

**Tribe Physorhinini**

**Habitus.** Body length 3–15 mm. ***Head*.** Supra-antennal carinae joining medially (forming shelf); nasale (head capsule below edge of frontal carina) with outline concave in lateral view; hypognathous; frons without triangular depression. Antennae not pectinate, sensory elements beginning on antennomere IV. ***Prothorax*.** Pronotum with dorsal punctures uniform sized; pronotal lateral carinae complete, meeting anterior edge of prothorax at ~ 90° in lateral view; hind angle carinae present (one or two carinae); hypomeral bead present; hypomera with arcuate concavity on posterior edge near hind angle. Prosternum with sides concave at midlength. ***Mesothorax*.** Mesoventral cavity without serration along sides. Scutellum with anterior edge not concave. Elytra. Striae present; anterior edge straight to arcuate near humeri in dorsal view; without pattern from differences in setal color; setal vestiture even and mainly parallel. ***Legs*.** Metacoxal plate elongate in mesal half; tarsal pads or membranous lobes present on tarsomere III only, tarsal claws simple. ***Aedeagus*.** Each paramere with three or more setae.

**Genus *Anchastus* LeConte, 1853**

**Habitus.** Body length 3–15 mm. ***Head*.** Hypognathous. Antennae with sensory elements beginning on antennomere IV. ***Prothorax*.** Pronotum with lateral carinae hidden anteriorly in dorsal view; hind angle carinae present (one or two carinae). ***Mesothorax*.** Mesocoxal cavities open, scutellum with posterior edge rounded (arcuate) or pointed (acuminate). Elytra. Integument marked with spots or bands in some. ***Aedeagus*.** Parameres with apical lateral expansions present.

**Summary.** Legs with only tarsomere 3 lobed and head with frons not contrasting orange.

**Genus *Physorhinus* Germar, 1840**

**Habitus.** Body length 6–12 mm. ***Head*.** Hypognathous; gena not broadened anteriorly below eye, not extending spine-like anterior to basal tubercle of mandible (ventral condyle). Antennae with sensory elements beginning on antennomere IV. ***Prothorax*.** Pronotum with hind angle carinae present (single); posterior edge of pronotum without sublateral notches; pronotosternal sutures open; prosternal process not curved upward ≥ 40° in lateral view. ***Mesothorax*.** Mesocoxal cavities not closed; scutellum rounded posterad. Elytra. Integument marked with spots or bands in some. ***Legs*.** Metacoxal plate reaching side. ***Aedeagus*.** Parameres without apical lateral expansions.

**Summary.** Legs with only tarsomere 3 lobed and head with frons orange and pronotum dark brown.

**Tribe Megapenthini**

**Genus *Megapenthes* Kiesenwetter, 1858**

**Habitus.** Body length 4–20 mm. ***Head*.** Supra-antennal carinae joining medially (forming shelf) or reaching anterior edge of head capsule; hypognathous in most; frons without triangular depression; gena not broadened anteriorly below eye in most, not extending spine-like anterior to basal tubercle of mandible (ventral condyle) in most. Antennae not pectinate, sensory elements beginning on antennomere III or IV. ***Prothorax*.** Pronotum with lateral carinae complete, hidden anteriorly in dorsal view, meeting anterior edge of prothorax at ~ 90° in lateral view; hind angle carinae present (single); pronotosternal sutures closed, hypomeral bead present; hypomera with arcuate or angulate concavity on posterior edge near hind angle. Prosternum with sides concave at midlength; prosternal process not curved upward ≥ 40° in lateral view. ***Mesothorax*.** Mesocoxal cavities open; mesoventral cavity without serration along sides. Scutellum with anterior edge not concave. Elytra. Striae present; anterior edge straight to arcuate near humeri in dorsal view, integument marked with spots or bands in some; some with pattern from differences in setal color; setal vestiture even and mainly parallel. ***Legs*.** Metacoxal plate elongate in mesal half; tarsal pads absent, claws simple. ***Aedeagus*.** Each paramere with three or more setae.

**Summary.** Supra-antennal carina complete across frons or reaching anterior edge of head capsule, pronotal lateral carina complete, pronotosternal sutures closed, prosternum with sides concave, prosternal process with ventral apex near dorsal apex (not stair step-like), mesocoxal cavities open, elytra striate, metacoxal plates elongate and claws and tarsi simple. Polyphyletic.

**Tribe Aplastini**

**Habitus.** Body length 6–30 mm. ***Head*.** Supra-antennal carinae fading on frons or absent; labrum notched, gena not broadened anteriorly below eye, not extending spine-like anterior to basal tubercle of mandible (ventral condyle). Antennae pectinate or not. ***Prothorax*.** Pronotum with dorsal punctures uniform sized; lateral carinae meeting anterior edge of prothorax at ~ 90° in lateral view; posterior edge of pronotum without sublateral notches, concavity on posterior edge of hypomeron arcuate. Prosternum with sides concave at midlength; prosternal process curved upward > 40° in lateral view. ***Mesothorax*.** Mesocoxal cavities open; mesoventral cavity without serration along sides. Scutellum with anterior edge not concave. Elytra. Integument unmarked; without pattern from differences in setal color; setal vestiture even and mainly parallel. ***Legs*.** Tarsal pads absent, claws simple. ***Aedeagus*.** Parameres without apical lateral expansions, without setae, each paramere with three or more setae.

**Genus *Aplastus* LeConte, 1859**

**Habitus.** Body length 10–30 mm. ***Head*.** Supra-antennal carinae fading on frons or absent; prognathous in most; frons without triangular depression. Antennae not pectinate, sensory elements beginning on antennomere IV; antennomere III closer in length to II than IV. ***Prothorax*.** Pronotum with punctures simple; pronotal lateral carinae complete or incomplete anteriorly, visible throughout length in dorsal view; hind angle carinae present (single); pronotosternal sutures open or closed; hypomera with concavity on posterior edge near hind angle. ***Mesothorax*.** Scutellum with posterior edge not bilobed. Elytra. Anterior edge straight to arcuate near humeri in dorsal view. ***Legs*.** Metacoxal plate without elongation in mesal half.

**Summary.** Male antennae not pectinate (strongly serrate) with sensory elements beginning on antennomere IV (widespread rough texture and short erect setae), pronotum with setae oriented posterad throughout, lateral carinae visible throughout length in dorsal view, prosternum with sides concave near midlength, scutellum not bilobed posterad. Females unknown.

**Genus *Euthysanius* LeConte, 1853**

**Habitus.** Body length 12–30 mm. ***Head*.** Supra-antennal carinae absent; prognathous. Antennae with 12 antennomeres and pectinate in males, sensory elements beginning on antennomere IV. ***Prothorax*.** Pronotum with hypomeral bead present. ***Mesothorax*.** Scutellum with posterior edge bilobed (concave at midline) or straight (truncate). Elytra. Striae present; anterior edge straight to arcuate near humeri in dorsal view. ***Legs*.** Metacoxal plate without elongation in mesal half, plate not reaching side.

**Summary.** Supra-antennal carinae absent, male antennae pectinate with 12 antennomeres. Females with short elytra and 11 or 12 antennomeres.

**Genus *Octinodes* Candèze, 1863**

**Habitus.** Body length 6–20 mm. ***Head*.** Supra-antennal carinae absent; prognathous; frons without triangular depression. Antennae pectinate in males, sensory elements beginning on antennomere IV. ***Prothorax*.** Pronotum with punctures simple; pronotal lateral carinae complete, visible throughout length in dorsal view; hind angle carinae present (single) or absent; pronotosternal sutures open or closed, hypomeral bead present. ***Mesothorax*.** Scutellum with posterior edge bilobed (concave at midline). Elytra. Striae present; anterior edge straight to arcuate near humeri in dorsal view. ***Legs*.** Metacoxal plate without elongation in mesal half, plate not reaching side.

**Summary.** Lack of supra-antennal carina, antennae pectinate with 11 antennomeres, scale-like setae absent. Females unknown.

**Tribe Cebrionini**

**Habitus.** Body length 6–30 mm. ***Head*.** Supra-antennal carinae absent; gena not broadened anteriorly below eye, not extending spine-like anterior to basal tubercle of mandible (ventral condyle). Antennae not pectinate. ***Prothorax*.** Pronotum with dorsal punctures uniform sized; lateral carinae incomplete anteriorly, also hidden anterad in dorsal view; posterior edge of pronotum without sublateral notches, concavity on posterior edge of hypomeron arcuate; pronotosternal sutures closed. Prosternum with sides concave at midlength. ***Mesothorax*.** Mesocoxal cavities open; mesoventral cavity without serration along sides. Scutellum with anterior edge not concave. Elytra. Integument unmarked; without pattern from differences in setal color; setal vestiture even and mainly parallel. ***Legs*.** Metacoxal plate not reaching side; tarsal pads absent, claws simple. ***Aedeagus*.** Parameres without apical lateral expansions.

**Genus *Scaptolenus* LeConte, 1853**

**Habitus.** Body length 12–30 mm. ***Head*.** Supra-antennal carinae absent; prognathous; frons without triangular depression. Antennae with sensory elements beginning on antennomere IV (evidence: erect hairs). ***Prothorax*.** Pronotum with punctures simple; hind angle carinae absent; hypomeral bead absent; hypomera with concavity on posterior edge near hind angle. ***Mesothorax*.** Scutellum with posterior edge pointed (acuminate). Elytra. Striae absent, anterior edge straight to arcuate near humeri in dorsal view. ***Aedeagus*.** Each paramere with two setae.

**Summary.** Supra-antennal carina absent, protibia with obtuse dorsal digging spine (or widening) near midlength. Also, prothorax with longest setae as long as antennomere I.

**Genus *Selonodon* Latreille, 1834**

**Habitus.** Body length 7–30 mm. ***Head*.** Supra-antennal carinae absent; frons without triangular depression. Antennae with sensory elements beginning on antennomere IV. ***Prothorax*.** Pronotum with punctures umbilicate in some; hind angle carinae present (single) or absent. ***Mesothorax*.** Scutellum with posterior edge rounded (arcuate), pointed (acuminate) or straight (truncate). ***Aedeagus*.** Parameres without setae or each paramere with three or more setae.

**Summary.** Supra-antennal carina absent, prosternum anterad of procoxae > 2× as wide as long, protibia without obtuse dorsal digging spine (or widening) near midlength. Also, prothorax with longest setae shorter than antennomere I.

**Tribe Elaterini**

**Habitus.** Body length 4–40 mm. ***Head*.** Hypognathous or weakly prognathous. Supra-antennal carinae fading on frons or vaguely reaching anterior edge of head capsule; frons without triangular depression; gena broadened anteriorly below eye and extending spine-like anterior to basal tubercle of mandible (ventral condyle) in most. Antennae not pectinate in most, sensory elements beginning on antennomere IV in most. ***Prothorax*.** Pronotum with dorsal punctures uniform sized, simple (not umbilicate) in most; lateral carinae, if complete, meeting anterior edge of prothorax at ~ 90° in lateral view; hind angle carinae present (single); posterior edge of pronotum without sublateral notches in most; hypomera with arcuate concavity on posterior edge near hind angle in most; pronotosternal sutures closed in most. Prosternum with sides concave at midlength. ***Mesothorax*.** Mesoventral cavity without serration along sides. Scutellum with anterior edge not concave. Mesocoxal cavities not closed. Elytra with anterior edge straight to arcuate near humeri in dorsal view, integument unmarked; without pattern from differences in setal color; setal vestiture even and mainly parallel. ***Legs*.** Metacoxal plate reaching side; tarsal pads absent, claws simple. ***Aedeagus*.** Each paramere with three or more setae.

**Genus *Campylomorphus* Jacquelin du Val, 1860**

**Habitus.** Body length 5–10 mm. ***Head*.** Gena not broadened anteriorly below eye, not extending spine-like anterior to basal tubercle of mandible (ventral condyle). Antennae with sensory elements beginning on antennomere III. ***Prothorax*.** Pronotal lateral carinae complete, visible throughout length in dorsal view; hypomeral bead present; prosternal process curved upward > 40° in lateral view. ***Mesothorax*.** Scutellum with posterior edge rounded (arcuate) or pointed (acuminate). Elytra. Striae present. ***Legs*.** Metacoxal plate without elongation in mesal half. ***Aedeagus*.** Parameres with apical lateral expansions present.

**Summary.** Antennal sensory elements beginning on III (widespread rough texture), pronotum with setae directed posterad on anterior half, lateral carinae complete, sublateral notches absent, prosternal sides concave, prosternal process ascending, tarsomeres simple. Previously placed in Dendrometrinae: Prosternini.

**Genus *Diplostethus* Schwarz, 1907**

**Habitus.** Body length 12–35 mm. ***Prothorax*.** Pronotum with punctures umbilicate in some; pronotal lateral carinae complete, visible throughout length in dorsal view; hypomeral bead absent; prosternal process not curved upward ≥ 40° in lateral view. ***Mesothorax*.** Scutellum rounded posterad or pointed (acuminate). Elytra. Striae present or absent. ***Legs*.** Metacoxal plate elongate in mesal half. ***Aedeagus*.** Parameres without apical lateral expansions.

**Summary.** Supra antennal carinae fading on frons or reaching anterior of head capsule, antennomere III closer in length to II than to IV, gena produced spine-like beyond base of mandible, pronotal lateral carina complete anterad and visible throughout length in dorsal view, sublateral notches absent, pronotosternal sutures closed, sides of prosternum concave, mesosternal fossa with sides not ledge-like in lateral view (not projecting below anterior part of mesocoxae) and prosternal process stair-step like in side view.

**Genus *Dolerosomus* Motschulsky, 1859**

**Habitus.** Body length 5–10 mm. ***Head*.** Gena not broadened anteriorly below eye, not extending spine-like anterior to basal tubercle of mandible (ventral condyle) or broadened anteriorly below eye, and extending spine-like anterior to basal tubercle of mandible. ***Prothorax*.** Pronotum with some punctures umbilicate; pronotal lateral carinae complete, hidden anteriorly in dorsal view; posterior edge of pronotum with sublateral notches in some; hypomera without concavity on posterior edge near hind angle in some; prosternal process not curved upward in lateral view. ***Mesothorax*.** Scutellum with posterior edge pointed (acuminate). Elytra. Striae present. ***Legs*.** Metacoxal plate without elongation in mesal half.

**Summary.** Supra-antennal carina reaching anterior edge of head capsule or fading on frons, gena produced beyond base of mandible, pronotosternal sutures closed, prosternal sides concave, scutellum pointed posterad, metacoxal plate not elongate.

**Genus *Elater* Linnaeus, 1758**

**Habitus.** Body length 12–18 mm. ***Head*.** Supra-antennal carinae reaching anterior edge of head capsule. ***Prothorax*.** Pronotal lateral carinae complete or incomplete anteriorly, hidden anteriorly in dorsal view; hypomeral bead present; prosternal process not curved upward ≥ 40° in lateral view. ***Mesothorax*.** Scutellum with posterior edge pointed (acuminate). Elytra. Striae absent. ***Aedeagus*.** Parameres with apical lateral expansions in some.

**Summary.** Body length less than 20 mm, supra-antennal carina reaching anterior of head capsule, pronotum with lateral carina complete but, hidden from above in dorsal view, sides of prosternum concave, elytral striae absent (suggested by linear depressions in some).

**Genus *Orthostethus* Lacordaire, 1857**

**Habitus.** Body length 20–40 mm. ***Head*.** Supra-antennal carinae reaching anterior edge of head capsule. Antennae pectinate or not. ***Prothorax*.** Pronotal lateral carinae complete; hypomeral bead present; prosternal process not curved upward ≥ 40° in lateral view. ***Mesothorax*.** Elytra. Striae absent. ***Legs*.** Metacoxal plate without elongation in mesal half. ***Aedeagus*.** Parameres without apical lateral expansions.

**Summary.** Body length > 20 mm, supra-antennal carina reaching anterior of head capsule, pronotum with lateral carina complete, sides of prosternum concave, mesosternal fossa with sides ledge-like in lateral view (projecting below anterior part of mesocoxae), elytra without striae.

**Genus *Parallelostethus* Schwarz, 1907**

**Habitus.** Body length 12–30 mm. ***Prothorax*.** Pronotal lateral carinae incomplete anteriorly; hypomeral bead present; prosternal process not curved upward ≥ 40° in lateral view. ***Mesothorax*.** Scutellum rounded posterad or bilobed (concave at midline). Elytra. Striae absent. ***Legs*.** Metacoxal plate elongate in mesal half. ***Aedeagus*.** Parameres without apical lateral expansions.

**Summary.** Supra antennal carinae fading on frons or reaching anterior edge of head capsule, pronotal lateral carinae incomplete anterad, mesoventral cavity with sides parallel along entire length, scutellum bilobed posterad, elytral striae absent.

**Genus *Sericus* Eschscholtz, 1829**

**Habitus.** Body length 6–17 mm. ***Prothorax*.** Pronotum with punctures umbilicate in some; pronotal lateral carinae complete, visible throughout length in dorsal view or hidden anterad; pronotosternal sutures open, hypomeral bead present; hypomera in some without concavity on posterior edge near hind angle; prosternal process not curved upward ≥ 40° in lateral view. ***Mesothorax*.** Scutellum narrowly rounded posterad. Elytra. Striae present. ***Legs*.** Metacoxal plate without elongation in mesal half. ***Aedeagus*.** Parameres without apical lateral expansions.

**Summary.** Hypognathous, supra-antennal carina not complete across frons, gena produced spine-like beyond base of mandible, lateral carinae meeting anterior edge of pronotum at right angle, pronotum with most setae on anterior half directed posterad, prosternal sides concave, elytra striate, mesoventral cavity not serrate, metacoxal plate not elongate (slight rounded elongation in some), width at side greater than half width near midline.

**Tribes Pomachiliini, Agriotini, and Synaptini**

**Habitus.** Body length 3–18 mm. ***Head*.** Supra-antennal carinae variable; gena broadened anteriorly below eye, extending spine-like anterior to basal tubercle of mandible (ventral condyle). Antennae not pectinate, sensory elements beginning on antennomere IV. ***Prothorax*.** Pronotum with dorsal punctures uniform sized, setae on anterior half directed posterad; pronotal lateral carinae complete in most; pronotosternal sutures open in most; hind angle carinae present (single); posterior edge of pronotum without sublateral notches in most; hypomeral bead present in most; prosternal process not curved upward ≥ 40° in lateral view. ***Mesothorax*.** Mesoventral cavity with serration along sides in most. Elytra. Striae present; anterior edge straight to arcuate near humeri in dorsal view; without pattern from differences in setal color; setal vestiture even and mainly parallel in most. ***Legs*.** Metacoxal plate reaching side in most; tarsal pads absent in most, claws simple in most.

These three similar and closely related tribes are treated collectively here.

**Genus *Idolus* Desbrochers, 1875**

**Habitus.** Body length 3–8 mm. ***Head*.** Supra-antennal carinae joining medially (forming shelf), becoming vague in some; nasale (head capsule below edge of frontal carina) with outline not concave in lateral view; hypognathous. ***Prothorax*.** Pronotum with some punctures umbilicate; pronotal lateral carinae hidden anteriorly in dorsal view, meeting mesal edge of hypomeron at ~ 30° in lateral view; hypomera with arcuate concavity on posterior edge near hind angle. Prosternum with sides concave at midlength. ***Mesothorax*.** Mesocoxal cavities not closed; mesoventral cavity with serration along sides. Scutellum with anterior edge not concave, posterior edge rounded (arcuate) or pointed (acuminate). Elytra. Integument marked with spots or bands in most. ***Legs*.** Metacoxal plate elongate in mesal half. ***Aedeagus*.** Parameres with apical lateral expansions present; each paramere with two setae.

**Summary.** Supra-antennal carinae complete across frons touching anterior edge of head capsule only at midline in some, lateral carinae meeting anterior edge of pronotum at an acute angle, microserrate mesosternal cavity, and simple claws.

***I.debilis*(LeConte)**

**Habitus.** Body length 3–5 mm. ***Head*.** Supra-antennal carinae joining medially (forming shelf); nasale (head capsule below edge of frontal carina) with outline concave in lateral view. ***Prothorax*.** Pronotum with punctures simple; pronotal lateral carinae meeting anterior edge of prothorax at ~ 90° in lateral view; pronotosternal sutures closed; hypomera with arcuate or angulate concavity on posterior edge near hind angle. Prosternum with sides straight at midlength. ***Mesothorax*.** Mesocoxal cavities open; mesoventral cavity without serration along sides. Scutellum with anterior edge not concave, posterior edge rounded (arcuate). Elytra. Integument marked with spots or transverse bands, or unmarked. ***Legs*.** Metacoxal plate elongate in mesal half. ***Aedeagus*.** Parameres without apical lateral expansions, without setae.

**Summary.** Supra-antennal carinae complete across frons, pronotum with all setae oriented posterad near midlength, pronotosternal sutures straight, mesocoxal cavities open, metacoxal plates elongate, and tarsomeres simple. Like some Megapenthini.

**Genus *Leptoschema* Horn, 1885**

**Habitus.** Body length 12–18 mm. ***Head*.** Supra-antennal carinae joining medially (forming shelf); prognathous. ***Prothorax*.** Pronotum with some punctures umbilicate; pronotal lateral carinae hidden anteriorly in dorsal view, meeting anterior edge of prothorax at ~ 90° in lateral view hypomera with arcuate concavity on posterior edge near hind angle in most specimens. Prosternum with sides concave at midlength. ***Mesothorax*.** Mesocoxal cavities open; mesoventral cavity with serration along sides in most. Scutellum with posterior edge bilobed (concave at midline). Elytra. Integument unmarked. ***Legs*.** Metacoxal plate without elongation in mesal half. ***Aedeagus*.** Parameres with apical lateral expansions present; each with three or more setae.

**Summary.** Supra-antennal carinae complete across frons, lateral carinae meeting anterior edge of pronotum at a right angle, concave prosternal sides, open mesocoxal cavities, short metacoxal plates, and simple tarsi.

**Genus *Agriotes* Eschscholtz, 1829**

**Habitus.** Body length 3–12 mm. ***Head*.** Supra-antennal carinae fading on frons or reaching anterior edge of head capsule; hypognathous. ***Prothorax*.** Pronotum with lateral carinae complete or incomplete anteriorly, hidden anteriorly in dorsal view, approaching mesal edge of hypomeron at ~ 30° in lateral view; posterior edge of pronotum with sublateral notches in some; hypomera with arcuate concavity on posterior edge near hind angle. ***Mesothorax*.** Mesocoxal cavities open; mesoventral cavity with serration along sides in most. Scutellum with anterior edge not concave, posterior edge rounded (arcuate) or pointed (acuminate). Elytra. Integument unmarked, setal vestiture direction and evenness variable. ***Legs*.** Metacoxal plate reaching side or not. ***Aedeagus*.** Parameres with apical lateral expansions present; apices setose.

**Summary.** Supra-antennal carinae reaching anterior edge of head capsule or fading in a few, lateral pronotal carina approaching anterior edge of pronotum at ~ 30°, open mesocoxal cavity, striate elytra, and simple claws.

**Genus *Dalopius* Eschscholtz, 1829**

**Habitus.** Body length 3–8 mm. ***Head*.** Supra-antennal carinae fading on frons (not reaching another structure); hypognathous in most. ***Prothorax*.** Pronotum with some punctures umbilicate; pronotal lateral carinae visible throughout length in dorsal view, meeting anterior edge of prothorax at ~ 90° in lateral view; hypomera with arcuate or angulate concavity on posterior edge near hind angle. Prosternum with sides concave at midlength. ***Mesothorax*.** Mesocoxal cavities open; mesoventral cavity with serration along sides. Elytra with integument unmarked. ***Legs*.** Metacoxal plate elongate in mesal half in some. ***Aedeagus*.** Parameres with apical lateral expansions present; apices setose.

**Summary.** Lateral carinae meeting anterior edge of pronotum at a right angle, carina visible in dorsal view anterad, edges of mesosternal fossa microserrate.

***D.inordinatus*Brown**

**Habitus.** Body length 3–5 mm. ***Head*.** Supra-antennal carinae fading on frons; hypognathous. ***Prothorax*.** Pronotum with some punctures umbilicate; pronotal lateral carinae meeting anterior edge of prothorax at ~ 90° in lateral view; hypomera with arcuate concavity on posterior edge near hind angle. Prosternum with sides concave at midlength. ***Mesothorax*.** Mesocoxal cavities open; mesoventral cavity without serration along sides. Scutellum with anterior edge concave, posterior edge rounded (arcuate) or pointed (acuminate). Elytra. Integument unmarked. ***Legs*.** Metacoxal plate slightly elongate in mesal half. ***Aedeagus*.** Parameres with apical lateral expansions present; each paramere with two or more setae.

**Summary.** Supra-antennal carinae fading on frons, gena produced spine-like beyond base of mandible, lateral carinae of meeting anterior edge of pronotum at right angle, pronotum with most setae on anterior half directed posterad, posterior edge without sublateral notches, prosternal sides concave, mesoventral cavity not serrate, metacoxal plate slightly elongate. Like *Sericus* spp.

**Genus *Glyphonyx* Candèze, 1863**

**Habitus.** Body length 3–8 mm. ***Head*.** Supra-antennal carinae joining medially (forming shelf) or reaching anterior edge of head capsule; nasale (head capsule below edge of frontal carina) with outline not concave in lateral view; hypognathous. ***Prothorax*.** Pronotum with punctures simple; pronotal lateral carinae hidden anteriorly in dorsal view, meeting mesal edge of hypomeron at ~ 30° in lateral view; posterior edge of pronotum with sublateral notches present; hypomera with arcuate concavity on posterior edge near hind angle. Prosternum with sides straight or concave at midlength. ***Mesothorax*.** Mesocoxal cavities not closed; mesoventral cavity with serration along sides. Elytra with integument unmarked with spots or transverse bands. ***Legs*.** Metacoxal plate without elongation in mesal half; tarsal pads present on tarsomere IV only, tarsal claws with 3 or more points. ***Aedeagus*.** Parameres without apical lateral expansions; each paramere with one or two setae.

**Summary.** Lateral pronotal carina meeting anterior edge of pronotum at ~ 30°, sides of mesoventral cavity serrate, and claws with 3–4 points per side.

**Subfamily Pityobiinae**

**Genus *Pityobius* LeConte, 1853**

**Habitus.** Body length 20–40 mm. Scale-like setae absent. ***Head*.** Supra-antennal carinae joining medially (forming shelf); nasale (head capsule below edge of frontal carina) with outline concave in lateral view; prognathous; frons with triangular depression. Antennae with 11 or 12 antennomeres, bipectinate or not, sensory elements beginning on antennomere IV. ***Prothorax*.** Pronotum with dorsal punctures uniform sized, simple, without tubercles between punctures; pronotal lateral carinae complete, meeting anterior edge of prothorax at ~ 90° in lateral view, not serrate; bioluminescent spots absent; hind angle carinae present (single); posterior edge of pronotum without sublateral notches, crenellations absent; pronotosternal sutures closed, hypomeral bead present; hypomera with arcuate concavity on posterior edge near hind angle. Prosternum with sides straight or concave at midlength. ***Mesothorax*.** Mesocoxal cavities open; mesoventral cavity without serration along sides. Scutellum with anterior edge not concave, posterior edge rounded (arcuate). Elytra. Striae present; anterior edge straight to arcuate near humeri in dorsal view, integument unmarked; without pattern from differences in setal color; setal vestiture even and mainly parallel. ***Legs*.** Metacoxal plate without elongation in mesal half, plate reaching side; membranous lobes present on multiple tarsomeres, (I–IV); claws without setae, apex simple. **Ventrites**. Sides not microserrate. ***Aedeagus*.** Parameres with articulation at base, apical lateral expansions present, without setae.

**Summary.** Body length > 20 mm, antennomere III closer in length to II than IV (antennae bipectinate in males), pronotum longer than wide, tarsomeres I–IV with short lobes.

**Subfamily Agrypninae**

**Habitus.** Body length 3–30 mm. ***Prothorax*.** Pronotum crenellations absent. ***Mesothorax*.** Mesoventral cavity without serration along sides. ***Legs*.** Tarsal claws with setae in most, claws simple. **Ventrites**. Sides not microserrate. ***Aedeagus*.** Parameres with articulation at base; each paramere with three or more setae.

**Tribe Pseudomelanactini**

**Habitus.** Body length 20–40 mm. Scale-like setae absent. ***Head*.** Prognathous; frons without triangular depression. Antennae with 11 antennomeres, not pectinate, sensory elements beginning on antennomere IV. ***Prothorax*.** Pronotum with dorsal punctures uniform sized, simple, without tubercles between punctures; pronotal lateral carinae complete, visible throughout length in dorsal view, meeting anterior edge of prothorax at ~ 90° in lateral view, not serrate; bioluminescent spots absent; hind angle carinae present (single); posterior edge of pronotum without sublateral notches, hypomeral bead present; prosternal process not curved upward ≥ 40° in lateral view. ***Mesothorax*.** Mesocoxal cavities open. Elytra. Striae present; anterior edge straight to arcuate near humeri in dorsal view, integument unmarked; without pattern from differences in setal color, apex not spinose. ***Legs*.** Metacoxal plate without elongation in mesal half; tarsal pads absent; claws with setae. ***Aedeagus*.** Parameres with apical lateral expansions present.

**Genus *Anthracalaus* Fairmaire, 1888**

**Habitus.** Body length 20–40 mm. ***Head*.** Supra-antennal carinae fading on frons or reaching anterior edge of head capsule. ***Prothorax*.** Pronotosternal sutures open; hypomera with arcuate or angulate concavity on posterior edge near hind angle. Prosternum with sides straight at midlength. ***Mesothorax*.** Scutellum with posterior edge rounded (arcuate). ***Legs*.** Metacoxal plate reaching side.

**Summary.** Body length > 20 mm, scale-like setae absent, bioluminescent spots absent, pronotosternal sutures open but not excavate, tarsal claws with setae.

**Genus *Lanelater* Arnett, 1952**

**Habitus.** Body length 12–30 mm. ***Head*.** Supra-antennal carinae variable; nasale (head capsule below edge of frontal carina) with outline concave or not in lateral view. ***Prothorax*.** Pronotum pronotosternal sutures excavate (able to contain antennae); hypomeron with posterior edge near hind angle without concavity. Prosternum with sides straight at midlength. ***Mesothorax*.** Elytra with setal vestiture even and mainly parallel.

**Summary.** Body length > 20 mm, scale-like setae absent, pronotosternal sutures excavate.

**Tribe Agrypnini**

**Habitus.** Body length 3–28 mm. Scale-like setae present. ***Head*.** Supra-antennal carinae fading on frons or reaching anterior edge of head capsule. Antennae with 11 antennomeres, not pectinate. ***Prothorax*.** Pronotum with punctures simple; bioluminescent spots absent; posterior edge of pronotum without sublateral notches; pronotosternal sutures excavate (able to contain antennae), hypomeral bead absent. ***Mesothorax*.** Elytra. Integument unmarked; setal vestiture even and mainly parallel, apex not spinose. ***Legs*.** Metacoxal plate reaching side; claws with or without setae.

**Summary.** Combination of scale-like setae and excavate pronotosternal sutures is distinctive.

**Genus *Agrypnus* Eschscholtz, 1829**

**Habitus.** Body length 6–12 mm. ***Head*.** Supra-antennal carinae fading on frons (not reaching another structure); hypognathous. ***Prothorax*.** Pronotum with dorsal punctures uniform sized, without tubercles between punctures; pronotal lateral carinae complete, visible throughout length in dorsal view, not serrate; hind angle carinae absent, hypomera with posterior concavities angulate. Prosternum with sides straight at midlength. ***Mesothorax*.** Mesocoxal cavities closed. Scutellum with anterior edge not concave. Elytra. Striae present; anterior edge sinuate (recurved) near humeri in dorsal view; without pattern from differences in setal color. ***Legs*.** Metacoxal plate without elongation in mesal half; tarsal pads absent; claws with setae. ***Aedeagus*.** Parameres without apical lateral expansions.

**Summary.** Scale-like setae present, supra-antennal carinae fading on frons, pronotosternal sutures excavate, mesocoxal cavities closed.

**Genus *Danosoma* Thompson, 1859**

**Habitus.** Body length 12–18 mm. ***Head*.** Supra-antennal carinae fading on frons or reaching anterior edge of head capsule. Antennal sensory elements beginning on antennomere III or IV. ***Prothorax*.** Pronotum with dorsal punctures uniform sized, without tubercles between punctures; pronotal lateral carinae complete, visible throughout length in dorsal view, not serrate; hind angle carinae absent, hypomera with posterior concavities angulate. Prosternum with sides convex at midlength; prosternal process not curved upward ≥ 40° in lateral view. ***Mesothorax*.** Mesocoxal cavities not closed. Scutellum rounded posterad. Elytra. Striae absent, anterior edge straight to arcuate near humeri in dorsal view; with pattern from differences in setal color. ***Legs*.** Tarsal pads absent; claws without setae.

**Summary.** Body length > 10 mm, scale-like setae present, tarsal claws without setae.

**Genus *Lacon* Laporte, 1838**

**Habitus.** Body length 7–28 mm. ***Head*.** Supra-antennal carinae fading on frons or reaching anterior edge of head capsule. Antennal sensory elements beginning on antennomere III. ***Prothorax*.** Pronotum with dorsal punctures uniform sized, without tubercles between punctures; pronotal lateral carinae complete, not serrate; hind angle carinae present (single) or absent, concavity arcuate. Prosternum with sides straight or convex at midlength; prosternal process not curved upward ≥ 40° in lateral view. ***Mesothorax*.** Mesocoxal cavities not closed. Scutellum with anterior edge concave or not. Elytra. Anterior edge straight to arcuate near humeri in dorsal view; some with pattern from differences in setal color. ***Legs*.** Metacoxal plate without elongation in mesal half; tarsal pads present on tarsomere IV only or absent; claws with setae. ***Aedeagus*.** Parameres with apical lateral expansions present.

**Summary.** Scale-like setae present, pronotosternal sutures excavate, mesepimeron reaching mesocoxal cavities, claws with basal setae.

**Genus *Meristhus* Candèze, 1857**

**Habitus.** Body length 3–5 mm. ***Head*.** Supra-antennal carinae reaching anterior edge of head capsule; hypognathous. Antennal sensory elements beginning on antennomere IV. ***Prothorax*.** Pronotum with dorsal punctures uniform-sized, with tubercles between punctures; pronotal lateral carinae incomplete anteriorly, visible throughout length in dorsal view; hind angle carinae absent, hypomera with posterior concavities angulate. Prosternum with prosternal process not curved upward ≥ 40° in lateral view. ***Mesothorax*.** Mesocoxal cavities closed. Scutellum with anterior edge not concave, posterior edge rounded (arcuate). Elytra without pattern from differences in setal color. ***Legs*.** Tarsal pads absent; claws with setae. ***Aedeagus*.** Parameres with apical lateral expansions present.

**Summary.** Scale-like setae present, scutellum with carina along midline, prothorax with hemispherical tubercles between punctures.

**Genus *Rismethus* Fleutiaux, 1947**

**Habitus.** Body length 3–5 mm. ***Head*.** Supra-antennal carinae reaching anterior edge of head capsule. Antennal sensory elements beginning on antennomere IV. ***Prothorax*.** Pronotum with dorsal punctures of two distinct intermixed sizes (heterogeneous), without tubercles between punctures; pronotal lateral carinae incomplete anteriorly in most, carina visible throughout length in dorsal view, not serrate; hind angle carinae absent, hypomera with posterior concavities angulate. Prosternal process not curved upward ≥ 40° in lateral view. ***Mesothorax*.** Mesocoxal cavities closed. Scutellum with anterior edge not concave, posterior edge rounded (arcuate). Elytra. Striae present; anterior edge sinuate (recurved) or with rectangular projections (crenellate) near humeri in dorsal view; without pattern from differences in setal color. ***Legs*.** Tarsal pads absent. ***Aedeagus*.** Parameres with apical lateral expansions present.

**Summary.** Scale-like setae present, scutellum with carina along midline, prothorax without tubercles between punctures (but some with projecting scales).

**Tribe Hemirhipini**

**Habitus.** Body length 13–35 mm. ***Prothorax*.** Pronotum with dorsal punctures uniform sized, without tubercles between punctures; pronotal lateral carinae complete, visible throughout length in dorsal view, not serrate; bioluminescent spots absent; posterior edge of pronotum without sublateral notches; pronotosternal sutures open or closed but not excavate; hypomera with concavity on posterior edge near hind angle; prosternal process not curved upward ≥ 40° in lateral view. ***Mesothorax*.** Mesocoxal cavities open. Scutellum with anterior edge not concave. Elytra. Striae present, apex not spinose. ***Legs*.** Metacoxal plate reaching side; tarsal pads absent; claws with setae.

**Summary.** Large size, lack of bioluminescent spots, non-excavate, pronotosternal sutures, elytra with either scales or integument color pattern (or both), and presence of setae on tarsal claws are distinctive.

**Genus *Alaus* Eschscholtz, 1829**

**Habitus.** Body length 17–35 mm. Scale-like setae present, not metallic. ***Head*.** Supra-antennal carinae variable; prognathous. Antennae with 11 antennomeres, not pectinate. ***Prothorax*.** Pronotum with punctures simple; pronotosternal sutures closed. ***Mesothorax*.** Scutellum rounded posterad. Elytra. Integument unmarked; with pattern from differences in setal color; vestiture with bare patches in some. ***Legs*.** Metacoxal plate without elongation in mesal half. ***Aedeagus*.** Parameres with apical lateral expansions present.

**Summary.** Body length > 15 mm, pronotosternal sutures closed, posterior edge of scutellum rounded, elytra with non-metallic scales. Most or all with two eye-like dark patches of scales on pronotum.

**Genus *Chalcolepidius* Eschscholtz, 1829**

**Habitus.** Body length 17–35 mm. Scale-like setae present, metallic in some. ***Head*.** Supra-antennal carinae absent; prognathous. Antennae with 11 antennomeres, pectinate or not, sensory elements beginning on antennomere IV. ***Prothorax*.** Pronotum with punctures simple; hind angle carinae absent; hypomeral bead absent, concavity angulate. Prosternum with sides convex at midlength. ***Mesothorax*.** Scutellum with posterior edge bilobed (concave at midline) or straight (truncate). Elytra. Anterior edge sinuate (recurved) near humeri in dorsal view, integument unmarked; some with pattern from differences in setal color; setal vestiture even and mainly parallel. ***Legs*.** Metacoxal plate without elongation in mesal half.

**Summary.** Body length > 15 mm, posterior edge of scutellum bilobed or truncate, and elytra with scales are distinctive. Scales metallic in some.

**Genus *Pherhimius* Fleutiaux, 1942**

**Habitus.** Body length 15–20 mm. Scale-like setae absent. ***Head*.** Supra-antennal carinae joining medially (forming shelf); hypognathous. Antennae with 11 or 12 antennomeres, pectinate, sensory elements beginning on antennomere IV. ***Prothorax*.** Pronotum with some punctures umbilicate; hind angle carinae present (two carinae); pronotosternal sutures open, hypomeral bead present; hypomera with posterior concavities arcuate. ***Mesothorax*.** Elytra with anterior edge straight to arcuate near humeri in dorsal view, integument marked longitudinal and transverse bands, also with pattern from differences in setal color; setal vestiture even and mainly parallel. ***Aedeagus*.** Parameres with apical lateral expansions present.

**Summary.** Body length > 15 mm, antennae pectinate, vestiture not scale-like and elytra with color pattern.

**Tribe Pyrophorini**

**Habitus.** Body length 10–40 mm. Scale-like setae absent. ***Head*.** Supra-antennal carinae joining medially (forming shelf) or reaching anterior edge of head capsule. Antennae with 11 antennomeres, not pectinate, sensory elements beginning on antennomere IV. ***Prothorax*.** Pronotum with dorsal punctures uniform sized, without tubercles between punctures; pronotal lateral carinae complete, visible throughout length in dorsal view, not serrate; bioluminescent spots present; posterior edge of pronotum without sublateral notches; pronotosternal sutures not excavate; hypomera with arcuate concavity on posterior edge near hind angle. Prosternum with sides straight at midlength; prosternal process not curved upward ≥ 40° in lateral view. ***Mesothorax*.** Mesocoxal cavities open. Elytra. Anterior edge straight to arcuate near humeri in dorsal view, integument unmarked; without pattern from differences in setal color; setal vestiture even and mainly parallel. ***Legs*.** Metacoxal plate without elongation in mesal half, plate reaching side; profemur without carina across anterior face; tarsal pads absent; claws with setae.

**Summary.** Bioluminescent organs on pronotum are distinctive.

**Genus *Deilelater* Costa, 1975**

**Habitus.** Body length 12–18 mm. ***Head*.** Supra-antennal carinae joining medially (forming shelf); nasale (head capsule below edge of frontal carina) with outline not concave in lateral view; prognathous. ***Prothorax*.** Pronotum with punctures simple; hind angle carinae present (single) or absent, hypomeral bead present. ***Mesothorax*.** Scutellum with anterior edge not concave, posterior edge rounded (arcuate). Elytra. Striae present, apex not spinose. ***Aedeagus*.** Parameres with apical lateral expansions present.

**Summary.** Antennae reaching beyond midlength of pronotum, pronotum with bioluminescent spots, and punctures simple, elytral apices not spinose.

**Genus *Ignelater* Costa, 1975**

**Habitus.** Body length 17–35 mm. ***Head*.** Supra-antennal carinae joining medially (forming shelf); nasale (head capsule below edge of frontal carina) with outline not concave in lateral view; prognathous; frons without triangular depression. ***Prothorax*.** Pronotum with punctures simple; hind angle carinae present (single). ***Mesothorax*.** Scutellum rounded posterad or pointed (acuminate). Elytra. Striae present, apex spinose. ***Aedeagus*.** Parameres with apical lateral expansions present.

**Summary.** Pronotum with bioluminescent spots, antennae reaching beyond midlength of pronotum, elytral apices spinose.

**Genus *Pyrophorus* Billberg, 1820**

**Habitus.** Body length 20–40 mm. ***Head*.** Supra-antennal carinae joining medially (forming shelf); prognathous; frons without triangular depression. ***Prothorax*.** Pronotum with punctures simple; hind angle carinae present (single); pronotosternal sutures open. ***Mesothorax*.** Scutellum with anterior edge not concave, posterior edge rounded (arcuate). Elytra. Elytral apex spinose. ***Aedeagus*.** Parameres without apical lateral expansions.

**Summary.** Pronotum with bioluminescent spots, antennae not reaching beyond midlength of pronotum. Not established in USA or Canada.

**Genus *Vesperelater* Costa, 1975**

**Habitus.** Body length 20–40 mm. ***Head*.** Supra-antennal carinae joining medially (forming shelf); nasale (head capsule below edge of frontal carina) with outline not concave in lateral view; prognathous. ***Prothorax*.** Pronotum with some punctures umbilicate; hind angle carinae present (single); hypomeral bead present. ***Mesothorax*.** Scutellum with anterior edge not concave, posterior edge rounded (arcuate). Elytra. Striae present, apex not spinose. ***Aedeagus*.** Parameres with apical lateral expansions present.

**Summary.** Antennae reaching beyond midlength of pronotum, pronotum with bioluminescent spots, and punctures umbilicate, elytral apices not spinose.

**Tribe Oophorini**

**Habitus.** Body length 3–12 mm. Scale-like setae absent. ***Head*.** Supra-antennal carinae joining medially (forming shelf) in most; frons without triangular depression. Antennae with 11 antennomeres, not pectinate, sensory elements beginning on antennomere IV. ***Prothorax*.** Pronotum with punctures simple, without tubercles between punctures; pronotal lateral carinae complete, not serrate; bioluminescent spots absent; pronotosternal sutures not excavate. ***Mesothorax*.** Scutellum with anterior edge not concave. Elytra. Striae present, elytra with anterior edge straight to arcuate near humeri in dorsal view, integument marked with spots or bands in many; setal vestiture even and mainly parallel. ***Legs*.** Metacoxal plate reaching side in most; tarsal pads present on tarsomere IV in most (not on any others); claws with setae in most.

Small to moderate size, lack of scale-like setae and bioluminescent spots, non-excavate pronotosternal sutures and presence of setae on tarsal claws are distinctive. Setae on claws are difficult to observe on small specimens (not to be confused with setae projecting between the claw bases). Most or all with striae not merging before elytral apex.

**Genus *Aeolus* Eschscholtz, 1829**

**Habitus.** Body length 3–10 mm. ***Head*.** Nasale (head capsule below edge of frontal carina) with outline concave in lateral view. ***Prothorax*.** Pronotum with dorsal punctures uniform sized; hind angle carinae present (single); posterior edge of pronotum without sublateral notches; pronotosternal sutures closed, hypomeral bead present; hypomera with concavity on posterior edge near hind angle. Prosternum with sides straight at midlength; prosternal process not curved upward ≥ 40° in lateral view. ***Mesothorax*.** Mesocoxal cavities not closed; scutellum rounded posterad. Elytra. Integument marked with spots or bands in many; without pattern from differences in setal color. ***Legs*.** Metacoxal plate elongate in mesal half; profemur with carina across anterior face (basidorsal to apicoventral); claws with or without setae. ***Aedeagus*.** Parameres without apical lateral expansions.

**Summary.** Profemur with carina across anterior face is distinctive. Most with body orange with darker markings.

**Genus *Deronocus* Johnson, 1995**

**Habitus.** Body length 6–12 mm. ***Head*.** Supra-antennal carinae fading on frons or reaching anterior edge of head capsule; prognathous. ***Prothorax*.** Pronotum with dorsal punctures uniform sized, lateral carinae hidden anteriorly in dorsal view; hind angle carinae present (single); posterior edge of pronotum with sublateral notches present; pronotosternal sutures open, hypomeral bead present; hypomera with arcuate or angulate concavity on posterior edge near hind angle. Prosternum with sides straight at midlength; prosternal process not curved upward ≥ 40° in lateral view. ***Mesothorax*.** Mesocoxal cavities open, scutellum rounded posterad. Elytra. Integument unmarked; without pattern from differences in setal color. ***Legs*.** Metacoxal plate without elongation in mesal half, plate reaching side; profemur without carina across anterior face; tarsal pads absent. ***Aedeagus*.** Parameres with apical lateral expansions present.

**Summary.** Size moderate (6–12 mm), scale-like setae and bioluminescent spots absent, pronotal lateral carina hidden anterad in dorsal view, pronotosternal sutures open (but not excavate), simple tarsomeres, tarsal claws with setae.

**Genus *Heteroderes* Latreille, 1834**

**Habitus.** Body length 5–10 mm. ***Head*.** Prognathous. ***Prothorax*.** Pronotum with dorsal punctures of two distinct intermixed sizes (heterogeneous), carina visible throughout length in dorsal view or hidden anterad; hind angle carinae present (one or two carinae); posterior edge of pronotum with sublateral notches in some; pronotosternal sutures open, hypomeral bead absent; hypomera with arcuate or angulate concavity on posterior edge near hind angle. Prosternum with sides straight or concave at midlength. ***Mesothorax*.** Mesocoxal cavities open, scutellum rounded posterad or pointed (acuminate). Elytra. Integument marked with spots or bands in some; without pattern from differences in setal color. ***Legs*.** Profemur without carina across anterior face. ***Aedeagus*.** Parameres with apical lateral expansions present.

**Summary.** Scale-like setae absent; hypomeral bead present, legs with profemur non-carinate, tarsomere IV lobed, tarsal claws setose.

**Genus *Monocrepidius* Eschscholtz, 1829**

**Habitus.** Body length 3–12 mm. ***Head*.** Nasale (head capsule below edge of frontal carina) with outline concave in lateral view. ***Prothorax*.** Pronotum with dorsal punctures uniform sized, lateral carinae visible throughout length in dorsal view or hidden anterad; hind angle carinae present (one or two carinae) or absent; hypomeral bead present; hypomera with posterior concavities present. Prosternal process not curved upward ≥ 40° in lateral view. ***Mesothorax*.** Mesocoxal cavities not closed. Elytra. Integument marked with spots or bands in some, some with pattern from differences in setal color. ***Legs*.** Profemur without carina across anterior face.

**Summary.** Scale-like setae absent; hypomeral bead present, legs with profemur non-carinate, tarsomere IV lobed, tarsal claws setose. Genus *Conoderus* Eschscholtz, 1829 is treated as a synonym here.

**Subfamily Hypnoidinae**

**Habitus.** Body length 3–12 mm. ***Head*.** Prognathous. Antennae with 11 antennomeres, not pectinate. ***Prothorax*.** Pronotum with punctures simple, without tubercles between punctures; pronotal lateral carinae complete, visible throughout length in dorsal view, meeting anterior edge of prothorax at ~ 90° in lateral view, not serrate; bioluminescent spots absent; hind angle carinae present (single), crenellations absent; pronotosternal sutures closed; hypomera with arcuate concavity on posterior edge near hind angle; prosternal process not curved upward ≥ 40° in lateral view. ***Mesothorax*.** Mesocoxal cavities not closed; mesoventral cavity without serration along sides. Scutellum with anterior edge not concave. Elytra. Striae present; anterior edge straight to arcuate near humeri in dorsal view, integument unmarked or marked with spot or band in apical half only; without pattern from differences in setal color; setal vestiture even and mainly parallel, apex not spinose. ***Legs*.** Tarsal pads absent; claws without setae, apex simple. **Ventrites**. Sides not microserrate. ***Aedeagus*.** Parameres with articulation at base.

**Summary.** Pronotosternal sutures closed, tarsi and claws simple (not distinctive). All, except arctic genus *Hypolithus*, have mesocoxal cavities open to mesepimeron only. All, except some *Ligmargus* and *Margaiostus* have the supra-antennal carinae joining medially. Most 10 mm long or less and with sublateral plicae and notches present. Identification of this group can be difficult at all levels.

**Genus *Ascoliocerus* Méquignon, 1930**

**Habitus.** Body length 3–10 mm. Setae not thickened at midlength. ***Head*.** Supra-antennal carinae joining medially (forming shelf); nasale (head capsule below edge of frontal carina) with outline concave in lateral view. Antennal sensory elements beginning on antennomere IV. ***Prothorax*.** Pronotum with dorsal punctures uniform sized; posterior edge of pronotum with sublateral notches present, hypomeral bead absent. ***Mesothorax*.** Mesocoxal cavities open to mesepimeron only; scutellum rounded posterad. Elytra. Integument unmarked. ***Legs*.** Metacoxal plate elongate in mesal half, plate not reaching side. ***Aedeagus*.** Parameres with articulation at base, apical lateral expansions absent; each paramere with three or more setae.

**Summary.** Supra-antennal carinae joining medially, antennae with sensory elements beginning on antennomere IV (dense sculpture and short erect setae), pronotum with most setae on anterior half directed posterad or mesad, hypomeral bead absent, mesocoxal cavities partly open, tarsi simple, male parameres with three or more setae. Also, pronotal hind angles and posterior part near midline with short pale setae in addition to longer setae.

**Genus *Berninelsonius* Leseigneur, 1970**

**Habitus.** Body length 5–10 mm. Setae not thickened at midlength. ***Head*.** Supra-antennal carinae joining medially (forming shelf); nasale (head capsule below edge of frontal carina) with outline not concave in lateral view. Antennal sensory elements beginning on antennomere IV. ***Prothorax*.** Pronotum with dorsal punctures uniform sized; posterior edge of pronotum with sublateral notches present, hypomeral bead absent. Prosternum with sides convex at midlength. ***Mesothorax*.** Mesocoxal cavities open to mesepimeron only; scutellum with posterior edge rounded (arcuate). Elytra. Integument unmarked. ***Aedeagus*.** Parameres with articulation at base, apical lateral expansions absent, without setae.

**Summary.** Supra-antennal carinae joining medially, antennae with sensory elements beginning on antennomere IV (apical sensorium), pronotum with most setae on anterior half directed posterad, hypomeral bead absent, tarsi simple, parameres without setae.

**Genus *Desolakerrus* Stibick, 1978**

**Habitus.** Body length 5–10 mm. Setae not thickened at midlength. ***Head*.** Supra-antennal carinae joining medially (forming shelf). Antennal sensory elements beginning on antennomere IV. ***Prothorax*.** Pronotum with dorsal punctures of two distinct intermixed sizes (heterogeneous); posterior edge of pronotum without sublateral notches, hypomeral bead present. ***Mesothorax*.** Mesocoxal cavities open to mesepimeron only; scutellum rounded posterad. Elytra. Integument unmarked. ***Legs*.** Metacoxal plate elongate in mesal half, plate not reaching side. ***Aedeagus*.** Parameres with articulation at base, apical lateral expansions absent; each paramere with three or more setae.

**Summary.** Elongate bodied, pronotum with setae directed anterad, mesocoxal cavities open to mesepimeron only, hypomeron metacoxal plates elongate medially. Also, hypomeron with larger punctures elongate.

**Genus *Hypnoidus* Dillwyn, 1829**

**Habitus.** Body length 3–10 mm. Setae thickened at midlength on scutellum in some. ***Head*.** Supra-antennal carinae joining medially (forming shelf). Antennal sensory elements beginning on antennomere IV or V. ***Prothorax*.** Pronotum with dorsal punctures uniform sized, with or without tubercles between punctures; posterior edge of pronotum with sublateral notches present, hypomeral bead absent. ***Mesothorax*.** Mesocoxal cavities open to mesepimeron only; scutellum rounded posterad. Elytra. Integument unmarked or marked with spot or band in apical half only. ***Legs*.** Metacoxal plate elongate in mesal half, plate not reaching side. ***Aedeagus*.** Parameres with articulation at base; each paramere with one seta.

**Summary.** Supra-antennal carinae joining medially, antennae with sensory elements beginning on antennomere IV or V (apical sensorium), hypomeral bead absent, tarsi simple, each paramere with one seta. We could not write a diagnosis that distinguished all specimens from *Margaiostus*.

**Genus *Hypolithus* Eschscholtz, 1829**

**Habitus.** Body length 6–12 mm. Setae not thickened at midlength. ***Head*.** Supra-antennal carinae joining medially (forming shelf); nasale (head capsule below edge of frontal carina) with outline not concave in lateral view. Antennal sensory elements beginning on antennomere IV. ***Prothorax*.** Pronotum with dorsal punctures uniform sized, without tubercles between punctures; posterior edge of pronotum without sublateral notches, hypomeral bead absent. Prosternum with sides straight at midlength. ***Mesothorax*.** Mesocoxal cavities open, scutellum rounded posterad. Elytra. Integument unmarked. ***Legs*.** Metacoxal plate without elongation in mesal half, plate not reaching side. ***Aedeagus*.** Parameres with articulation at base, apical lateral expansions present; each paramere with three or more setae.

**Summary.** Supra-antennal carinae joining medially, pronotum dark with pale sides, pronotal sublateral notches absent, hypomeral bead absent, mesocoxal cavities open, metacoxal plate not reaching side of metacoxa. Also, hindwings short.

**Genus *Ligmargus* Stibick, 1976**

**Habitus.** Body length 6–12 mm. Setae not thickened at midlength. ***Head*.** Supra-antennal carinae joining medially (forming shelf) or reaching anterior edge of head capsule; nasale (head capsule below edge of frontal carina) with outline not concave in lateral view. Antennal sensory elements beginning on antennomere III or IV. ***Prothorax*.** Pronotum with dorsal punctures of two distinct intermixed sizes (heterogeneous), with or without tubercles between punctures; posterior edge of pronotum with sublateral notches present, hypomeral bead absent. ***Mesothorax*.** Mesocoxal cavities open to mesepimeron only. Elytra. Integument unmarked. ***Legs*.** Metacoxal plate not reaching side. ***Aedeagus*.** Parameres with articulation at base or at midlength, without setae or each paramere with three or more setae.

**Summary.** Supra-antennal carinae joining medially, pronotum with two size classes of intermixed punctures, posterior slope of pronotum with setae directed posterad, pronotosternal sutures closed, mesocoxal cavities open to mesepimeron only. Also, pronotum with dense longitudinal sculpture around punctures.

**Genus *Margaiostus* Stibick, 1978**

**Habitus.** Body length 6–12 mm. Setae not thickened at midlength. ***Head*.** Supra-antennal carinae joining medially (forming shelf) or reaching anterior edge of head capsule; nasale (head capsule below edge of frontal carina) with outline not concave in lateral view. Antennal sensory elements beginning on antennomere IV or V. ***Prothorax*.** Pronotum with dorsal punctures uniform sized; posterior edge of pronotum with sublateral notches present, hypomeral bead absent. ***Mesothorax*.** Mesocoxal cavities open to mesepimeron only; scutellum rounded posterad. Elytra. Integument unmarked. ***Legs*.** Metacoxal plate elongate in mesal half, plate not reaching side. ***Aedeagus*.** Parameres with articulation at base, apical lateral expansions present; each paramere with one or two setae.

**Summary.** Body length 6–12 mm. Supra-antennal carinae joining medially, antennae with sensory elements beginning on antennomere IV or V (apical sensorium), tarsi simple, aedeagus with one seta. We could not write a diagnosis that distinguished all specimens from *Hypnoidus*.

**Subfamily Negastriinae**

**Habitus.** Body length 3–6 mm. Setae not thickened at midlength. ***Head*.** Frons without triangular depression. Antennae with 11 antennomeres, not pectinate. ***Prothorax*.** Pronotal lateral carinae complete, meeting anterior edge of prothorax at ~ 90° in lateral view, not serrate; bioluminescent spots absent; hind angle carinae present (single); posterior edge of pronotum without sublateral notches, crenellations absent; hypomera with concavity on posterior edge near hind angle; pronotosternal sutures closed. Prosternum with sides convex at midlength; prosternal process not curved upward ≥ 40° in lateral view. ***Mesothorax*.** Mesocoxal cavities closed; mesoventral cavity without serration along sides. Elytra. Striae present; integument marked with spots or bands in some; without pattern from differences in setal color; setal vestiture even and mainly parallel, apex not spinose. ***Legs*.** Tarsal pads absent; claws without setae, apex simple, appendiculate, or bifid. **Ventrites**. Sides not microserrate. ***Aedeagus*.** Parameres with articulation at midlength, fused at base, apical lateral expansions absent.

**Summary.** Small beetles. Wide prosternum and closed mesocoxal cavities are diagnostic in combination with simple setae. Most like Cardiophorinae, Hypnoidinae, and some Agrypnini.

**Genus *Fleutiauxellus* Méquignon, 1930**

**Habitus.** Body length 2–6 mm. ***Head*.** Prognathous. Antennal sensory elements beginning on antennomere III. ***Prothorax*.** Pronotum with dorsal punctures uniform sized, simple, antero-medial portion with or without tubercles between punctures; pronotal lateral carinae visible throughout length in dorsal view, hypomeral bead present; hypomera with posterior concavities arcuate. ***Mesothorax*.** Elytra. Striae present; anterior edge straight to arcuate near humeri in dorsal view, integument unmarked. ***Legs*.** Tarsal claws simple. ***Aedeagus*.** Each paramere with three or more setae.

**Summary.** Small size, pronotum not strigose or tuberculate between punctures, prosternum with sides convex, mesocoxal cavities closed (barely in some), claws simple, parameres each with three or more setae (two in *Neohypdonus*).

**Genus *Microhypnus* Kishii, 1976**

**Habitus.** Body length 3–5 mm. ***Head*.** Supra-antennal carinae joining medially (forming shelf); prognathous. Antennal sensory elements beginning on antennomere IV. ***Prothorax*.** Pronotum with dorsal punctures uniform sized, simple, with elongate tubercles between punctures; pronotal lateral carinae visible throughout length in dorsal view, hypomeral bead present. ***Mesothorax*.** Scutellum with anterior edge not concave. Elytra. Striae present; anterior edge straight to arcuate near humeri in dorsal view, integument unmarked. ***Legs*.** Metacoxal plate elongate in mesal half, plate reaching side, tarsal claws simple. ***Aedeagus*.** Each paramere with two setae.

**Summary.** Small size, elytron without contrasting spots or bands, pronotum with elongate tubercles between punctures, prosternum with sides convex, mesocoxal cavities closed, claws simple. Incompletely distinguished from *Negastrius*.

**Genus *Migiwa* Kishii, 1966**

**Habitus.** Body length 3–5 mm. ***Head*.** Supra-antennal carinae joining medially (forming shelf); hypognathous. Antennal sensory elements beginning on antennomere IV. ***Prothorax*.** Pronotum with dorsal punctures uniform sized, some or all punctures umbilicate, without tubercles between punctures; pronotal lateral carinae hidden anteriorly in dorsal view, hypomeral bead present; hypomera with posterior concavities arcuate. ***Mesothorax*.** Scutellum with anterior edge concave, posterior edge rounded (arcuate). Elytra. Striae present; integument unmarked with spots or transverse bands. ***Legs*.** Metacoxal plate elongate in mesal half, plate reaching side, tarsal claws simple. ***Aedeagus*.** Each paramere with three or more setae.

**Summary.** Prothorax with umbilicate punctures (requires 80X magnification), prosternum with sides convex, mesocoxal cavities closed, metacoxal plate reaching side.

**Genus *Negastrius* Thomson, 1859**

**Habitus.** Body length 3–5 mm. ***Head*.** Supra-antennal carinae joining medially (forming shelf); prognathous. Antennal sensory elements beginning on antennomere IV. ***Prothorax*.** Pronotum with dorsal punctures uniform sized, simple, with longitudinal carinae between punctures; pronotal lateral carinae complete or incomplete anteriorly, visible throughout length in dorsal view, hypomeral bead present; hypomera with posterior concavities arcuate. ***Mesothorax*.** Elytra. Striae present integument marked with spots or bands in many. ***Legs*.** Metacoxal plate elongate in mesal half, plate reaching side, tarsal claws simple. ***Aedeagus*.** Each paramere with two setae.

**Summary.** Small size, pronotum strigose between punctures, prosternum with sides convex, mesocoxal cavities closed. Most with elytra bicolored, and evenly convex in lateral view.

**Genus *Neohypdonus* Stibick, 1971**

**Habitus.** Body length 2–5 mm. ***Head*.** Supra-antennal carinae variable. Antennal sensory elements beginning on antennomere III or IV. ***Prothorax*.** Pronotum with dorsal punctures all simple, without tubercles between punctures. ***Mesothorax*.** Elytra. Integument marked with spots or bands in some. ***Legs*.** Metacoxal plate elongate in mesal half, tarsal claws simple. ***Aedeagus*.** Each paramere with two setae.

**Summary.** Small size, pronotum not strigose or tuberculate between punctures (ambiguous in *N.musculus* (Erichson)), prosternum with sides convex, mesocoxal cavities closed, claws simple, parameres each with two setae (three or more in *Fleutiauxellus*). Elytral striae weak near apex.

**Genus *Oedostethus* LeConte, 1853**

**Habitus.** Body length 3–5 mm. ***Head*.** Supra-antennal carinae joining medially (forming shelf); prognathous. Antennal sensory elements beginning on antennomere IV. ***Prothorax*.** Pronotum with dorsal punctures uniform sized, simple, without tubercles between punctures; hypomeral concavity arcuate. ***Mesothorax*.** Scutellum with anterior edge concave. Elytra. Striae present; anterior edge sinuate (recurved), with projection near humeri in dorsal view, integument unmarked with spots or transverse bands. ***Legs*.** Metacoxal plate reaching side, tarsal claws appendiculate. ***Aedeagus*.** Each paramere with two setae.

**Summary.** Small size, sides of prothorax convex, and claws appendiculate or at least thickened at base.

**Genus *Paradonus* Stibick, 1971**

**Habitus.** Body length 1–4 mm. ***Head*.** Supra-antennal carinae joining medially (forming shelf). Antennal sensory elements beginning on antennomere IV. ***Prothorax*.** Pronotum with dorsal punctures uniform sized, simple, without tubercles between punctures; pronotosternal sutures open, hypomeral bead present; hypomera with posterior concavities arcuate. ***Mesothorax*.** Scutellum with anterior edge concave, posterior edge pointed (acuminate). Elytra. Striae absent, anterior edge sinuate (recurved) with projection near humeri in dorsal view, integument marked with spots or transverse bands, or unmarked. ***Legs*.** Tarsal claws simple. ***Aedeagus*.** Each paramere with one seta.

**Summary.** Small size, sides of prothorax convex and elytral striae absent.

**Genus *Zorochros* Thomson, 1859**

**Habitus.** Body length 2–5 mm. ***Head*.** Supra-antennal carinae joining medially (forming shelf). Antennal sensory elements beginning on antennomere IV. ***Prothorax*.** Pronotum with antero-medial portion with or without tubercles between punctures; hypomeral bead present. ***Mesothorax*.** Scutellum with anterior edge concave. Elytra. Striae present; integument marked with spots or transverse bands, or unmarked. ***Legs*.** Metacoxal plate elongate in mesal half, plate not reaching side, tarsal claws simple. ***Aedeagus*.** Each paramere with three or more setae.

**Summary.** Small size, sensory elements beginning on antennomere IV (antennomere III cylindrical), sides of prosternum convex, mesocoxal cavities closed, elytra with striae present, metacoxal plate not reaching side (most). Most with tubercles on anterior half of pronotum near midline.

**Subfamily Cardiophorinae**

**Habitus.** Body length 3–12 mm. Scale-like setae absent. ***Head*.** Supra-antennal carinae joining medially (forming shelf); nasale (head capsule below edge of frontal carina) with outline concave in lateral view. Antennae with 11 antennomeres, not pectinate. ***Prothorax*.** Pronotum with sides not serrate; posterior crenellations absent; bioluminescent spots absent; without tubercles or ridges between punctures (but punctures on tubercles in *Floridelater*) hypomera with concavity on posterior edge near hind angle. Prosternum with sides straight or concave at midlength. ***Mesothorax*.** Mesocoxal cavities closed; mesoventral cavity without serration along sides. Scutellum with anterior edge concave in most (notched in many), posterior edge pointed (acuminate) in most or bilobed (concave at midline). Elytra. Striae present; integument marked with spots or bands in some; setal vestiture even and mainly parallel, apex not spinose. ***Legs*.** Tarsal claws without setae. ***Aedeagus*.** Parameres with articulation at midlength, fused at base.

Mid-sized beetles. Non-convex sides of prosternum, closed mesocoxal cavities and absence of scale-like setae are distinctive.

**Genus *Aphricus* LeConte, 1853**

**Habitus.** Body length 5–10 mm. ***Head*.** Hypognathous. Antennal sensory elements beginning on antennomere III. ***Prothorax*.** Pronotum with dorsal punctures uniform sized, simple; pronotal lateral carinae incomplete anteriorly; posterior edge of pronotum without sublateral notches; pronotosternal sutures open, hypomeral bead absent. Prosternal process curved upward > 40° in lateral view. ***Mesothorax*.** Scutellum with anterior edge concave, posterior edge pointed (acuminate). Elytra. Anterior edge straight to arcuate near humeri in dorsal view, integument unmarked; without pattern from differences in setal color. ***Legs*.** Metacoxal plate without elongation in mesal half; tarsal pads absent, claws simple. **Ventrites**. Sides not microserrate. ***Aedeagus*.** Each paramere with three or more setae.

**Summary.** Mesocoxal cavities closed, scutellum pointed posterad, claws simple, parameres each with three or more setae. Most or all with mandibles simple and antennae reaching beyond scutellum. Females unknown.

**Genus *Aptopus* Eschscholtz, 1829**

**Habitus.** Body length 6–12 mm. ***Head*.** Hypognathous. Antennal sensory elements beginning on antennomere III in most. ***Prothorax*.** Pronotum with dorsal punctures of two distinct intermixed sizes (heterogeneous), all simple; pronotal lateral carinae incomplete anteriorly, hidden in dorsal view; hind angle carinae present (single); posterior edge of pronotum with sublateral notches present; pronotosternal sutures closed. ***Mesothorax*.** Scutellum with anterior edge notched, posterior edge pointed (acuminate). Elytra. Integument unmarked; without pattern from differences in setal color. ***Legs*.** Metacoxal plate elongate in mesal half, plate not reaching side; tarsal pads absent, tarsal claws more than three points. ***Aedeagus*.** Each paramere with two setae.

**Summary.** Scutellum shaped like valentine heart, claws pectinate (6 or 7 apices per side).

**Genus *Cardiophorus* Eschscholtz, 1829**

**Habitus.** Body length 6–12 mm. ***Head*.** Antennal sensory elements beginning on antennomere III. ***Prothorax*.** Pronotum with dorsal punctures of two distinct intermixed sizes (heterogeneous), simple; pronotal lateral carinae incomplete anteriorly, hidden in dorsal view; hind angle carinae present (single); posterior edge of pronotum with sublateral notches present; pronotosternal sutures closed, hypomera with posterior concavity angulate. ***Mesothorax*.** Scutellum with anterior edge notched, posterior edge pointed (acuminate). Elytra. Anterior edge sinuate (recurved) or with rectangular projection near humeri in dorsal view, integument marked with humeral spots or bands in some; some with pattern from differences in setal color. ***Legs*.** Metacoxal plate not reaching side; tarsal pads absent, claws simple. **Ventrites**. Microserration at sides (e.g., 100 points per mm) present or absent. ***Aedeagus*.** Each paramere with one or two setae.

**Summary.** Scutellum shaped like valentine heart, pronotal lateral carinae incomplete (or absent), if present then visible from ventral view but not dorsal view, hypomera with posterior emargination angulate, claws simple.

**Genus *Esthesopus* Eschscholtz, 1829**

**Habitus.** Body length 3–10 mm. ***Head*.** Hypognathous. Antennal sensory elements beginning on antennomere III or IV. ***Prothorax*.** Pronotum with dorsal punctures of two distinct intermixed sizes (heterogeneous), all simple; pronotal lateral carinae incomplete anteriorly in most; hind angle carinae present (single) or absent; posterior edge of pronotum without sublateral notches; pronotosternal sutures closed. ***Mesothorax*.** Scutellum with posterior edge pointed (acuminate). Elytra. Integument marked with spots or bands in some; without pattern from differences in setal color. ***Legs*.** Metacoxal plate not reaching side; tarsal pads present on tarsomere IV, tarsal claws appendiculate. **Ventrites**. Microserration at sides (e.g., 100 points per mm) present. ***Aedeagus*.** Parameres without apical lateral expansions; each paramere with two setae.

**Summary.** Scutellum pointed posterad, mesocoxal cavities closed, tarsomere IV lobed, claws appendiculate.

**Genus *Floridelater* Douglas, 2017**

**Habitus.** Body length 3–10 mm. ***Head*.** Hypognathous. Antennal sensory elements beginning on antennomere III. ***Prothorax*.** Pronotum with dorsal punctures uniform sized, simple, with punctures situated on tubercles; pronotal lateral carinae incomplete anteriorly, hidden in dorsal view; hind angle carinae present (single); posterior edge of pronotum with sublateral notches present; pronotosternal sutures open, hypomeral bead present. Prosternal process curved upward > 40° in lateral view. ***Mesothorax*.** Scutellum with anterior edge concave, posterior edge bilobed (concave at midline). Elytra. Anterior edge straight to arcuate near humeri in dorsal view, integument unmarked; without pattern from differences in setal color. ***Legs*.** Metacoxal plate without elongation in mesal half; tarsal pads absent, claws simple. **Ventrites**. Sides not microserrate. ***Aedeagus*.** Each paramere with two setae.

**Summary.** Pronotum with punctures situated on tubercles. Also, flight wings short (rudimentary), elytron short (~ 2.3× longer than pronotum in dorsal view) and evenly arched in lateral view.

**Genus *Horistonotus* Candèze, 1860**

**Habitus.** Body length 3–10 mm. ***Head*.** Hypognathous. Antennal sensory elements beginning on antennomere III or IV. ***Prothorax*.** Pronotum with dorsal punctures of two distinct intermixed sizes (heterogeneous); pronotal lateral carinae incomplete anteriorly; hind angle carinae not distinct from lateral carinae; pronotosternal sutures closed, hypomera with posterior concavity angulate. Prosternal process not curved upward ≥ 40° in lateral view. ***Mesothorax*.** Scutellum with anterior edge concave or not, posterior edge pointed (acuminate). Elytra. Anterior edge sinuate (recurved) or with rectangular projection near humeri in dorsal view, integument marked with spots or bands in some; without pattern from differences in setal color. ***Legs*.** Metacoxal plate not reaching side; tarsal pads absent, tarsal claw apices appendiculate, or bifid in most, simple in a few. **Ventrites**. Microserration at sides (e.g., 100 points per mm) present. ***Aedeagus*.** Parameres without apical lateral expansions; each paramere with two setae.

**Summary.** Pronotal lateral carinae not reaching anterior edge of pronotum, hind angle carinae absent, scutellum pointed posterad, mesocoxal cavities closed.

**Genus *Paracardiophorus* Schwarz, 1895**

**Habitus.** Body length 6–12 mm. ***Head*.** Antennal sensory elements beginning on antennomere III. ***Prothorax*.** Pronotum with dorsal punctures uniform sized or of two distinct intermixed sizes (heterogeneous), all simple; pronotal lateral carinae incomplete anteriorly, hidden in dorsal view; hind angle carinae present (single); posterior edge of pronotum with sublateral notches present; pronotosternal sutures closed, hypomeral bead present; hypomera with posterior concavities arcuate. ***Mesothorax*.** Scutellum with anterior edge notched, posterior edge pointed (acuminate). Elytra. Anterior edge straight to arcuate near humeri in dorsal view, integument marked with spots or bands in many; without pattern from differences in setal color. ***Legs*.** Metacoxal plate elongate in mesal half, plate not reaching side; tarsal pads absent, claws simple. **Ventrites**. Sides not microserrate. ***Aedeagus*.** Parameres without apical lateral expansions; each paramere with one or two setae.

**Summary.** Scutellum shaped like valentine heart, pronotal lateral carinae incomplete, visible from ventral view but not dorsal view, hypomera with posterior emarginations arcuate, claws simple.

**Subfamily Dendrometrinae**

**Habitus.** Body length 3–35 mm. ***Vestiture*.** Scale-like setae absent. ***Prothorax*.** Pronotum with lateral carinae meeting anterior edge of prothorax at ~ 90° in lateral view; bioluminescent spots absent; pronotal punctures not of two discrete size classes, and without associated tubercles or carinae in most. ***Mesothorax*.** Mesoventral cavity without serration along sides; mesocoxal cavities not closed by juncture of mesoventrite and metaventrite laterad of cavities. Elytra. Apex not spinose. ***Legs*.** Metacoxal plate without elongation in mesal half; claws without setae. ***Aedeagus*.** Parameres with articulation at base.

This group is defined based on evidence from larvae and DNA. It is difficult to define based on adult morphology.

**Tribe Oxynopterini**

**Habitus.** Body length 17–35 mm. ***Head*.** Prognathous; frons without triangular depression. Antennae not pectinate. ***Prothorax*.** Pronotum with punctures simple, without tubercles between punctures; pronotal lateral carinae complete, visible throughout length in dorsal view, not serrate; posterior edge of pronotum without sublateral notches, crenellations present or absent; hypomera with concavity on posterior edge near hind angle; pronotosternal sutures closed. Prosternum with sides straight at midlength. ***Mesothorax*.** Scutellum with anterior edge not concave. Elytra with anterior edge straight to arcuate near humeri in dorsal view; without pattern from differences in setal color. ***Legs*.** Tarsal pads absent, claws simple. **Ventrites**. Sides not microserrate.

**Summary.** Large, prognathous, dark-bodied beetles. Most with crenelations at posterior edge of pronotum.

**Genus *Melanactes* LeConte, 1853**

**Habitus.** Body length 20–40 mm. ***Head*.** Supra-antennal carinae reaching anterior edge of head capsule. Antennae with 11 antennomeres, sensory elements beginning on antennomere IV. ***Prothorax*.** Pronotum hind angle carinae present (single), crenellations present or absent, hypomeral bead present. Prosternal process not curved upward ≥ 40° in lateral view. ***Mesothorax*.** Mesocoxal cavities open, scutellum with posterior edge rounded (arcuate). Elytra. Striae absent, integument unmarked; setal vestiture sparse on disk. ***Legs*.** Metacoxal plate reaching side. ***Aedeagus*.** Each paramere with three or more setae.

**Summary.** Large beetles with elytra not striate and mostly bare of setae. Also, hind edge of pronotum crenelate in most, claws without setae.

**Genus *Oistus* Candèze, 1857**

**Habitus.** Body length 17–35 mm. ***Head*.** Supra-antennal carinae not complete across frons. Antennae with 11 antennomeres, sensory elements beginning on antennomere IV. ***Prothorax*.** Pronotum hind angle carinae absent, crenellations present, hypomera with posterior concavity present. Prosternal process not curved upward ≥ 40° in lateral view. ***Mesothorax*.** Mesocoxal cavities not closed. Elytra. Striae present; integument marked with spots or bands in some; setal vestiture even and mainly parallel or with bare patches. ***Legs*.** Metacoxal plate reaching side. ***Aedeagus*.** Parameres with apical lateral expansions present, without setae.

**Summary.** Posterior edge of pronotum crenellate (weak in some), antennal sensory elements beginning on antennomere IV (widespread rough texture), elytra setose (setae longer than width of antennomere II).

**Genus *Perissarthron* Hyslop, 1917**

**Habitus.** Body length 17–35 mm. ***Head*.** Supra-antennal carinae fading on frons or reaching anterior edge of head capsule. Antennae with 12 antennomeres in most, sensory elements beginning on antennomere III. ***Prothorax*.** Pronotum hind angle carinae present (single), crenellations present, hypomeral bead present; hypomera with posterior concavities arcuate. ***Mesothorax*.** Mesocoxal cavities open. Elytra. Striae present; integument unmarked; setal vestiture even and mainly parallel. ***Aedeagus*.** Each paramere with two setae.

**Summary.** Large beetles with posterior edge of pronotum crenellate, antennal sensory elements beginning on antennomere III (widespread rough texture).

**Tribe Prosternini**

**Habitus.** Body length 3–30 mm. ***Head*.** Supra-antennal carinae, fading on frons (not reaching another structure) in most; most prognathous. Antennae with 11 antennomeres, not pectinate in most. ***Prothorax*.** Pronotum without tubercles between punctures; pronotal lateral carinae complete in most, visible throughout length in most or all, not serrate; hind angle carinae single or absent, posterior edge with crenellations absent in most; pronotosternal sutures closed in most; hypomeron with posterior edge near hind angle with concavity in most; prosternal process not curved upward ≥ 40° in lateral view in most. ***Mesothorax*.** Mesocoxal cavities fully open in most, not closed; scutellum with anterior edge not concave in most or all. Elytra. Anterior edge straight to arcuate near humeri in dorsal view in most, striate in most, setal vestiture even and mainly parallel in most; most without pattern from differences in setal color. ***Legs*.** Metacoxal plate reaching side in most; tarsal pads absent, claws simple and long in most. **Ventrites**. Sides not microserrate in most, ventrite V apex arcuate without paired setal brushes in most.

**Summary.** It is difficult to define this group collectively. They include most Dendrometrinae with supra-antennal carinae not both continuous and raised across frons. Tarsomeres simple. Hind edge of pronotum defined by a right-angled ledge near hind angles in dorsal view (edge rounded in some Dendrometrini).

**Genus *Acteniceromorphus* Kishii, 1955**

**Habitus.** Body length 12–18 mm. ***Head*.** Antennal, sensory elements beginning on antennomere III. ***Prothorax*.** Pronotum with punctures simple; hind angle carinae present; posterior edge of pronotum with sublateral notches present; pronotosternal sutures closed, hypomeral bead absent. Prosternum with sides straight at midlength. ***Mesothorax*.** Scutellum with posterior edge rounded (arcuate) or pointed (acuminate). Elytra. Integument unmarked. ***Aedeagus*.** Each paramere with two or more setae.

**Summary.** Supra-antennal carinae fading on frons, sensory elements beginning on antennomere III (widespread rough sculpturing and appressed setae); pronotum with setae on anterior half directed anterad, without setae directed mesad near midlength, hind angles with carinae, sublateral notches present; parameres each with two or more setae. Not distinguished here from all *Paractenicera* and *Proludius* species.

***A.sagitticollis*(Eschscholtz)**

**Habitus.** Body length 10–15 mm. ***Head*.** Antennae with sensory elements beginning on antennomere IV (widespread rough sculpturing and appressed setae). ***Prothorax*.** Pronotum with punctures simple, lateral carinae complete; hind angle carinae present; posterior edge of pronotum with sublateral notches present; pronotosternal sutures closed, hypomeral bead absent. Prosternum with sides straight at midlength. ***Mesothorax*.** Scutellum with posterior edge rounded (arcuate). Elytra. Integument unmarked. ***Aedeagus*.** Parameres without apical lateral expansions; each with three or more setae.

**Summary.** Supra-antennal carinae fading on frons, sensory elements beginning on antennomere IV, pronotal sublateral notches present, hypomeral bead absent, ventrites without microserration, parameres without preapical expansions, each with three or more setae.

**Genus *Actenicerus* Kiesenwetter, 1858**

**Habitus.** Body length 12–18 mm. ***Head*.** Antennae with sensory elements beginning on antennomere III. ***Prothorax*.** Pronotal lateral carinae complete, posterior crenellations absent; pronotosternal sutures closed, hypomeral bead present. Prosternum with sides straight at midlength. ***Mesothorax*.** Scutellum with posterior edge rounded (arcuate). Elytra. Integument unmarked, but with pattern from differences in setal color. ***Aedeagus*.** Parameres with apical lateral expansions present, without setae.

**Summary.** Dark bodied with elytral pattern from patches of pale setae among darker setae, supra-antennal carinae fading on frons, pronotum with most setae near midline on anterior half directed mesad.

**Genus *Anostirus* C.G. Thomson, 1859**

**Habitus.** Body length 6–12 mm. ***Head*.** Antennae serrate, sensory elements beginning on antennomere III or IV. ***Prothorax*.** Pronotum with punctures simple; hind angle carinae absent; posterior edge of pronotum with sublateral notches present; hypomera with concavity on posterior edge near hind angle; pronotosternal sutures closed. Prosternum with sides straight at midlength. ***Mesothorax*.** Scutellum rounded posterad. Elytra. Integument marked with spots or transverse bands on a pale background; some with pattern from differences in setal color. **Ventrites**. Microserration at sides (e.g., 100 points per mm) present. ***Aedeagus*.** Each paramere with two or more setae.

**Summary.** Supra-antennal carinae fading on frons, pronotal hind angles carinae absent (or faint); elytra pale with paired dark markings, abdominal ventrites microserrate.

**Genus *Anthracopteryx* Horn, 1891**

**Habitus.** Body length 5–10 mm. ***Head*.** Antennae with sensory elements beginning on antennomere IV. ***Prothorax*.** Pronotum with punctures simple; hind angle carinae present; posterior edge of pronotum without sublateral notches; pronotosternal sutures closed, hypomeral bead absent; hypomera with angulate concavity on posterior edge near hind angle. Prosternum with sides concave at midlength. ***Mesothorax*.** Scutellum with anterior edge concave or not, posterior edge rounded (arcuate). Elytra. Integument unmarked. ***Legs*.** Metacoxal plate reaching side or not. ***Aedeagus*.** Parameres with apical lateral expansions present; each paramere with three or more setae.

**Summary.** All black, supra-antennal carinae fading on frons, pronotum on anterior half with setae oriented posterad or mesad, and with some setae near midlength oriented laterad, hind angles with carinae, sublateral notches absent, pronotosternal sutures closed, prosternum with sides concave, prosternal process not ascendent. Also, hind wings short.

**Genus *Athoplastus* Johnson & Etzler, 2018**

**Habitus.** Body length 12–18 mm. ***Head*.** Antennae with sensory elements beginning on antennomere IV. ***Prothorax*.** Pronotum with punctures simple; hind angle carinae present; posterior edge of pronotum with sublateral notches present; pronotosternal sutures closed. Prosternum with sides concave at midlength; prosternal process curved upward > 40° in lateral view. ***Mesothorax*.** Scutellum rounded posterad. Elytra. Integument unmarked. ***Aedeagus*.** Parameres with apical lateral expansions present; each paramere with three or more setae.

**Summary.** Supra-antennal carinae fading on frons, sensory elements beginning on antennomere IV (erect setae), pronotum with sublateral notches present, pronotum and elytra not contrasting, prosternum with sides concave, prosternal process ascendent, parameres with three or more setae. Not completely separated from *Metanomus*.

**Genus *Beckerus* Johnson, 2008**

**Habitus.** Body length 6–10 mm. ***Head*.** Antennae serrate to pectinate, sensory elements beginning on antennomere III. ***Prothorax*.** Pronotum with punctures simple; hind angle carinae present; posterior edge of pronotum without sublateral notches, but with crenellations present; pronotosternal sutures closed, hypomeral bead absent. Prosternum with sides concave at midlength; prosternal process curved upward > 40° in lateral view. ***Mesothorax*.** Scutellum rounded posterad or pointed (acuminate). Elytra. Integument marked with spots or transverse bands; setal vestiture sparse on disk or even and mainly parallel. ***Aedeagus*.** Parameres with apical lateral expansions present; each paramere with three or more setae.

**Summary.** Pronotum and elytra each with contrasting color patterns, posterior edge of pronotum crenellate, antennal sensory elements beginning on antennomere III (erect setae, widespread rough sculpturing).

**Genus *Billbrownia* Johnson, 2023**

**Habitus.** Body length 6–12 mm. ***Head*.** Supra-antennal carinae fading on frons or reaching anterior edge of head capsule. Antennae with sensory elements beginning on antennomere IV. ***Prothorax*.** Pronotum with punctures simple; hind angle carinae present; posterior edge of pronotum with sublateral notches present; pronotosternal sutures closed, hypomeral bead weak or absent. Prosternum with sides straight at midlength. ***Mesothorax*.** Scutellum rounded posterad. Elytra. Integument unmarked; setal vestiture sparse on disk or even and mainly parallel. ***Aedeagus*.** Parameres without apical lateral expansions, without setae.

**Summary.** Sensory elements (apical sensorium) beginning on antennomere IV, pronotum with punctures simple, setae on anterior half directed anterad or laterad, sublateral notches present, elytra bare or with vestiture even and mainly parallel, parameres without lateral expansions or setae. We could not write a diagnosis that distinguished this from some *Setasomus*.

***B* . *signaticollis* (Melsheimer, 1845), and similar species**

**Habitus.** Body length 6–12 mm. ***Head*.** Supra-antennal carinae fading on frons or reaching anterior edge of head capsule. Antennae with sensory elements beginning on antennomere III (apical sensorium). ***Prothorax*.** Pronotum with punctures simple; hind angle carinae present; posterior edge of pronotum with sublateral notches present; pronotosternal sutures closed, hypomeral bead absent. Prosternum with sides straight or convex at midlength. ***Mesothorax*.** Scutellum rounded posterad. Elytra. Integument unmarked with spots or transverse bands; setal vestiture sparse on disk or even and mainly parallel. ***Aedeagus*.** Parameres with apical lateral expansions in some, without setae.

**Summary.** Differ from type species by having sensory elements begin on antennomere III. Remaining characters: pronotum with punctures simple, setae on anterior half directed anterad or laterad, hind angles carinate, parameres without lateral expansions or setae.

**Genus *Corymbitodes* Buysson, 1904**

**Habitus.** Body length 6–12 mm. ***Head*.** Supra-antennal carinae fading on frons or reaching anterior edge of head capsule. Antennae with sensory elements beginning on antennomere III. ***Prothorax*.** Pronotum with some punctures umbilicate; hind angle carinae absent; posterior edge of pronotum with sublateral notches in some; pronotosternal sutures closed, hypomeral bead present. Prosternum with sides straight at midlength. ***Mesothorax*.** Scutellum rounded posterad or pointed (acuminate). Elytra. Integument unmarked except for humeral spots or dark sutural striae in some. ***Legs*.** Metacoxal plate reaching side or not. ***Aedeagus*.** Parameres without apical lateral expansions, without setae, each paramere with one or two setae.

**Summary.** Supra-antennal carina not complete across frons, sensory elements beginning on antennomere III (apical sensorium, widespread appressed setae), pronotum with umbilicate punctures at sides, some setae directed laterad near midlength (near sides), pronotal hind angles non-carinate, pronotosternal sutures with hypomeral bead (narrow).

**Genus *Ctenicera* Latreille, 1829**

**Habitus.** Body length 12–18 mm. ***Head*.** Antennae serrate to pectinate, sensory elements beginning on antennomere III. ***Prothorax*.** Pronotum with punctures simple; hind angle carinae present; posterior edge of pronotum with sublateral notches present; pronotosternal sutures closed, hypomeral bead present. Prosternum with sides straight at midlength. ***Mesothorax*.** Scutellum rounded posterad. Elytra. Integument marked with diagonal band in apical half only. ***Aedeagus*.** Parameres without apical lateral expansions; each paramere with three or more setae.

**Summary.** Supra-antennal carinae fading on frons, antennae serrate or pectinate, prosternum with sides straight, elytra with dark markings only in apical third.

***C.angularis* (LeConte)**

**Habitus.** Body length 6–12 mm. ***Head*.** Antennae with sensory elements beginning on antennomere IV. ***Prothorax*.** Pronotum with punctures simple; hind angle carinae present; hind angles pale; posterior edge of pronotum with sublateral notches present; pronotosternal sutures closed, hypomeral bead present; hypomeron with posterior edge near hind angle with slight concavity, concavity arcuate. Prosternum with sides straight at midlength. ***Mesothorax*.** Scutellum rounded posterad. Elytra. Integument unmarked. ***Aedeagus*.** Parameres with apical lateral expansions present; each paramere with one or two setae.

**Summary.** Dark bodied with pale pronotal hind angles. Supra-antennal carinae fading on frons, prognathous, pronotum with setae on anterior half near midline directed posterad, pronotum with sublateral notches present (small), parameres with one or two setae. The key distinguished all specimens from *Liotrichus*, although this diagnosis does not. This species is associated with *Ctenicera* for historical reasons only.

***C.uliginosa*(Van Dyke)**

**Habitus.** Body length 6–12 mm. ***Head*.** Antennae with sensory elements beginning on antennomere IV. ***Prothorax*.** Pronotum with punctures simple; hind angle carinae present; posterior edge of pronotum with sublateral notches present; pronotosternal sutures closed, hypomeral bead absent. Prosternum with sides straight at midlength. ***Mesothorax*.** Scutellum with anterior edge concave or not, posterior edge rounded (arcuate). Elytra. Integument dark. ***Aedeagus*.** Parameres with apical lateral expansions present; each paramere with two or more setae.

**Summary.** Supra-antennal carinae fading on frons, sensory elements beginning on antennomere IV, antennomere IV not longer than each of V to VII, pronotum with setae near midline on anterior half directed mesad, sublateral notches present, hypomeral bead absent, elytra dark throughout, parameres lateral expansions present and with two or more setae. This species is associated with *Ctenicera* for historical reasons only.

**Genus *Dixicollis* Johnson, 2021**

**Habitus.** Body length 6–12 mm. ***Head*.** Antennae with sensory elements beginning on antennomere III. ***Prothorax*.** Pronotum with punctures simple; hind angle carinae present; posterior edge of pronotum without sublateral notches; pronotosternal sutures open, hypomeral bead present; hypomera with arcuate or angulate concavity on posterior edge near hind angle. Prosternum with sides straight at midlength; prosternal process curved upward > 40° in lateral view. ***Mesothorax*.** Scutellum rounded posterad. Elytra. Integument unmarked. ***Aedeagus*.** Parameres with apical lateral expansions present, without setae.

**Summary.** Supra-antennal carinae fading on frons, sensory elements beginning on antennomere III (not dense), pronotum without sublateral notches, pronotosternal sutures narrowly open.

**Genus *Eanus* LeConte, 1861**

**Habitus.** Body length 3–10 mm. ***Head*.** Antennae with sensory elements beginning on antennomere III or IV. ***Prothorax*.** Pronotum with punctures simple; pronotal lateral carinae complete or not; hind angle carinae absent; posterior edge of pronotum without sublateral notches; pronotosternal sutures closed, hypomeral bead present. Prosternum with sides concave at midlength; prosternal process curved upward > 40° in lateral view. ***Mesothorax*.** Scutellum with posterior edge pointed (acuminate). Elytra. Integument in some marked with spots or transverse bands. ***Aedeagus*.** Each paramere with one or two setae.

**Summary.** Supra-antennal carinae fading on frons, antennomere III closer in length to antennomere IV than to II, pronotum with setae near midline on anterior half directed anterad, with some setae directed laterad near midlength, prosternal sides concave, prosternal process ascendant, parameres with one or two setae each.

***E.striatipennis*Brown**

**Habitus.** Body length 5–10 mm. ***Head*.** Antennae with sensory elements beginning on antennomere IV. ***Prothorax*.** Pronotum with punctures simple; hind angle carinae absent; posterior edge of pronotum without sublateral notches; pronotosternal sutures closed, hypomeral bead absent. Prosternum with sides straight or concave at midlength; prosternal process curved upward > 40° in lateral view. ***Mesothorax*.** Scutellum rounded posterad or pointed (acuminate). Elytra. Striae present or absent, integument with metallic reflections. ***Aedeagus*.** Parameres with apical lateral expansions present; each paramere with three or more setae.

**Summary.** Supra-antennal carinae fading on frons, antennomere III closer in length to antennomere II than to IV, prosternal sides concave, prosternal process ascendant, elytra with metallic reflections. Treated as distinctive to improve overall effectiveness of the key.

**Genus *Hadromorphus* Motschulsky, 1859**

**Habitus.** Body length 6–12 mm, setal vestiture dense but not hiding integument. ***Head*.** Antennae with sensory elements beginning on antennomere IV. ***Prothorax*.** Pronotum with some punctures umbilicate; hind angle carinae absent in most; posterior edge of pronotum with sublateral notches in some; pronotosternal sutures open, hypomeral bead present in most. Prosternum with sides straight at midlength. ***Mesothorax*.** Mesocoxal cavities not closed. Scutellum rounded posterad. Elytra. Integument unmarked. ***Legs*.** Metacoxal plate reaching side or not. **Ventrites**. Microserration at sides (e.g., 100 points per mm) present or absent. ***Aedeagus*.** Parameres with apical lateral expansions present; each paramere with three or more setae.

**Summary.** Supra-antennal carinae fading on frons, sensory elements beginning on antennomere IV, pronotum with setae directed both mesad and laterad near midlength (converging or swirled), pronotosternal sutures open, and straight at midlength.

**Genus *Hypoganus* Kiesenwetter, 1858**

**Habitus.** Body length 6–12 mm. ***Head*.** Antennae with sensory elements beginning on antennomere IV. ***Prothorax*.** Pronotum with punctures simple; hind angle carinae present; posterior edge of pronotum without sublateral notches; pronotosternal sutures closed, hypomeral bead present. Prosternum with sides straight or convex at midlength. ***Mesothorax*.** Scutellum rounded posterad. Elytra. Integument unmarked. ***Aedeagus*.** Parameres with apical lateral expansions present, without setae.

**Summary.** Brown or black bodied, some with red on pronotum, hypognathous, supra-antennal carinae fading on frons, sensory elements beginning on antennomere IV, pronotum wider than long with setae fine and mainly directed laterad, sublateral notches absent, parameres with sublateral expansions present and setae absent. Not separated here from *Selatosomusnigricans*.

**Genus *Laneganus* Johnson, 2001**

**Habitus.** Body length 6–18 mm. ***Head*.** Supra-antennal carinae fading on frons, or complete but without concavity below in lateral view. Antennae with sensory elements beginning on antennomere IV. ***Prothorax*.** Pronotum with punctures simple; hind angle carinae present; posterior edge of pronotum without sublateral notches; pronotosternal sutures closed, hypomeral bead present. Prosternum with sides straight or convex at midlength. ***Mesothorax*.** Mesocoxal cavities not closed. Scutellum rounded posterad. Elytra. Integument unmarked or marked with spot or band in apical half only. ***Legs*.** Metacoxal plates reaching side or not. ***Aedeagus*.** Parameres with apical lateral expansions present, without setae or each paramere with three or more setae.

**Summary.** Supra-antennal carinae fading on frons or complete across frons but not concave below, sensory elements beginning on antennomere IV, pronotum longer than wide with setae fine and mainly directed posterad or laterad on anterior half, sublateral notches absent, long pronotal hind angles reaching to midlength of scutellum when body straightened, pronotosternal sutures closed and straight, hypomeral bead present, tarsal claws without basal setae.

**Genus *Liotrichus* Kiesenwetter, 1858**

**Habitus.** Body length 6–12 mm. ***Head*.** Antennae with sensory elements beginning on antennomere IV. ***Prothorax*.** Pronotum with punctures simple; hind angle carinae present or absent; posterior edge of pronotum with sublateral notches in some; pronotosternal sutures closed, hypomeral bead present; hypomera with posterior concavities arcuate or angulate. Prosternum with sides straight or concave at midlength. ***Mesothorax*.** Scutellum rounded posterad. Elytra. Integument marked with bands in some. ***Aedeagus*.** Parameres with apical lateral expansions present.

**Summary.** Pronotum darker than elytra, supra-antennal carinae fading on frons, prognathous, antennae not pectinate or serrate, antennomere III closer in length to IV than to II, pronotum with setae on anterior half near midline directed posterad, some directed laterad near midlength, and directed anterad on posterior part near midline, pronotosternal sutures closed, hypomeral bead present, mesocoxal cavities open, claws simple. The key distinguished all specimens from *Setasomus*, although this diagnosis does not.

**Genus *Metanomus* Buysson, 1887**

**Habitus.** Body length 6–12 mm. ***Head*.** Antennae with sensory elements beginning on antennomere IV. ***Prothorax*.** Pronotum with punctures simple; hind angle carinae present; posterior edge of pronotum with sublateral notches present; pronotosternal sutures closed. Prosternum with sides straight to concave at midlength. ***Mesothorax*.** Scutellum rounded posterad. Elytra. Integument unmarked. ***Aedeagus*.** Parameres with apical lateral expansions present; each paramere with three or more setae.

**Summary.** Supra-antennal carinae fading on frons, sensory elements beginning on antennomere IV, antennomere III closer in length to II than to IV, pronotum on anterior part with most setae directed posterad, some directed laterad near midlength, and anterad on posterior slope, prosternum with sides straight or concave, claws simple, parameres with three or more setae. Not completely separated from *Athoplastus* here.

**Genus *Neopristilophus* Buysson, 1894**

**Habitus.** Body length 12–25 mm. ***Head*.** Supra-antennal carinae fading on frons or reaching anterior edge of head capsule. Antennae with sensory elements beginning on antennomere III or IV. ***Prothorax*.** Pronotum with some punctures umbilicate; hind angle carinae present; posterior edge of pronotum with sublateral notches present in most; pronotosternal sutures closed, hypomeral bead absent. Prosternum with sides straight at midlength. ***Mesothorax*.** Scutellum rounded posterad. Elytra. Integument unmarked. ***Aedeagus*.** Parameres without apical lateral expansions, without setae.

**Summary.** Large, dark-bodied beetles. Supra-antennal carinae fading on frons or directed anterad, pronotum with some punctures umbilicate, pronotum with hind angles carinate, pronotosternal sutures closed, parameres with both preapical expansions and setae absent.

**Genus *Nitidolimonius* Johnson, 2008**

**Habitus.** Body length 6–12 mm. ***Head*.** Supra-antennal carinae variable. Antennae with sensory elements beginning on antennomere IV. ***Prothorax*.** Pronotum with punctures umbilicate in some; hind angle carinae present; posterior edge of pronotum with sublateral notches present; pronotosternal sutures open, hypomeral bead present. Prosternum with sides straight at midlength. ***Mesothorax*.** Scutellum rounded posterad. Elytra. Integument with metallic reflections in most; setal vestiture sparse on disk or even and mainly parallel. ***Aedeagus*.** Parameres with apical lateral expansions present, without setae or each paramere with three or more setae.

**Summary.** Pronotum with metallic reflections, pronotosternal sutures open, prosternum with sides straight, ventrites without microserration at sides.

**Genus *Oxygonus* LeConte, 1863**

**Habitus.** Body length 3–12 mm. ***Head*.** Supra-antennal carinae fading on frons or reaching anterior edge of head capsule. Antennae with sensory elements beginning on antennomere IV. ***Prothorax*.** Pronotum with punctures umbilicate in some; hind angle carinae present or absent; posterior edge of pronotum with sublateral notches in some; pronotosternal sutures closed, hypomeral bead present. Prosternum with sides straight at midlength. ***Mesothorax*.** Scutellum rounded posterad. Elytra. Integument unmarked; setal vestiture even and mainly parallel or partially transverse in patches. ***Legs*.** Tarsal claws appendiculate. ***Aedeagus*.** Parameres with apical lateral expansions present, without setae or each paramere with three or more setae.

**Summary.** Pronotosternal sutures straight, tarsi simple, tarsal claws appendiculate.

**Genus *Paractenicera* Johnson, 2008**

**Habitus.** Body length 12–18 mm. ***Head*.** Antennae with sensory elements beginning on antennomere III. ***Prothorax*.** Pronotum with some punctures umbilicate; hind angle carinae present; posterior edge of pronotum with sublateral notches present; pronotosternal sutures closed, hypomeral bead absent. Prosternum with sides straight at midlength. ***Mesothorax*.** Scutellum with posterior edge pointed (acuminate). Elytra. Integument unmarked. ***Aedeagus*.** Parameres without apical lateral expansions; each paramere with two setae.

**Summary.** Supra-antennal carinae fading on frons, pronotum with setae on anterior half directed anterad, some setae directed laterad near midlength, hind angles with carinae, sublateral notches present, scutellum obtusely pointed posterad, parameres with two setae. *Paractenicerafulvipes* is not distinguished from *Acteniceromorphus* here.

**Genus *Proludius* Lane, 1971**

**Habitus.** Body length 12–18 mm. ***Head*.** Supra-antennal not complete across frons or complete, but without concavity below in lateral view. Antennae with sensory elements beginning on antennomere III. ***Prothorax*.** Pronotum with punctures umbilicate in some; hind angle carinae present; posterior edge of pronotum with sublateral notches in some; pronotosternal sutures closed; hypomera with arcuate or angulate concavity on posterior edge near hind angle. Prosternum with sides straight at midlength. ***Mesothorax*.** Scutellum with anterior edge concave or not, posterior edge rounded (arcuate). Elytra. Integument unmarked. ***Aedeagus*.** Parameres with apical lateral expansions present; each paramere with two or more setae.

**Summary.** Supra-antennal carina not complete across frons or complete, but without concavity below in lateral view, sensory elements beginning on antennomere III, pronotum not contrasting with elytra in color, with setae on anterior half directed anterad or mesad, some setae directed laterad or mesad near midlength, hind angles with carinae (faint in some), prosternal process not ascendant, scutellum rounded posterad, mesocoxal cavities open, elytral setal color pattern absent, tarsal pads absent. *Proludius* is not distinguished from some *Acteniceromorphus* or *Sylvanelater* here.

***P.angusticollis*(Mannerheim), and similar species**

**Habitus.** Body length 12–18 mm. ***Head*.** Antennae with sensory elements beginning on antennomere IV. ***Prothorax*.** Pronotum with punctures umbilicate in some; hind angle carinae present; posterior edge of pronotum with sublateral notches present; pronotosternal sutures closed. Prosternum with sides straight at midlength. ***Mesothorax*.** Scutellum rounded posterad. Elytra. Integument unmarked. ***Aedeagus*.** Parameres with apical lateral expansions present, without setae.

**Summary.** Antennomere III closer in length to II than IV, anterior half of pronotum with most setae directed mesad, posterior slope of pronotum without setae directed anterad, parameres without setae. Captures species with sensory elements beginning on antennomere IV and parameres without setae.

***P.sylvaticus*(Van Dyke)**

**Habitus.** Body length 12–18 mm. ***Head*.** Antennae with sensory elements beginning on antennomere III. ***Prothorax*.** Pronotum with some punctures umbilicate; hind angle carinae present; posterior edge of pronotum with sublateral notches present; pronotosternal sutures closed, hypomeral bead absent. Prosternum with sides straight at midlength. ***Mesothorax*.** Scutellum rounded posterad. Elytra. Integument unmarked. ***Aedeagus*.** Parameres with apical lateral expansions present, without setae.

**Summary.** Sensory elements beginning on antennomere III, antennomere IV longer than each of V to VII, pronotum with most setae near midline on anterior half directed anterad, some directed laterad near midlength, parameres with rounded preapical expansions and without setae. Captures species with sensory elements beginning on antennomere III and parameres without setae.

**Genus *Prosternon* Latreille, 1834**

**Habitus.** Body length 6–12 mm. ***Head*.** Antennae with sensory elements beginning on antennomere IV. ***Prothorax*.** Pronotum with punctures umbilicate in some; hind angle carinae present; posterior edge of pronotum with sublateral notches present in most; pronotosternal sutures closed, hypomeral bead present; hypomeron with posterior edge near hind angle without concavity. Prosternum with sides straight at midlength. ***Mesothorax*.** Scutellum rounded posterad. Elytra. Integument unmarked; setal vestiture partially transverse in patches. **Ventrites**. Microserration at sides (e.g., 100 points per mm) present. ***Aedeagus*.** Parameres with apical lateral expansions present, each paramere without or with two or more setae.

**Summary.** Prognathous, elytra with setae swirled and pale, claws simple.

**Genus *Pseudanostirus* Dolin, 1964**

***P.ochreipennis*(LeConte), and similar species**

**Habitus.** Body length 6–12 mm. ***Head*.** Supra-antennal carinae fading on frons. Antennae with sensory elements beginning on antennomere IV. ***Prothorax*.** Pronotum with punctures simple; hind angle carinae present or absent; posterior edge of pronotum with sublateral notches present; pronotosternal sutures closed. Prosternum with sides straight at midlength. ***Mesothorax*.** Scutellum rounded posterad. Elytra. Integument unmarked with spots or transverse bands, vestiture not swirled. **Ventrites**. Microserration at sides (e.g., 100 points per mm) present. ***Aedeagus*.** Parameres without apical lateral expansions; each paramere with two or more setae.

**Summary.** Supra-antennal carinae fading on frons, pronotosternal sutures closed, elytra without transverse bands or apical markings, vestiture not swirled, ventrites microserrate. Species without swirled elytral vestiture, with ventrites microserrate, and without paramere expansions treated as distinctive sub-group here to improve diagnostic effectiveness.

***P.laricis*(Brown)**

**Habitus.** Body length 5–10 mm. ***Head*.** Antennae with sensory elements beginning on antennomere IV. ***Prothorax*.** Pronotum with punctures simple; hind angle carinae present or absent; posterior edge of pronotum with sublateral notches present; pronotosternal sutures closed, hypomeral bead absent. Prosternum with sides straight at midlength. ***Mesothorax*.** Scutellum rounded posterad. Elytra. Integument unmarked, vestiture not swirled. ***Aedeagus*.** Parameres with apical lateral expansions present; each paramere with three or more setae.

**Summary.** Pronotum not darker than elytra, supra-antennal carinae fading on frons, sensory elements beginning on antennomere IV, antennomere III closer in length to IV than II, pronotal punctures simple, setae on anterior half of pronotum directed posterad, sublateral notches present, hypomeral bead absent, parameres with three or more setae. Treated as distinctive subgroup to improve diagnostic effectiveness.

***P.triundulatus*(Randall), and similar species**

**Habitus.** Body length 6–12 mm. ***Head*.** Antennal sensory elements beginning on antennomere IV. ***Prothorax*.** Pronotum with punctures simple; hind angle carinae absent; posterior edge of pronotum without sublateral notches; pronotosternal sutures closed, hypomeral bead present; hypomeron with posterior edge near hind angle without concavity. Prosternum with sides straight at midlength. ***Mesothorax*.** Scutellum rounded posterad. Elytra. Integument unmarked with spots or transverse bands; but with pattern from differences in setal color, vestiture not swirled. ***Aedeagus*.** Parameres without apical lateral expansions, without setae or each paramere with one seta.

**Summary.** Pronotum darker than elytra, elytra with transverse bands of dark and pale setae, supra-antennal carinae fading on frons, sublateral notches absent, pronotosternal sutures closed. Species with pronotal sublateral notches, concavity on posterior edge of hypomeron, and bands of dark setae on elytra treated included as distinctive sub-group here to improve diagnostic effectiveness.

**Genus *Selatosomus* Stephens, 1830**

**Habitus.** Body length 7–18 mm. ***Head*.** Antennae with sensory elements beginning on antennomere IV. ***Prothorax*.** Pronotum with punctures umbilicate in some; hind angle carinae present; posterior edge of pronotum with sublateral notches present; pronotosternal sutures closed. Prosternum with sides straight or concave at midlength. ***Mesothorax*.** Scutellum rounded posterad. Elytra. Integument marked with spots or transverse bands, with metallic reflections in some; setal vestiture sparse on disk. ***Legs*.** Metacoxal plate reaching side or not. ***Aedeagus*.** Parameres with apical lateral expansions present, each paramere without or with two or more setae.

**Summary.** Sensory elements (apical sensorium) beginning on antennomere IV, pronotum with sublateral notches, pronotosternal sutures closed, elytral disk mostly without setal vestiture (if present then appressed and only 1/4 as long as interstrial width), ventrite V with apex arcuate, parameres with lateral expansions. We could not write a diagnosis that distinguished some species of *Billbrownia* from this, although the key accurately diagnosed all specimens.

***S.nigricans*(Fall)**

**Habitus.** Body length 6–12 mm. ***Head*.** Antennae with sensory elements beginning on antennomere IV. ***Prothorax*.** Pronotum with punctures simple; hind angle carinae present; posterior edge of pronotum without sublateral notches; pronotosternal sutures closed, hypomeral bead present. Prosternum with sides straight or convex at midlength. ***Mesothorax*.** Scutellum rounded posterad. Elytra. Integument unmarked; setal vestiture sparse on disk or even and mainly parallel. ***Aedeagus*.** Parameres with apical lateral expansions present, without setae.

**Summary.** Brown or black bodied, some with red on pronotum, sensory elements beginning on antennomere IV, pronotum wider than long with setae fine and mainly directed laterad, sublateral notches absent, hypomeral bead impunctate. We could not write a diagnosis that distinguished this from *Hypoganus*. Treated as distinctive subgroup to improve diagnostic effectiveness because of lack of pronotal sublateral notches.

***S.pruininus*(Horn)**

**Habitus.** Body length 6–12 mm. ***Head*.** Antennae with sensory elements beginning on antennomere IV. ***Prothorax*.** Pronotum with punctures umbilicate in some; hind angle carinae present; posterior edge of pronotum with sublateral notches present; pronotosternal sutures closed, hypomeral bead present. Prosternum with sides concave at midlength. ***Mesothorax*.** Scutellum rounded posterad. Elytra. Integument unmarked. ***Aedeagus*.** Parameres with apical lateral expansions present; each paramere with three or more setae.

**Summary.** Supra-antennal carinae fading on frons, sensory elements beginning on antennomere IV (apical sensorium), pronotum setae on anterior half directed posterolaterad, and posterior slope without setae directed anterad, with sublateral notches present, pronotosternal sutures closed, hypomeral bead present at midlength (partly punctate), elytra with setae evenly distributed and mainly parallel, ventrite V not sinuate apicad and without setal brushes. Treated as distinctive subgroup to improve diagnostic effectiveness because of setose elytra. Not morphologically distinguished here from *Setasomus*.

**Genus *Setasomus* Gurjeva, 1985**

**Habitus.** Body length 6–12 mm. ***Head*.** Supra-antennal carinae joining medially (forming shelf) or fading on frons; nasale (head capsule below edge of frontal carina) with outline not concave in lateral view. Antennae with sensory elements beginning on antennomere IV. ***Prothorax*.** Pronotum with punctures simple; hind angle carinae present; posterior edge of pronotum with sublateral notches present; pronotosternal sutures closed, hypomeral bead present. Prosternum with sides straight at midlength. ***Mesothorax*.** Mesocoxal cavities not closed. Scutellum rounded posterad. Elytra. Integument marked with spots or bands in some. ***Aedeagus*.** Parameres with apical lateral expansions present or absent, without setae or each paramere with three or more setae.

**Summary.** Supra-antennal carinae fading on frons, or joining medially (not concave below in lateral view), sensory elements beginning on antennomere IV, antennomere III closer in length to IV than to II, pronotum with sublateral notches present, pronotosternal sutures closed, hypomeral bead present at midlength (punctate in some), prosternal sides straight, elytra with setae evenly distributed and mainly parallel, tarsi and claws simple, ventrites not microserrate at sides and without setal brushes apically, parameres with zero or more than three setae. We could not write a diagnosis that distinguished this from some *Liotrichus or Sylvanelater*.

**Genus *Stropenron* Johnson, 2021**

**Habitus.** Body length 6–12 mm. ***Head*.** Supra-antennal carinae fading on frons. Antennae with sensory elements beginning on antennomere IV. ***Prothorax*.** Pronotum with punctures simple; hind angle carinae present; posterior edge of pronotum with sublateral notches present; pronotosternal sutures closed. Prosternum with sides straight at midlength. ***Mesothorax*.** Scutellum rounded posterad. Elytra. Integument marked with spots or transverse bands or marked in apical half only. **Ventrites**. Microserration at sides (e.g., 100 points per mm) present. ***Aedeagus*.** Parameres setose.

**Summary.** Supra-antennal carinae fading on frons, pronotum with hind angle carina present in most, elytra pale with angulate paired dark markings at least on apical half, abdominal ventrites microserrate. Incompletely separated from *Anostirus* in key.

**Genus *Sylvanelater* Johnson, 2008**

**Habitus.** Body length 6–18 mm. ***Head*.** Antennae with sensory elements beginning on antennomere III. ***Prothorax*.** Pronotum with punctures simple; hind angle carinae present; posterior edge of pronotum with sublateral notches present; pronotosternal sutures closed; hypomera with arcuate or angulate concavity on posterior edge near hind angle. Prosternum with sides straight at midlength; prosternal process curved upward > 40° in lateral view in some. ***Mesothorax*.** Scutellum rounded posterad. Elytra. Integument unmarked. ***Aedeagus*.** Parameres with apical lateral expansions present, without setae or each paramere with three or more setae.

**Summary.** Supra-antennal carinae fading on frons, sensory elements beginning on antennomere III, pronotum with setae on anterior half directed anterad or mesad, some setae directed mesad near midlength, sublateral notches present, pronotosternal sutures closed, elytral setal and integument color patterns absent. Not distinguished from some *Proludius* here.

***S.atropurpureus* (Melsheimer)**

**Habitus.** Body length 6–12 mm. ***Head*.** Supra-antennal carinae joining medially (forming shelf) or reaching anterior edge of head capsule; nasale (head capsule below edge of frontal carina) with outline not concave in lateral view. Antennae with sensory elements beginning on antennomere III. ***Prothorax*.** Pronotum with punctures simple; hind angle carinae present or absent; posterior edge of pronotum with sublateral notches present; pronotosternal sutures open, hypomeral bead present. ***Mesothorax*.** Scutellum rounded posterad or pointed (acuminate). Elytra. Anterior edge variable in shape, integument with metallic reflections or unmarked. ***Aedeagus*.** Parameres with apical lateral expansions present, without setae.

**Summary.** Sensory elements beginning on antennomere III, pronotum with setae on anterior half directed anterad or mesad, pronotosternal sutures open.

***S.mendax* (LeConte), and similar species**

**Habitus.** Body length 6–12 mm. ***Head*.** Supra-antennal carinae fading on frons, or complete across frons but not concave below in lateral view. Antennae with sensory elements beginning on antennomere IV. ***Prothorax*.** Pronotum with punctures simple; hind angle carinae present or absent; posterior edge of pronotum with sublateral notches present; pronotosternal sutures open, hypomeral bead present. Prosternum with sides straight or concave at midlength. ***Mesothorax*.** Scutellum rounded posterad. Elytra. Integument with metallic reflections or unmarked. ***Aedeagus*.** Parameres with apical lateral expansions present, without setae.

**Summary.** Sensory elements beginning on antennomere IV, pronotum non-metallic, with setae on anterior half not directed posterad, some setae directed mesad near midlength, sublateral notches present, pronotosternal sutures open.

**Genus *Tesolasomus* Johnson, 2021**

**Habitus.** Body length 6–18 mm. ***Head*.** Antennae with sensory elements beginning on antennomere IV. ***Prothorax*.** Pronotum with punctures simple; hind angle carinae present; posterior edge of pronotum with sublateral notches present; pronotosternal sutures closed, hypomeral bead absent. Prosternum with sides straight or concave at midlength. ***Mesothorax*.** Scutellum rounded posterad. Elytra. Integument marked with spots or bands in some. ***Aedeagus*.** Parameres with apical lateral expansions present, without setae.

**Summary.** Sensory elements beginning on antennomere IV, pronotum with setae on anterior half directed posterad, hind angles with sublateral notches present, hypomeral bead absent (not raised, punctate), mesocoxal cavities open, parameres without setae.

***T.morulus* (LeConte) and *T.deceptor* (Brown)**

**Habitus.** Body length 6–12 mm. ***Head*.** Antennae with sensory elements beginning on antennomere IV. ***Prothorax*.** Pronotum with punctures simple; hind angle carinae present; posterior edge of pronotum with sublateral notches present; pronotosternal sutures closed, hypomeral bead present; hypomera with concavity on posterior edge near hind angle. Prosternum with sides straight or concave at midlength. ***Mesothorax*.** Scutellum rounded posterad. Elytra. Integument unmarked; setal vestiture sparse on disk or even and mainly parallel. **Ventrites**. Ventrite V apex bisinuate with paired setal brushes (larger in males). ***Aedeagus*.** Parameres with apical lateral expansions present; each paramere with three or more setae.

**Summary.** Supra-antennal carinae fading on frons, sensory elements beginning on antennomere IV, hind angles with sublateral notches present, ventrite V apex bisinuate in ventral view with paired setal brushes. Species with hypomeral bead present and modified ventrite V treated as distinctive here.

**Tribe Dendrometrini**

**Habitus.** Body length 3–30 mm. ***Head*.** Prognathous in most; most with supra-antennal carinae joining medially forming a shelf across the frons with a concavity below in lateral view, frons with triangular depression in some. Antennae with 11 antennomeres, not pectinate. ***Prothorax*.** Pronotum without tubercles between punctures in most, pronotal lateral carinae complete, visible throughout length in dorsal view in most, microserrate near hind angles in some; posterior crenellations absent, posterior edge of hind angles not defined by a dorsal carina in some; most with setae directed anterad throughout; pronotosternal sutures straight in most. ***Mesothorax*.** Scutellum with posterior edge rounded in most. Elytra. Striae present in most, setal vestiture even and mainly parallel, elytral integument unmarked with spots or transverse bands in most, setae not forming color pattern in most. ***Legs*.** Tarsal pads or membranous lobes present in many. Tarsal claws simple. **Ventrites**. Microserration at sides (e.g., 100 points per mm) present in some. Ventrite V arcuate apicad, without setal brushes. ***Aedeagus*.** Parameres with more than three setae each in most.

**Notes.** It is difficult to define this group collectively based on adult morphology. Most with supra-antennal carinae continuous and raised across frons and some with tarsal pads lobed. Some with hind angles of prothorax depressed or not defined mesally by a right-angled edge (carina) in dorsal view.

**Genus *Athous* Eschscholtz, 1829**

**Habitus.** Body length 3–30 mm. ***Head*.** Supra-antennal carinae joining medially (forming shelf), nasale (head capsule below edge of frontal carina) with outline concave in lateral view; frons with triangular depression in most. ***Prothorax*.** Pronotum with lateral carina microserrate in some; pronotosternal sutures closed, hypomeron with posterior edge near hind angle without concavity in most. Prosternum with sides straight at midlength in most. ***Mesothorax*.** Scutellum with posterior edge rounded in most; mesocoxal cavities fully open in most. Elytra. Striae present; integument unmarked in most; without pattern from differences in setal color. ***Legs*.** Tarsal pads or membranous lobes present on multiple tarsomeres in most (II and III). ***Aedeagus*.** Parameres with apical lateral expansions present in most; each paramere with three or more setae in most.

Diagnostic summaries are provided separately here for Becker’s (1979) species groups and European subgenera present in North America.

***A.brightwelli*(Kirby) group**

**Habitus.** Body length 6–18 mm. ***Head*.** Antennal sensory elements beginning on antennomere III. ***Prothorax*.** Pronotum with punctures simple, lateral carina microserrate at hind angles only (posterior 10%, in concavity); hind angle carinae absent; posterior edge of pronotum with sublateral notches in some; hypomeral bead present. Prosternal process not curved upward ≥ 40° in lateral view. ***Mesothorax*.** Scutellum with anterior edge concave. Elytra. Anterior edge straight to arcuate or sinuate (recurved) or with rectangular projection near humeri in dorsal view. ***Legs*.** Metacoxal plate reaching side. **Ventrites**. Sides not microserrate.

**Summary.** Supra-antennal carina complete and elevated, frons with broad semi-triangular impression across much of dorsal surface, sensory elements beginning on antennomere III (widespread rough texture and/or apical sensorium), pronotum without dorsal hind angle carinae, tarsomeres II and III with lobes, ventrites not microserrate at sides.

***A.campyloides*Newman group**

**Habitus.** Body length 6–18 mm. ***Head*.** Antennal sensory elements beginning on antennomere IV. ***Prothorax*.** Pronotum with punctures umbilicate in some, lateral carina microserrate along entire side (e.g., ~ 70 points per mm) or at hind angles only (posterior 10%, in concavity); hind angle carinae absent; posterior edge of pronotum with sublateral notches present; hypomeral bead present. Prosternal process not curved upward ≥ 40° in lateral view. ***Mesothorax*.** Scutellum with anterior edge not concave. Elytra. Anterior edge sinuate (recurved) in dorsal view. ***Legs*.** Metacoxal plate reaching side or not. **Ventrites**. Sides not microserrate.

**Summary.** Supra-antennal carina complete and elevated, frons with broad semi-triangular impression across much of dorsal surface, sensory elements beginning on antennomere IV (apical sensorium), pronotum without dorsal hind angle carinae, tarsomeres II and III with lobes, ventrites not microserrate at sides.

***A.cucullatus* (Say) group**

**Habitus.** Body length 6–12 mm. ***Head*.** Antennal sensory elements beginning on antennomere III. ***Prothorax*.** Pronotum with some punctures umbilicate, lateral carina microserrate along entire side (e.g., ~ 70 points per mm); hind angle carinae present or absent; posterior edge of pronotum with sublateral notches in some; hypomeral bead absent. Prosternum with sides straight or convex at midlength; prosternal process not curved upward ≥ 40° in lateral view. ***Mesothorax*.** Scutellum with anterior edge concave or not, posterior edge rounded (arcuate) or pointed (acuminate). Elytra. Anterior edge straight to arcuate or sinuate (recurved) in dorsal view. ***Legs*.** Metacoxal plate reaching side or not. **Ventrites**. Microserration at sides (e.g., 100 points per mm) present or absent. ***Aedeagus*.** Parameres without apical lateral expansions.

**Summary.** Supra-antennal carina complete and elevated, frons with broad semi-triangular impression across much of dorsal surface, lateral carina microserrate for much of length (best observed from below, not necessary to see), pronotal hind angles with dorsal carina (weak in some), multiple tarsomeres with lobes, parameres without preapical expansions.

***A.imitans* Fall group**

**Habitus.** Body length 3–12 mm. ***Head*.** Frons without triangular depression. Antennal sensory elements beginning on antennomere IV. ***Prothorax*.** Pronotum with punctures simple, lateral carina microserrate at hind angles only (posterior 10%, in concavity) or not serrate; hind angle carinae absent; posterior edge of pronotum without sublateral notches; pronotosternal sutures closed. Prosternal process not curved upward ≥ 40° in lateral view. ***Mesothorax*.** Mesocoxal cavities open to mesepimeron only. Scutellum with anterior edge concave. Elytra. Anterior edge straight to arcuate near humeri in dorsal view, integument marked with spots or bands in some. ***Legs*.** Metacoxal plate not reaching side. **Ventrites**. Sides not microserrate.

**Summary.** Supra-antennal carina complete and elevated, frons without triangular impression, pronotal hind angles without dorsal carina, posterior edge depressed and without basal notches, prosternal process not ascendant, multiple tarsomeres with lobes.

***A.productus* (Randall) group**

**Habitus.** Body length 12–18 mm. ***Head*.** Antennal sensory elements beginning on antennomere III. ***Prothorax*.** Pronotum with some punctures umbilicate, lateral carinae not serrate; hind angle carinae absent; posterior edge of pronotum without sublateral notches; hypomeral bead present; hypomera with arcuate concavity on posterior edge near hind angle. Prosternum with sides straight or concave at midlength; prosternal process not curved upward ≥ 40° in lateral view. ***Mesothorax*.** Scutellum with anterior edge not concave, posterior edge rounded (arcuate) or straight (truncate). Elytra. Anterior edge straight to arcuate near humeri in dorsal view. ***Legs*.** Metacoxal plate reaching side. **Ventrites**. Sides not microserrate.

**Summary.** Supra-antennal carina complete and elevated, frons with broad triangular impression across much of dorsal surface, pronotum without hind angle carinae, lateral carinae not microserrate (even at hind angles), multiple tarsomeres with lobes.

***A.rufifrons* (Randall) group**

**Habitus.** Body length 12–18 mm. ***Head*.** Antennal sensory elements beginning on antennomere III. ***Prothorax*.** Pronotum with some punctures umbilicate, lateral carina microserrate at hind angles only (posterior 10%, in concavity); hind angle carinae absent; posterior edge of pronotum with sublateral notches present; hypomeral bead present. Prosternal process not curved upward ≥ 40° in lateral view. ***Mesothorax*.** Scutellum with anterior edge concave. Elytra. Anterior edge sinuate (recurved) in dorsal view. ***Legs*.** Metacoxal plate not reaching side; tarsal pads absent. **Ventrites**. Microserration at sides (e.g., 100 points per mm) present.

**Summary.** Supra-antennal carina complete and elevated, frons with broad triangular impression across much of dorsal surface, pronotum without hind angle carinae, tarsi without ventral lobes, claws without basal setae, ventrites microserrate (especially apex of ventrite V).

***A.scapularis* (Say) group**

**Habitus.** Body length 6–18 mm. ***Head*.** Antennal sensory elements beginning on antennomere III or IV. ***Prothorax*.** Pronotum with some punctures umbilicate, lateral carina microserrate along entire side (e.g., ~ 70 points per mm) or at hind angles only (posterior 10%, in concavity); hind angle carinae present (single); posterior edge of pronotum without sublateral notches; hypomeral bead absent. Prosternal process not curved upward ≥ 40° in lateral view. ***Mesothorax*.** Scutellum with anterior edge concave, posterior edge rounded (arcuate) or pointed (acuminate). Elytra. Anterior edge straight to arcuate near humeri in dorsal view. ***Legs*.** Metacoxal plate reaching side. **Ventrites**. Sides not microserrate.

**Summary.** Supra-antennal carinae complete and elevated, lateral carinae microserrate at least near hind angle, pronotal hind angles with dorsal carina, multiple tarsomeres with lobes, parameres with preapical expansions.

***A.scissus* LeConte group**

**Habitus.** Body length 12–30 mm. ***Head*.** Antennal sensory elements beginning on antennomere III or IV. ***Prothorax*.** Pronotum with some punctures umbilicate, lateral carina microserrate at hind angles only (posterior 10%, in concavity); hind angle carinae absent; posterior edge of pronotum with sublateral notches present; pronotosternal sutures closed. ***Mesothorax*.** Scutellum with anterior edge not concave, posterior edge rounded (arcuate) or pointed (acuminate). Elytra. Anterior edge straight to arcuate or sinuate (recurved) in dorsal view. ***Legs*.** Metacoxal plate reaching side or not. **Ventrites**. Microserration at sides (e.g., 100 points per mm) present.

**Summary.** Supra-antennal carina complete and elevated, frons with broad semi-triangular impression across much of dorsal surface, sensory elements beginning on antennomere III in most or all (apical sensorium), pronotum without dorsal hind angle carinae, lateral carina briefly microserrate near hind angle, multiple tarsomeres with lobes, ventrites not microserrate at sides.

***Athous*, subgenus Athous**

**Habitus.** Body length 10–15 mm. ***Head*.** Frons without triangular depression. Antennal sensory elements beginning on antennomere IV. ***Prothorax*.** Pronotum with punctures umbilicate in some, lateral carina microserrate along entire side (e.g., ~ 70 points per mm); hind angle carinae absent; posterior edge of pronotum with sublateral notches present; pronotosternal sutures closed. Prosternal process not curved upward ≥ 40° in lateral view. ***Mesothorax*.** Scutellum with anterior edge concave. Elytra. Anterior edge straight to arcuate near humeri in dorsal view. ***Legs*.** Metacoxal plate reaching side or not. **Ventrites**. Microserration at sides (e.g., 100 points per mm) present. ***Aedeagus*.** Parameres without setae or each paramere with three or more setae.

**Summary.** Supra-antennal carina complete and elevated (slightly), frons without triangular impression (weak transverse anterior impression only), pronotum with lateral carina microserrate throughout (best seen in lateroventral view), multiple tarsomeres lobed. For *A.haemorrhoidalis* (Fabricius).

**Genus *Barrelater* Johnson, 2014**

**Habitus.** Body length 12–18 mm. ***Head*.** Supra-antennal carinae joining medially (forming shelf), nasale (head capsule below edge of frontal carina) with outline concave in lateral view; frons without triangular depression. Antennal sensory elements beginning on antennomere III. ***Prothorax*.** Pronotum with some punctures umbilicate, lateral carina microserrate at hind angles only (posterior 10%, in concavity); hind angle carinae absent; posterior edge of pronotum with sublateral notches present; pronotosternal sutures closed, hypomeral bead absent; hypomeron with posterior edge near hind angle without concavity. Prosternum with sides straight at midlength; prosternal process curved upward > 40° in lateral view. ***Mesothorax*.** Mesocoxal cavities open. Scutellum with anterior edge concave. Elytra. Striae present; anterior edge straight to arcuate near humeri in dorsal view. ***Legs*.** Metacoxal plate reaching side; tarsal pads or membranous lobes present on multiple tarsomeres (or appearing absent), (I, II, III, and IV or II, III, and IV). **Ventrites**. Sides not microserrate. ***Aedeagus*.** Parameres with apical lateral expansions present; each paramere with three or more setae.

**Summary.** Supra-antennal carina complete and elevated, frons without triangular impression, pronotum with umbilicate punctures, dorsal hind angles without carinae, lateral carinae briefly microserrate near hind angle. Incompletely separated from *Hemicrepidius* here.

**Genus *Denticollis* Piller & Mitterpacher, 1783**

**Habitus.** Body length 7–13 mm. ***Head*.** Supra-antennal carinae joining medially (forming shelf), nasale (head capsule below edge of frontal carina) with outline concave in lateral view; nasale (head capsule below edge of frontal carina) with outline concave in lateral view; frons with triangular depression. Antennal sensory elements beginning on antennomere III. ***Prothorax*.** Pronotum with punctures umbilicate in some, lateral carinae not serrate; hind angle carinae absent; posterior edge of pronotum without sublateral notches; pronotosternal sutures closed, hypomeral bead present; hypomeron with posterior edge near hind angle without concavity. ***Mesothorax*.** Mesocoxal cavities open. Scutellum with anterior edge concave or not. Elytra. Anterior edge straight to arcuate or sinuate (recurved) in dorsal view. ***Legs*.** Metacoxal plate reaching side or not; tarsal pads or membranous lobes absent (or present on tarsomere IV only). **Ventrites**. Sides not microserrate. ***Aedeagus*.** Parameres with apical lateral expansions present.

**Summary.** Supra-antennal carina complete and elevated (concave below in lateral view), frons with triangular impression, pronotum without dorsal hind angle carinae, tarsomeres not lobed (or tarsomere IV weakly lobed in some), ventrites not microserrate at sides.

**Genus *Diacanthous* Reitter, 1852**

**Habitus.** Body length 10–15 mm. ***Head*.** Supra-antennal carinae joining medially (forming shelf), nasale (head capsule below edge of frontal carina) with outline concave in lateral view; nasale (head capsule below edge of frontal carina) with outline concave in lateral view; frons with triangular depression. Antennal sensory elements beginning on antennomere III. ***Prothorax*.** Pronotum with some punctures umbilicate, lateral carina microserrate along entire side (e.g., ~ 70 points per mm); hind angle carinae absent; posterior edge of pronotum without sublateral notches; pronotosternal sutures closed, hypomeral bead absent; hypomera with arcuate concavity on posterior edge near hind angle. Prosternum with sides straight or concave at midlength. ***Mesothorax*.** Mesocoxal cavities open. Scutellum with anterior edge not concave. Elytra. Striae present; anterior edge straight to arcuate near humeri in dorsal view; with pattern from differences in setal color. ***Legs*.** Metacoxal plate reaching side; tarsal pads or membranous lobes present on multiple tarsomeres, (I, II, III, and IV or II, III, and IV). **Ventrites**. Sides not microserrate. ***Aedeagus*.** Parameres without apical lateral expansions.

**Summary.** Supra-antennal carina complete and elevated (concave below in lateral view), frons with triangular impression, elytra with diagonal bands due to differences in setal color, setae simple (not scale-like).

**Genus *Elathous* Reitter, 1890**

**Habitus.** Body length 6–12 mm. ***Head*.** Supra-antennal carinae joining medially (forming shelf), nasale (head capsule below edge of frontal carina) with outline concave in lateral view; nasale (head capsule below edge of frontal carina) with outline concave in lateral view; prognathous, rarely hypognathous; frons with triangular depression. Antennal sensory elements beginning on antennomere IV. ***Prothorax*.** Pronotum with some punctures umbilicate, lateral carinae not serrate; hind angle carinae present (single); posterior edge of pronotum with sublateral notches in some; pronotosternal sutures open, hypomeral bead present; hypomeron with posterior edge near hind angle without concavity. ***Mesothorax*.** Mesocoxal cavities not closed. Scutellum with anterior edge not concave. Elytra. Striae present; anterior edge straight to arcuate near humeri in dorsal view. ***Legs*.** Metacoxal plate reaching side; tarsal pads absent. **Ventrites**. Microserration at sides (e.g., 100 points per mm) present or absent. ***Aedeagus*.** Parameres with apical lateral expansions present.

**Summary.** Supra-antennal carina complete and elevated (concave below in lateral view), frons with broad impression, sensory elements beginning on antennomere IV (dorsal and ventral sensoria), hypomeron without concavity on posterior edge, tarsi not lobed.

**Genus *Euplastius* Schwarz, 1903**

**Habitus.** Body length 6–12 mm. ***Head*.** Supra-antennal carinae fading on frons; frons without triangular depression. Antennal sensory elements beginning on antennomere III. ***Prothorax*.** Pronotum with punctures umbilicate in some; lateral carinae hidden anteriorly in dorsal view in some, not serrate; hind angle carinae present (single) or absent; posterior edge of pronotum without sublateral notches; pronotosternal sutures closed, hypomera with posterrior concavity long and arcuate. Prosternum with sides straight or convex at midlength; prosternal process not curved upward ≥ 40° in lateral view. ***Mesothorax*.** Mesocoxal cavities open. Scutellum with anterior edge not concave. Elytra. Striae present; anterior edge straight to arcuate or sinuate (recurved) or with rectangular projection near humeri in dorsal view. ***Legs*.** Metacoxal plate reaching side; tarsal pads absent. **Ventrites**. Sides not microserrate. ***Aedeagus*.** Parameres with apical lateral expansions present, without setae.

**Summary.** Supra-antennal carinae fading on frons, sensory elements beginning on antennomere III (widespread with short erect pubescence), pronotum punctures umbilicate, setae near midline on anterior half directed mesad, sublateral notches absent, posterior edge of pronotum not defined by a transverse carina near hind angles.

**Genus *Gambrinus* LeConte, 1853**

**Habitus.** Body length 6–18 mm. ***Head*.** Nasale (head capsule below edge of frontal carina) with outline concave in lateral view; frons without triangular depression. Antennal sensory elements beginning on antennomere IV. ***Prothorax*.** Pronotum with punctures umbilicate in some; lateral carinae hidden anteriorly in dorsal view in some, not microserrate; hind angle carinae present (single); posterior edge of pronotum without sublateral notches; pronotosternal sutures open or closed, hypomeral bead present; hypomera with arcuate or angulate concavity on posterior edge near hind angle. Prosternum with sides straight or concave at midlength. ***Mesothorax*.** Mesocoxal cavities not closed. Scutellum with anterior edge not concave, posterior edge rounded (arcuate) or pointed (acuminate). Elytra. Striae present; anterior edge straight to arcuate near humeri in dorsal view, integument marked with spots or transverse bands in some. ***Legs*.** Metacoxal plate reaching side; tarsal pads absent. **Ventrites**. Microserration at sides (e.g., 100 points per mm) present. ***Aedeagus*.** Parameres with apical lateral expansions present or absent.

**Summary.** Supra-antennal carina complete and elevated (concave below in lateral view), frons without triangular impression, pronotum with lateral carina complete, sublateral notches absent, hypomeron with concavity on posterior edge, tarsi not lobed, ventrites microserrate at sides.

***G.bicolor*Van Dyke**

**Habitus.** Body length 5–10 mm. ***Head*.** Nasale (head capsule below edge of frontal carina) with outline concave in lateral view; frons with triangular depression. Antennal sensory elements beginning on antennomere IV. ***Prothorax*.** Pronotum with some punctures umbilicate; lateral carinae hidden anteriorly in dorsal view in some, not serrate; hind angle carinae present (single); posterior edge of pronotum without sublateral notches; pronotosternal sutures open, hypomeral bead present; hypomera with arcuate concavity on posterior edge near hind angle. Prosternum with sides straight at midlength; prosternal process not curved upward ≥ 40° in lateral view. ***Mesothorax*.** Mesocoxal cavities open. Scutellum with anterior edge not concave, posterior edge rounded (arcuate) or pointed (acuminate). Elytra. Striae present; anterior edge straight to arcuate near humeri in dorsal view. ***Legs*.** Metacoxal plate reaching side; tarsal pads absent. **Ventrites**. Microserration at sides (e.g., 100 points per mm) present. ***Aedeagus*.** Parameres with apical lateral expansions present.

**Summary.** Supra-antennal carina complete and elevated (concave below in lateral view), frons with triangular impression, pronotum without sublateral notches, hypomeron with concavity on posterior edge, ventrites microserrate at sides. Treated as distinctive here to improve diagnostic effectiveness, because of triangular depression on frons.

**Genus *Hemicrepidius* Germar, 1839**

**Habitus.** Body length 12–30 mm. ***Head*.** Supra-antennal carinae joining medially (forming shelf) or reaching anterior edge of head capsule; frons without triangular depression. Antennal sensory elements beginning on antennomere III or IV. ***Prothorax*.** Pronotum with punctures simple, lateral carina microserrate at hind angles only (posterior 10%, in concavity) or not serrate; hind angle carinae present (single); posterior edge of pronotum with sublateral notches in some; pronotosternal sutures closed; hypomeron with posterior edge near hind angle with concavity in most. ***Mesothorax*.** Mesocoxal cavities open. Scutellum with anterior edge concave or not, posterior edge rounded (arcuate) or pointed (acuminate). Elytra. Striae present; anterior edge straight to arcuate or sinuate (recurved) in dorsal view, integument unmarked. ***Legs*.** Metacoxal plate reaching side or not; tarsal pads or membranous lobes present on multiple tarsomeres, (I, II, III, and IV or II, III, and IV). **Ventrites**. Microserration at sides (e.g., 100 points per mm) present or absent. ***Aedeagus*.** Parameres with apical lateral expansions present.

**Summary.** Supra-antennal carina complete or joining anterior edge of head capsule, frons without triangular impression, pronotum wider than long, hind angle not microserrate or microserrate only in concavity at hind angle, pronotosternal sutures closed, multiple tarsomeres lobed. Pronotal hind angle carinae present (inconspicuous in some), hypomeron notched near hind angle in most. Incompletely distinguished from *Barrelater* here.

**Genus *Limonius* Eschscholtz, 1829**

**Habitus.** Body length 3–12 mm. ***Head*.** Supra-antennal carinae joining medially (forming shelf); frons without triangular depression. Antennal sensory elements beginning on antennomere IV. ***Prothorax*.** Pronotum with punctures umbilicate in some; lateral carinae hidden anteriorly in dorsal view in some, not serrate; hind angle carinae present (single) or absent; posterior edge of pronotum without sublateral notches; pronotosternal sutures open or closed, hypomeral bead present; hypomeron with posterior edge near hind angle without concavity. ***Mesothorax*.** Mesocoxal cavities not closed. Scutellum with anterior edge not concave. Elytra. Striae present; anterior edge straight to arcuate near humeri in dorsal view, integument marked with spots or bands in some. ***Legs*.** Metacoxal plate reaching side or not; tarsal pads absent. **Ventrites**. Microserration at sides (e.g., 100 points per mm) present. ***Aedeagus*.** Each paramere with three or more setae.

**Summary.** Supra-antennal carina complete and elevated, frons without triangular impression, hypomeron without concavity on posterior edge, scutellum rounded posterad, tarsi not lobed, ventrites microserrate at sides.

***L.brevis*Van Dyke**

**Habitus.** Body length 5–8 mm. ***Head*.** Nasale (head capsule below edge of frontal carina) with outline concave in lateral view; frons without triangular depression. Antennal sensory elements beginning on antennomere IV. ***Prothorax*.** Pronotum with punctures simple, with or without tubercles between punctures; lateral carinae hidden anteriorly in dorsal view in some, not serrate; hind angle carinae present (single) or absent; posterior edge of pronotum without sublateral notches; pronotosternal sutures open, hypomeral bead present; hypomeron with posterior edge near hind angle without concavity. Prosternum with sides straight or concave at midlength; prosternal process curved upward > 40° in lateral view. ***Mesothorax*.** Mesocoxal cavities open to mesepimeron only. Scutellum with anterior edge not concave. Elytra. Striae present; anterior edge straight to arcuate near humeri in dorsal view. ***Legs*.** Metacoxal plate reaching side; small tarsal pads or membranous lobes present on multiple tarsomeres (II and III). **Ventrites**. Microserration at sides (e.g., 100 points per mm) present. ***Aedeagus*.** Parameres with apical lateral expansions present.

**Summary.** Supra-antennal carina complete and elevated (concave below in lateral view), pronotosternal sutures open anterad, hypomeron without concavity posterad, tarsi lobed on tarsomeres II and III (short apicoventral lobes). Included as distinct sub-group here to improve diagnostic effectiveness because of lobed tarsomeres.

**Genus *Pheletes* Kiesenwetter, 1858**

**Habitus.** Body length 5–7 mm. ***Head*.** Supra-antennal carinae joining medially (forming shelf); frons without triangular depression. Antennal sensory elements beginning on antennomere IV. ***Prothorax*.** Pronotum with punctures simple, lateral carinae not serrate; hind angle carinae present (single); posterior edge of pronotum without sublateral notches; pronotosternal sutures closed, hypomeral bead present; hypomeron with posterior edge near hind angle without concavity. ***Mesothorax*.** Mesocoxal cavities not closed. Scutellum with anterior edge not concave, posterior edge pointed (acuminate). Elytra. Striae present. ***Legs*.** Metacoxal plate reaching side; tarsal pads absent. **Ventrites**. Microserration at sides (e.g., 100 points per mm) present. ***Aedeagus*.** Parameres with apical lateral expansions present.

**Summary.** Supra-antennal carina complete and elevated (concave below in lateral view), hypomeron without concavity near hind angle, scutellum pointed posterad, tarsomeres simple, parameres with preapical lateral expansions.

**Genus *Tetralimonius* Etzler, 2019**

**Habitus.** Body length 3–6 mm. ***Head*.** Nasale (head capsule below edge of frontal carina) with outline concave in lateral view; frons without triangular depression. Antennal sensory elements beginning on antennomere IV. ***Prothorax*.** Pronotum with punctures umbilicate in some, lateral carinae not serrate; hind angle carinae present (single) or absent; posterior edge of pronotum without sublateral notches; pronotosternal sutures open, hypomeral bead present; hypomeron with posterior edge near hind angle without concavity. ***Mesothorax*.** Mesocoxal cavities not closed. Scutellum with anterior edge not concave, posterior edge pointed (acuminate). Elytra. Striae present; anterior edge straight to arcuate near humeri in dorsal view, integument marked with spots or bands in many. ***Legs*.** Metacoxal plate reaching side; tarsal pads absent. **Ventrites**. Microserration at sides (e.g., 100 points per mm) present. ***Aedeagus*.** Parameres without apical lateral expansions; each paramere with one or two setae.

**Summary.** Supra-antennal carina complete and elevated (concave below in lateral view), hypomeron without concavity near hind angle, scutellum pointed posterad, tarsomeres simple, parameres without preapical lateral expansions.

**Genus *Vittathous* Johnson, 2021**

**Habitus.** Body length 12–18 mm. ***Head*.** Supra-antennal carinae fading on frons or reaching anterior edge of head capsule; frons without triangular depression. Antennal sensory elements beginning on antennomere III. ***Prothorax*.** Pronotum with punctures large but simple, lateral carinae not serrate; hind angle carinae present (single); posterior edge of pronotum without sublateral notches; pronotosternal sutures closed, hypomeral bead absent; hypomeron with posterior edge near hind angle without concavity. Prosternal process not curved upward ≥ 40° in lateral view. ***Mesothorax*.** Mesocoxal cavities open. Scutellum with anterior edge concave or not. Elytra. Striae present; anterior edge straight to arcuate near humeri in dorsal view, integument with longitudinal bands. ***Legs*.** Metacoxal plate reaching side; tarsal pads absent. **Ventrites**. Sides not microserrate. ***Aedeagus*.** Parameres with apical lateral expansions present; each paramere with two setae.

**Summary.** Sensory elements beginning on antennomere III, pronotum with setae near midline on anterior half directed mesad, sublateral notches absent (posterior edge of pronotum not defined by a transverse carina), parameres with two setae.

## ﻿Conclusions

A first interactive key is provided for genera of Elateridae from Canada and the USA. This publication also summarizes species habitat requirements at multiple scales, and includes the most comprehensive descriptive information for genera of Elateridae from Canada and USA. Diversity of elaterid genera was high throughout warm and cool temperate regions, especially in mountainous areas and mesic woodlands. Larvae of most genera were associated with soil, litter, and decaying wood. We invite colleagues to borrow this descriptive format for their own generic descriptions and diagnoses for Elateridae. We ask researchers to cite this work in the methods section of manuscripts where it was used for generic identifications as outlined by Packer et al. (2018).

## ﻿Additional information

### Author ORCIDs

Hume B. Douglas https://orcid.org/0000-0003-1722-7554

### Data availability

All of the data that support the findings of this study are available in the main text or Supplementary Information.

## Supplementary materials

Supplementary material 1 Key to the genera of Nearctic Elateridae v. 1.0, LIF3 file Data type php Explanation note This XML-based format allows exchange of the key with other key developers. This format can be imported directly LUCID and used as a non-illustrated interactive key. https://binary.pensoft.net/file/1042934This dataset is made available under the Open Database License (http://opendatacommons.org/licenses/odbl/1.0/). The Open Database License (ODbL) is a license agreement intended to allow users to freely share, modify, and use this Dataset while maintaining this same freedom for others, provided that the original source and author(s) are credited.Hume B. Douglas, Frank E. Etzler, Paul J. Johnson, H.E. James Hammond

Supplementary material 2 Key to the genera of Nearctic Elateridae v.- 1.0, CSV file Data type php Explanation note This comma-separated values file may be used to exchange the Lucid key with other CSV-compliant applications. https://binary.pensoft.net/file/1042935This dataset is made available under the Open Database License (http://opendatacommons.org/licenses/odbl/1.0/). The Open Database License (ODbL) is a license agreement intended to allow users to freely share, modify, and use this Dataset while maintaining this same freedom for others, provided that the original source and author(s) are credited.Hume B. Douglas, Frank E. Etzler, Paul J. Johnson, H.E. James Hammond

Supplementary material 3 Informal descriptions for Nearctic Elateridae v. 1.0, .PDF file Data type pdf Explanation note This PDF file includes expanded diagnostic information for all Nearctic elaterid genera or distinctive subsets of species, including all information used in the online key. These often differ from formal diagnoses used to globally define these groups. However, in some cases these are more informative than original diagnoses. https://binary.pensoft.net/file/1042936This dataset is made available under the Open Database License (http://opendatacommons.org/licenses/odbl/1.0/). The Open Database License (ODbL) is a license agreement intended to allow users to freely share, modify, and use this Dataset while maintaining this same freedom for others, provided that the original source and author(s) are credited.Hume B. Douglas, Frank E. Etzler, Paul J. Johnson, H.E. James Hammond
