# Supplementary material for: ﻿Matrix-based key to the click beetle genera of Canada and USA with a summary of habitat use (Coleoptera, Elateridae)
Source: Zookeys. 2024 May 7;1200:75–144. doi: 10.3897/zookeys.1200.119315 (PMC11094399; doi:10.3897/zookeys.1200.119315)
Supplement: Supplementary material 3 — Informal descriptions for Nearctic Elateridae v. 1.0, .PDF file [file zookeys-1200-075_article-119315__-s003.pdf]

Supplementary Material 3. Full-length informal descriptions for genera and parts of genera of Elateridae for Canada and USA. These are natural language descriptions generated using LucidBuilder 4.0.6 software (Identic Ltd. 2020).

Matrix-based key to the click beetle genera of Canada and USA with a summary of habitat use (Coleoptera: Elateridae)

Authors: Hume B. Douglas, Frank E. Etzler, Paul J. Johnson, H.E. James Hammond.

1 DRAPETES Dejean, 1821

Habitus. Body Length 1-5 mm or 5-10 mm. Vestiture. Scale like setae absent. **Head.** Supra-antennal carinae joining medially (forming shelf), fading on frons (not reaching another structure) or directed anteriorly (reaching anterior part of head capsule); nasale (head capsule below edge of frontal carina) with outline not concave in lateral view; hypognathous (labrum oriented downward 90 degrees or more); frons without triangular depression. Antennae with 11 antennomeres, not pectinate, sensory elements beginning on antennomere IV. **Prothorax.** Pronotum wider than long at widest point (including hind angles); dorsal punctures uniform sized, all simple (floor of puncture concave), without tubercles or longitudinal carinae between punctures; pronotal lateral carina complete anteriorly, carina visible throughout length in dorsal view or carina not visible anteriorly in dorsal view, meeting anterior edge of prothorax at about 90 degrees in lateral view, not serrate; bioluminescent spots absent; hind angle carinae present (single); posterior edge of pronotum with sublateral plicae and notches absent, crenellations absent; hypomeron posterior edge near each hind angle with or without concavity; pronotosternal sutures excavated (able to contain antennae). Prosternum with sides straight at midlength in ventral view; prosternal process not curved upward more than 40 degrees in lateral view. **Mesothorax.** Mesocoxal cavity open to mesepimeron only; mesoventral cavity without serration along sides. Elytra. Striae present or absent; anterior edge outline straight to arcuate near humeri in dorsal view; integument marked with spots or transverse bands or unmarked with spots or tranverse bands; with or without pattern from differences in setal colour; setal vestiture mainly absent on disk or even and mainly parallel. **Legs.** Metacoxal plate with or without elongation in mesal half, plate reaching lateral edge; tarsal pads or membranous lobes present on multiple tarsomeres, (I, II, III, and IV or II, III, and IV); tarsal claws without setae, with two points, or appendiculate. **Ventrites.** Microserration at sides (e.g. 100 points per mm) absent, ventrite 5 apex arcuate without paired setal brushes. **Aedeagus.** Parameres with articulation at base, apical lateral expansions absent. **Geography.** Known from Ontario, Quebec, Arizona+ (AZ, NM), Texas, Michigan+ (MN, WI, MI), Indiana+ (IL, IN, OH), Arkansas+ (KS, MO, OK, AR, LA, MS), Vermont+ (ME, VT, NH), Massachusetts+ (MA, CT, RI), New York, Pennsylvania+ (PA, WV), Virginia+ (NJ, DE, MD, DC, VA), Carolinas+ (KY, TN, NC, SC, GA, AL) and Florida ['+' refers to several states or provinces collectively].

2 BLADUS LeConte, 1861

Habitus. Body Length 1-5 mm or 5-10 mm. Vestiture. Scale like setae absent. **Head.** Supra-antennal carinae fading on frons (not reaching another structure) or absent; hypognathous (labrum oriented downward 90 degrees or more); frons without triangular depression; gena not broadened anteriorly below eye, not extending spine-like anterior to basal tubercle of mandible (ventral condyle). Antennae with 11 antennomeres, not pectinate, sensory elements beginning on antennomere IV. **Prothorax.** Pronotum wider than long at widest point (including hind angles); dorsal punctures uniform sized, some or all punctures umbilicate (floor of puncture flat), without tubercles or longitudinal

carinae between punctures; pronotal lateral carina complete anteriorly, carina visible throughout length in dorsal view, meeting anterior edge of prothorax at about 90 degrees in lateral view, not serrate; bioluminescent spots absent; hind angle carinae absent; posterior edge of pronotum with sublateral plicae and notches absent, crenellations absent; hypomeron posterior edge near each hind angle without concavity; pronotosternal sutures closed, hypomeral beads present. Prosternum with sides straight at midlength in ventral view; prosternal process curved upward more than 40 degrees in lateral view. **Mesothorax.** Mesocoxal cavity open; mesoventral cavity without serration along sides. Elytra. Striae present; anterior edge outline straight to arcuate near humeri in dorsal view; integument unmarked with spots or tranverse bands; without pattern from differences in setal colour; setal vestiture even and mainly parallel. **Legs.** Metacoxal plate without elongation in mesal half, plate reaching lateral edge; tarsal pads or membranous lobes absent; tarsal claws without setae, simple. **Ventrites.** Microserration at sides (e.g. 100 points per mm) absent, ventrite 5 apex arcuate without paired setal brushes. **Aedeagus.** Parameres with articulation at base, apical lateral expansions absent. **Geography.** Known from Texas, Indiana+ (IL, IN, OH), Arkansas+ (KS, MO, OK, AR, LA, MS) and Carolinas+ (KY, TN, NC, SC, GA, AL) ['+' refers to several states or provinces collectively].

2 OESTODES LeConte, 1853

Habitus. Body Length 5-10 mm or 10-15 mm. Vestiture. Scale like setae absent. **Head.** Supra-antennal carinae fading on frons (not reaching another structure); hypognathous (labrum oriented downward 90 degrees or more); frons without triangular depression; gena not broadened anteriorly below eye, not extending spine-like anterior to basal tubercle of mandible (ventral condyle). Antennae with 11 antennomeres, not pectinate, sensory elements beginning on antennomere III. **Prothorax.** Pronotum longer than wide at widest point (including hind angles); dorsal punctures uniform sized, all simple (floor of puncture concave), without tubercles or longitudinal carinae between punctures; pronotal lateral carina complete anteriorly, carina visible throughout length in dorsal view or carina not visible anteriorly in dorsal view, meeting anterior edge of prothorax at about 90 degrees in lateral view, not serrate; bioluminescent spots absent; hind angle carinae present (single); posterior edge of pronotum with sublateral plicae and notches present, crenellations absent; hypomeron posterior edge near each hind angle with concavity, concavity arcuate; pronotosternal sutures open or closed, hypomeral beads present or absent. Prosternum with sides concave at midlength in ventral view; prosternal process curved upward more than 40 degrees in lateral view. **Mesothorax.** Mesocoxal cavity open; mesoventral cavity without serration along sides. Elytra. Striae present or absent; anterior edge outline straight to arcuate near humeri in dorsal view; integument unmarked with spots or tranverse bands; without pattern from differences in setal colour; setal vestiture mainly absent on disk or even and mainly parallel. **Legs.** Metacoxal plate without elongation in mesal half, plate reaching lateral edge; tarsal pads or membranous lobes absent; tarsal claws without setae, simple. **Ventrites.** Microserration at sides (e.g. 100 points per mm) absent, ventrite 5 apex arcuate without paired setal brushes. **Aedeagus.** Parameres with articulation at base, apical lateral expansions present; parameres without setae or each paramere with three or more setae. **Geography.** Known from Alberta, Manitoba+ (MB, SK), Ontario, Quebec, New Brunswick+ (NB, NS, PE), Montana+ (MT, WY, ND), Nebraska+ (SD, NE, IA), Michigan+ (MN, WI, MI), Vermont+ (ME, VT, NH), Massachusetts+ (MA, CT, RI), New York, Pennsylvania+ (PA, WV), Virginia+ (NJ, DE, MD, DC, VA) and Carolinas+ (KY, TN, NC, SC, GA, AL) ['+' refers to several states or provinces collectively].

3.0 MELANOTUS Eschscholtz, 1829

Habitus. Body Length 5-10 mm, 10-15 mm, 15-20 mm or 20-40 mm. Vestiture. Scale like setae absent. **Head.** Supra-antennal carinae joining medially (forming shelf); nasale (head capsule below edge of frontal carina) with outline concave in lateral view; hypognathous (labrum oriented downward 90 degrees or more) or prognathous (labrum oriented less than 90 degrees downward); frons without triangular depression; gena not broadened anteriorly below eye, not extending spine-like anterior to basal tubercle of mandible (ventral condyle). Antennae with 11 antennomeres, not pectinate, sensory elements beginning on antennomere IV. **Prothorax.** Pronotum wider than long at widest point (including hind angles); dorsal punctures uniform sized or of two distinct intermixed sizes (heterogeneous), some or all punctures umbilicate (floor of puncture flat), without tubercles or longitudinal carinae between punctures; pronotal lateral carina complete anteriorly, carina visible throughout length in dorsal view or carina not visible anteriorly in dorsal view, meeting anterior edge of prothorax at about 90 degrees in lateral view, not serrate; bioluminescent spots absent; hind angle carinae present (single) or present (two carinae); posterior edge of pronotum with sublateral plicae and notches present, crenellations absent; hypomeron posterior edge near each hind angle with concavity, concavity arcuate or angulate; pronotosternal sutures open, hypomeral beads present. Prosternum with sides concave at midlength in ventral view; prosternal process not curved upward more than 40 degrees in lateral view. **Mesothorax.** Mesocoxal cavity open or open to mesepimeron only; mesoventral cavity without serration along sides. Elytra. Striae present; anterior edge outline straight to arcuate or sinuate (recurved) or with rectangular projection near humeri in dorsal view; integument unmarked with spots or tranverse bands; without pattern from differences in setal colour; setal vestiture even and mainly parallel. **Legs.** Metacoxal plate without elongation in mesal half, plate reaching or not reaching lateral edge; tarsal pads or membranous lobes absent; tarsal claws without setae, with 3 or more points. **Ventrites.** Microserration at sides (e.g. 100 points per mm) absent, ventrite 5 apex arcuate without paired setal brushes. **Aedeagus.** Parameres with articulation at base, apical lateral expansions present or absent; each paramere with three or more setae. **Geography.** Known from British Columbia, Manitoba+ (MB, SK), Ontario, Quebec, New Brunswick+ (NB, NS, PE), Newfoundland and Labrador, Oregon+ (WA, OR, ID), California, Montana+ (MT, WY, ND), Colorado+ (NV, UT, CO), Arizona+ (AZ, NM), Nebraska+ (SD, NE, IA), Texas, Michigan+ (MN, WI, MI), Indiana+ (IL, IN, OH), Arkansas+ (KS, MO, OK, AR, LA, MS), Vermont+ (ME, VT, NH), Massachusetts+ (MA, CT, RI), New York, Pennsylvania+ (PA, WV), Virginia+ (NJ, DE, MD, DC, VA), Carolinas+ (KY, TN, NC, SC, GA, AL) and Florida ['+' refers to several states or provinces collectively].

3.1 BLAUTA LeConte, 1853

Habitus. Body Length 5-10 mm, 10-15 mm or 15-20 mm. Vestiture. Scale like setae absent. **Head.** Supra-antennal carinae joining medially (forming shelf); nasale (head capsule below edge of frontal carina) with outline concave in lateral view; hypognathous (labrum oriented downward 90 degrees or more); frons without triangular depression; gena not broadened anteriorly below eye, not extending spine-like anterior to basal tubercle of mandible (ventral condyle). Antennae with 11 antennomeres, not pectinate, sensory elements beginning on antennomere III or IV. **Prothorax.** Pronotum wider than long at widest point (including hind angles); dorsal punctures uniform sized, some or all punctures umbilicate (floor of puncture flat), without tubercles or longitudinal carinae between punctures; pronotal lateral carina complete anteriorly or incomplete anteriorly, carina not visible anteriorly in dorsal view, meeting anterior edge of prothorax at about 90 degrees in lateral view, not serrate; bioluminescent spots absent; hind angle carinae present (single); posterior edge of pronotum with sublateral plicae and notches present or absent, crenellations absent; hypomeron posterior edge near each hind angle with concavity, concavity arcuate; pronotosternal sutures open, hypomeral beads present or absent. Prosternum with sides concave at midlength in ventral view; prosternal process not curved upward more than 40 degrees

in lateral view. **Mesothorax.** Mesocoxal cavity open; mesoventral cavity without serration along sides. Elytra. Striae present; anterior edge outline straight to arcuate near humeri in dorsal view; integument unmarked with spots or tranverse bands; without pattern from differences in setal colour; setal vestiture even and mainly parallel. **Legs.** Metacoxal plate without elongation in mesal half, plate reaching or not reaching lateral edge; tarsal pads or membranous lobes present on multiple tarsomeres, (II and III); tarsal claws without setae, simple. **Ventrites.** Microserration at sides (e.g. 100 points per mm) absent, ventrite 5 apex arcuate without paired setal brushes. **Aedeagus.** Parameres with articulation at base, apical lateral expansions present; each paramere with three or more setae. **Geography.** Known from California, Texas, Arkansas+ (KS, MO, OK, AR, LA, MS), Virginia+ (NJ, DE, MD, DC, VA), Carolinas+ (KY, TN, NC, SC, GA, AL) and Florida ['+' refers to several states or provinces collectively].

3.1 DICREPIDIUS Eschscholtz, 1829

Habitus. Body Length 10-15 mm. Vestiture. Scale like setae absent. **Head.** Supra-antennal carinae joining medially (forming shelf); nasale (head capsule below edge of frontal carina) with outline not concave in lateral view; hypognathous (labrum oriented downward 90 degrees or more), rarely prognathous (labrum oriented less than 90 degrees downward) (by misinterpretation); frons without triangular depression; gena not broadened anteriorly below eye, not extending spine-like anterior to basal tubercle of mandible (ventral condyle). Antennae with 11 antennomeres, pectinate or bipectinate or not pectinate, sensory elements beginning on antennomere III. **Prothorax.** Pronotum wider than long or longer than wide at widest point (including hind angles); dorsal punctures uniform sized, some or all punctures umbilicate (floor of puncture flat), without tubercles or longitudinal carinae between punctures; pronotal lateral carina complete anteriorly, carina not visible anteriorly in dorsal view, meeting anterior edge of prothorax at about 90 degrees in lateral view, not serrate; bioluminescent spots absent; hind angle carinae present (single); posterior edge of pronotum with sublateral plicae and notches present, crenellations absent; hypomeron posterior edge near each hind angle with concavity, concavity arcuate; pronotosternal sutures open, hypomeral beads present. Prosternum with sides concave at midlength in ventral view; prosternal process not curved upward more than 40 degrees in lateral view. **Mesothorax.** Mesocoxal cavity open; mesoventral cavity with serration along sides. Elytra. Striae present; anterior edge outline straight to arcuate near humeri in dorsal view; integument unmarked with spots or tranverse bands; without pattern from differences in setal colour; setal vestiture even and mainly parallel. **Legs.** Metacoxal plate without elongation in mesal half; tarsal pads or membranous lobes present on multiple tarsomeres, (II and III); tarsal claws without setae, simple. **Ventrites.** Microserration at sides (e.g. 100 points per mm) absent, ventrite 5 apex arcuate without paired setal brushes. **Aedeagus.** Parameres with articulation at base, apical lateral expansions present; each paramere with three or more setae. **Geography.** Known from California, Colorado+ (NV, UT, CO), Arizona+ (AZ, NM), Texas, Indiana+ (IL, IN, OH), Arkansas+ (KS, MO, OK, AR, LA, MS), Virginia+ (NJ, DE, MD, DC, VA), Carolinas+ (KY, TN, NC, SC, GA, AL) and Florida ['+' refers to several states or provinces collectively].

3.1 DIPROPUS Germar, 1839

Habitus. Body Length 5-10 mm, 10-15 mm or 15-20 mm. Vestiture. Scale like setae absent. **Head.** Supra-antennal carinae joining medially (forming shelf); nasale (head capsule below edge of frontal carina) with outline concave in lateral view; hypognathous (labrum oriented downward 90 degrees or more); frons without triangular depression; gena not broadened anteriorly below eye, not extending spine-like anterior to basal tubercle of mandible (ventral condyle). Antennae with 11 antennomeres, not pectinate, sensory elements beginning on antennomere III. **Prothorax.** Pronotum wider than long at

widest point (including hind angles); dorsal punctures uniform sized, some or all punctures umbilicate (floor of puncture flat), without tubercles or longitudinal carinae between punctures; pronotal lateral carina complete anteriorly, carina not visible anteriorly in dorsal view, meeting anterior edge of prothorax at about 90 degrees in lateral view, not serrate; bioluminescent spots absent; hind angle carinae present (single); posterior edge of pronotum with sublateral plicae and notches present, crenellations absent; hypomeron posterior edge near each hind angle with concavity, concavity arcuate; pronotosternal sutures open, hypomeral beads present. Prosternum with sides concave at midlength in ventral view; prosternal process curved or not curved upward more than 40 degrees in lateral view. **Mesothorax.** Mesocoxal cavity open; mesoventral cavity without serration along sides. Elytra. Striae present; anterior edge outline straight to arcuate near humeri in dorsal view; integument unmarked with spots or tranverse bands; without pattern from differences in setal colour; setal vestiture even and mainly parallel. **Legs.** Metacoxal plate with or without elongation in mesal half, plate reaching or not reaching lateral edge; tarsal pads or membranous lobes present on multiple tarsomeres, (II and III); tarsal claws without setae, simple. **Ventrites.** Microserration at sides (e.g. 100 points per mm) absent, ventrite 5 apex arcuate without paired setal brushes. **Aedeagus.** Parameres with articulation at base, apical lateral expansions present; each paramere with three or more setae. **Geography.** Known from California, Arizona+ (AZ, NM), Texas, Michigan+ (MN, WI, MI), Arkansas+ (KS, MO, OK, AR, LA, MS), Vermont+ (ME, VT, NH), Massachusetts+ (MA, CT, RI), New York, Pennsylvania+ (PA, WV), Virginia+ (NJ, DE, MD, DC, VA), Carolinas+ (KY, TN, NC, SC, GA, AL) and Florida ['+' refers to several states or provinces collectively].

3.2 AMPEDUS Dejean, 1833

Habitus. Body Length 1-5 mm, 5-10 mm or 10-15 mm. Vestiture. Scale like setae absent. **Head.** Supra-antennal carinae joining medially (forming shelf), fading on frons (not reaching another structure) or directed anteriorly (reaching anterior part of head capsule); nasale (head capsule below edge of frontal carina) with outline concave or not concave in lateral view; hypognathous (labrum oriented downward 90 degrees or more), rarely prognathous (labrum oriented less than 90 degrees downward) (by misinterpretation); frons without triangular depression; gena not broadened anteriorly below eye, not extending spine-like anterior to basal tubercle of mandible (ventral condyle). Antennae with 11 antennomeres, not pectinate, sensory elements beginning on antennomere IV. **Prothorax.** Pronotum wider than long at widest point (including hind angles); dorsal punctures uniform sized, all simple (floor of puncture concave) or some or all punctures umbilicate (floor of puncture flat), without tubercles or longitudinal carinae between punctures; pronotal lateral carina complete anteriorly, carina visible throughout length in dorsal view or carina not visible anteriorly in dorsal view, meeting anterior edge of prothorax at about 90 degrees in lateral view, not serrate; bioluminescent spots absent; hind angle carinae present (single) or present (two carinae); posterior edge of pronotum with sublateral plicae and notches absent, crenellations absent; hypomeron posterior edge near each hind angle with concavity, concavity arcuate; pronotosternal sutures open, hypomeral beads present. Prosternum with sides straight or concave at midlength in ventral view; prosternal process curved or not curved upward more than 40 degrees in lateral view. **Mesothorax.** Mesocoxal cavity open; mesoventral cavity without serration along sides. Elytra. Striae present; anterior edge outline straight to arcuate near humeri in dorsal view; integument marked with spots or transverse bands, marked with spot or band in apical 2/5 only or unmarked with spots or tranverse bands; without pattern from differences in setal colour; setal vestiture even and mainly parallel. **Legs.** Metacoxal plate with elongation in mesal half, plate reaching lateral edge; tarsal pads or membranous lobes absent; tarsal claws without setae, simple. **Ventrites.** Microserration at sides (e.g. 100 points per mm) absent, ventrite 5 apex arcuate without paired setal brushes. **Aedeagus.** Parameres with articulation at base, apical lateral expansions

present; each paramere with three or more setae. **Geography.** Known from Nunavut+ (NT, NU), Yukon Territory, British Columbia, Alberta, Manitoba+ (MB, SK), Ontario, Quebec, New Brunswick+ (NB, NS, PE), Newfoundland and Labrador, Alaska, Oregon+ (WA, OR, ID), California, Montana+ (MT, WY, ND), Colorado+ (NV, UT, CO), Arizona+ (AZ, NM), Nebraska+ (SD, NE, IA), Texas, Michigan+ (MN, WI, MI), Indiana+ (IL, IN, OH), Arkansas+ (KS, MO, OK, AR, LA, MS), Vermont+ (ME, VT, NH), Massachusetts+ (MA, CT, RI), New York, Pennsylvania+ (PA, WV), Virginia+ (NJ, DE, MD, DC, VA), Carolinas+ (KY, TN, NC, SC, GA, AL) and Florida ['+' refers to several states or provinces collectively].

3.3 ANCHASTUS LeConte, 1853

Habitus. Body Length 1-5 mm, 5-10 mm or 10-15 mm. Vestiture. Scale like setae absent. **Head.** Supra-antennal carinae joining medially (forming shelf); nasale (head capsule below edge of frontal carina) with outline concave in lateral view; hypognathous (labrum oriented downward 90 degrees or more); frons without triangular depression; gena not broadened anteriorly below eye, not extending spine-like anterior to basal tubercle of mandible (ventral condyle). Antennae with 11 antennomeres, not pectinate, sensory elements beginning on antennomere IV. **Prothorax.** Pronotum wider than long or longer than wide at widest point (including hind angles); dorsal punctures uniform sized, all simple (floor of puncture concave) or some or all punctures umbilicate (floor of puncture flat), without tubercles or longitudinal carinae between punctures; pronotal lateral carina complete anteriorly, carina not visible anteriorly in dorsal view, meeting anterior edge of prothorax at about 90 degrees in lateral view, not serrate; bioluminescent spots absent; hind angle carinae present (single) or present (two carinae); posterior edge of pronotum with sublateral plicae and notches present or absent, crenellations absent; hypomeron posterior edge near each hind angle with concavity, concavity arcuate; pronotosternal sutures open or closed, hypomeral beads present. Prosternum with sides concave at midlength in ventral view; prosternal process curved or not curved upward more than 40 degrees in lateral view. **Mesothorax.** Mesocoxal cavity open; mesoventral cavity without serration along sides. Elytra. Striae present; anterior edge outline straight to arcuate near humeri in dorsal view; integument marked with spots or transverse bands or unmarked with spots or tranverse bands; without pattern from differences in setal colour; setal vestiture even and mainly parallel. **Legs.** Metacoxal plate with elongation in mesal half, plate reaching or not reaching lateral edge; tarsal pads or membranous lobes present on tasomere III only; tarsal claws without setae, simple. **Ventrites.** Microserration at sides (e.g. 100 points per mm) absent, ventrite 5 apex arcuate without paired setal brushes. **Aedeagus.** Parameres with articulation at base, apical lateral expansions present; each paramere with three or more setae. **Geography.** Known from British Columbia, Alberta, Manitoba+ (MB, SK), Oregon+ (WA, OR, ID), California, Montana+ (MT, WY, ND), Colorado+ (NV, UT, CO), Arizona+ (AZ, NM), Texas, Arkansas+ (KS, MO, OK, AR, LA, MS), Pennsylvania+ (PA, WV), Virginia+ (NJ, DE, MD, DC, VA), Carolinas+ (KY, TN, NC, SC, GA, AL) and Florida ['+' refers to several states or provinces collectively].

3.3 PHYSORHINUS Germar, 1840

Habitus. Body Length 5-10 mm or 10-15 mm. Vestiture. Scale like setae absent. **Head.** Supra-antennal carinae joining medially (forming shelf); nasale (head capsule below edge of frontal carina) with outline concave in lateral view; hypognathous (labrum oriented downward 90 degrees or more); frons without triangular depression; gena not broadened anteriorly below eye, not extending spine-like anterior to basal tubercle of mandible (ventral condyle). Antennae with 11 antennomeres, not pectinate, sensory elements beginning on antennomere IV. **Prothorax.** Pronotum wider than long at widest point (including hind angles); dorsal punctures uniform sized, all simple (floor of puncture concave) or some or all punctures umbilicate (floor of puncture flat), without tubercles or longitudinal carinae between

punctures; pronotal lateral carina complete anteriorly, carina visible throughout length in dorsal view or carina not visible anteriorly in dorsal view, meeting anterior edge of prothorax at about 90 degrees in lateral view, not serrate; bioluminescent spots absent; hind angle carinae present (single); posterior edge of pronotum with sublateral plicae and notches absent, crenellations absent; hypomeron posterior edge near each hind angle with concavity, concavity arcuate; pronotosternal sutures open, hypomeral beads present. Prosternum with sides concave at midlength in ventral view; prosternal process not curved upward more than 40 degrees in lateral view. **Mesothorax.** Mesocoxal cavity open or open to mesepimeron only; mesoventral cavity without serration along sides. Elytra. Striae present; anterior edge outline straight to arcuate near humeri in dorsal view; integument marked with spots or transverse bands or unmarked with spots or tranverse bands; without pattern from differences in setal colour; setal vestiture even and mainly parallel. **Legs.** Metacoxal plate with elongation in mesal half, plate reaching lateral edge; tarsal pads or membranous lobes present on tasomere III only; tarsal claws without setae, simple. **Ventrites.** Microserration at sides (e.g. 100 points per mm) absent, ventrite 5 apex arcuate without paired setal brushes. **Aedeagus.** Parameres with articulation at base, apical lateral expansions absent; each paramere with three or more setae. **Geography.** Known from Arizona+ (AZ, NM) and Texas ['+' refers to several states or provinces collectively].

3.4 MEGAPENTHES Kiesenwetter, 1858

Habitus. Body Length 1-5 mm, 5-10 mm, 10-15 mm or 15-20 mm. Vestiture. Scale like setae absent. **Head.** Supra-antennal carinae joining medially (forming shelf) or directed anteriorly (reaching anterior part of head capsule); nasale (head capsule below edge of frontal carina) with outline concave or not concave in lateral view; hypognathous (labrum oriented downward 90 degrees or more), rarely prognathous (labrum oriented less than 90 degrees downward) (by misinterpretation); frons without triangular depression; gena not broadened anteriorly below eye, not extending spine-like anterior to basal tubercle of mandible (ventral condyle) or broadened anteriorly below eye, extending spine-like anterior to basal tubercle of mandible (ventral condyle). Antennae with 11 antennomeres, not pectinate, sensory elements beginning on antennomere III or IV. **Prothorax.** Pronotum wider than long at widest point (including hind angles); dorsal punctures uniform sized, some or all punctures umbilicate (floor of puncture flat), without tubercles or longitudinal carinae between punctures; pronotal lateral carina complete anteriorly, carina not visible anteriorly in dorsal view, meeting anterior edge of prothorax at about 90 degrees in lateral view, not serrate; bioluminescent spots absent; hind angle carinae present (single); posterior edge of pronotum with sublateral plicae and notches present or absent, crenellations absent; hypomeron posterior edge near each hind angle with concavity, concavity arcuate or angulate; pronotosternal sutures closed, hypomeral beads present. Prosternum with sides concave at midlength in ventral view; prosternal process not curved upward more than 40 degrees in lateral view. **Mesothorax.** Mesocoxal cavity open; mesoventral cavity without serration along sides. Elytra. Striae present; anterior edge outline straight to arcuate near humeri in dorsal view; integument marked with spots or transverse bands, marked with spot or band in apical 2/5 only or unmarked with spots or tranverse bands; with or without pattern from differences in setal colour; setal vestiture even and mainly parallel. **Legs.** Metacoxal plate with elongation in mesal half, plate reaching or not reaching lateral edge; tarsal pads or membranous lobes absent; tarsal claws without setae, simple. **Ventrites.** Microserration at sides (e.g. 100 points per mm) absent, ventrite 5 apex arcuate without paired setal brushes. **Aedeagus.** Parameres with articulation at base, apical lateral expansions present or absent; each paramere with three or more setae. **Geography.** Known from British Columbia, Alberta, Manitoba+ (MB, SK), Ontario, Quebec, New Brunswick+ (NB, NS, PE), Alaska, Oregon+ (WA, OR, ID), California, Montana+ (MT, WY, ND), Colorado+ (NV, UT, CO), Arizona+ (AZ, NM), Nebraska+ (SD, NE, IA), Texas, Michigan+ (MN, WI, MI), Indiana+ (IL, IN, OH), Arkansas+ (KS, MO, OK, AR, LA, MS), Vermont+

(ME, VT, NH), Massachusetts+ (MA, CT, RI), New York, Pennsylvania+ (PA, WV), Virginia+ (NJ, DE, MD, DC, VA), Carolinas+ (KY, TN, NC, SC, GA, AL) and Florida ['+' refers to several states or provinces collectively].

3.5.0 APLASTUS LeConte, 1859

Habitus. Body Length 10-15 mm, 15-20 mm or 20-40 mm. Vestiture. Scale like setae absent. **Head.** Supra-antennal carinae fading on frons (not reaching another structure) or absent; prognathous (labrum oriented less than 90 degrees downward), rarely hypognathous (labrum oriented downward 90 degrees or more) (by misinterpretation); frons without triangular depression; gena not broadened anteriorly below eye, not extending spine-like anterior to basal tubercle of mandible (ventral condyle). Antennae with 11 antennomeres, not pectinate, sensory elements beginning on antennomere IV. **Prothorax.** Pronotum wider than long or longer than wide at widest point (including hind angles); dorsal punctures uniform sized, all simple (floor of puncture concave), without tubercles or longitudinal carinae between punctures; pronotal lateral carina complete anteriorly or incomplete anteriorly, carina visible throughout length in dorsal view, meeting anterior edge of prothorax at about 90 degrees in lateral view, not serrate; bioluminescent spots absent; hind angle carinae present (single); posterior edge of pronotum with sublateral plicae and notches absent, crenellations absent; hypomeron posterior edge near each hind angle with concavity, concavity arcuate; pronotosternal sutures open or closed, hypomeral beads present or absent. Prosternum with sides concave at midlength in ventral view; prosternal process curved upward more than 40 degrees in lateral view. **Mesothorax.** Mesocoxal cavity open; mesoventral cavity without serration along sides. Elytra. Striae present or absent; anterior edge outline straight to arcuate near humeri in dorsal view; integument unmarked with spots or tranverse bands; without pattern from differences in setal colour; setal vestiture even and mainly parallel. **Legs.** Metacoxal plate without elongation in mesal half, plate reaching or not reaching lateral edge; tarsal pads or membranous lobes absent; tarsal claws without setae, simple. **Ventrites.** Microserration at sides (e.g. 100 points per mm) absent, ventrite 5 apex arcuate without paired setal brushes. **Aedeagus.** Parameres with articulation at base, apical lateral expansions absent; each paramere with three or more setae. **Geography.** Known from California, Colorado+ (NV, UT, CO) and Arizona+ (AZ, NM) ['+' refers to several states or provinces collectively].

3.5.0 EUTHYSANIUS LeConte, 1853

Habitus. Body Length 15-20 mm or 20-40 mm. Vestiture. Scale like setae absent. **Head.** Supra-antennal carinae absent; prognathous (labrum oriented less than 90 degrees downward); frons with or without triangular depression; gena not broadened anteriorly below eye, not extending spine-like anterior to basal tubercle of mandible (ventral condyle). Antennae with 12 antennomeres, pectinate or bipectinate or not pectinate, sensory elements beginning on antennomere IV. **Prothorax.** Pronotum wider than long at widest point (including hind angles); dorsal punctures uniform sized, all simple (floor of puncture concave) or some or all punctures umbilicate (floor of puncture flat), without tubercles or longitudinal carinae between punctures; pronotal lateral carina complete anteriorly or incomplete anteriorly, carina visible throughout length in dorsal view or carina not visible anteriorly in dorsal view, meeting anterior edge of prothorax at about 90 degrees in lateral view, not serrate; bioluminescent spots absent; hind angle carinae present (single); posterior edge of pronotum with sublateral plicae and notches absent, crenellations absent; hypomeron posterior edge near each hind angle with or without concavity, concavity arcuate; pronotosternal sutures open or closed, hypomeral beads present. Prosternum with sides concave at midlength in ventral view; prosternal process curved upward more than 40 degrees in lateral view. **Mesothorax.** Mesocoxal cavity open; mesoventral cavity without serration along sides.

Elytra. Striae present; anterior edge outline straight to arcuate near humeri in dorsal view; integument unmarked with spots or tranverse bands; without pattern from differences in setal colour; setal vestiture even and mainly parallel. **Legs.** Metacoxal plate without elongation in mesal half, plate not reaching lateral edge; tarsal pads or membranous lobes absent; tarsal claws without setae, simple. **Ventrites.** Microserration at sides (e.g. 100 points per mm) absent, ventrite 5 apex arcuate without paired setal brushes. **Aedeagus.** Parameres with articulation at base, apical lateral expansions absent; each paramere with three or more setae. **Geography.** Known from California and Arizona+ (AZ, NM) ['+' refers to several states or provinces collectively].

3.5.0 OCTINODES Candèze, 1863

Habitus. Body Length 5-10 mm, 10-15 mm or 15-20 mm. Vestiture. Scale like setae absent. **Head.** Supra-antennal carinae absent; prognathous (labrum oriented less than 90 degrees downward); frons without triangular depression; gena not broadened anteriorly below eye, not extending spine-like anterior to basal tubercle of mandible (ventral condyle). Antennae with 11 antennomeres, pectinate or bipectinate or not pectinate, sensory elements beginning on antennomere IV. **Prothorax.** Pronotum wider than long or longer than wide at widest point (including hind angles); dorsal punctures uniform sized, all simple (floor of puncture concave), without tubercles or longitudinal carinae between punctures; pronotal lateral carina complete anteriorly or incomplete anteriorly, carina visible throughout length in dorsal view, meeting anterior edge of prothorax at about 90 degrees in lateral view, not serrate; bioluminescent spots absent; hind angle carinae present (single) or absent; posterior edge of pronotum with sublateral plicae and notches absent, crenellations absent; hypomeron posterior edge near each hind angle with or without concavity; pronotosternal sutures open or closed, hypomeral beads present. Prosternum with sides concave at midlength in ventral view; prosternal process curved upward more than 40 degrees in lateral view. **Mesothorax.** Mesocoxal cavity open; mesoventral cavity without serration along sides. Elytra. Striae present; anterior edge outline straight to arcuate near humeri in dorsal view; integument unmarked with spots or tranverse bands; without pattern from differences in setal colour; setal vestiture even and mainly parallel. **Legs.** Metacoxal plate without elongation in mesal half, plate not reaching lateral edge; tarsal pads or membranous lobes absent; tarsal claws without setae, simple. **Ventrites.** Microserration at sides (e.g. 100 points per mm) absent, ventrite 5 apex arcuate without paired setal brushes. **Aedeagus.** Parameres with articulation at base, apical lateral expansions absent; each paramere with three or more setae. **Geography.** Known from California and Texas ['+' refers to several states or provinces collectively].

3.5.1 SCAPTOLENUS LeConte, 1853

Habitus. Body Length 10-15 mm, 15-20 mm or 20-40 mm. Vestiture. Scale like setae absent. **Head.** Supra-antennal carinae absent; prognathous (labrum oriented less than 90 degrees downward); frons without triangular depression; gena not broadened anteriorly below eye, not extending spine-like anterior to basal tubercle of mandible (ventral condyle). Antennae with 11 antennomeres, not pectinate, sensory elements beginning on antennomere IV. **Prothorax.** Pronotum wider than long at widest point (including hind angles); dorsal punctures uniform sized, all simple (floor of puncture concave), without tubercles or longitudinal carinae between punctures; pronotal lateral carina incomplete anteriorly, not serrate; bioluminescent spots absent; hind angle carinae absent; posterior edge of pronotum with sublateral plicae and notches absent, crenellations absent; hypomeron posterior edge near each hind angle with concavity, concavity arcuate; pronotosternal sutures closed, hypomeral beads absent. Prosternum with sides concave at midlength in ventral view; prosternal process curved upward more than 40 degrees in lateral view. **Mesothorax.** Mesocoxal cavity open;

mesoventral cavity without serration along sides. Elytra. Striae absent; anterior edge outline straight to arcuate near humeri in dorsal view; integument unmarked with spots or tranverse bands; without pattern from differences in setal colour; setal vestiture even and mainly parallel. **Legs.** Metacoxal plate with or without elongation in mesal half, plate not reaching lateral edge; tarsal pads or membranous lobes absent; tarsal claws without setae, simple. **Ventrites.** Microserration at sides (e.g. 100 points per mm) absent, ventrite 5 apex arcuate without paired setal brushes. **Aedeagus.** Parameres with articulation at base, apical lateral expansions absent; each paramere with two setae. **Geography.** Known from Arizona+ (AZ, NM), Texas and Arkansas+ (KS, MO, OK, AR, LA, MS) ['+' refers to several states or provinces collectively].

3.5.1 SELONODON Latreille, 1834

Habitus. Body Length 5-10 mm, 10-15 mm, 15-20 mm or 20-40 mm. Vestiture. Scale like setae absent. **Head.** Supra-antennal carinae absent; hypognathous (labrum oriented downward 90 degrees or more) or prognathous (labrum oriented less than 90 degrees downward); frons without triangular depression; gena not broadened anteriorly below eye, not extending spine-like anterior to basal tubercle of mandible (ventral condyle). Antennae with 11 antennomeres, not pectinate, sensory elements beginning on antennomere IV. **Prothorax.** Pronotum wider than long at widest point (including hind angles); dorsal punctures uniform sized, all simple (floor of puncture concave) or some or all punctures umbilicate (floor of puncture flat), without tubercles or longitudinal carinae between punctures; pronotal lateral carina incomplete anteriorly, not serrate; bioluminescent spots absent; hind angle carinae present (single) or absent; posterior edge of pronotum with sublateral plicae and notches absent, crenellations absent; hypomeron posterior edge near each hind angle with or without concavity, concavity arcuate; pronotosternal sutures closed, hypomeral beads present or absent. Prosternum with sides concave at midlength in ventral view; prosternal process curved or not curved upward more than 40 degrees in lateral view. **Mesothorax.** Mesocoxal cavity open; mesoventral cavity without serration along sides. Elytra. Striae present or absent; anterior edge outline straight to arcuate or sinuate (recurved) or with rectangular projection near humeri in dorsal view; integument unmarked with spots or tranverse bands; without pattern from differences in setal colour; setal vestiture even and mainly parallel. **Legs.** Metacoxal plate with or without elongation in mesal half, plate not reaching lateral edge; tarsal pads or membranous lobes absent; tarsal claws without setae, simple. **Ventrites.** Microserration at sides (e.g. 100 points per mm) absent, ventrite 5 apex arcuate without paired setal brushes. **Aedeagus.** Parameres with articulation at base, apical lateral expansions absent; parameres without setae or each paramere with three or more setae. **Geography.** Known from Colorado+ (NV, UT, CO), Arizona+ (AZ, NM), Michigan+ (MN, WI, MI), Arkansas+ (KS, MO, OK, AR, LA, MS), Carolinas+ (KY, TN, NC, SC, GA, AL) and Florida ['+' refers to several states or provinces collectively].

3.6 CAMPYLOMORPHUS Jacquelin du Val, 1860

Habitus. Body Length 5-10 mm. Vestiture. Scale like setae absent. **Head.** Supra-antennal carinae fading on frons (not reaching another structure) or directed anteriorly (reaching anterior part of head capsule); hypognathous (labrum oriented downward 90 degrees or more) or prognathous (labrum oriented less than 90 degrees downward); frons without triangular depression; gena not broadened anteriorly below eye, not extending spine-like anterior to basal tubercle of mandible (ventral condyle). Antennae with 11 antennomeres, not pectinate, sensory elements beginning on antennomere III. **Prothorax.** Pronotum wider than long at widest point (including hind angles); dorsal punctures uniform sized, all simple (floor of puncture concave), without tubercles or longitudinal carinae between punctures; pronotal lateral carina complete anteriorly, carina visible throughout length in dorsal view, meeting anterior edge of

prothorax at about 90 degrees in lateral view, not serrate; bioluminescent spots absent; hind angle carinae present (single); posterior edge of pronotum with sublateral plicae and notches absent, crenellations absent; hypomeron posterior edge near each hind angle with concavity, concavity arcuate; pronotosternal sutures closed, hypomeral beads present. Prosternum with sides concave at midlength in ventral view; prosternal process curved upward more than 40 degrees in lateral view. **Mesothorax.** Mesocoxal cavity open or open to mesepimeron only; mesoventral cavity without serration along sides. Elytra. Striae present; anterior edge outline straight to arcuate near humeri in dorsal view; integument unmarked with spots or tranverse bands; without pattern from differences in setal colour; setal vestiture even and mainly parallel. **Legs.** Metacoxal plate without elongation in mesal half, plate reaching lateral edge; tarsal pads or membranous lobes absent; tarsal claws without setae, simple. **Ventrites.** Microserration at sides (e.g. 100 points per mm) absent, ventrite 5 apex arcuate without paired setal brushes. **Aedeagus.** Parameres with articulation at base, apical lateral expansions present; each paramere with three or more setae. **Geography.** Known from British Columbia, Oregon+ (WA, OR, ID) and California ['+' refers to several states or provinces collectively].

3.6 DIPLOSTETHUS Schwarz, 1907

Habitus. Body Length 10-15 mm, 15-20 mm or 20-40 mm. Vestiture. Scale like setae absent. **Head.** Supra-antennal carinae fading on frons (not reaching another structure) or directed anteriorly (reaching anterior part of head capsule); hypognathous (labrum oriented downward 90 degrees or more); frons without triangular depression; gena broadened anteriorly below eye, extending spine-like anterior to basal tubercle of mandible (ventral condyle). Antennae with 11 antennomeres, not pectinate, sensory elements beginning on antennomere IV. **Prothorax.** Pronotum wider than long at widest point (including hind angles); dorsal punctures uniform sized, all simple (floor of puncture concave) or some or all punctures umbilicate (floor of puncture flat), without tubercles or longitudinal carinae between punctures; pronotal lateral carina complete anteriorly, carina visible throughout length in dorsal view, meeting anterior edge of prothorax at about 90 degrees in lateral view, not serrate; bioluminescent spots absent; hind angle carinae present (single); posterior edge of pronotum with sublateral plicae and notches absent, crenellations absent; hypomeron posterior edge near each hind angle with concavity, concavity arcuate; pronotosternal sutures closed, hypomeral beads absent. Prosternum with sides concave at midlength in ventral view; prosternal process not curved upward more than 40 degrees in lateral view. **Mesothorax.** Mesocoxal cavity open; mesoventral cavity without serration along sides. Elytra. Striae present or absent; anterior edge outline straight to arcuate near humeri in dorsal view; integument unmarked with spots or tranverse bands; without pattern from differences in setal colour; setal vestiture even and mainly parallel. **Legs.** Metacoxal plate with elongation in mesal half, plate reaching lateral edge; tarsal pads or membranous lobes absent; tarsal claws without setae, simple. **Ventrites.** Microserration at sides (e.g. 100 points per mm) absent, ventrite 5 apex arcuate without paired setal brushes. **Aedeagus.** Parameres with articulation at base, apical lateral expansions absent; each paramere with three or more setae. **Geography.** Known from California, Arizona+ (AZ, NM), Texas, Arkansas+ (KS, MO, OK, AR, LA, MS), Virginia+ (NJ, DE, MD, DC, VA), Carolinas+ (KY, TN, NC, SC, GA, AL) and Florida ['+' refers to several states or provinces collectively].

3.6 DOLEROSOMUS Motschulsky, 1859

Habitus. Body Length 5-10 mm. Vestiture. Scale like setae absent. **Head.** Supra-antennal carinae fading on frons (not reaching another structure) or directed anteriorly (reaching anterior part of head capsule); hypognathous (labrum oriented downward 90 degrees or more) or prognathous (labrum oriented less than 90 degrees downward); frons without triangular depression; gena not broadened anteriorly below

eye, not extending spine-like anterior to basal tubercle of mandible (ventral condyle) or broadened anteriorly below eye, extending spine-like anterior to basal tubercle of mandible (ventral condyle). Antennae with 11 antennomeres, not pectinate, sensory elements beginning on antennomere IV. **Prothorax.** Pronotum wider than long or longer than wide at widest point (including hind angles); dorsal punctures uniform sized, some or all punctures umbilicate (floor of puncture flat), without tubercles or longitudinal carinae between punctures; pronotal lateral carina complete anteriorly, carina not visible anteriorly in dorsal view, meeting anterior edge of prothorax at about 90 degrees in lateral view, not serrate; bioluminescent spots absent; hind angle carinae present (single); posterior edge of pronotum with sublateral plicae and notches present or absent, crenellations absent; hypomeron posterior edge near each hind angle with or without concavity, concavity arcuate or angulate; pronotosternal sutures closed, hypomeral beads present or absent. Prosternum with sides concave at midlength in ventral view; prosternal process not curved upward more than 40 degrees in lateral view. **Mesothorax.** Mesocoxal cavity open; mesoventral cavity without serration along sides. Elytra. Striae present; anterior edge outline straight to arcuate near humeri in dorsal view; integument unmarked with spots or tranverse bands; without pattern from differences in setal colour; setal vestiture even and mainly parallel. **Legs.** Metacoxal plate without elongation in mesal half, plate reaching lateral edge; tarsal pads or membranous lobes absent; tarsal claws without setae, simple. **Ventrites.** Microserration at sides (e.g. 100 points per mm) absent, ventrite 5 apex arcuate without paired setal brushes. **Aedeagus.** Parameres with articulation at base, apical lateral expansions present or absent; each paramere with three or more setae. **Geography.** Known from British Columbia, Ontario, Quebec, Oregon+ (WA, OR, ID), California, Colorado+ (NV, UT, CO), Michigan+ (MN, WI, MI), Indiana+ (IL, IN, OH), Arkansas+ (KS, MO, OK, AR, LA, MS), Massachusetts+ (MA, CT, RI), New York, Pennsylvania+ (PA, WV), Virginia+ (NJ, DE, MD, DC, VA), Carolinas+ (KY, TN, NC, SC, GA, AL) and Florida ['+' refers to several states or provinces collectively].

3.6 ELATER Linnaeus, 1758

Habitus. Body Length 10-15 mm or 15-20 mm. Vestiture. Scale like setae absent. **Head.** Supra-antennal carinae directed anteriorly (reaching anterior part of head capsule); hypognathous (labrum oriented downward 90 degrees or more) or prognathous (labrum oriented less than 90 degrees downward); frons without triangular depression; gena broadened anteriorly below eye, extending spine-like anterior to basal tubercle of mandible (ventral condyle). Antennae with 11 antennomeres, not pectinate, sensory elements beginning on antennomere IV. **Prothorax.** Pronotum wider than long at widest point (including hind angles); dorsal punctures uniform sized, all simple (floor of puncture concave), without tubercles or longitudinal carinae between punctures; pronotal lateral carina complete anteriorly or incomplete anteriorly, carina not visible anteriorly in dorsal view, meeting anterior edge of prothorax at about 90 degrees in lateral view, not serrate; bioluminescent spots absent; hind angle carinae present (single); posterior edge of pronotum with sublateral plicae and notches absent, crenellations absent; hypomeron posterior edge near each hind angle with concavity; pronotosternal sutures closed, hypomeral beads present. Prosternum with sides concave at midlength in ventral view; prosternal process not curved upward more than 40 degrees in lateral view. **Mesothorax.** Mesocoxal cavity open or open to mesepimeron only; mesoventral cavity without serration along sides. Elytra. Striae absent; anterior edge outline straight to arcuate near humeri in dorsal view; integument unmarked with spots or tranverse bands; without pattern from differences in setal colour; setal vestiture even and mainly parallel. **Legs.** Metacoxal plate with or without elongation in mesal half, plate reaching lateral edge; tarsal pads or membranous lobes absent; tarsal claws without setae, simple. **Ventrites.** Microserration at sides (e.g. 100 points per mm) absent, ventrite 5 apex arcuate without paired setal brushes. **Aedeagus.** Parameres with articulation at base, apical lateral expansions present or absent;

each paramere with three or more setae. **Geography.** Known from Manitoba+ (MB, SK), Ontario, Quebec, New Brunswick+ (NB, NS, PE), Oregon+ (WA, OR, ID), California, Montana+ (MT, WY, ND), Arizona+ (AZ, NM), Michigan+ (MN, WI, MI), Indiana+ (IL, IN, OH), Arkansas+ (KS, MO, OK, AR, LA, MS), Vermont+ (ME, VT, NH), Massachusetts+ (MA, CT, RI), New York, Pennsylvania+ (PA, WV), Virginia+ (NJ, DE, MD, DC, VA) and Carolinas+ (KY, TN, NC, SC, GA, AL) ['+' refers to several states or provinces collectively].

3.6 ORTHOSTETHUS Lacordaire, 1857

Habitus. Body Length 20-40 mm. Vestiture. Scale like setae absent. **Head.** Supra-antennal carinae directed anteriorly (reaching anterior part of head capsule); hypognathous (labrum oriented downward 90 degrees or more) or prognathous (labrum oriented less than 90 degrees downward); frons without triangular depression; gena broadened anteriorly below eye, extending spine-like anterior to basal tubercle of mandible (ventral condyle). Antennae with 11 antennomeres, pectinate or bipectinate or not pectinate, sensory elements beginning on antennomere IV. **Prothorax.** Pronotum wider than long at widest point (including hind angles); dorsal punctures uniform sized, all simple (floor of puncture concave), without tubercles or longitudinal carinae between punctures; pronotal lateral carina complete anteriorly, carina visible throughout length in dorsal view or carina not visible anteriorly in dorsal view, meeting anterior edge of prothorax at about 90 degrees in lateral view, not serrate; bioluminescent spots absent; hind angle carinae present (single); posterior edge of pronotum with sublateral plicae and notches absent, crenellations absent; hypomeron posterior edge near each hind angle with concavity, concavity arcuate; pronotosternal sutures closed, hypomeral beads present. Prosternum with sides concave at midlength in ventral view; prosternal process not curved upward more than 40 degrees in lateral view. **Mesothorax.** Mesocoxal cavity open; mesoventral cavity without serration along sides. Elytra. Striae absent; anterior edge outline straight to arcuate near humeri in dorsal view; integument unmarked with spots or tranverse bands; without pattern from differences in setal colour; setal vestiture even and mainly parallel. **Legs.** Metacoxal plate without elongation in mesal half, plate reaching lateral edge; tarsal pads or membranous lobes absent; tarsal claws without setae, simple. **Ventrites.** Microserration at sides (e.g. 100 points per mm) absent, ventrite 5 apex arcuate without paired setal brushes. **Aedeagus.** Parameres with articulation at base, apical lateral expansions absent; each paramere with three or more setae. **Geography.** Known from Arizona+ (AZ, NM), Texas, Indiana+ (IL, IN, OH), Arkansas+ (KS, MO, OK, AR, LA, MS), New York, Virginia+ (NJ, DE, MD, DC, VA), Carolinas+ (KY, TN, NC, SC, GA, AL) and Florida ['+' refers to several states or provinces collectively].

3.6 PARALLELOSTETHUS Schwarz, 1907

Habitus. Body Length 10-15 mm, 15-20 mm or 20-40 mm. Vestiture. Scale like setae absent. **Head.** Supra-antennal carinae fading on frons (not reaching another structure) or directed anteriorly (reaching anterior part of head capsule); hypognathous (labrum oriented downward 90 degrees or more) or prognathous (labrum oriented less than 90 degrees downward); frons without triangular depression; gena broadened anteriorly below eye, extending spine-like anterior to basal tubercle of mandible (ventral condyle). Antennae with 11 antennomeres, not pectinate, sensory elements beginning on antennomere IV. **Prothorax.** Pronotum wider than long at widest point (including hind angles); dorsal punctures uniform sized, all simple (floor of puncture concave), without tubercles or longitudinal carinae between punctures; pronotal lateral carina incomplete anteriorly, not serrate; bioluminescent spots absent; hind angle carinae present (single); posterior edge of pronotum with sublateral plicae and notches absent, crenellations absent; hypomeron posterior edge near each hind angle with concavity, concavity arcuate; pronotosternal sutures closed, hypomeral beads present.

Prosternum with sides concave at midlength in ventral view; prosternal process not curved upward more than 40 degrees in lateral view. **Mesothorax.** Mesocoxal cavity open; mesoventral cavity without serration along sides. Elytra. Striae absent; anterior edge outline straight to arcuate near humeri in dorsal view; integument unmarked with spots or tranverse bands; without pattern from differences in setal colour; setal vestiture even and mainly parallel. **Legs.** Metacoxal plate with elongation in mesal half, plate reaching lateral edge; tarsal pads or membranous lobes absent; tarsal claws without setae, simple. **Ventrites.** Microserration at sides (e.g. 100 points per mm) absent, ventrite 5 apex arcuate without paired setal brushes. **Aedeagus.** Parameres with articulation at base, apical lateral expansions absent; each paramere with three or more setae. **Geography.** Known from Ontario, California, Texas, Michigan+ (MN, WI, MI), Indiana+ (IL, IN, OH), Arkansas+ (KS, MO, OK, AR, LA, MS), Massachusetts+ (MA, CT, RI), New York, Pennsylvania+ (PA, WV), Virginia+ (NJ, DE, MD, DC, VA), Carolinas+ (KY, TN, NC, SC, GA, AL) and Florida ['+' refers to several states or provinces collectively].

3.6 SERICUS Eschscholtz, 1829

Habitus. Body Length 5-10 mm, 10-15 mm or 15-20 mm. Vestiture. Scale like setae absent. **Head.** Supra-antennal carinae fading on frons (not reaching another structure) or directed anteriorly (reaching anterior part of head capsule); hypognathous (labrum oriented downward 90 degrees or more); frons without triangular depression; gena broadened anteriorly below eye, extending spine-like anterior to basal tubercle of mandible (ventral condyle). Antennae with 11 antennomeres, not pectinate, sensory elements beginning on antennomere IV. **Prothorax.** Pronotum wider than long at widest point (including hind angles); dorsal punctures uniform sized, all simple (floor of puncture concave) or some or all punctures umbilicate (floor of puncture flat), without tubercles or longitudinal carinae between punctures; pronotal lateral carina complete anteriorly, carina visible throughout length in dorsal view or carina not visible anteriorly in dorsal view, meeting anterior edge of prothorax at about 90 degrees in lateral view, not serrate; bioluminescent spots absent; hind angle carinae present (single); posterior edge of pronotum with sublateral plicae and notches absent, crenellations absent; hypomeron posterior edge near each hind angle with or without concavity, concavity arcuate; pronotosternal sutures open or closed, hypomeral beads present. Prosternum with sides concave at midlength in ventral view; prosternal process not curved upward more than 40 degrees in lateral view. **Mesothorax.** Mesocoxal cavity open; mesoventral cavity without serration along sides. Elytra. Striae present; anterior edge outline straight to arcuate near humeri in dorsal view; integument unmarked with spots or tranverse bands; without pattern from differences in setal colour; setal vestiture even and mainly parallel. **Legs.** Metacoxal plate without elongation in mesal half, plate reaching lateral edge; tarsal pads or membranous lobes absent; tarsal claws without setae, simple. **Ventrites.** Microserration at sides (e.g. 100 points per mm) absent, ventrite 5 apex arcuate without paired setal brushes. **Aedeagus.** Parameres with articulation at base, apical lateral expansions absent; each paramere with three or more setae. **Geography.** Known from Nunavut+ (NT, NU), Yukon Territory, British Columbia, Alberta, Manitoba+ (MB, SK), Ontario, Quebec, New Brunswick+ (NB, NS, PE), Newfoundland and Labrador, Alaska, Oregon+ (WA, OR, ID), Montana+ (MT, WY, ND), Nebraska+ (SD, NE, IA), Michigan+ (MN, WI, MI), Indiana+ (IL, IN, OH), Vermont+ (ME, VT, NH), Massachusetts+ (MA, CT, RI), New York, Pennsylvania+ (PA, WV), Virginia+ (NJ, DE, MD, DC, VA) and Carolinas+ (KY, TN, NC, SC, GA, AL) ['+' refers to several states or provinces collectively].

3.7 IDOLUS Desbrochers 1875

Habitus. Body Length 1-5 mm or 5-10 mm. Vestiture. Scale like setae absent. **Head.** Supra-antennal carinae joining medially (forming shelf) or fading on frons (not reaching another structure); nasale (head

capsule below edge of frontal carina) with outline not concave in lateral view; hypognathous (labrum oriented downward 90 degrees or more); frons without triangular depression; gena broadened anteriorly below eye, extending spine-like anterior to basal tubercle of mandible (ventral condyle). Antennae with 11 antennomeres, not pectinate, sensory elements beginning on antennomere IV. **Prothorax.** Pronotum wider than long or longer than wide at widest point (including hind angles); dorsal punctures uniform sized, some or all punctures umbilicate (floor of puncture flat), without tubercles or longitudinal carinae between punctures; pronotal lateral carina complete anteriorly, carina not visible anteriorly in dorsal view, meeting mesal edge of hypomeron at about 30 degrees in lateral view, not serrate; bioluminescent spots absent; hind angle carinae present (single); posterior edge of pronotum with sublateral plicae and notches absent, crenellations absent; hypomeron posterior edge near each hind angle with concavity, concavity arcuate; pronotosternal sutures open, hypomeral beads present. Prosternum with sides concave at midlength in ventral view; prosternal process not curved upward more than 40 degrees in lateral view. **Mesothorax.** Mesocoxal cavity open or open to mesepimeron only; mesoventral cavity with serration along sides. Elytra. Striae present; anterior edge outline straight to arcuate near humeri in dorsal view; integument marked with spots or transverse bands or unmarked with spots or tranverse bands; without pattern from differences in setal colour; setal vestiture even and mainly parallel. **Legs.** Metacoxal plate with elongation in mesal half, plate reaching lateral edge; tarsal pads or membranous lobes absent; tarsal claws without setae, simple. **Ventrites.** Microserration at sides (e.g. 100 points per mm) absent, ventrite 5 apex arcuate without paired setal brushes. **Aedeagus.** Parameres with articulation at base, apical lateral expansions present; each paramere with two setae. **Geography.** Known from British Columbia, Alberta, Manitoba+ (MB, SK), Ontario, Quebec, New Brunswick+ (NB, NS, PE), Oregon+ (WA, OR, ID), California, Montana+ (MT, WY, ND), Michigan+ (MN, WI, MI), Vermont+ (ME, VT, NH), Massachusetts+ (MA, CT, RI), New York and Pennsylvania+ (PA, WV) ['+' refers to several states or provinces collectively].

I. debilis (LeConte, 1884)

Habitus. Body Length 1-5 mm. Vestiture. Scale like setae absent. **Head.** Supra-antennal carinae joining medially (forming shelf); nasale (head capsule below edge of frontal carina) with outline concave in lateral view; frons without triangular depression; gena broadened anteriorly below eye, extending spine-like anterior to basal tubercle of mandible (ventral condyle). Antennae with 11 antennomeres, not pectinate, sensory elements beginning on antennomere IV. **Prothorax.** Pronotum wider than long or longer than wide at widest point (including hind angles); dorsal punctures uniform sized, all simple (floor of puncture concave), without tubercles or longitudinal carinae between punctures; pronotal lateral carina complete anteriorly, carina visible throughout length in dorsal view or carina not visible anteriorly in dorsal view, meeting anterior edge of prothorax at about 90 degrees in lateral view, not serrate; bioluminescent spots absent; hind angle carinae present (single); posterior edge of pronotum with sublateral plicae and notches absent, crenellations absent; hypomeron posterior edge near each hind angle with concavity, concavity arcuate or angulate; pronotosternal sutures closed, hypomeral beads present. Prosternum with sides straight at midlength in ventral view; prosternal process not curved upward more than 40 degrees in lateral view. **Mesothorax.** Mesocoxal cavity open; mesoventral cavity without serration along sides. Elytra. Striae present; anterior edge outline straight to arcuate near humeri in dorsal view; integument marked with spots or transverse bands, marked with spot or band in apical 2/5 only or unmarked with spots or tranverse bands; without pattern from differences in setal colour; setal vestiture even and mainly parallel. **Legs.** Metacoxal plate with elongation in mesal half, plate reaching lateral edge; tarsal pads or membranous lobes absent; tarsal claws without setae, simple. **Ventrites.** Microserration at sides (e.g. 100 points per mm) absent, ventrite 5 apex arcuate without paired setal brushes. **Aedeagus.** Parameres with articulation at base, apical lateral expansions

absent; parameres without setae. **Geography.** Known from Yukon Territory, British Columbia, Alberta, Manitoba+ (MB, SK), Ontario, Quebec, New Brunswick+ (NB, NS, PE), Newfoundland and Labrador, Alaska, Oregon+ (WA, OR, ID), Montana+ (MT, WY, ND), Colorado+ (NV, UT, CO), Nebraska+ (SD, NE, IA), Michigan+ (MN, WI, MI), Vermont+ (ME, VT, NH), Massachusetts+ (MA, CT, RI), New York and Carolinas+ (KY, TN, NC, SC, GA, AL) ['+' refers to several states or provinces collectively].

3.7 LEPTOSCHEMA Horn, 1885

Habitus. Body Length 10-15 mm or 15-20 mm. Vestiture. Scale like setae absent. **Head.** Supra-antennal carinae joining medially (forming shelf); nasale (head capsule below edge of frontal carina) with outline concave or not concave in lateral view; prognathous (labrum oriented less than 90 degrees downward); frons without triangular depression; gena broadened anteriorly below eye, extending spine-like anterior to basal tubercle of mandible (ventral condyle). Antennae with 11 antennomeres, not pectinate, sensory elements beginning on antennomere IV. **Prothorax.** Pronotum longer than wide at widest point (including hind angles); dorsal punctures uniform sized, some or all punctures umbilicate (floor of puncture flat), without tubercles or longitudinal carinae between punctures; pronotal lateral carina complete anteriorly, carina not visible anteriorly in dorsal view, meeting anterior edge of prothorax at about 90 degrees in lateral view, not serrate; bioluminescent spots absent; hind angle carinae present (single); posterior edge of pronotum with sublateral plicae and notches absent, crenellations absent; hypomeron posterior edge near each hind angle with or without concavity, concavity arcuate; pronotosternal sutures open, hypomeral beads present. Prosternum with sides concave at midlength in ventral view; prosternal process not curved upward more than 40 degrees in lateral view. **Mesothorax.** Mesocoxal cavity open; mesoventral cavity with or without serration along sides. Elytra. Striae present; anterior edge outline straight to arcuate near humeri in dorsal view; integument unmarked with spots or tranverse bands; without pattern from differences in setal colour; setal vestiture even and mainly parallel. **Legs.** Metacoxal plate without elongation in mesal half, plate reaching lateral edge; tarsal pads or membranous lobes absent; tarsal claws without setae, simple. **Ventrites.** Microserration at sides (e.g. 100 points per mm) absent, ventrite 5 apex arcuate without paired setal brushes. **Aedeagus.** Parameres with articulation at base, apical lateral expansions present; each paramere with three or more setae. **Geography.** Known from California ['+' refers to several states or provinces collectively].

3.8 AGRIOTES Eschscholtz, 1829

Habitus. Body Length 1-5 mm, 5-10 mm or 10-15 mm. Vestiture. Scale like setae absent. **Head.** Supra-antennal carinae fading on frons (not reaching another structure) or directed anteriorly (reaching anterior part of head capsule); hypognathous (labrum oriented downward 90 degrees or more); frons without triangular depression; gena broadened anteriorly below eye, extending spine-like anterior to basal tubercle of mandible (ventral condyle). Antennae with 11 antennomeres, not pectinate, sensory elements beginning on antennomere IV. **Prothorax.** Pronotum wider than long or longer than wide at widest point (including hind angles); dorsal punctures uniform sized, all simple (floor of puncture concave) or some or all punctures umbilicate (floor of puncture flat), without tubercles or longitudinal carinae between punctures; pronotal lateral carina complete anteriorly or incomplete anteriorly, carina not visible anteriorly in dorsal view, meeting mesal edge of hypomeron at about 30 degrees in lateral view, not serrate; bioluminescent spots absent; hind angle carinae present (single); posterior edge of pronotum with sublateral plicae and notches present or absent, crenellations absent; hypomeron posterior edge near each hind angle with concavity, concavity arcuate; pronotosternal sutures open, hypomeral beads present. Prosternum with sides straight or concave at midlength in ventral view;

prosternal process not curved upward more than 40 degrees in lateral view. **Mesothorax.** Mesocoxal cavity open; mesoventral cavity with or without serration along sides. Elytra. Striae present; anterior edge outline straight to arcuate near humeri in dorsal view; integument unmarked with spots or tranverse bands; without pattern from differences in setal colour; setal vestiture even and mainly parallel, partially transverse in patches or colour pattern from differences in setal colour. **Legs.** Metacoxal plate with or without elongation in mesal half, plate reaching or not reaching lateral edge; tarsal pads or membranous lobes absent; tarsal claws without setae, simple. **Ventrites.** Microserration at sides (e.g. 100 points per mm) absent, ventrite 5 apex arcuate without paired setal brushes. **Aedeagus.** Parameres with articulation at base, apical lateral expansions present; parameres without setae, each paramere with one seta, each paramere with two setae or each paramere with three or more setae. **Geography.** Known from Nunavut+ (NT, NU), Yukon Territory, British Columbia, Alberta, Manitoba+ (MB, SK), Ontario, Quebec, New Brunswick+ (NB, NS, PE), Newfoundland and Labrador, Oregon+ (WA, OR, ID), California, Montana+ (MT, WY, ND), Colorado+ (NV, UT, CO), Arizona+ (AZ, NM), Nebraska+ (SD, NE, IA), Texas, Michigan+ (MN, WI, MI), Indiana+ (IL, IN, OH), Arkansas+ (KS, MO, OK, AR, LA, MS), Vermont+ (ME, VT, NH), Massachusetts+ (MA, CT, RI), New York, Pennsylvania+ (PA, WV), Virginia+ (NJ, DE, MD, DC, VA) and Carolinas+ (KY, TN, NC, SC, GA, AL) ['+' refers to several states or provinces collectively].

3.8 DALOPIUS Eschscholtz, 1829

Habitus. Body Length 1-5 mm or 5-10 mm. Vestiture. Scale like setae absent. **Head.** Supra-antennal carinae fading on frons (not reaching another structure); nasale (head capsule below edge of frontal carina) with outline not concave in lateral view; hypognathous (labrum oriented downward 90 degrees or more), rarely prognathous (labrum oriented less than 90 degrees downward) (by misinterpretation); frons without triangular depression; gena broadened anteriorly below eye, extending spine-like anterior to basal tubercle of mandible (ventral condyle). Antennae with 11 antennomeres, not pectinate, sensory elements beginning on antennomere IV. **Prothorax.** Pronotum wider than long or longer than wide at widest point (including hind angles); dorsal punctures uniform sized, some or all punctures umbilicate (floor of puncture flat), without tubercles or longitudinal carinae between punctures; pronotal lateral carina complete anteriorly, carina visible throughout length in dorsal view, meeting anterior edge of prothorax at about 90 degrees in lateral view, not serrate; bioluminescent spots absent; hind angle carinae present (single); posterior edge of pronotum with sublateral plicae and notches absent, crenellations absent; hypomeron posterior edge near each hind angle with concavity, concavity arcuate or angulate; pronotosternal sutures open, hypomeral beads present. Prosternum with sides concave at midlength in ventral view; prosternal process not curved upward more than 40 degrees in lateral view. **Mesothorax.** Mesocoxal cavity open; mesoventral cavity with serration along sides. Elytra. Striae present; anterior edge outline straight to arcuate near humeri in dorsal view; integument unmarked with spots or tranverse bands; without pattern from differences in setal colour; setal vestiture even and mainly parallel. **Legs.** Metacoxal plate with or without elongation in mesal half, plate reaching lateral edge; tarsal pads or membranous lobes absent; tarsal claws without setae, simple. **Ventrites.** Microserration at sides (e.g. 100 points per mm) absent, ventrite 5 apex arcuate without paired setal brushes. **Aedeagus.** Parameres with articulation at base, apical lateral expansions present; each paramere with one seta, each paramere with two setae or each paramere with three or more setae. **Geography.** Known from Yukon Territory, British Columbia, Alberta, Manitoba+ (MB, SK), Ontario, Quebec, New Brunswick+ (NB, NS, PE), Newfoundland and Labrador, Alaska, Oregon+ (WA, OR, ID), California, Montana+ (MT, WY, ND), Colorado+ (NV, UT, CO), Arizona+ (AZ, NM), Nebraska+ (SD, NE, IA), Michigan+ (MN, WI, MI), Indiana+ (IL, IN, OH), New York, Pennsylvania+ (PA, WV) and Carolinas+ (KY, TN, NC, SC, GA, AL) ['+' refers to several states or provinces collectively].

D. inordinatus Brown, 1934

Habitus. Body Length 1-5 mm. Vestiture. Scale like setae absent. **Head.** Supra-antennal carinae fading on frons (not reaching another structure); hypognathous (labrum oriented downward 90 degrees or more); frons without triangular depression; gena broadened anteriorly below eye, extending spine-like anterior to basal tubercle of mandible (ventral condyle). Antennae with 11 antennomeres, not pectinate, sensory elements beginning on antennomere IV. **Prothorax.** Pronotum wider than long at widest point (including hind angles); dorsal punctures uniform sized, some or all punctures umbilicate (floor of puncture flat), without tubercles or longitudinal carinae between punctures; pronotal lateral carina complete anteriorly, carina visible throughout length in dorsal view or carina not visible anteriorly in dorsal view, meeting anterior edge of prothorax at about 90 degrees in lateral view, not serrate; bioluminescent spots absent; hind angle carinae present (single); posterior edge of pronotum with sublateral plicae and notches absent, crenellations absent; hypomeron posterior edge near each hind angle with concavity, concavity arcuate; pronotosternal sutures open, hypomeral beads present. Prosternum with sides concave at midlength in ventral view; prosternal process not curved upward more than 40 degrees in lateral view. **Mesothorax.** Mesocoxal cavity open; mesoventral cavity without serration along sides. Elytra. Striae present; anterior edge outline straight to arcuate near humeri in dorsal view; integument unmarked with spots or tranverse bands; without pattern from differences in setal colour; setal vestiture even and mainly parallel. **Legs.** Metacoxal plate with or without elongation in mesal half, plate reaching lateral edge; tarsal pads or membranous lobes absent; tarsal claws without setae, simple. **Ventrites.** Microserration at sides (e.g. 100 points per mm) absent, ventrite 5 apex arcuate without paired setal brushes. **Aedeagus.** Parameres with articulation at base, apical lateral expansions present; each paramere with two setae or each paramere with three or more setae. **Geography.** Known from British Columbia, Alberta, Manitoba+ (MB, SK), California, Colorado+ (NV, UT, CO) and Arizona+ (AZ, NM) ['+' refers to several states or provinces collectively].

3.9 GLYPHONYX Candèze, 1863

Habitus. Body Length 1-5 mm or 5-10 mm. Vestiture. Scale like setae absent. **Head.** Supra-antennal carinae joining medially (forming shelf) or directed anteriorly (reaching anterior part of head capsule); nasale (head capsule below edge of frontal carina) with outline not concave in lateral view; hypognathous (labrum oriented downward 90 degrees or more); frons without triangular depression; gena broadened anteriorly below eye, extending spine-like anterior to basal tubercle of mandible (ventral condyle). Antennae with 11 antennomeres, not pectinate, sensory elements beginning on antennomere IV. **Prothorax.** Pronotum wider than long at widest point (including hind angles); dorsal punctures uniform sized, all simple (floor of puncture concave), without tubercles or longitudinal carinae between punctures; pronotal lateral carina complete anteriorly, carina not visible anteriorly in dorsal view, meeting mesal edge of hypomeron at about 30 degrees in lateral view, not serrate; bioluminescent spots absent; hind angle carinae present (single); posterior edge of pronotum with sublateral plicae and notches present, crenellations absent; hypomeron posterior edge near each hind angle with concavity, concavity arcuate; pronotosternal sutures open, hypomeral beads present or absent. Prosternum with sides straight or concave at midlength in ventral view; prosternal process not curved upward more than 40 degrees in lateral view. **Mesothorax.** Mesocoxal cavity open or open to mesepimeron only; mesoventral cavity with serration along sides. Elytra. Striae present; anterior edge outline straight to arcuate near humeri in dorsal view; integument unmarked with spots or tranverse bands; without pattern from differences in setal colour; setal vestiture even and mainly parallel. **Legs.** Metacoxal plate without elongation in mesal half, plate reaching lateral edge; tarsal pads or membranous lobes present on tarsomere IV only; tarsal claws without setae, with two points, or

appendiculate or with 3 or more points. **Ventrites.** Microserration at sides (e.g. 100 points per mm) absent, ventrite 5 apex arcuate without paired setal brushes. **Aedeagus.** Parameres with articulation at base, apical lateral expansions absent; each paramere with one seta or each paramere with two setae. **Geography.** Known from Ontario, Oregon+ (WA, OR, ID), California, Montana+ (MT, WY, ND), Colorado+ (NV, UT, CO), Arizona+ (AZ, NM), Nebraska+ (SD, NE, IA), Texas, Indiana+ (IL, IN, OH), Arkansas+ (KS, MO, OK, AR, LA, MS), Vermont+ (ME, VT, NH), Massachusetts+ (MA, CT, RI), New York, Pennsylvania+ (PA, WV), Virginia+ (NJ, DE, MD, DC, VA), Carolinas+ (KY, TN, NC, SC, GA, AL) and Florida ['+' refers to several states or provinces collectively].

4 PITYOBIUS LeConte, 1853

Habitus. Body Length 20-40 mm. Vestiture. Scale like setae absent. **Head.** Supra-antennal carinae joining medially (forming shelf); nasale (head capsule below edge of frontal carina) with outline concave in lateral view; prognathous (labrum oriented less than 90 degrees downward); frons with triangular depression. Antennae with 11 or 12 antennomeres, pectinate or bipectinate or not pectinate, sensory elements beginning on antennomere IV. **Prothorax.** Pronotum longer than wide at widest point (including hind angles); dorsal punctures uniform sized, all simple (floor of puncture concave), without tubercles or longitudinal carinae between punctures; pronotal lateral carina complete anteriorly, carina visible throughout length in dorsal view or carina not visible anteriorly in dorsal view, meeting anterior edge of prothorax at about 90 degrees in lateral view, not serrate; bioluminescent spots absent; hind angle carinae present (single); posterior edge of pronotum with sublateral plicae and notches absent, crenellations absent; hypomeron posterior edge near each hind angle with concavity, concavity arcuate; pronotosternal sutures closed, hypomeral beads present. Prosternum with sides straight or concave at midlength in ventral view; prosternal process curved or not curved upward more than 40 degrees in lateral view. **Mesothorax.** Mesocoxal cavity open; mesoventral cavity without serration along sides. Elytra. Striae present; anterior edge outline straight to arcuate near humeri in dorsal view; integument unmarked with spots or tranverse bands; without pattern from differences in setal colour; setal vestiture even and mainly parallel. **Legs.** Metacoxal plate without elongation in mesal half, plate reaching lateral edge; tarsal pads or membranous lobes present on multiple tarsomeres, (I, II, III, and IV or II, III, and IV); tarsal claws without setae, simple. **Ventrites.** Microserration at sides (e.g. 100 points per mm) absent, ventrite 5 apex arcuate without paired setal brushes. **Aedeagus.** Parameres with articulation at base, apical lateral expansions present; parameres without setae. **Geography.** Known from Alberta, Manitoba+ (MB, SK), Ontario, Quebec, New Brunswick+ (NB, NS, PE), Oregon+ (WA, OR, ID), California, Michigan+ (MN, WI, MI), Vermont+ (ME, VT, NH), Pennsylvania+ (PA, WV), Carolinas+ (KY, TN, NC, SC, GA, AL) and Florida ['+' refers to several states or provinces collectively].

5.1 ANTHRACALAUS Fairmaire, 1888

Habitus. Body Length 20-40 mm. Vestiture. Scale like setae absent. **Head.** Supra-antennal carinae fading on frons (not reaching another structure) or directed anteriorly (reaching anterior part of head capsule); prognathous (labrum oriented less than 90 degrees downward); frons without triangular depression. Antennae with 11 antennomeres, not pectinate, sensory elements beginning on antennomere IV. **Prothorax.** Pronotum wider than long or longer than wide at widest point (including hind angles); dorsal punctures uniform sized, all simple (floor of puncture concave), without tubercles or longitudinal carinae between punctures; pronotal lateral carina complete anteriorly, carina visible throughout length in dorsal view, meeting anterior edge of prothorax at about 90 degrees in lateral view, not serrate; bioluminescent spots absent; hind angle carinae present (single); posterior edge of pronotum with sublateral plicae and notches absent, crenellations absent; hypomeron posterior edge near each hind

angle with concavity, concavity arcuate or angulate; pronotosternal sutures open, hypomeral beads present. Prosternum with sides straight at midlength in ventral view; prosternal process not curved upward more than 40 degrees in lateral view. **Mesothorax.** Mesocoxal cavity open; mesoventral cavity without serration along sides. Elytra. Striae present; anterior edge outline straight to arcuate near humeri in dorsal view; integument unmarked with spots or tranverse bands; without pattern from differences in setal colour; setal vestiture mainly absent on disk or even and mainly parallel. **Legs.** Metacoxal plate without elongation in mesal half, plate reaching lateral edge; profemur without carina across full length of anterior face (basodorsal to apicoventral); tarsal pads or membranous lobes absent; tarsal claws with setae, simple. **Ventrites.** Microserration at sides (e.g. 100 points per mm) absent, ventrite 5 apex arcuate without paired setal brushes. **Aedeagus.** Parameres with articulation at base, apical lateral expansions present; each paramere with three or more setae. **Geography.** Known from Arizona+ (AZ, NM) ['+' refers to several states or provinces collectively].

5.1 LANELATER Arnett, 1952

Habitus. Body Length 10-15 mm, 15-20 mm or 20-40 mm. Vestiture. Scale like setae absent. **Head.** Supra-antennal carinae joining medially (forming shelf), fading on frons (not reaching another structure) or directed anteriorly (reaching anterior part of head capsule); nasale (head capsule below edge of frontal carina) with outline concave or not concave in lateral view; prognathous (labrum oriented less than 90 degrees downward); frons without triangular depression. Antennae with 11 antennomeres, not pectinate, sensory elements beginning on antennomere IV. **Prothorax.** Pronotum longer than wide at widest point (including hind angles); dorsal punctures uniform sized, all simple (floor of puncture concave), without tubercles or longitudinal carinae between punctures; pronotal lateral carina complete anteriorly, carina visible throughout length in dorsal view, meeting anterior edge of prothorax at about 90 degrees in lateral view, not serrate; bioluminescent spots absent; hind angle carinae present (single); posterior edge of pronotum with sublateral plicae and notches absent, crenellations absent; hypomeron posterior edge near each hind angle without concavity, concavity arcuate; pronotosternal sutures excavated (able to contain antennae), hypomeral beads present. Prosternum with sides straight at midlength in ventral view; prosternal process not curved upward more than 40 degrees in lateral view. **Mesothorax.** Mesocoxal cavity open; mesoventral cavity without serration along sides. Elytra. Striae present; anterior edge outline straight to arcuate near humeri in dorsal view; integument unmarked with spots or tranverse bands; without pattern from differences in setal colour; setal vestiture even and mainly parallel. **Legs.** Metacoxal plate without elongation in mesal half, plate reaching or not reaching lateral edge; profemur without carina across full length of anterior face (basodorsal to apicoventral); tarsal pads or membranous lobes absent; tarsal claws with setae, simple. **Ventrites.** Microserration at sides (e.g. 100 points per mm) absent, ventrite 5 apex arcuate without paired setal brushes. **Aedeagus.** Parameres with articulation at base, apical lateral expansions present; each paramere with three or more setae. **Geography.** Known from Arizona+ (AZ, NM), Texas, Arkansas+ (KS, MO, OK, AR, LA, MS), New York, Virginia+ (NJ, DE, MD, DC, VA), Carolinas+ (KY, TN, NC, SC, GA, AL) and Florida ['+' refers to several states or provinces collectively].

5.2 AGRYPNUS Eschscholtz, 1829

Habitus. Body Length 5-10 mm or 10-15 mm. Vestiture. Scale like setae present. **Head.** Supra-antennal carinae fading on frons (not reaching another structure); hypognathous (labrum oriented downward 90 degrees or more); frons with or without triangular depression. Antennae with 11 antennomeres, not pectinate. **Prothorax.** Pronotum wider than long or longer than wide at widest point (including hind angles); dorsal punctures uniform sized, all simple (floor of puncture concave), without tubercles or

longitudinal carinae between punctures; pronotal lateral carina complete anteriorly, carina visible throughout length in dorsal view, meeting anterior edge of prothorax at about 90 degrees in lateral view, not serrate; bioluminescent spots absent; hind angle carinae absent; posterior edge of pronotum with sublateral plicae and notches absent, crenellations absent; hypomeron posterior edge near each hind angle with or without concavity, concavity angulate; pronotosternal sutures excavated (able to contain antennae), hypomeral beads absent. Prosternum with sides straight at midlength in ventral view; prosternal process curved or not curved upward more than 40 degrees in lateral view. **Mesothorax.** Mesocoxal cavity closed; mesoventral cavity without serration along sides. Elytra. Striae present; anterior edge outline sinuate (recurved) or with rectangular projection near humeri in dorsal view; integument unmarked with spots or tranverse bands; without pattern from differences in setal colour; setal vestiture even and mainly parallel. **Legs.** Metacoxal plate without elongation in mesal half, plate reaching lateral edge; profemur without carina across full length of anterior face (basodorsal to apicoventral); tarsal pads or membranous lobes absent; tarsal claws with setae, simple. **Ventrites.** Microserration at sides (e.g. 100 points per mm) absent, ventrite 5 apex arcuate without paired setal brushes. **Aedeagus.** Parameres with articulation at base, apical lateral expansions absent; each paramere with three or more setae. **Geography.** Known from Arizona+ (AZ, NM), Nebraska+ (SD, NE, IA), Texas, Indiana+ (IL, IN, OH), Arkansas+ (KS, MO, OK, AR, LA, MS), Carolinas+ (KY, TN, NC, SC, GA, AL) and Florida ['+' refers to several states or provinces collectively].

5.2 DANOSOMA Thompson, 1859

Habitus. Body Length 10-15 mm or 15-20 mm. Vestiture. Scale like setae present. **Head.** Supra-antennal carinae fading on frons (not reaching another structure) or directed anteriorly (reaching anterior part of head capsule); hypognathous (labrum oriented downward 90 degrees or more) or prognathous (labrum oriented less than 90 degrees downward); frons with or without triangular depression. Antennae with 11 antennomeres, not pectinate, sensory elements beginning on antennomere III or IV. **Prothorax.** Pronotum wider than long or longer than wide at widest point (including hind angles); dorsal punctures uniform sized, all simple (floor of puncture concave), without tubercles or longitudinal carinae between punctures; pronotal lateral carina complete anteriorly, carina visible throughout length in dorsal view, meeting anterior edge of prothorax at about 90 degrees in lateral view, not serrate; bioluminescent spots absent; hind angle carinae absent; posterior edge of pronotum with sublateral plicae and notches absent, crenellations absent; hypomeron posterior edge near each hind angle with or without concavity, concavity angulate; pronotosternal sutures excavated (able to contain antennae), hypomeral beads absent. Prosternum with sides convex at midlength in ventral view; prosternal process not curved upward more than 40 degrees in lateral view. **Mesothorax.** Mesocoxal cavity open or open to mesepimeron only; mesoventral cavity without serration along sides. Elytra. Striae absent; anterior edge outline straight to arcuate near humeri in dorsal view; integument unmarked with spots or tranverse bands; with pattern from differences in setal colour; setal vestiture even and mainly parallel. **Legs.** Metacoxal plate with or without elongation in mesal half, plate reaching lateral edge; profemur without carina across full length of anterior face (basodorsal to apicoventral); tarsal pads or membranous lobes absent; tarsal claws without setae, simple. **Ventrites.** Microserration at sides (e.g. 100 points per mm) absent, ventrite 5 apex arcuate without paired setal brushes. **Aedeagus.** Parameres with articulation at base, apical lateral expansions present; each paramere with three or more setae. **Geography.** Known from Nunavut+ (NT, NU), Yukon Territory, British Columbia, Alberta, Manitoba+ (MB, SK), Ontario, Quebec, New Brunswick+ (NB, NS, PE), Alaska, Oregon+ (WA, OR, ID), California, Montana+ (MT, WY, ND), Colorado+ (NV, UT, CO), Nebraska+ (SD, NE, IA), Michigan+ (MN, WI, MI), Vermont+ (ME, VT, NH), Massachusetts+ (MA, CT, RI) and Pennsylvania+ (PA, WV) ['+' refers to several states or provinces collectively].

5.2 LACON Laporte, 1838

Habitus. Body Length 5-10 mm, 10-15 mm, 15-20 mm or 20-40 mm. Vestiture. Scale like setae present. **Head.** Supra-antennal carinae fading on frons (not reaching another structure) or directed anteriorly (reaching anterior part of head capsule); hypognathous (labrum oriented downward 90 degrees or more) or prognathous (labrum oriented less than 90 degrees downward); frons with or without triangular depression. Antennae with 11 antennomeres, not pectinate, sensory elements beginning on antennomere III. **Prothorax.** Pronotum wider than long or longer than wide at widest point (including hind angles); dorsal punctures uniform sized, all simple (floor of puncture concave), without tubercles or longitudinal carinae between punctures; pronotal lateral carina complete anteriorly, carina visible throughout length in dorsal view or carina not visible anteriorly in dorsal view, meeting anterior edge of prothorax at about 90 degrees in lateral view, not serrate; bioluminescent spots absent; hind angle carinae present (single) or absent; posterior edge of pronotum with sublateral plicae and notches absent, crenellations absent; hypomeron posterior edge near each hind angle with or without concavity, concavity arcuate; pronotosternal sutures excavated (able to contain antennae), hypomeral beads absent. Prosternum with sides straight or convex at midlength in ventral view; prosternal process not curved upward more than 40 degrees in lateral view. **Mesothorax.** Mesocoxal cavity open or open to mesepimeron only; mesoventral cavity without serration along sides. Elytra. Striae present or absent; anterior edge outline straight to arcuate near humeri in dorsal view; integument unmarked with spots or tranverse bands; with or without pattern from differences in setal colour; setal vestiture even and mainly parallel. **Legs.** Metacoxal plate without elongation in mesal half, plate reaching lateral edge; profemur without carina across full length of anterior face (basodorsal to apicoventral); tarsal pads or membranous lobes present on tarsomere IV only or absent; tarsal claws with setae, simple. **Ventrites.** Microserration at sides (e.g. 100 points per mm) absent, ventrite 5 apex arcuate without paired setal brushes. **Aedeagus.** Parameres with articulation at base, apical lateral expansions present; each paramere with three or more setae. **Geography.** Known from British Columbia, Alberta, Manitoba+ (MB, SK), Ontario, Quebec, New Brunswick+ (NB, NS, PE), Oregon+ (WA, OR, ID), California, Montana+ (MT, WY, ND), Colorado+ (NV, UT, CO), Arizona+ (AZ, NM), Nebraska+ (SD, NE, IA), Texas, Michigan+ (MN, WI, MI), Indiana+ (IL, IN, OH), Arkansas+ (KS, MO, OK, AR, LA, MS), Vermont+ (ME, VT, NH), Massachusetts+ (MA, CT, RI), New York, Pennsylvania+ (PA, WV), Virginia+ (NJ, DE, MD, DC, VA), Carolinas+ (KY, TN, NC, SC, GA, AL) and Florida ['+' refers to several states or provinces collectively].

5.2 MERISTHUS Candèze, 1857

Habitus. Body Length 1-5 mm. Vestiture. Scale like setae present. **Head.** Supra-antennal carinae directed anteriorly (reaching anterior part of head capsule); hypognathous (labrum oriented downward 90 degrees or more); frons without triangular depression. Antennae with 11 antennomeres, not pectinate, sensory elements beginning on antennomere IV. **Prothorax.** Pronotum wider than long or longer than wide at widest point (including hind angles); dorsal punctures uniform sized, with tubercles or longitudinal carinae between punctures; pronotal lateral carina incomplete anteriorly, carina visible throughout length in dorsal view, microserrate along entire side (e.g. about 70 points per mm) or not serrate; bioluminescent spots absent; hind angle carinae absent; posterior edge of pronotum with sublateral plicae and notches absent, crenellations absent; hypomeron posterior edge near each hind angle with or without concavity, concavity angulate; pronotosternal sutures excavated (able to contain antennae), hypomeral beads absent. Prosternum with sides straight or concave at midlength in ventral view; prosternal process not curved upward more than 40 degrees in lateral view. **Mesothorax.** Mesocoxal cavity closed; mesoventral cavity without serration along sides. Elytra. Striae present or absent; anterior edge outline sinuate (recurved) or with rectangular projection near

humeri in dorsal view; integument unmarked with spots or tranverse bands; without pattern from differences in setal colour; setal vestiture even and mainly parallel. **Legs.** Metacoxal plate with or without elongation in mesal half, plate reaching lateral edge; profemur without carina across full length of anterior face (basodorsal to apicoventral); tarsal pads or membranous lobes absent; tarsal claws with setae, simple. **Ventrites.** Microserration at sides (e.g. 100 points per mm) absent, ventrite 5 apex arcuate without paired setal brushes. **Aedeagus.** Parameres with articulation at base, apical lateral expansions present; each paramere with three or more setae. **Geography.** Known from Arizona+ (AZ, NM) and Texas ['+' refers to several states or provinces collectively].

5.2 RISMETHUS Fleutiaux, 1947

Habitus. Body Length 1-5 mm. Vestiture. Scale like setae present. **Head.** Supra-antennal carinae directed anteriorly (reaching anterior part of head capsule); hypognathous (labrum oriented downward 90 degrees or more) or prognathous (labrum oriented less than 90 degrees downward); frons without triangular depression. Antennae with 11 antennomeres, not pectinate, sensory elements beginning on antennomere IV. **Prothorax.** Pronotum wider than long at widest point (including hind angles); dorsal punctures of two distinct intermixed sizes (heterogeneous), all simple (floor of puncture concave), without tubercles or longitudinal carinae between punctures; pronotal lateral carina complete anteriorly (by misinterpretation) or incomplete anteriorly, carina visible throughout length in dorsal view, meeting anterior edge of prothorax at about 90 degrees in lateral view, not serrate; bioluminescent spots absent; hind angle carinae absent; posterior edge of pronotum with sublateral plicae and notches absent, crenellations absent; hypomeron posterior edge near each hind angle with or without concavity, concavity angulate; pronotosternal sutures excavated (able to contain antennae), hypomeral beads absent. Prosternum with sides straight or concave at midlength in ventral view; prosternal process not curved upward more than 40 degrees in lateral view. **Mesothorax.** Mesocoxal cavity closed; mesoventral cavity without serration along sides. Elytra. Striae present; anterior edge outline sinuate (recurved) or with rectangular projection near humeri in dorsal view; integument unmarked with spots or tranverse bands; without pattern from differences in setal colour; setal vestiture even and mainly parallel. **Legs.** Metacoxal plate with or without elongation in mesal half, plate reaching lateral edge; profemur without carina across full length of anterior face (basodorsal to apicoventral); tarsal pads or membranous lobes absent, simple. **Ventrites.** Microserration at sides (e.g. 100 points per mm) absent, ventrite 5 apex arcuate without paired setal brushes. **Aedeagus.** Parameres with articulation at base, apical lateral expansions present; each paramere with three or more setae. **Geography.** Known from Arizona+ (AZ, NM), Texas, Carolinas+ (KY, TN, NC, SC, GA, AL) and Florida ['+' refers to several states or provinces collectively].

5.3 ALAUS Eschscholtz, 1829

Habitus. Body Length 15-20 mm or 20-40 mm. Vestiture. Scale like setae present. **Head.** Supra-antennal carinae joining medially (forming shelf), fading on frons (not reaching another structure) or directed anteriorly (reaching anterior part of head capsule); nasale (head capsule below edge of frontal carina) with outline not concave in lateral view; prognathous (labrum oriented less than 90 degrees downward); frons with or without triangular depression. Antennae with 11 antennomeres, not pectinate, sensory elements beginning on antennomere III or IV. **Prothorax.** Pronotum longer than wide at widest point (including hind angles); dorsal punctures uniform sized, all simple (floor of puncture concave), without tubercles or longitudinal carinae between punctures; pronotal lateral carina complete anteriorly, carina visible throughout length in dorsal view, meeting anterior edge of prothorax at about 90 degrees in lateral view, not serrate; bioluminescent spots absent; hind angle carinae present (single) or absent;

posterior edge of pronotum with sublateral plicae and notches absent, crenellations absent; hypomeron posterior edge near each hind angle with concavity, concavity arcuate or angulate; pronotosternal sutures closed, hypomeral beads present or absent. Prosternum with sides straight or concave at midlength in ventral view; prosternal process not curved upward more than 40 degrees in lateral view. **Mesothorax.** Mesocoxal cavity open; mesoventral cavity without serration along sides. Elytra. Striae present; anterior edge outline straight to arcuate near humeri in dorsal view; integument unmarked with spots or tranverse bands; with pattern from differences in setal colour; setal vestiture even and mainly parallel or with bare patches. **Legs.** Metacoxal plate without elongation in mesal half, plate reaching lateral edge; profemur without carina across full length of anterior face (basodorsal to apicoventral); tarsal pads or membranous lobes absent; tarsal claws with setae, simple. **Ventrites.** Microserration at sides (e.g. 100 points per mm) absent, ventrite 5 apex arcuate without paired setal brushes. **Aedeagus.** Parameres with articulation at base, apical lateral expansions present; each paramere with three or more setae. **Geography.** Known from British Columbia, Manitoba+ (MB, SK), Ontario, Quebec, Oregon+ (WA, OR, ID), California, Montana+ (MT, WY, ND), Colorado+ (NV, UT, CO), Arizona+ (AZ, NM), Nebraska+ (SD, NE, IA), Texas, Michigan+ (MN, WI, MI), Indiana+ (IL, IN, OH), Arkansas+ (KS, MO, OK, AR, LA, MS), Vermont+ (ME, VT, NH), Massachusetts+ (MA, CT, RI), New York, Pennsylvania+ (PA, WV), Virginia+ (NJ, DE, MD, DC, VA), Carolinas+ (KY, TN, NC, SC, GA, AL) and Florida ['+' refers to several states or provinces collectively].

5.3 CHALCOLEPIDIUS Eschscholtz, 1829

Habitus. Body Length 15-20 mm or 20-40 mm. Vestiture. Scale like setae present. **Head.** Supra-antennal carinae absent; prognathous (labrum oriented less than 90 degrees downward); frons with or without triangular depression. Antennae with 11 antennomeres, pectinate or bipectinate or not pectinate, sensory elements beginning on antennomere IV. **Prothorax.** Pronotum longer than wide at widest point (including hind angles); dorsal punctures uniform sized, all simple (floor of puncture concave), without tubercles or longitudinal carinae between punctures; pronotal lateral carina complete anteriorly, carina visible throughout length in dorsal view, meeting anterior edge of prothorax at about 90 degrees in lateral view, not serrate; bioluminescent spots absent; hind angle carinae absent; posterior edge of pronotum with sublateral plicae and notches absent, crenellations absent; hypomeron posterior edge near each hind angle with concavity, concavity angulate; pronotosternal sutures open or closed, hypomeral beads absent. Prosternum with sides convex at midlength in ventral view; prosternal process not curved upward more than 40 degrees in lateral view. **Mesothorax.** Mesocoxal cavity open; mesoventral cavity without serration along sides. Elytra. Striae present; anterior edge outline sinuate (recurved) or with rectangular projection near humeri in dorsal view; integument with metallic reflections (by misinterpretation) or unmarked with spots or tranverse bands; with or without pattern from differences in setal colour; setal vestiture even and mainly parallel. **Legs.** Metacoxal plate without elongation in mesal half, plate reaching lateral edge; profemur without carina across full length of anterior face (basodorsal to apicoventral); tarsal pads or membranous lobes absent; tarsal claws with setae, simple. **Ventrites.** Microserration at sides (e.g. 100 points per mm) absent, ventrite 5 apex arcuate without paired setal brushes. **Aedeagus.** Parameres with articulation at midlength, fused at base, apical lateral expansions present or absent; each paramere with three or more setae. **Geography.** Known from California, Arizona+ (AZ, NM), Texas, Indiana+ (IL, IN, OH), Arkansas+ (KS, MO, OK, AR, LA, MS), Pennsylvania+ (PA, WV), Virginia+ (NJ, DE, MD, DC, VA), Carolinas+ (KY, TN, NC, SC, GA, AL) and Florida ['+' refers to several states or provinces collectively].

5.3 PHERHEMIUS Fleutiaux, 1942

Habitus. Body Length 15-20 mm. Vestiture. Scale like setae absent. **Head.** Supra-antennal carinae joining medially (forming shelf); nasale (head capsule below edge of frontal carina) with outline concave or not concave in lateral view; hypognathous (labrum oriented downward 90 degrees or more); frons with or without triangular depression. Antennae with 11 or 12 antennomeres, pectinate or bipectinate, sensory elements beginning on antennomere IV. **Prothorax.** Pronotum longer than wide at widest point (including hind angles); dorsal punctures uniform sized, some or all punctures umbilicate (floor of puncture flat), without tubercles or longitudinal carinae between punctures; pronotal lateral carina complete anteriorly, carina visible throughout length in dorsal view, meeting anterior edge of prothorax at about 90 degrees in lateral view, not serrate; bioluminescent spots absent; hind angle carinae present (two carinae); posterior edge of pronotum with sublateral plicae and notches absent, crenellations absent; hypomeron posterior edge near each hind angle with concavity, concavity arcuate; pronotosternal sutures open, hypomeral beads present. Prosternum with sides straight or convex at midlength in ventral view; prosternal process not curved upward more than 40 degrees in lateral view. **Mesothorax.** Mesocoxal cavity open; mesoventral cavity without serration along sides. Elytra. Striae present; anterior edge outline straight to arcuate near humeri in dorsal view; integument marked with spots or transverse bands; with pattern from differences in setal colour; setal vestiture even and mainly parallel. **Legs.** Metacoxal plate with or without elongation in mesal half, plate reaching lateral edge; profemur without carina across full length of anterior face (basodorsal to apicoventral); tarsal pads or membranous lobes absent; tarsal claws with setae, simple. **Ventrites.** Microserration at sides (e.g. 100 points per mm) absent, ventrite 5 apex arcuate without paired setal brushes. **Aedeagus.** Parameres with articulation at base, apical lateral expansions present; each paramere with three or more setae. **Geography.** Known from Texas, Arkansas+ (KS, MO, OK, AR, LA, MS), Pennsylvania+ (PA, WV), Virginia+ (NJ, DE, MD, DC, VA), Carolinas+ (KY, TN, NC, SC, GA, AL) and Florida ['+' refers to several states or provinces collectively].

5.4 DEILELATER Costa, 1975

Habitus. Body Length 10-15 mm or 15-20 mm. Vestiture. Scale like setae absent. **Head.** Supra-antennal carinae joining medially (forming shelf); nasale (head capsule below edge of frontal carina) with outline not concave in lateral view; prognathous (labrum oriented less than 90 degrees downward); frons without triangular depression. Antennae with 11 antennomeres, not pectinate, sensory elements beginning on antennomere IV. **Prothorax.** Pronotum longer than wide at widest point (including hind angles); dorsal punctures uniform sized, all simple (floor of puncture concave), without tubercles or longitudinal carinae between punctures; pronotal lateral carina complete anteriorly, carina visible throughout length in dorsal view, meeting anterior edge of prothorax at about 90 degrees in lateral view, not serrate; bioluminescent spots present; hind angle carinae present (single) or absent; posterior edge of pronotum with sublateral plicae and notches absent, crenellations absent; hypomeron posterior edge near each hind angle with concavity, concavity arcuate; pronotosternal sutures open or closed, hypomeral beads present. Prosternum with sides straight at midlength in ventral view; prosternal process not curved upward more than 40 degrees in lateral view. **Mesothorax.** Mesocoxal cavity open; mesoventral cavity without serration along sides. Elytra. Striae present; anterior edge outline straight to arcuate near humeri in dorsal view; integument unmarked with spots or tranverse bands; without pattern from differences in setal colour; setal vestiture even and mainly parallel; Elytral apex spinose? (acute projection at suture) no. **Legs.** Metacoxal plate without elongation in mesal half, plate reaching lateral edge; profemur without carina across full length of anterior face (basodorsal to apicoventral); tarsal pads or membranous lobes absent; tarsal claws with setae, simple. **Ventrites.** Microserration at sides (e.g. 100 points per mm) absent, ventrite 5 apex arcuate without paired setal brushes. **Aedeagus.** Parameres with articulation at base, apical lateral expansions present; each

paramere with three or more setae. **Geography.** Known from Arizona+ (AZ, NM), Texas, Carolinas+ (KY, TN, NC, SC, GA, AL) and Florida ['+' refers to several states or provinces collectively].

5.4 IGNELATER Costa, 1975

Habitus. Body Length 15-20 mm or 20-40 mm. Vestiture. Scale like setae absent. **Head.** Supra-antennal carinae joining medially (forming shelf) or directed anteriorly (reaching anterior part of head capsule); nasale (head capsule below edge of frontal carina) with outline not concave in lateral view; prognathous (labrum oriented less than 90 degrees downward); frons without triangular depression. Antennae with 11 antennomeres, not pectinate, sensory elements beginning on antennomere IV. **Prothorax.** Pronotum wider than long or longer than wide at widest point (including hind angles); dorsal punctures uniform sized, all simple (floor of puncture concave), without tubercles or longitudinal carinae between punctures; pronotal lateral carina complete anteriorly, carina visible throughout length in dorsal view, meeting anterior edge of prothorax at about 90 degrees in lateral view, not serrate; bioluminescent spots present; hind angle carinae present (single); posterior edge of pronotum with sublateral plicae and notches absent, crenellations absent; hypomeron posterior edge near each hind angle with concavity, concavity arcuate; pronotosternal sutures open or closed, hypomeral beads present or absent. Prosternum with sides straight at midlength in ventral view; prosternal process not curved upward more than 40 degrees in lateral view. **Mesothorax.** Mesocoxal cavity open; mesoventral cavity without serration along sides. Elytra. Striae present; anterior edge outline straight to arcuate near humeri in dorsal view; integument unmarked with spots or tranverse bands; without pattern from differences in setal colour; setal vestiture even and mainly parallel; Elytral apex spinose? (acute projection at suture) yes. **Legs.** Metacoxal plate without elongation in mesal half, plate reaching lateral edge; profemur without carina across full length of anterior face (basodorsal to apicoventral); tarsal pads or membranous lobes absent; tarsal claws with setae, simple. **Ventrites.** Microserration at sides (e.g. 100 points per mm) absent, ventrite 5 apex arcuate without paired setal brushes. **Aedeagus.** Parameres with articulation at base, apical lateral expansions present; each paramere with three or more setae. **Geography.** Known from Florida ['+' refers to several states or provinces collectively].

5.4 PYROPHORUS Billberg, 1820

Habitus. Body Length 20-40 mm. Vestiture. Scale like setae absent. **Head.** Supra-antennal carinae joining medially (forming shelf); nasale (head capsule below edge of frontal carina) with outline concave or not concave in lateral view; prognathous (labrum oriented less than 90 degrees downward); frons without triangular depression. Antennae with 11 antennomeres, not pectinate, sensory elements beginning on antennomere IV. **Prothorax.** Pronotum wider than long at widest point (including hind angles); dorsal punctures uniform sized, all simple (floor of puncture concave), without tubercles or longitudinal carinae between punctures; pronotal lateral carina complete anteriorly, carina visible throughout length in dorsal view, meeting anterior edge of prothorax at about 90 degrees in lateral view, not serrate; bioluminescent spots present; hind angle carinae present (single); posterior edge of pronotum with sublateral plicae and notches absent, crenellations absent; hypomeron posterior edge near each hind angle with concavity, concavity arcuate; pronotosternal sutures open, hypomeral beads present or absent. Prosternum with sides straight at midlength in ventral view; prosternal process not curved upward more than 40 degrees in lateral view. **Mesothorax.** Mesocoxal cavity open; mesoventral cavity without serration along sides. Elytra. Striae present or absent; anterior edge outline straight to arcuate near humeri in dorsal view; integument unmarked with spots or tranverse bands; without pattern from differences in setal colour; setal vestiture even and mainly parallel; Elytral apex spinose? (acute projection at suture) yes. **Legs.** Metacoxal plate without elongation in mesal half, plate reaching lateral

edge; profemur without carina across full length of anterior face (basodorsal to apicoventral); tarsal pads or membranous lobes absent; tarsal claws with setae, simple. **Ventrites.** Microserration at sides (e.g. 100 points per mm) absent, ventrite 5 apex arcuate without paired setal brushes. **Aedeagus.** Parameres with articulation at base, apical lateral expansions absent; each paramere with three or more setae.

5.4 VESPERELATER Costa, 1975

Habitus. Body Length 20-40 mm. Vestiture. Scale like setae absent. **Head.** Supra-antennal carinae joining medially (forming shelf); nasale (head capsule below edge of frontal carina) with outline not concave in lateral view; prognathous (labrum oriented less than 90 degrees downward); frons with or without triangular depression. Antennae with 11 antennomeres, not pectinate, sensory elements beginning on antennomere IV. **Prothorax.** Pronotum longer than wide at widest point (including hind angles); dorsal punctures uniform sized, some or all punctures umbilicate (floor of puncture flat), without tubercles or longitudinal carinae between punctures; pronotal lateral carina complete anteriorly, carina visible throughout length in dorsal view, meeting anterior edge of prothorax at about 90 degrees in lateral view, not serrate; bioluminescent spots present; hind angle carinae present (single); posterior edge of pronotum with sublateral plicae and notches absent, crenellations absent; hypomeron posterior edge near each hind angle with concavity, concavity arcuate; pronotosternal sutures open or closed, hypomeral beads present. Prosternum with sides straight at midlength in ventral view; prosternal process not curved upward more than 40 degrees in lateral view. **Mesothorax.** Mesocoxal cavity open; mesoventral cavity without serration along sides. Elytra. Striae present; anterior edge outline straight to arcuate near humeri in dorsal view; integument unmarked with spots or tranverse bands; without pattern from differences in setal colour; setal vestiture even and mainly parallel; Elytral apex spinose? (acute projection at suture) no. **Legs.** Metacoxal plate without elongation in mesal half, plate reaching lateral edge; profemur without carina across full length of anterior face (basodorsal to apicoventral); tarsal pads or membranous lobes absent; tarsal claws with setae, simple. **Ventrites.** Microserration at sides (e.g. 100 points per mm) absent, ventrite 5 apex arcuate without paired setal brushes. **Aedeagus.** Parameres with articulation at base, apical lateral expansions present; each paramere with three or more setae. **Geography.** Known from Arizona+ (AZ, NM) ['+' refers to several states or provinces collectively].

5.5 AEOLUS Eschscholtz, 1829

Habitus. Body Length 1-5 mm or 5-10 mm. Vestiture. Scale like setae absent. **Head.** Supra-antennal carinae joining medially (forming shelf); nasale (head capsule below edge of frontal carina) with outline concave in lateral view; hypognathous (labrum oriented downward 90 degrees or more) or prognathous (labrum oriented less than 90 degrees downward); frons without triangular depression. Antennae with 11 antennomeres, not pectinate, sensory elements beginning on antennomere IV. **Prothorax.** Pronotum wider than long or longer than wide at widest point (including hind angles); dorsal punctures uniform sized, all simple (floor of puncture concave), without tubercles or longitudinal carinae between punctures; pronotal lateral carina complete anteriorly, carina visible throughout length in dorsal view or carina not visible anteriorly in dorsal view, meeting anterior edge of prothorax at about 90 degrees in lateral view, not serrate; bioluminescent spots absent; hind angle carinae present (single); posterior edge of pronotum with sublateral plicae and notches absent, crenellations absent; hypomeron posterior edge near each hind angle with concavity, concavity arcuate or angulate; pronotosternal sutures closed, hypomeral beads present. Prosternum with sides straight at midlength in ventral view; prosternal process not curved upward more than 40 degrees in lateral view. **Mesothorax.** Mesocoxal cavity open

or open to mesepimeron only; mesoventral cavity without serration along sides. Elytra. Striae present; anterior edge outline straight to arcuate near humeri in dorsal view; integument marked with spots or transverse bands or unmarked with spots or tranverse bands; without pattern from differences in setal colour; setal vestiture even and mainly parallel. **Legs.** Metacoxal plate with elongation in mesal half, plate reaching or not reaching lateral edge; profemur with carina across full length of anterior face (basodorsal to apicoventral); tarsal pads or membranous lobes present on tarsomere IV only; tarsal claws with setae or without setae, simple. **Ventrites.** Microserration at sides (e.g. 100 points per mm) absent, ventrite 5 apex arcuate without paired setal brushes. **Aedeagus.** Parameres with articulation at base, apical lateral expansions absent; each paramere with three or more setae. **Geography.** Known from Nunavut+ (NT, NU), British Columbia, Alberta, Manitoba+ (MB, SK), Ontario, Quebec, New Brunswick+ (NB, NS, PE), Oregon+ (WA, OR, ID), California, Colorado+ (NV, UT, CO), Arizona+ (AZ, NM), Nebraska+ (SD, NE, IA), Texas, Indiana+ (IL, IN, OH), Arkansas+ (KS, MO, OK, AR, LA, MS), Pennsylvania+ (PA, WV), Virginia+ (NJ, DE, MD, DC, VA) and Carolinas+ (KY, TN, NC, SC, GA, AL) ['+' refers to several states or provinces collectively].

5.5 DERONOCUS Johnson, 1995

Habitus. Body Length 5-10 mm or 10-15 mm. Vestiture. Scale like setae absent. **Head.** Supra-antennal carinae fading on frons (not reaching another structure) or directed anteriorly (reaching anterior part of head capsule); prognathous (labrum oriented less than 90 degrees downward); frons without triangular depression. Antennae with 11 antennomeres, not pectinate, sensory elements beginning on antennomere IV. **Prothorax.** Pronotum longer than wide at widest point (including hind angles); dorsal punctures uniform sized, all simple (floor of puncture concave), without tubercles or longitudinal carinae between punctures; pronotal lateral carina complete anteriorly, carina not visible anteriorly in dorsal view, meeting anterior edge of prothorax at about 90 degrees in lateral view, not serrate; bioluminescent spots absent; hind angle carinae present (single); posterior edge of pronotum with sublateral plicae and notches present, crenellations absent; hypomeron posterior edge near each hind angle with concavity, concavity arcuate or angulate; pronotosternal sutures open, hypomeral beads present. Prosternum with sides straight at midlength in ventral view; prosternal process not curved upward more than 40 degrees in lateral view. **Mesothorax.** Mesocoxal cavity open; mesoventral cavity without serration along sides. Elytra. Striae present; anterior edge outline straight to arcuate near humeri in dorsal view; integument unmarked with spots or tranverse bands; without pattern from differences in setal colour; setal vestiture even and mainly parallel. **Legs.** Metacoxal plate without elongation in mesal half, plate reaching lateral edge; profemur without carina across full length of anterior face (basodorsal to apicoventral); tarsal pads or membranous lobes absent; tarsal claws with setae, simple. **Ventrites.** Microserration at sides (e.g. 100 points per mm) absent, ventrite 5 apex arcuate without paired setal brushes. **Aedeagus.** Parameres with articulation at base, apical lateral expansions present; each paramere with three or more setae. **Geography.** Known from California ['+' refers to several states or provinces collectively].

5.5 HETERODERES Latreille, 1834

Habitus. Body Length 5-10 mm. Vestiture. Scale like setae absent. **Head.** Supra-antennal carinae joining medially (forming shelf); nasale (head capsule below edge of frontal carina) with outline concave or not concave in lateral view; prognathous (labrum oriented less than 90 degrees downward); frons without triangular depression. Antennae with 11 antennomeres, not pectinate, sensory elements beginning on antennomere IV. **Prothorax.** Pronotum wider than long or longer than wide at widest point (including hind angles); dorsal punctures of two distinct intermixed sizes (heterogeneous), all simple (floor of

puncture concave), without tubercles or longitudinal carinae between punctures; pronotal lateral carina complete anteriorly, carina visible throughout length in dorsal view or carina not visible anteriorly in dorsal view, meeting anterior edge of prothorax at about 90 degrees in lateral view, not serrate; bioluminescent spots absent; hind angle carinae present (single) or present (two carinae); posterior edge of pronotum with sublateral plicae and notches present or absent, crenellations absent; hypomeron posterior edge near each hind angle with concavity, concavity arcuate or angulate; pronotosternal sutures open, hypomeral beads absent. Prosternum with sides straight or concave at midlength in ventral view; prosternal process curved or not curved upward more than 40 degrees in lateral view. **Mesothorax.** Mesocoxal cavity open; mesoventral cavity without serration along sides. Elytra. Striae present; anterior edge outline straight to arcuate near humeri in dorsal view; integument marked with spots or transverse bands or unmarked with spots or tranverse bands; without pattern from differences in setal colour; setal vestiture even and mainly parallel. **Legs.** Metacoxal plate with or without elongation in mesal half, plate reaching lateral edge; profemur without carina across full length of anterior face (basodorsal to apicoventral); tarsal pads or membranous lobes present on tarsomere IV only; tarsal claws with setae, simple. **Ventrites.** Microserration at sides (e.g. 100 points per mm) absent, ventrite 5 apex arcuate without paired setal brushes. **Aedeagus.** Parameres with articulation at base, apical lateral expansions present; each paramere with three or more setae. **Geography.** Known from California, Colorado+ (NV, UT, CO), Arizona+ (AZ, NM), Texas, Arkansas+ (KS, MO, OK, AR, LA, MS), Carolinas+ (KY, TN, NC, SC, GA, AL) and Florida ['+' refers to several states or provinces collectively].

5.5 MONOCREPIDIUS Eschscholtz, 1829

Habitus. Body Length 1-5 mm, 5-10 mm or 10-15 mm. Vestiture. Scale like setae absent. **Head.** Supra-antennal carinae joining medially (forming shelf); nasale (head capsule below edge of frontal carina) with outline concave in lateral view; hypognathous (labrum oriented downward 90 degrees or more) or prognathous (labrum oriented less than 90 degrees downward); frons without triangular depression. Antennae with 11 antennomeres, not pectinate, sensory elements beginning on antennomere IV. **Prothorax.** Pronotum wider than long or longer than wide at widest point (including hind angles); dorsal punctures uniform sized, all simple (floor of puncture concave), without tubercles or longitudinal carinae between punctures; pronotal lateral carina complete anteriorly, carina visible throughout length in dorsal view or carina not visible anteriorly in dorsal view, meeting anterior edge of prothorax at about 90 degrees in lateral view, not serrate; bioluminescent spots absent; hind angle carinae present (single), present (two carinae) or absent; posterior edge of pronotum with sublateral plicae and notches present or absent, crenellations absent; hypomeron posterior edge near each hind angle with or without concavity, concavity arcuate or angulate; pronotosternal sutures open or closed, hypomeral beads present. Prosternum with sides straight or convex at midlength in ventral view; prosternal process not curved upward more than 40 degrees in lateral view. **Mesothorax.** Mesocoxal cavity open or open to mesepimeron only; mesoventral cavity without serration along sides. Elytra. Striae present; anterior edge outline straight to arcuate near humeri in dorsal view; integument marked with spots or transverse bands or unmarked with spots or tranverse bands; with or without pattern from differences in setal colour; setal vestiture even and mainly parallel. **Legs.** Metacoxal plate with or without elongation in mesal half, plate reaching lateral edge; profemur without carina across full length of anterior face (basodorsal to apicoventral); tarsal pads or membranous lobes present on tarsomere IV only; tarsal claws with setae, simple. **Ventrites.** Microserration at sides (e.g. 100 points per mm) absent, ventrite 5 apex arcuate without paired setal brushes. **Aedeagus.** Parameres with articulation at base, apical lateral expansions present or absent; each paramere with three or more setae. **Geography.** Known from Alberta, Manitoba+ (MB, SK), Ontario, Quebec, New Brunswick+ (NB, NS, PE), California, Montana+ (MT, WY, ND), Arizona+ (AZ, NM), Nebraska+ (SD, NE, IA), Texas, Michigan+ (MN, WI, MI), Indiana+ (IL, IN,

OH), Arkansas+ (KS, MO, OK, AR, LA, MS), Vermont+ (ME, VT, NH), Massachusetts+ (MA, CT, RI), New York, Pennsylvania+ (PA, WV), Virginia+ (NJ, DE, MD, DC, VA), Carolinas+ (KY, TN, NC, SC, GA, AL) and Florida ['+' refers to several states or provinces collectively].

6 ASCOLIOCERUS Méquignon, 1930

Habitus. Body Length 1-5 mm or 5-10 mm. Vestiture. Scale like setae absent. **Head.** Supra-antennal carinae joining medially (forming shelf); nasale (head capsule below edge of frontal carina) with outline concave in lateral view; prognathous (labrum oriented less than 90 degrees downward); frons with or without triangular depression. Antennae with 11 antennomeres, not pectinate, sensory elements beginning on antennomere IV. **Prothorax.** Pronotum wider than long at widest point (including hind angles); dorsal punctures uniform sized, all simple (floor of puncture concave), without tubercles or longitudinal carinae between punctures; pronotal lateral carina complete anteriorly, carina visible throughout length in dorsal view, meeting anterior edge of prothorax at about 90 degrees in lateral view, not serrate; bioluminescent spots absent; hind angle carinae present (single); posterior edge of pronotum with sublateral plicae and notches present, crenellations absent; hypomeron posterior edge near each hind angle with concavity, concavity arcuate; pronotosternal sutures closed, hypomeral beads absent. Prosternum with sides straight or convex at midlength in ventral view; prosternal process not curved upward more than 40 degrees in lateral view. **Mesothorax.** Mesocoxal cavity open to mesepimeron only; mesoventral cavity without serration along sides. Elytra. Striae present; anterior edge outline straight to arcuate near humeri in dorsal view; integument unmarked with spots or tranverse bands; without pattern from differences in setal colour; setal vestiture even and mainly parallel. **Legs.** Metacoxal plate with elongation in mesal half, plate not reaching lateral edge; tarsal pads or membranous lobes absent; tarsal claws without setae, simple. **Ventrites.** Microserration at sides (e.g. 100 points per mm) absent, ventrite 5 apex arcuate without paired setal brushes. **Aedeagus.** Parameres with articulation at base, apical lateral expansions absent; each paramere with three or more setae. **Geography.** Known from Nunavut+ (NT, NU), Yukon Territory, Alberta, Manitoba+ (MB, SK), Quebec, Newfoundland and Labrador and Alaska ['+' refers to several states or provinces collectively].

6 BERNINELSONIUS Leseigneur, 1970

Habitus. Body Length 5-10 mm. Vestiture. Scale like setae absent. **Head.** Supra-antennal carinae joining medially (forming shelf); nasale (head capsule below edge of frontal carina) with outline not concave in lateral view; prognathous (labrum oriented less than 90 degrees downward); frons without triangular depression. Antennae with 11 antennomeres, not pectinate, sensory elements beginning on antennomere IV. **Prothorax.** Pronotum wider than long or longer than wide at widest point (including hind angles); dorsal punctures uniform sized, all simple (floor of puncture concave), without tubercles or longitudinal carinae between punctures; pronotal lateral carina complete anteriorly, carina visible throughout length in dorsal view, meeting anterior edge of prothorax at about 90 degrees in lateral view, not serrate; bioluminescent spots absent; hind angle carinae present (single); posterior edge of pronotum with sublateral plicae and notches present, crenellations absent; hypomeron posterior edge near each hind angle with concavity, concavity arcuate; pronotosternal sutures closed, hypomeral beads absent. Prosternum with sides convex at midlength in ventral view; prosternal process not curved upward more than 40 degrees in lateral view. **Mesothorax.** Mesocoxal cavity open to mesepimeron only; mesoventral cavity without serration along sides. Elytra. Striae present; anterior edge outline straight to arcuate near humeri in dorsal view; integument unmarked with spots or tranverse bands; without pattern from differences in setal colour; setal vestiture even and mainly parallel. **Legs.** Metacoxal plate with or without elongation in mesal half, plate reaching or not reaching

lateral edge; tarsal pads or membranous lobes absent; tarsal claws without setae, simple. **Ventrites.** Microserration at sides (e.g. 100 points per mm) absent, ventrite 5 apex arcuate without paired setal brushes. **Aedeagus.** Parameres with articulation at base, apical lateral expansions absent; parameres without setae. **Geography.** Known from Nunavut+ (NT, NU) and Alaska ['+' refers to several states or provinces collectively].

6 DESOLAKERRUS Stibick, 1978

Habitus. Body Length 5-10 mm. Vestiture. Scale like setae absent. **Head.** Supra-antennal carinae joining medially (forming shelf); nasale (head capsule below edge of frontal carina) with outline concave or not concave in lateral view; prognathous (labrum oriented less than 90 degrees downward); frons without triangular depression. Antennae with 11 antennomeres, not pectinate, sensory elements beginning on antennomere IV. **Prothorax.** Pronotum longer than wide at widest point (including hind angles); dorsal punctures of two distinct intermixed sizes (heterogeneous), all simple (floor of puncture concave), without tubercles or longitudinal carinae between punctures; pronotal lateral carina complete anteriorly, carina visible throughout length in dorsal view, meeting anterior edge of prothorax at about 90 degrees in lateral view, not serrate; bioluminescent spots absent; hind angle carinae present (single); posterior edge of pronotum with sublateral plicae and notches absent, crenellations absent; hypomeron posterior edge near each hind angle with concavity, concavity arcuate; pronotosternal sutures closed, hypomeral beads present. Prosternum with sides straight or convex at midlength in ventral view; prosternal process not curved upward more than 40 degrees in lateral view. **Mesothorax.** Mesocoxal cavity open to mesepimeron only; mesoventral cavity without serration along sides. Elytra. Striae present; anterior edge outline straight to arcuate near humeri in dorsal view; integument unmarked with spots or tranverse bands; without pattern from differences in setal colour; setal vestiture even and mainly parallel. **Legs.** Metacoxal plate with elongation in mesal half, plate not reaching lateral edge; tarsal pads or membranous lobes absent; tarsal claws without setae, simple. **Ventrites.** Microserration at sides (e.g. 100 points per mm) absent, ventrite 5 apex arcuate without paired setal brushes. **Aedeagus.** Parameres with articulation at base, apical lateral expansions absent; each paramere with three or more setae. **Geography.** Known from California, Colorado+ (NV, UT, CO) and Arizona+ (AZ, NM) ['+' refers to several states or provinces collectively].

6 HYPNOIDUS Dillwyn, 1829

Habitus. Body Length 1-5 mm or 5-10 mm. Vestiture. Scale like setae present or absent. **Head.** Supra-antennal carinae joining medially (forming shelf); nasale (head capsule below edge of frontal carina) with outline concave or not concave in lateral view; prognathous (labrum oriented less than 90 degrees downward); frons without triangular depression. Antennae with 11 antennomeres, not pectinate, sensory elements beginning on antennomere IV or V. **Prothorax.** Pronotum wider than long at widest point (including hind angles); dorsal punctures uniform sized, all simple (floor of puncture concave), with or without tubercles or longitudinal carinae between punctures; pronotal lateral carina complete anteriorly, carina visible throughout length in dorsal view, meeting anterior edge of prothorax at about 90 degrees in lateral view, not serrate; bioluminescent spots absent; hind angle carinae present (single); posterior edge of pronotum with sublateral plicae and notches present, crenellations absent; hypomeron posterior edge near each hind angle with concavity, concavity arcuate; pronotosternal sutures closed, hypomeral beads absent. Prosternum with sides straight or convex at midlength in ventral view; prosternal process not curved upward more than 40 degrees in lateral view. **Mesothorax.** Mesocoxal cavity open to mesepimeron only; mesoventral cavity without serration along sides. Elytra. Striae present; anterior edge outline straight to arcuate near humeri in dorsal view;

integument marked with spot or band in apical 2/5 only or unmarked with spots or tranverse bands; without pattern from differences in setal colour; setal vestiture even and mainly parallel. **Legs.** Metacoxal plate with elongation in mesal half, plate not reaching lateral edge; tarsal pads or membranous lobes absent; tarsal claws without setae, simple. **Ventrites.** Microserration at sides (e.g. 100 points per mm) absent, ventrite 5 apex arcuate without paired setal brushes. **Aedeagus.** Parameres with articulation at base, apical lateral expansions present or absent; each paramere with one seta. **Geography.** Known from Nunavut+ (NT, NU), Yukon Territory, British Columbia, Alberta, Manitoba+ (MB, SK), Ontario, Quebec, New Brunswick+ (NB, NS, PE), Newfoundland and Labrador, Alaska, Oregon+ (WA, OR, ID), California, Montana+ (MT, WY, ND), Colorado+ (NV, UT, CO), Arizona+ (AZ, NM), Nebraska+ (SD, NE, IA), Texas, Michigan+ (MN, WI, MI), Indiana+ (IL, IN, OH), Arkansas+ (KS, MO, OK, AR, LA, MS), Vermont+ (ME, VT, NH), Massachusetts+ (MA, CT, RI), New York, Pennsylvania+ (PA, WV), Virginia+ (NJ, DE, MD, DC, VA) and Carolinas+ (KY, TN, NC, SC, GA, AL) ['+' refers to several states or provinces collectively].

6 HYPOLITHUS Eschscholtz, 1829

Habitus. Body Length 5-10 mm or 10-15 mm. Vestiture. Scale like setae absent. **Head.** Supra-antennal carinae joining medially (forming shelf); nasale (head capsule below edge of frontal carina) with outline not concave in lateral view; prognathous (labrum oriented less than 90 degrees downward); frons with or without triangular depression. Antennae with 11 antennomeres, not pectinate, sensory elements beginning on antennomere IV. **Prothorax.** Pronotum wider than long at widest point (including hind angles); dorsal punctures uniform sized, all simple (floor of puncture concave), without tubercles or longitudinal carinae between punctures; pronotal lateral carina complete anteriorly, carina visible throughout length in dorsal view, meeting anterior edge of prothorax at about 90 degrees in lateral view, not serrate; bioluminescent spots absent; hind angle carinae present (single); posterior edge of pronotum with sublateral plicae and notches absent, crenellations absent; hypomeron posterior edge near each hind angle with concavity, concavity arcuate; pronotosternal sutures closed, hypomeral beads absent. Prosternum with sides straight at midlength in ventral view; prosternal process not curved upward more than 40 degrees in lateral view. **Mesothorax.** Mesocoxal cavity open; mesoventral cavity without serration along sides. Elytra. Striae present; anterior edge outline straight to arcuate near humeri in dorsal view; integument unmarked with spots or tranverse bands; without pattern from differences in setal colour; setal vestiture even and mainly parallel. **Legs.** Metacoxal plate without elongation in mesal half, plate not reaching lateral edge; tarsal pads or membranous lobes absent; tarsal claws without setae, simple. **Ventrites.** Microserration at sides (e.g. 100 points per mm) absent, ventrite 5 apex arcuate without paired setal brushes. **Aedeagus.** Parameres with articulation at base, apical lateral expansions present; each paramere with three or more setae. **Geography.** Known from Alaska ['+' refers to several states or provinces collectively].

6 LIGMARGUS Stibick, 1976

Habitus. Body Length 5-10 mm or 10-15 mm. Vestiture. Scale like setae absent. **Head.** Supra-antennal carinae joining medially (forming shelf) or directed anteriorly (reaching anterior part of head capsule); nasale (head capsule below edge of frontal carina) with outline not concave in lateral view; prognathous (labrum oriented less than 90 degrees downward); frons without triangular depression. Antennae with 11 antennomeres, not pectinate, sensory elements beginning on antennomere III or IV. **Prothorax.** Pronotum wider than long at widest point (including hind angles); dorsal punctures of two distinct intermixed sizes (heterogeneous), all simple (floor of puncture concave), with or without tubercles or longitudinal carinae between punctures; pronotal lateral carina complete anteriorly, carina

visible throughout length in dorsal view, meeting anterior edge of prothorax at about 90 degrees in lateral view, not serrate; bioluminescent spots absent; hind angle carinae present (single); posterior edge of pronotum with sublateral plicae and notches present, crenellations absent; hypomeron posterior edge near each hind angle with concavity, concavity arcuate; pronotosternal sutures closed, hypomeral beads absent. Prosternum with sides straight or convex at midlength in ventral view; prosternal process not curved upward more than 40 degrees in lateral view. **Mesothorax.** Mesocoxal cavity open to mesepimeron only; mesoventral cavity without serration along sides. Elytra. Striae present; anterior edge outline straight to arcuate near humeri in dorsal view; integument unmarked with spots or tranverse bands; without pattern from differences in setal colour; setal vestiture even and mainly parallel. **Legs.** Metacoxal plate with or without elongation in mesal half, plate not reaching lateral edge; tarsal pads or membranous lobes absent; tarsal claws without setae, simple. **Ventrites.** Microserration at sides (e.g. 100 points per mm) absent, ventrite 5 apex arcuate without paired setal brushes. **Aedeagus.** Parameres with articulation at base or at midlength, fused at base, apical lateral expansions present or absent; parameres without setae or each paramere with three or more setae. **Geography.** Known from Nunavut+ (NT, NU), Yukon Territory, British Columbia, Alberta, Ontario, Quebec, Alaska, Oregon+ (WA, OR, ID), California, Montana+ (MT, WY, ND), Colorado+ (NV, UT, CO), Vermont+ (ME, VT, NH), Massachusetts+ (MA, CT, RI), New York and Pennsylvania+ (PA, WV) ['+' refers to several states or provinces collectively].

6 MARGAIOSTUS Stibick, 1978

Habitus. Body Length 5-10 mm or 10-15 mm. Vestiture. Scale like setae absent. **Head.** Supra-antennal carinae joining medially (forming shelf) or directed anteriorly (reaching anterior part of head capsule); nasale (head capsule below edge of frontal carina) with outline not concave in lateral view; prognathous (labrum oriented less than 90 degrees downward); frons without triangular depression. Antennae with 11 antennomeres, not pectinate, sensory elements beginning on antennomere IV. **Prothorax.** Pronotum wider than long or longer than wide at widest point (including hind angles); dorsal punctures uniform sized, all simple (floor of puncture concave), without tubercles or longitudinal carinae between punctures; pronotal lateral carina complete anteriorly, carina visible throughout length in dorsal view or carina not visible anteriorly in dorsal view, meeting anterior edge of prothorax at about 90 degrees in lateral view, not serrate; bioluminescent spots absent; hind angle carinae present (single); posterior edge of pronotum with sublateral plicae and notches present, crenellations absent; hypomeron posterior edge near each hind angle with concavity, concavity arcuate; pronotosternal sutures closed, hypomeral beads present or absent. Prosternum with sides straight or convex at midlength in ventral view; prosternal process not curved upward more than 40 degrees in lateral view. **Mesothorax.** Mesocoxal cavity open to mesepimeron only; mesoventral cavity without serration along sides. Elytra. Striae present; anterior edge outline straight to arcuate near humeri in dorsal view; integument unmarked with spots or tranverse bands; without pattern from differences in setal colour; setal vestiture even and mainly parallel. **Legs.** Metacoxal plate with elongation in mesal half, plate not reaching lateral edge; tarsal pads or membranous lobes absent; tarsal claws without setae, simple. **Ventrites.** Microserration at sides (e.g. 100 points per mm) absent, ventrite 5 apex arcuate without paired setal brushes. **Aedeagus.** Parameres with articulation at base, apical lateral expansions present; each paramere with one seta or each paramere with two setae. **Geography.** Known from British Columbia, Alberta, Ontario, Quebec, New Brunswick+ (NB, NS, PE), Alaska, Oregon+ (WA, OR, ID), California, Montana+ (MT, WY, ND) and Arkansas+ (KS, MO, OK, AR, LA, MS) ['+' refers to several states or provinces collectively].

7 FLEUTIAUXELLUS Méquignon, 1930

Habitus. Body Length 1-5 mm or 5-10 mm. Vestiture. Scale like setae absent. **Head.** Supra-antennal carinae joining medially (forming shelf) or directed anteriorly (reaching anterior part of head capsule); nasale (head capsule below edge of frontal carina) with outline concave or not concave in lateral view; prognathous (labrum oriented less than 90 degrees downward); frons without triangular depression. Antennae with 11 antennomeres, not pectinate, sensory elements beginning on antennomere III. **Prothorax.** Pronotum wider than long at widest point (including hind angles); dorsal punctures uniform sized, all simple (floor of puncture concave), antero-medial portion with or without tubercles or longitudinal carinae between punctures; pronotal lateral carina complete anteriorly, carina visible throughout length in dorsal view, meeting anterior edge of prothorax at about 90 degrees in lateral view, not serrate; bioluminescent spots absent; hind angle carinae present (single); posterior edge of pronotum with sublateral plicae and notches absent, crenellations absent; hypomeron posterior edge near each hind angle with concavity, concavity arcuate; pronotosternal sutures closed, hypomeral beads present. Prosternum with sides convex at midlength in ventral view; prosternal process not curved upward more than 40 degrees in lateral view. **Mesothorax.** Mesocoxal cavity closed; mesoventral cavity without serration along sides. Elytra. Striae present; anterior edge outline straight to arcuate near humeri in dorsal view; integument unmarked with spots or tranverse bands; without pattern from differences in setal colour; setal vestiture even and mainly parallel. **Legs.** Metacoxal plate with or without elongation in mesal half, plate reaching or not reaching lateral edge; tarsal pads or membranous lobes absent; tarsal claws without setae, simple. **Ventrites.** Microserration at sides (e.g. 100 points per mm) absent, ventrite 5 apex arcuate without paired setal brushes. **Aedeagus.** Parameres with articulation at midlength, fused at base, apical lateral expansions absent; each paramere with three or more setae. **Geography.** Known from Nunavut+ (NT, NU), Yukon Territory, British Columbia, Alberta, Alaska, Montana+ (MT, WY, ND), Michigan+ (MN, WI, MI), Vermont+ (ME, VT, NH) and Massachusetts+ (MA, CT, RI) ['+' refers to several states or provinces collectively].

7 MICROHYPNUS Kishii, 1976

Habitus. Body Length 1-5 mm. Vestiture. Scale like setae absent. **Head.** Supra-antennal carinae joining medially (forming shelf); nasale (head capsule below edge of frontal carina) with outline concave or not concave in lateral view; prognathous (labrum oriented less than 90 degrees downward); frons without triangular depression. Antennae with 11 antennomeres, not pectinate, sensory elements beginning on antennomere IV. **Prothorax.** Pronotum wider than long at widest point (including hind angles); dorsal punctures uniform sized, all simple (floor of puncture concave), with tubercles or longitudinal carinae between punctures; pronotal lateral carina complete anteriorly, carina visible throughout length in dorsal view, meeting anterior edge of prothorax at about 90 degrees in lateral view, not serrate; bioluminescent spots absent; hind angle carinae present (single); posterior edge of pronotum with sublateral plicae and notches absent, crenellations absent; hypomeron posterior edge near each hind angle with concavity, concavity arcuate or angulate; pronotosternal sutures closed, hypomeral beads present. Prosternum with sides convex at midlength in ventral view; prosternal process not curved upward more than 40 degrees in lateral view. **Mesothorax.** Mesocoxal cavity closed; mesoventral cavity without serration along sides. Elytra. Striae present; anterior edge outline straight to arcuate near humeri in dorsal view; integument unmarked with spots or tranverse bands; without pattern from differences in setal colour; setal vestiture even and mainly parallel. **Legs.** Metacoxal plate with elongation in mesal half, plate reaching lateral edge; tarsal pads or membranous lobes absent; tarsal claws without setae, simple. **Ventrites.** Microserration at sides (e.g. 100 points per mm) absent, ventrite 5 apex arcuate without paired setal brushes. **Aedeagus.** Parameres with articulation at midlength, fused at base, apical lateral expansions absent; each paramere with two setae. **Geography.** Known from British Columbia, Alberta, Manitoba+ (MB, SK), Ontario, Quebec, New Brunswick+ (NB, NS, PE), Alaska,

Montana+ (MT, WY, ND), Michigan+ (MN, WI, MI), Indiana+ (IL, IN, OH), Vermont+ (ME, VT, NH), New York and Pennsylvania+ (PA, WV) ['+' refers to several states or provinces collectively].

7 MIGIWA Kishii, 1966

Habitus. Body Length 1-5 mm. Vestiture. Scale like setae absent. **Head.** Supra-antennal carinae joining medially (forming shelf); nasale (head capsule below edge of frontal carina) with outline concave or not concave in lateral view; hypognathous (labrum oriented downward 90 degrees or more); frons without triangular depression. Antennae with 11 antennomeres, not pectinate, sensory elements beginning on antennomere IV. **Prothorax.** Pronotum wider than long at widest point (including hind angles); dorsal punctures uniform sized, some or all punctures umbilicate (floor of puncture flat), without tubercles or longitudinal carinae between punctures; pronotal lateral carina complete anteriorly, carina not visible anteriorly in dorsal view, meeting anterior edge of prothorax at about 90 degrees in lateral view, not serrate; bioluminescent spots absent; hind angle carinae present (single); posterior edge of pronotum with sublateral plicae and notches absent, crenellations absent; hypomeron posterior edge near each hind angle with concavity, concavity arcuate; pronotosternal sutures closed, hypomeral beads present. Prosternum with sides convex at midlength in ventral view; prosternal process not curved upward more than 40 degrees in lateral view. **Mesothorax.** Mesocoxal cavity closed; mesoventral cavity without serration along sides. Elytra. Striae present; anterior edge outline straight to arcuate or sinuate (recurved) or with rectangular projection near humeri in dorsal view; integument unmarked with spots or tranverse bands; without pattern from differences in setal colour; setal vestiture even and mainly parallel. **Legs.** Metacoxal plate with elongation in mesal half, plate reaching lateral edge; tarsal pads or membranous lobes absent; tarsal claws without setae, simple. **Ventrites.** Microserration at sides (e.g. 100 points per mm) absent, ventrite 5 apex arcuate without paired setal brushes. **Aedeagus.** Parameres with articulation at midlength, fused at base, apical lateral expansions absent; each paramere with three or more setae. **Geography.** Known from Alberta, Manitoba+ (MB, SK), Montana+ (MT, WY, ND) and Nebraska+ (SD, NE, IA) ['+' refers to several states or provinces collectively].

7 NEGASTRIUS Thomson, 1859

Habitus. Body Length 1-5 mm. Vestiture. Scale like setae absent. **Head.** Supra-antennal carinae joining medially (forming shelf); nasale (head capsule below edge of frontal carina) with outline concave or not concave in lateral view; prognathous (labrum oriented less than 90 degrees downward); frons without triangular depression. Antennae with 11 antennomeres, not pectinate, sensory elements beginning on antennomere IV. **Prothorax.** Pronotum wider than long at widest point (including hind angles); dorsal punctures uniform sized, all simple (floor of puncture concave), with tubercles or longitudinal carinae between punctures; pronotal lateral carina complete anteriorly or incomplete anteriorly, carina visible throughout length in dorsal view, meeting anterior edge of prothorax at about 90 degrees in lateral view, not serrate; bioluminescent spots absent; hind angle carinae present (single); posterior edge of pronotum with sublateral plicae and notches absent, crenellations absent; hypomeron posterior edge near each hind angle with concavity, concavity arcuate; pronotosternal sutures closed, hypomeral beads present. Prosternum with sides convex at midlength in ventral view; prosternal process not curved upward more than 40 degrees in lateral view. **Mesothorax.** Mesocoxal cavity closed; mesoventral cavity without serration along sides. Elytra. Striae present; anterior edge outline straight to arcuate or sinuate (recurved) or with rectangular projection near humeri in dorsal view; integument marked with spots or transverse bands or unmarked with spots or tranverse bands; without pattern from differences in setal colour; setal vestiture even and mainly parallel. **Legs.** Metacoxal plate with elongation in mesal half, plate reaching lateral edge; tarsal pads or membranous lobes absent; tarsal claws without setae,

simple. **Ventrites.** Microserration at sides (e.g. 100 points per mm) absent, ventrite 5 apex arcuate without paired setal brushes. **Aedeagus.** Parameres with articulation at midlength, fused at base, apical lateral expansions absent; each paramere with two setae. **Geography.** Known from British Columbia, Alberta, Manitoba+ (MB, SK), Ontario, Quebec, New Brunswick+ (NB, NS, PE), Oregon+ (WA, OR, ID), California, Montana+ (MT, WY, ND), Colorado+ (NV, UT, CO), Nebraska+ (SD, NE, IA), Michigan+ (MN, WI, MI), Indiana+ (IL, IN, OH), Arkansas+ (KS, MO, OK, AR, LA, MS), Vermont+ (ME, VT, NH), Massachusetts+ (MA, CT, RI), New York, Pennsylvania+ (PA, WV), Virginia+ (NJ, DE, MD, DC, VA) and Carolinas+ (KY, TN, NC, SC, GA, AL) ['+' refers to several states or provinces collectively].

7 NEOHYPDONUS Stibick, 1971

Habitus. Body Length 1-5 mm. Vestiture. Scale like setae absent. **Head.** Supra-antennal carinae joining medially (forming shelf), fading on frons (not reaching another structure) or directed anteriorly (reaching anterior part of head capsule); nasale (head capsule below edge of frontal carina) with outline concave or not concave in lateral view; hypognathous (labrum oriented downward 90 degrees or more) or prognathous (labrum oriented less than 90 degrees downward); frons without triangular depression. Antennae with 11 antennomeres, not pectinate, sensory elements beginning on antennomere III or IV. **Prothorax.** Pronotum wider than long at widest point (including hind angles); dorsal punctures uniform sized or of two distinct intermixed sizes (heterogeneous), all simple (floor of puncture concave), without tubercles or longitudinal carinae between punctures; pronotal lateral carina complete anteriorly, carina visible throughout length in dorsal view or carina not visible anteriorly in dorsal view, meeting anterior edge of prothorax at about 90 degrees in lateral view, not serrate; bioluminescent spots absent; hind angle carinae present (single); posterior edge of pronotum with sublateral plicae and notches absent, crenellations absent; hypomeron posterior edge near each hind angle with concavity, concavity arcuate; pronotosternal sutures closed, hypomeral beads present or absent. Prosternum with sides convex at midlength in ventral view; prosternal process not curved upward more than 40 degrees in lateral view. **Mesothorax.** Mesocoxal cavity closed; mesoventral cavity without serration along sides. Elytra. Striae present; anterior edge outline straight to arcuate or sinuate (recurved) or with rectangular projection near humeri in dorsal view; integument marked with spots or transverse bands or unmarked with spots or tranverse bands; without pattern from differences in setal colour; setal vestiture even and mainly parallel. **Legs.** Metacoxal plate with elongation in mesal half, plate reaching or not reaching lateral edge; tarsal pads or membranous lobes absent; tarsal claws without setae, simple. **Ventrites.** Microserration at sides (e.g. 100 points per mm) absent, ventrite 5 apex arcuate without paired setal brushes. **Aedeagus.** Parameres with articulation at midlength, fused at base, apical lateral expansions absent; each paramere with two setae. **Geography.** Known from Nunavut+ (NT, NU), Yukon Territory, British Columbia, Alberta, Manitoba+ (MB, SK), Ontario, Quebec, New Brunswick+ (NB, NS, PE), Alaska, Oregon+ (WA, OR, ID), Montana+ (MT, WY, ND), Colorado+ (NV, UT, CO), Nebraska+ (SD, NE, IA), Michigan+ (MN, WI, MI), Indiana+ (IL, IN, OH), Arkansas+ (KS, MO, OK, AR, LA, MS), Vermont+ (ME, VT, NH), New York, Pennsylvania+ (PA, WV), Virginia+ (NJ, DE, MD, DC, VA) and Carolinas+ (KY, TN, NC, SC, GA, AL) ['+' refers to several states or provinces collectively].

7 OEDOSTETHUS LeConte, 1853

Habitus. Body Length 1-5 mm. Vestiture. Scale like setae absent. **Head.** Supra-antennal carinae joining medially (forming shelf); nasale (head capsule below edge of frontal carina) with outline concave or not concave in lateral view; prognathous (labrum oriented less than 90 degrees downward); frons without triangular depression. Antennae with 11 antennomeres, not pectinate, sensory elements beginning on antennomere IV. **Prothorax.** Pronotum wider than long at widest point (including hind angles); dorsal

punctures uniform sized, all simple (floor of puncture concave), without tubercles or longitudinal carinae between punctures; pronotal lateral carina complete anteriorly, carina visible throughout length in dorsal view or carina not visible anteriorly in dorsal view, meeting anterior edge of prothorax at about 90 degrees in lateral view, not serrate; bioluminescent spots absent; hind angle carinae present (single); posterior edge of pronotum with sublateral plicae and notches absent, crenellations absent; hypomeron posterior edge near each hind angle with concavity, concavity arcuate; pronotosternal sutures closed, hypomeral beads present or absent. Prosternum with sides convex at midlength in ventral view; prosternal process not curved upward more than 40 degrees in lateral view. **Mesothorax.** Mesocoxal cavity closed; mesoventral cavity without serration along sides. Elytra. Striae present; anterior edge outline sinuate (recurved) or with rectangular projection near humeri in dorsal view; integument unmarked with spots or tranverse bands; without pattern from differences in setal colour; setal vestiture even and mainly parallel. **Legs.** Metacoxal plate with or without elongation in mesal half, plate reaching lateral edge; tarsal pads or membranous lobes absent; tarsal claws without setae, with two points, or appendiculate. **Ventrites.** Microserration at sides (e.g. 100 points per mm) absent, ventrite 5 apex arcuate without paired setal brushes. **Aedeagus.** Parameres with articulation at midlength, fused at base, apical lateral expansions absent; each paramere with two setae. **Geography.** Known from Alberta, Manitoba+ (MB, SK), Ontario, Quebec, New Brunswick+ (NB, NS, PE), Oregon+ (WA, OR, ID), Montana+ (MT, WY, ND), Colorado+ (NV, UT, CO), Nebraska+ (SD, NE, IA), Michigan+ (MN, WI, MI), Indiana+ (IL, IN, OH), Arkansas+ (KS, MO, OK, AR, LA, MS), Vermont+ (ME, VT, NH), Massachusetts+ (MA, CT, RI), New York, Pennsylvania+ (PA, WV) and Virginia+ (NJ, DE, MD, DC, VA) ['+' refers to several states or provinces collectively].

7 PARADONUS Stibick, 1971

Habitus. Body Length 1-5 mm. Vestiture. Scale like setae absent. **Head.** Supra-antennal carinae joining medially (forming shelf); nasale (head capsule below edge of frontal carina) with outline concave or not concave in lateral view; hypognathous (labrum oriented downward 90 degrees or more) or prognathous (labrum oriented less than 90 degrees downward); frons without triangular depression. Antennae with 11 antennomeres, not pectinate, sensory elements beginning on antennomere IV. **Prothorax.** Pronotum wider than long at widest point (including hind angles); dorsal punctures uniform sized, all simple (floor of puncture concave), without tubercles or longitudinal carinae between punctures; pronotal lateral carina complete anteriorly, carina visible throughout length in dorsal view or carina not visible anteriorly in dorsal view, meeting anterior edge of prothorax at about 90 degrees in lateral view, not serrate; bioluminescent spots absent; hind angle carinae present (single); posterior edge of pronotum with sublateral plicae and notches absent, crenellations absent; hypomeron posterior edge near each hind angle with concavity, concavity arcuate; pronotosternal sutures open or closed, hypomeral beads present. Prosternum with sides convex at midlength in ventral view; prosternal process not curved upward more than 40 degrees in lateral view. **Mesothorax.** Mesocoxal cavity closed; mesoventral cavity without serration along sides. Elytra. Striae absent; anterior edge outline sinuate (recurved) or with rectangular projection near humeri in dorsal view; integument marked with spots or transverse bands, marked with spot or band in apical 2/5 only or unmarked with spots or tranverse bands; without pattern from differences in setal colour; setal vestiture even and mainly parallel. **Legs.** Metacoxal plate with or without elongation in mesal half, plate reaching or not reaching lateral edge; tarsal pads or membranous lobes absent; tarsal claws without setae, simple. **Ventrites.** Microserration at sides (e.g. 100 points per mm) absent, ventrite 5 apex arcuate without paired setal brushes. **Aedeagus.** Parameres with articulation at midlength, fused at base, apical lateral expansions absent; each paramere with one seta. **Geography.** Known from British Columbia, Alberta, Manitoba+ (MB, SK), Ontario, Quebec, New Brunswick+ (NB, NS, PE), California, Montana+ (MT, WY, ND), Nebraska+ (SD, NE, IA), Texas, Michigan+

(MN, WI, MI), Indiana+ (IL, IN, OH), Arkansas+ (KS, MO, OK, AR, LA, MS), Vermont+ (ME, VT, NH), New York, Pennsylvania+ (PA, WV), Virginia+ (NJ, DE, MD, DC, VA) and Carolinas+ (KY, TN, NC, SC, GA, AL) ['+' refers to several states or provinces collectively].

7 ZOROCHROS Thomson, 1859

Habitus. Body Length 1-5 mm. Vestiture. Scale like setae absent. **Head.** Supra-antennal carinae joining medially (forming shelf); nasale (head capsule below edge of frontal carina) with outline concave or not concave in lateral view; hypognathous (labrum oriented downward 90 degrees or more) or prognathous (labrum oriented less than 90 degrees downward); frons without triangular depression. Antennae with 11 antennomeres, not pectinate, sensory elements beginning on antennomere IV. **Prothorax.** Pronotum wider than long at widest point (including hind angles); dorsal punctures uniform sized or of two distinct intermixed sizes (heterogeneous), all simple (floor of puncture concave) or some or all punctures umbilicate (floor of puncture flat), antero-medial portion with or without tubercles or longitudinal carinae between punctures; pronotal lateral carina complete anteriorly, carina visible throughout length in dorsal view or carina not visible anteriorly in dorsal view, meeting anterior edge of prothorax at about 90 degrees in lateral view, not serrate; bioluminescent spots absent; hind angle carinae present (single); posterior edge of pronotum with sublateral plicae and notches absent, crenellations absent; hypomeron posterior edge near each hind angle with concavity, concavity arcuate or angulate; pronotosternal sutures closed, hypomeral beads present. Prosternum with sides convex at midlength in ventral view; prosternal process not curved upward more than 40 degrees in lateral view. **Mesothorax.** Mesocoxal cavity closed; mesoventral cavity without serration along sides. Elytra. Striae present; anterior edge outline straight to arcuate or sinuate (recurved) or with rectangular projection near humeri in dorsal view; integument marked with spots or transverse bands, marked with spot or band in apical 2/5 only or unmarked with spots or tranverse bands; without pattern from differences in setal colour; setal vestiture even and mainly parallel. **Legs.** Metacoxal plate with elongation in mesal half, plate not reaching lateral edge; tarsal pads or membranous lobes absent; tarsal claws without setae, simple. **Ventrites.** Microserration at sides (e.g. 100 points per mm) absent, ventrite 5 apex arcuate without paired setal brushes. **Aedeagus.** Parameres with articulation at midlength, fused at base, apical lateral expansions absent; each paramere with three or more setae. **Geography.** Known from British Columbia, Quebec, New Brunswick+ (NB, NS, PE), Oregon+ (WA, OR, ID), California, Montana+ (MT, WY, ND), Colorado+ (NV, UT, CO), Indiana+ (IL, IN, OH), Vermont+ (ME, VT, NH), New York, Pennsylvania+ (PA, WV), Virginia+ (NJ, DE, MD, DC, VA) and Carolinas+ (KY, TN, NC, SC, GA, AL) ['+' refers to several states or provinces collectively].

8 APHRICUS LeConte, 1853

Habitus. Body Length 1-5 mm or 5-10 mm. Vestiture. Scale like setae absent. **Head.** Supra-antennal carinae joining medially (forming shelf); nasale (head capsule below edge of frontal carina) with outline concave in lateral view; hypognathous (labrum oriented downward 90 degrees or more); frons without triangular depression. Antennae with 11 antennomeres, not pectinate, sensory elements beginning on antennomere III. **Prothorax.** Pronotum wider than long at widest point (including hind angles); dorsal punctures uniform sized, all simple (floor of puncture concave), without tubercles or longitudinal carinae between punctures; pronotal lateral carina incomplete anteriorly, carina not visible anteriorly in dorsal view, not serrate; bioluminescent spots absent; hind angle carinae absent; posterior edge of pronotum with sublateral plicae and notches absent, crenellations absent; hypomeron posterior edge near each hind angle with concavity, concavity arcuate or angulate; pronotosternal sutures closed, hypomeral beads absent. Prosternum with sides concave at midlength in ventral view; prosternal process curved

upward more than 40 degrees in lateral view. **Mesothorax.** Mesocoxal cavity closed; mesoventral cavity without serration along sides. Elytra. Striae present; anterior edge outline straight to arcuate near humeri in dorsal view; integument unmarked with spots or tranverse bands; without pattern from differences in setal colour; setal vestiture even and mainly parallel. **Legs.** Metacoxal plate without elongation in mesal half; tarsal pads or membranous lobes absent; tarsal claws without setae, simple. **Ventrites.** Microserration at sides (e.g. 100 points per mm) absent, ventrite 5 apex arcuate without paired setal brushes. **Aedeagus.** Parameres with articulation at midlength, fused at base, apical lateral expansions present or absent; each paramere with three or more setae. **Geography.** Known from California, Colorado+ (NV, UT, CO) and Arizona+ (AZ, NM) ['+' refers to several states or provinces collectively].

8 APTOPUS Eschscholtz, 1829

Habitus. Body Length 5-10 mm or 10-15 mm. Vestiture. Scale like setae absent. **Head.** Supra-antennal carinae joining medially (forming shelf); nasale (head capsule below edge of frontal carina) with outline concave in lateral view; hypognathous (labrum oriented downward 90 degrees or more); frons without triangular depression. Antennae with 11 antennomeres, not pectinate, sensory elements beginning on antennomere III or IV (by misinterpretation). **Prothorax.** Pronotum wider than long at widest point (including hind angles); dorsal punctures uniform sized (by misinterpretation) or of two distinct intermixed sizes (heterogeneous), all simple (floor of puncture concave), without tubercles or longitudinal carinae between punctures; pronotal lateral carina incomplete anteriorly, carina not visible anteriorly in dorsal view, not serrate; bioluminescent spots absent; hind angle carinae present (single) or absent (by misinterpretation); posterior edge of pronotum with sublateral plicae and notches present, crenellations absent; hypomeron posterior edge near each hind angle with concavity, concavity arcuate or angulate; pronotosternal sutures closed, hypomeral beads present or absent. Prosternum with sides concave at midlength in ventral view; prosternal process curved or not curved upward more than 40 degrees in lateral view. **Mesothorax.** Mesocoxal cavity closed; mesoventral cavity without serration along sides. Elytra. Striae present; anterior edge outline straight to arcuate or sinuate (recurved) or with rectangular projection near humeri in dorsal view; integument unmarked with spots or tranverse bands; without pattern from differences in setal colour; setal vestiture even and mainly parallel. **Legs.** Metacoxal plate with elongation in mesal half, plate not reaching lateral edge; tarsal pads or membranous lobes absent; tarsal claws without setae, with 3 or more points. **Ventrites.** Microserration at sides (e.g. 100 points per mm) present or absent, ventrite 5 apex arcuate without paired setal brushes. **Aedeagus.** Parameres with articulation at midlength, fused at base, apical lateral expansions present or absent; each paramere with two setae. **Geography.** Known from California, Arizona+ (AZ, NM) and Texas ['+' refers to several states or provinces collectively].

8 CARDIOPHORUS Eschscholtz, 1829

Habitus. Body Length 5-10 mm or 10-15 mm. Vestiture. Scale like setae absent. **Head.** Supra-antennal carinae joining medially (forming shelf); nasale (head capsule below edge of frontal carina) with outline concave in lateral view; hypognathous (labrum oriented downward 90 degrees or more) or prognathous (labrum oriented less than 90 degrees downward); frons with or without triangular depression. Antennae with 11 antennomeres, not pectinate, sensory elements beginning on antennomere III. **Prothorax.** Pronotum wider than long at widest point (including hind angles); dorsal punctures of two distinct intermixed sizes (heterogeneous), all simple (floor of puncture concave), without tubercles or longitudinal carinae between punctures; pronotal lateral carina incomplete anteriorly, carina not visible anteriorly in dorsal view, not serrate; bioluminescent spots absent; hind angle carinae present (single);

posterior edge of pronotum with sublateral plicae and notches present, crenellations absent; hypomeron posterior edge near each hind angle with concavity, concavity angulate; pronotosternal sutures closed, hypomeral beads present or absent. Prosternum with sides straight or concave at midlength in ventral view; prosternal process curved or not curved upward more than 40 degrees in lateral view. **Mesothorax.** Mesocoxal cavity closed; mesoventral cavity without serration along sides. Elytra. Striae present; anterior edge outline sinuate (recurved) or with rectangular projection near humeri in dorsal view; integument marked with spots or transverse bands or unmarked with spots or tranverse bands; with or without pattern from differences in setal colour; setal vestiture even and mainly parallel. **Legs.** Metacoxal plate with or without elongation in mesal half, plate not reaching lateral edge; tarsal pads or membranous lobes absent; tarsal claws without setae, simple. **Ventrites.** Microserration at sides (e.g. 100 points per mm) present or absent, ventrite 5 apex arcuate without paired setal brushes. **Aedeagus.** Parameres with articulation at midlength, fused at base, apical lateral expansions present or absent; each paramere with one seta or each paramere with two setae. **Geography.** Known from British Columbia, Alberta, Manitoba+ (MB, SK), Ontario, Quebec, New Brunswick+ (NB, NS, PE), Oregon+ (WA, OR, ID), California, Montana+ (MT, WY, ND), Colorado+ (NV, UT, CO), Arizona+ (AZ, NM), Nebraska+ (SD, NE, IA), Texas, Michigan+ (MN, WI, MI), Indiana+ (IL, IN, OH), Arkansas+ (KS, MO, OK, AR, LA, MS), Vermont+ (ME, VT, NH), Massachusetts+ (MA, CT, RI), New York, Pennsylvania+ (PA, WV), Virginia+ (NJ, DE, MD, DC, VA), Carolinas+ (KY, TN, NC, SC, GA, AL) and Florida ['+' refers to several states or provinces collectively].

8 ESTHESOPUS Eschscholtz, 1829

Habitus. Body Length 1-5 mm or 5-10 mm. Vestiture. Scale like setae absent. **Head.** Supra-antennal carinae joining medially (forming shelf); nasale (head capsule below edge of frontal carina) with outline concave in lateral view; hypognathous (labrum oriented downward 90 degrees or more); frons without triangular depression. Antennae with 11 antennomeres, not pectinate, sensory elements beginning on antennomere III or IV. **Prothorax.** Pronotum wider than long at widest point (including hind angles); dorsal punctures of two distinct intermixed sizes (heterogeneous), all simple (floor of puncture concave), without tubercles or longitudinal carinae between punctures; pronotal lateral carina complete anteriorly or incomplete anteriorly, not serrate; bioluminescent spots absent; hind angle carinae present (single) or absent; posterior edge of pronotum with sublateral plicae and notches absent, crenellations absent; hypomeron posterior edge near each hind angle with concavity, concavity arcuate or angulate; pronotosternal sutures closed, hypomeral beads present or absent. Prosternum with sides concave at midlength in ventral view; prosternal process curved or not curved upward more than 40 degrees in lateral view. **Mesothorax.** Mesocoxal cavity closed; mesoventral cavity without serration along sides. Elytra. Striae present; anterior edge outline straight to arcuate or sinuate (recurved) or with rectangular projection near humeri in dorsal view; integument marked with spots or transverse bands or unmarked with spots or tranverse bands; without pattern from differences in setal colour; setal vestiture even and mainly parallel. **Legs.** Metacoxal plate with or without elongation in mesal half, plate not reaching lateral edge; tarsal pads or membranous lobes present on tarsomere IV only; tarsal claws without setae, with two points, or appendiculate. **Ventrites.** Microserration at sides (e.g. 100 points per mm) present, ventrite 5 apex arcuate without paired setal brushes. **Aedeagus.** Parameres with articulation at midlength, fused at base, apical lateral expansions absent; each paramere with two setae. **Geography.** Known from Ontario, California, Colorado+ (NV, UT, CO), Arizona+ (AZ, NM), Texas, Indiana+ (IL, IN, OH), Arkansas+ (KS, MO, OK, AR, LA, MS), Virginia+ (NJ, DE, MD, DC, VA), Carolinas+ (KY, TN, NC, SC, GA, AL) and Florida ['+' refers to several states or provinces collectively].

8 FLORIDELATER Douglas, 2017

Habitus. Body Length 1-5 mm or 5-10 mm. Vestiture. Scale like setae absent. **Head.** Supra-antennal carinae joining medially (forming shelf); nasale (head capsule below edge of frontal carina) with outline concave in lateral view; hypognathous (labrum oriented downward 90 degrees or more); frons without triangular depression. Antennae with 11 antennomeres, not pectinate, sensory elements beginning on antennomere III. **Prothorax.** Pronotum wider than long at widest point (including hind angles); dorsal punctures uniform sized, all simple (floor of puncture concave), with (by misinterpretation) or without tubercles or longitudinal carinae between punctures; pronotal lateral carina incomplete anteriorly, carina not visible anteriorly in dorsal view, not serrate; bioluminescent spots absent; hind angle carinae present (single); posterior edge of pronotum with sublateral plicae and notches present, crenellations absent; hypomeron posterior edge near each hind angle with concavity, concavity arcuate or angulate; pronotosternal sutures open, hypomeral beads present. Prosternum with sides concave at midlength in ventral view; prosternal process curved upward more than 40 degrees in lateral view. **Mesothorax.** Mesocoxal cavity closed; mesoventral cavity without serration along sides. Elytra. Striae present; anterior edge outline straight to arcuate near humeri in dorsal view; integument unmarked with spots or tranverse bands; without pattern from differences in setal colour; setal vestiture mainly absent on disk or even and mainly parallel. **Legs.** Metacoxal plate without elongation in mesal half, plate reaching or not reaching lateral edge; tarsal pads or membranous lobes absent; tarsal claws without setae, simple. **Ventrites.** Microserration at sides (e.g. 100 points per mm) absent, ventrite 5 apex arcuate without paired setal brushes. **Aedeagus.** Parameres with articulation at midlength, fused at base, apical lateral expansions present or absent; each paramere with two setae. **Geography.** Known from Arkansas+ (KS, MO, OK, AR, LA, MS), Carolinas+ (KY, TN, NC, SC, GA, AL) and Florida ['+' refers to several states or provinces collectively].

8 HORISTONOTUS Candèze, 1860

Habitus. Body Length 1-5 mm or 5-10 mm. Vestiture. Scale like setae absent. **Head.** Supra-antennal carinae joining medially (forming shelf); nasale (head capsule below edge of frontal carina) with outline concave in lateral view; hypognathous (labrum oriented downward 90 degrees or more); frons without triangular depression. Antennae with 11 antennomeres, not pectinate, sensory elements beginning on antennomere III or IV. **Prothorax.** Pronotum wider than long at widest point (including hind angles); dorsal punctures of two distinct intermixed sizes (heterogeneous), all simple (floor of puncture concave) or some or all punctures umbilicate (floor of puncture flat), without tubercles or longitudinal carinae between punctures; pronotal lateral carina incomplete anteriorly, not serrate; bioluminescent spots absent; hind angle carinae absent; posterior edge of pronotum with sublateral plicae and notches present or absent, crenellations absent; hypomeron posterior edge near each hind angle with concavity, concavity angulate; pronotosternal sutures closed, hypomeral beads present or absent. Prosternum with sides concave at midlength in ventral view; prosternal process not curved upward more than 40 degrees in lateral view. **Mesothorax.** Mesocoxal cavity closed; mesoventral cavity without serration along sides. Elytra. Striae present; anterior edge outline sinuate (recurved) or with rectangular projection near humeri in dorsal view; integument marked with spots or transverse bands or unmarked with spots or tranverse bands; without pattern from differences in setal colour; setal vestiture even and mainly parallel. **Legs.** Metacoxal plate with or without elongation in mesal half, plate not reaching lateral edge; tarsal pads or membranous lobes absent; tarsal claws without setae, with two points, or appendiculate, with 3 or more points (by misinterpretation) or simple. **Ventrites.** Microserration at sides (e.g. 100 points per mm) present, ventrite 5 apex arcuate without paired setal brushes. **Aedeagus.** Parameres with articulation at midlength, fused at base, apical lateral expansions absent; each paramere with two setae. **Geography.** Known from British Columbia, Ontario, Oregon+ (WA, OR, ID), California, Montana+ (MT, WY, ND), Arizona+ (AZ, NM), Texas, Indiana+ (IL, IN, OH), New

York, Pennsylvania+ (PA, WV), Virginia+ (NJ, DE, MD, DC, VA), Carolinas+ (KY, TN, NC, SC, GA, AL) and Florida ['+' refers to several states or provinces collectively].

8 PARACARDIOPHORUS Schwarz, 1895

Habitus. Body Length 5-10 mm or 10-15 mm. Vestiture. Scale like setae absent. **Head.** Supra-antennal carinae joining medially (forming shelf); nasale (head capsule below edge of frontal carina) with outline concave in lateral view; hypognathous (labrum oriented downward 90 degrees or more) or prognathous (labrum oriented less than 90 degrees downward); frons without triangular depression. Antennae with 11 antennomeres, not pectinate, sensory elements beginning on antennomere III. **Prothorax.** Pronotum wider than long at widest point (including hind angles); dorsal punctures uniform sized or of two distinct intermixed sizes (heterogeneous), all simple (floor of puncture concave), without tubercles or longitudinal carinae between punctures; pronotal lateral carina incomplete anteriorly, carina not visible anteriorly in dorsal view, not serrate; bioluminescent spots absent; hind angle carinae present (single); posterior edge of pronotum with sublateral plicae and notches present, crenellations absent; hypomeron posterior edge near each hind angle with concavity, concavity arcuate; pronotosternal sutures closed, hypomeral beads present. Prosternum with sides straight or concave at midlength in ventral view; prosternal process curved or not curved upward more than 40 degrees in lateral view. **Mesothorax.** Mesocoxal cavity closed; mesoventral cavity without serration along sides. Elytra. Striae present; anterior edge outline straight to arcuate near humeri in dorsal view; integument marked with spots or transverse bands or unmarked with spots or tranverse bands; without pattern from differences in setal colour; setal vestiture even and mainly parallel. **Legs.** Metacoxal plate with elongation in mesal half, plate not reaching lateral edge; tarsal pads or membranous lobes absent; tarsal claws without setae, simple. **Ventrites.** Microserration at sides (e.g. 100 points per mm) absent, ventrite 5 apex arcuate without paired setal brushes. **Aedeagus.** Parameres with articulation at midlength, fused at base, apical lateral expansions absent; each paramere with one seta or each paramere with two setae. **Geography.** Known from Nunavut+ (NT, NU), British Columbia, Alberta, Manitoba+ (MB, SK), Ontario, Quebec, New Brunswick+ (NB, NS, PE), Oregon+ (WA, OR, ID), California, Montana+ (MT, WY, ND), Colorado+ (NV, UT, CO), Michigan+ (MN, WI, MI), Indiana+ (IL, IN, OH), Arkansas+ (KS, MO, OK, AR, LA, MS), Vermont+ (ME, VT, NH), Massachusetts+ (MA, CT, RI), New York, Pennsylvania+ (PA, WV), Virginia+ (NJ, DE, MD, DC, VA) and Florida ['+' refers to several states or provinces collectively].

9.1 MELANACTES LeConte, 1853

Habitus. Body Length 20-40 mm. Vestiture. Scale like setae absent. **Head.** Supra-antennal carinae directed anteriorly (reaching anterior part of head capsule); prognathous (labrum oriented less than 90 degrees downward); frons without triangular depression. Antennae with 11 antennomeres, not pectinate, sensory elements beginning on antennomere IV. **Prothorax.** Pronotum wider than long or longer than wide at widest point (including hind angles); dorsal punctures uniform sized, all simple (floor of puncture concave), without tubercles or longitudinal carinae between punctures; pronotal lateral carina complete anteriorly, carina visible throughout length in dorsal view, meeting anterior edge of prothorax at about 90 degrees in lateral view, not serrate; bioluminescent spots absent; hind angle carinae present (single); posterior edge of pronotum with sublateral plicae and notches absent, crenellations present or absent; hypomeron posterior edge near each hind angle with concavity, concavity arcuate or angulate; pronotosternal sutures closed, hypomeral beads present. Prosternum with sides straight at midlength in ventral view; prosternal process not curved upward more than 40 degrees in lateral view. **Mesothorax.** Mesocoxal cavity open; mesoventral cavity without serration along sides. Elytra. Striae absent; anterior edge outline straight to arcuate near humeri in dorsal view;

integument unmarked with spots or tranverse bands; without pattern from differences in setal colour; setal vestiture mainly absent on disk. **Legs.** Metacoxal plate without elongation in mesal half, plate reaching lateral edge; tarsal pads or membranous lobes absent; tarsal claws without setae, simple. **Ventrites.** Microserration at sides (e.g. 100 points per mm) absent, ventrite 5 apex arcuate without paired setal brushes. **Aedeagus.** Parameres with articulation at base, apical lateral expansions present or absent; each paramere with three or more setae. **Geography.** Known from California, Nebraska+ (SD, NE, IA), Indiana+ (IL, IN, OH), Arkansas+ (KS, MO, OK, AR, LA, MS), Massachusetts+ (MA, CT, RI), New York, Pennsylvania+ (PA, WV), Virginia+ (NJ, DE, MD, DC, VA), Carolinas+ (KY, TN, NC, SC, GA, AL) and Florida ['+' refers to several states or provinces collectively].

9.1 OISTUS Candèze, 1857

Habitus. Body Length 15-20 mm or 20-40 mm. Vestiture. Scale like setae absent. **Head.** Supra-antennal carinae fading on frons (not reaching another structure), directed anteriorly (reaching anterior part of head capsule) or absent; prognathous (labrum oriented less than 90 degrees downward); frons without triangular depression. Antennae with 11 antennomeres, not pectinate, sensory elements beginning on antennomere IV. **Prothorax.** Pronotum wider than long at widest point (including hind angles); dorsal punctures uniform sized, all simple (floor of puncture concave), without tubercles or longitudinal carinae between punctures; pronotal lateral carina complete anteriorly, carina visible throughout length in dorsal view, meeting anterior edge of prothorax at about 90 degrees in lateral view, not serrate; bioluminescent spots absent; hind angle carinae absent; posterior edge of pronotum with sublateral plicae and notches absent, crenellations present; hypomeron posterior edge near each hind angle with concavity, concavity arcuate or angulate; pronotosternal sutures closed, hypomeral beads present or absent. Prosternum with sides straight at midlength in ventral view; prosternal process not curved upward more than 40 degrees in lateral view. **Mesothorax.** Mesocoxal cavity open or open to mesepimeron only; mesoventral cavity without serration along sides. Elytra. Striae present; anterior edge outline straight to arcuate near humeri in dorsal view; integument marked with spots or transverse bands or unmarked with spots or tranverse bands; without pattern from differences in setal colour; setal vestiture even and mainly parallel or with bare patches. **Legs.** Metacoxal plate without elongation in mesal half, plate reaching lateral edge; tarsal pads or membranous lobes absent; tarsal claws without setae, simple. **Ventrites.** Microserration at sides (e.g. 100 points per mm) absent, ventrite 5 apex arcuate without paired setal brushes. **Aedeagus.** Parameres with articulation at base, apical lateral expansions present; parameres without setae. **Geography.** Known from Oregon+ (WA, OR, ID) and California ['+' refers to several states or provinces collectively].

9.1 PERISSARTHRON Hyslop, 1917

Habitus. Body Length 15-20 mm or 20-40 mm. Vestiture. Scale like setae absent. **Head.** Supra-antennal carinae fading on frons (not reaching another structure) or directed anteriorly (reaching anterior part of head capsule); prognathous (labrum oriented less than 90 degrees downward); frons without triangular depression. Antennae with 11 (by misinterpretation) or 12 antennomeres, not pectinate, sensory elements beginning on antennomere III. **Prothorax.** Pronotum wider than long at widest point (including hind angles); dorsal punctures uniform sized, all simple (floor of puncture concave), without tubercles or longitudinal carinae between punctures; pronotal lateral carina complete anteriorly, carina visible throughout length in dorsal view, meeting anterior edge of prothorax at about 90 degrees in lateral view, not serrate; bioluminescent spots absent; hind angle carinae present (single) or absent (by misinterpretation); posterior edge of pronotum with sublateral plicae and notches absent, crenellations present; hypomeron posterior edge near each hind angle with concavity, concavity arcuate;

pronotosternal sutures closed, hypomeral beads present or absent (by misinterpretation). Prosternum with sides straight at midlength in ventral view; prosternal process curved or not curved upward more than 40 degrees in lateral view. **Mesothorax.** Mesocoxal cavity open; mesoventral cavity without serration along sides. Elytra. Striae present or absent (by misinterpretation); anterior edge outline straight to arcuate near humeri in dorsal view; integument unmarked with spots or tranverse bands; without pattern from differences in setal colour; setal vestiture even and mainly parallel. **Legs.** Metacoxal plate without elongation in mesal half, plate reaching or not reaching lateral edge; tarsal pads or membranous lobes absent; tarsal claws without setae, simple. **Ventrites.** Microserration at sides (e.g. 100 points per mm) absent, ventrite 5 apex arcuate without paired setal brushes. **Aedeagus.** Parameres with articulation at base, apical lateral expansions present or absent; each paramere with two setae. **Geography.** Known from Texas ['+' refers to several states or provinces collectively].

9.2 ACTENICEROMORPHUS Kishii

Habitus. Body Length 10-15 mm or 15-20 mm. Vestiture. Scale like setae absent. **Head.** Supra-antennal carinae fading on frons (not reaching another structure); prognathous (labrum oriented less than 90 degrees downward); frons without triangular depression. Antennae with 11 antennomeres, not pectinate, sensory elements beginning on antennomere III. **Prothorax.** Pronotum wider than long or longer than wide at widest point (including hind angles); dorsal punctures uniform sized, all simple (floor of puncture concave), without tubercles or longitudinal carinae between punctures; pronotal lateral carina complete anteriorly, carina visible throughout length in dorsal view, meeting anterior edge of prothorax at about 90 degrees in lateral view, not serrate; bioluminescent spots absent; hind angle carinae present (single); posterior edge of pronotum with sublateral plicae and notches present, crenellations absent; hypomeron posterior edge near each hind angle with concavity, concavity arcuate; pronotosternal sutures closed, hypomeral beads absent. Prosternum with sides straight at midlength in ventral view; prosternal process not curved upward more than 40 degrees in lateral view. **Mesothorax.** Mesocoxal cavity open; mesoventral cavity without serration along sides. Elytra. Striae present; anterior edge outline straight to arcuate near humeri in dorsal view; integument unmarked with spots or tranverse bands; without pattern from differences in setal colour; setal vestiture even and mainly parallel. **Legs.** Metacoxal plate without elongation in mesal half, plate reaching lateral edge; tarsal pads or membranous lobes absent; tarsal claws without setae, simple. **Ventrites.** Microserration at sides (e.g. 100 points per mm) absent, ventrite 5 apex arcuate without paired setal brushes. **Aedeagus.** Parameres with articulation at base, apical lateral expansions present or absent; each paramere with two setae or each paramere with three or more setae. **Geography.** Known from Nunavut+ (NT, NU), British Columbia, Alberta, Manitoba+ (MB, SK), Ontario, Quebec, New Brunswick+ (NB, NS, PE), Newfoundland and Labrador, Alaska, Oregon+ (WA, OR, ID), California, Montana+ (MT, WY, ND), Colorado+ (NV, UT, CO) and New York ['+' refers to several states or provinces collectively].

A. sagitticollis (Eschscholtz)

Habitus. Body Length 10-15 mm. Vestiture. Scale like setae absent. **Head.** Supra-antennal carinae fading on frons (not reaching another structure); prognathous (labrum oriented less than 90 degrees downward); frons without triangular depression. Antennae with 11 antennomeres, not pectinate, sensory elements beginning on antennomere IV. **Prothorax.** Pronotum wider than long at widest point (including hind angles); dorsal punctures uniform sized, all simple (floor of puncture concave), without tubercles or longitudinal carinae between punctures; pronotal lateral carina complete anteriorly, carina

visible throughout length in dorsal view, meeting anterior edge of prothorax at about 90 degrees in lateral view, not serrate; bioluminescent spots absent; hind angle carinae present (single); posterior edge of pronotum with sublateral plicae and notches present, crenellations absent; hypomeron posterior edge near each hind angle with concavity, concavity arcuate; pronotosternal sutures closed, hypomeral beads absent. Prosternum with sides straight at midlength in ventral view; prosternal process not curved upward more than 40 degrees in lateral view. **Mesothorax.** Mesocoxal cavity open; mesoventral cavity without serration along sides. Elytra. Striae present; anterior edge outline straight to arcuate near humeri in dorsal view; integument unmarked with spots or tranverse bands; without pattern from differences in setal colour; setal vestiture even and mainly parallel. **Legs.** Metacoxal plate without elongation in mesal half, plate reaching lateral edge; tarsal pads or membranous lobes absent; tarsal claws without setae, simple. **Ventrites.** Microserration at sides (e.g. 100 points per mm) absent, ventrite 5 apex arcuate without paired setal brushes. **Aedeagus.** Parameres with articulation at base, apical lateral expansions absent; each paramere with three or more setae. **Geography.** Known from British Columbia, Alaska, Oregon+ (WA, OR, ID) and Montana+ (MT, WY, ND) ['+' refers to several states or provinces collectively].

9.2 ACTENICERUS Kiesenwetter, 1858

Habitus. Body Length 10-15 mm or 15-20 mm. Vestiture. Scale like setae absent. **Head.** Supra-antennal carinae fading on frons (not reaching another structure); prognathous (labrum oriented less than 90 degrees downward); frons without triangular depression. Antennae with 11 antennomeres, not pectinate, sensory elements beginning on antennomere III. **Prothorax.** Pronotum wider than long or longer than wide at widest point (including hind angles); dorsal punctures uniform sized, all simple (floor of puncture concave) or some or all punctures umbilicate (floor of puncture flat), without tubercles or longitudinal carinae between punctures; pronotal lateral carina complete anteriorly, carina visible throughout length in dorsal view, meeting anterior edge of prothorax at about 90 degrees in lateral view, not serrate; bioluminescent spots absent; hind angle carinae present (single) or absent; posterior edge of pronotum with sublateral plicae and notches present or absent, crenellations absent; hypomeron posterior edge near each hind angle with concavity, concavity arcuate; pronotosternal sutures closed, hypomeral beads present. Prosternum with sides straight at midlength in ventral view; prosternal process not curved upward more than 40 degrees in lateral view. **Mesothorax.** Mesocoxal cavity open; mesoventral cavity without serration along sides. Elytra. Striae present; anterior edge outline straight to arcuate near humeri in dorsal view; integument unmarked with spots or tranverse bands; with pattern from differences in setal colour; setal vestiture even and mainly parallel or partially transverse in patches. **Legs.** Metacoxal plate without elongation in mesal half, plate reaching lateral edge; tarsal pads or membranous lobes absent; tarsal claws without setae, simple. **Ventrites.** Microserration at sides (e.g. 100 points per mm) absent, ventrite 5 apex arcuate without paired setal brushes. **Aedeagus.** Parameres with articulation at base, apical lateral expansions present; parameres without setae. **Geography.** Known from Ontario, Quebec, New Brunswick+ (NB, NS, PE), Nebraska+ (SD, NE, IA), Michigan+ (MN, WI, MI), Vermont+ (ME, VT, NH), Massachusetts+ (MA, CT, RI), New York, Pennsylvania+ (PA, WV) and Virginia+ (NJ, DE, MD, DC, VA) ['+' refers to several states or provinces collectively].

9.2 ANOSTIRUS C.G. Thomson, 1859

Habitus. Body Length 5-10 mm or 10-15 mm. Vestiture. Scale like setae absent. **Head.** Supra-antennal carinae fading on frons (not reaching another structure); prognathous (labrum oriented less than 90 degrees downward); frons without triangular depression. Antennae with 11 antennomeres, pectinate or

bipectinate (by misinterpretation) or not pectinate, sensory elements beginning on antennomere III or IV. **Prothorax.** Pronotum wider than long or longer than wide at widest point (including hind angles); dorsal punctures uniform sized, all simple (floor of puncture concave), without tubercles or longitudinal carinae between punctures; pronotal lateral carina complete anteriorly, carina visible throughout length in dorsal view, meeting anterior edge of prothorax at about 90 degrees in lateral view, not serrate; bioluminescent spots absent; hind angle carinae absent; posterior edge of pronotum with sublateral plicae and notches present, crenellations absent; hypomeron posterior edge near each hind angle with concavity; pronotosternal sutures closed, hypomeral beads present or absent. Prosternum with sides straight at midlength in ventral view; prosternal process not curved upward more than 40 degrees in lateral view. **Mesothorax.** Mesocoxal cavity open; mesoventral cavity without serration along sides. Elytra. Striae present; anterior edge outline straight to arcuate near humeri in dorsal view; integument marked with spots or transverse bands; with or without pattern from differences in setal colour; setal vestiture even and mainly parallel. **Legs.** Metacoxal plate without elongation in mesal half, plate reaching lateral edge; tarsal pads or membranous lobes absent; tarsal claws without setae, simple. **Ventrites.** Microserration at sides (e.g. 100 points per mm) present, ventrite 5 apex arcuate without paired setal brushes. **Aedeagus.** Parameres with articulation at base, apical lateral expansions present or absent; each paramere with two setae or each paramere with three or more setae. **Geography.** Known from British Columbia, Ontario, Quebec, New Brunswick+ (NB, NS, PE), California, Montana+ (MT, WY, ND), Indiana+ (IL, IN, OH), Vermont+ (ME, VT, NH), Massachusetts+ (MA, CT, RI), Pennsylvania+ (PA, WV), Virginia+ (NJ, DE, MD, DC, VA) and Carolinas+ (KY, TN, NC, SC, GA, AL) ['+' refers to several states or provinces collectively].

9.2 ANTHRACOPTERYX Horn, 1891

Habitus. Body Length 5-10 mm. Vestiture. Scale like setae absent. **Head.** Supra-antennal carinae fading on frons (not reaching another structure); prognathous (labrum oriented less than 90 degrees downward); frons without triangular depression. Antennae with 11 antennomeres, not pectinate, sensory elements beginning on antennomere IV. **Prothorax.** Pronotum wider than long at widest point (including hind angles); dorsal punctures uniform sized, all simple (floor of puncture concave), without tubercles or longitudinal carinae between punctures; pronotal lateral carina complete anteriorly, carina visible throughout length in dorsal view, meeting anterior edge of prothorax at about 90 degrees in lateral view, not serrate; bioluminescent spots absent; hind angle carinae present (single); posterior edge of pronotum with sublateral plicae and notches absent, crenellations absent; hypomeron posterior edge near each hind angle with concavity, concavity angulate; pronotosternal sutures closed, hypomeral beads absent. Prosternum with sides concave at midlength in ventral view; prosternal process not curved upward more than 40 degrees in lateral view. **Mesothorax.** Mesocoxal cavity open; mesoventral cavity without serration along sides. Elytra. Striae present; anterior edge outline straight to arcuate near humeri in dorsal view; integument unmarked with spots or tranverse bands; without pattern from differences in setal colour; setal vestiture even and mainly parallel. **Legs.** Metacoxal plate without elongation in mesal half, plate reaching or not reaching lateral edge; tarsal pads or membranous lobes absent; tarsal claws without setae, simple. **Ventrites.** Microserration at sides (e.g. 100 points per mm) absent, ventrite 5 apex arcuate without paired setal brushes. **Aedeagus.** Parameres with articulation at base, apical lateral expansions present; each paramere with three or more setae. **Geography.** Known from Montana+ (MT, WY, ND) and Colorado+ (NV, UT, CO) ['+' refers to several states or provinces collectively].

9.2 ATHOPLASTUS Johnson and Etzler, 2018

Habitus. Body Length 10-15 mm or 15-20 mm. Vestiture. Scale like setae absent. **Head.** Supra-antennal carinae fading on frons (not reaching another structure); prognathous (labrum oriented less than 90 degrees downward); frons without triangular depression. Antennae with 11 antennomeres, not pectinate, sensory elements beginning on antennomere IV. **Prothorax.** Pronotum wider than long at widest point (including hind angles); dorsal punctures uniform sized, all simple (floor of puncture concave), without tubercles or longitudinal carinae between punctures; pronotal lateral carina complete anteriorly, carina visible throughout length in dorsal view, meeting anterior edge of prothorax at about 90 degrees in lateral view, not serrate; bioluminescent spots absent; hind angle carinae present (single); posterior edge of pronotum with sublateral plicae and notches present, crenellations absent; hypomeron posterior edge near each hind angle with concavity, concavity arcuate; pronotosternal sutures closed, hypomeral beads present or absent. Prosternum with sides concave at midlength in ventral view; prosternal process curved upward more than 40 degrees in lateral view. **Mesothorax.** Mesocoxal cavity open; mesoventral cavity without serration along sides. Elytra. Striae present; anterior edge outline straight to arcuate near humeri in dorsal view; integument unmarked with spots or tranverse bands; without pattern from differences in setal colour; setal vestiture even and mainly parallel. **Legs.** Metacoxal plate without elongation in mesal half, plate reaching lateral edge; tarsal pads or membranous lobes absent; tarsal claws without setae, simple. **Ventrites.** Microserration at sides (e.g. 100 points per mm) absent, ventrite 5 apex arcuate without paired setal brushes. **Aedeagus.** Parameres with articulation at base, apical lateral expansions present; each paramere with three or more setae. **Geography.** Known from Oregon+ (WA, OR, ID) and Montana+ (MT, WY, ND) ['+' refers to several states or provinces collectively].

9.2 BECKERUS Johnson, 2008

Habitus. Body Length 5-10 mm or 10-15 mm. Vestiture. Scale like setae absent. **Head.** Supra-antennal carinae fading on frons (not reaching another structure); prognathous (labrum oriented less than 90 degrees downward); frons without triangular depression. Antennae with 11 antennomeres, pectinate or bipectinate (by misinterpretation) or not pectinate, sensory elements beginning on antennomere III. **Prothorax.** Pronotum wider than long at widest point (including hind angles); dorsal punctures uniform sized, all simple (floor of puncture concave), without tubercles or longitudinal carinae between punctures; pronotal lateral carina complete anteriorly, carina visible throughout length in dorsal view, meeting anterior edge of prothorax at about 90 degrees in lateral view, not serrate; bioluminescent spots absent; hind angle carinae present (single); posterior edge of pronotum with sublateral plicae and notches absent, crenellations present; hypomeron posterior edge near each hind angle with concavity, concavity arcuate; pronotosternal sutures closed, hypomeral beads absent. Prosternum with sides concave at midlength in ventral view; prosternal process curved upward more than 40 degrees in lateral view. **Mesothorax.** Mesocoxal cavity open; mesoventral cavity without serration along sides. Elytra. Striae present; anterior edge outline straight to arcuate near humeri in dorsal view; integument marked with spots or transverse bands; setal vestiture mainly absent on disk or even and mainly parallel. **Legs.** Metacoxal plate without elongation in mesal half, plate reaching lateral edge; tarsal pads or membranous lobes absent; tarsal claws without setae, simple. **Ventrites.** Microserration at sides (e.g. 100 points per mm) absent, ventrite 5 apex arcuate without paired setal brushes. **Aedeagus.** Parameres with articulation at base, apical lateral expansions present; each paramere with three or more setae. **Geography.** Known from Ontario, Quebec, New Brunswick+ (NB, NS, PE), Newfoundland and Labrador, Oregon+ (WA, OR, ID), Montana+ (MT, WY, ND) and Vermont+ (ME, VT, NH) ['+' refers to several states or provinces collectively].

9.2 BILLROWNIA Johnson, 2021. (B. conjugens LeConte, also B. lecontei, B. obversa, B. rupestris)

Habitus. Body Length 5-10 mm or 10-15 mm. Vestiture. Scale like setae absent. **Head.** Supra-antennal carinae fading on frons (not reaching another structure) or directed anteriorly (reaching anterior part of head capsule); nasale (head capsule below edge of frontal carina) with outline not concave in lateral view; prognathous (labrum oriented less than 90 degrees downward); frons without triangular depression. Antennae with 11 antennomeres, not pectinate, sensory elements beginning on antennomere IV. **Prothorax.** Pronotum wider than long or longer than wide at widest point (including hind angles); dorsal punctures uniform sized, all simple (floor of puncture concave), without tubercles or longitudinal carinae between punctures; pronotal lateral carina complete anteriorly, carina visible throughout length in dorsal view, meeting anterior edge of prothorax at about 90 degrees in lateral view, not serrate; bioluminescent spots absent; hind angle carinae present (single); posterior edge of pronotum with sublateral plicae and notches present, crenellations absent; hypomeron posterior edge near each hind angle with concavity, concavity arcuate; pronotosternal sutures closed, hypomeral beads absent. Prosternum with sides straight at midlength in ventral view; prosternal process not curved upward more than 40 degrees in lateral view. **Mesothorax.** Mesocoxal cavity open; mesoventral cavity without serration along sides. Elytra. Striae present; anterior edge outline straight to arcuate near humeri in dorsal view; integument unmarked with spots or tranverse bands; without pattern from differences in setal colour; setal vestiture mainly absent on disk or even and mainly parallel. **Legs.** Metacoxal plate without elongation in mesal half, plate reaching lateral edge; tarsal pads or membranous lobes absent; tarsal claws without setae, simple. **Ventrites.** Microserration at sides (e.g. 100 points per mm) absent, ventrite 5 apex arcuate without paired setal brushes. **Aedeagus.** Parameres with articulation at base, apical lateral expansions absent; parameres without setae. **Geography.** Known from British Columbia, Oregon+ (WA, OR, ID), California, Montana+ (MT, WY, ND) and Colorado+ (NV, UT, CO) ['+' refers to several states or provinces collectively].

B. copei, B. praeses, B. signaticollis

Habitus. Body Length 5-10 mm or 10-15 mm. Vestiture. Scale like setae absent. **Head.** Supra-antennal carinae fading on frons (not reaching another structure) or directed anteriorly (reaching anterior part of head capsule); nasale (head capsule below edge of frontal carina) with outline not concave in lateral view; prognathous (labrum oriented less than 90 degrees downward); frons without triangular depression. Antennae with 11 antennomeres, not pectinate, sensory elements beginning on antennomere III. **Prothorax.** Pronotum wider than long or longer than wide at widest point (including hind angles); dorsal punctures uniform sized, all simple (floor of puncture concave), without tubercles or longitudinal carinae between punctures; pronotal lateral carina complete anteriorly, carina visible throughout length in dorsal view, meeting anterior edge of prothorax at about 90 degrees in lateral view, not serrate; bioluminescent spots absent; hind angle carinae present (single); posterior edge of pronotum with sublateral plicae and notches present, crenellations absent; hypomeron posterior edge near each hind angle with concavity, concavity arcuate; pronotosternal sutures closed, hypomeral beads absent. Prosternum with sides straight or convex at midlength in ventral view; prosternal process not curved upward more than 40 degrees in lateral view. **Mesothorax.** Mesocoxal cavity open; mesoventral cavity without serration along sides. Elytra. Striae present; anterior edge outline straight to arcuate near humeri in dorsal view; integument unmarked with spots or tranverse bands; without pattern from differences in setal colour; setal vestiture mainly absent on disk or even and mainly parallel. **Legs.** Metacoxal plate without elongation in mesal half, plate reaching lateral edge; tarsal pads or membranous lobes absent; tarsal claws without setae, simple. **Ventrites.** Microserration at sides (e.g. 100 points per mm) absent, ventrite 5 apex arcuate without paired setal brushes. **Aedeagus.** Parameres with articulation at base, apical lateral expansions present or absent; parameres without setae. **Geography.** Known from British Columbia, Oregon+ (WA, OR, ID), California, Montana+ (MT, WY,

ND), Colorado+ (NV, UT, CO), Indiana+ (IL, IN, OH), Arkansas+ (KS, MO, OK, AR, LA, MS), Pennsylvania+ (PA, WV) and Carolinas+ (KY, TN, NC, SC, GA, AL) ['+' refers to several states or provinces collectively].

9.2 CORYMBITODES Buysson, 1904

Habitus. Body Length 5-10 mm or 10-15 mm. Vestiture. Scale like setae absent. **Head.** Supra-antennal carinae fading on frons (not reaching another structure) or directed anteriorly (reaching anterior part of head capsule); prognathous (labrum oriented less than 90 degrees downward); frons without triangular depression. Antennae with 11 antennomeres, not pectinate, sensory elements beginning on antennomere III. **Prothorax.** Pronotum longer than wide at widest point (including hind angles); dorsal punctures uniform sized, some or all punctures umbilicate (floor of puncture flat), without tubercles or longitudinal carinae between punctures; pronotal lateral carina complete anteriorly, carina visible throughout length in dorsal view, meeting anterior edge of prothorax at about 90 degrees in lateral view, not serrate; bioluminescent spots absent; hind angle carinae absent; posterior edge of pronotum with sublateral plicae and notches present or absent, crenellations absent; hypomeron posterior edge near each hind angle with concavity, concavity arcuate; pronotosternal sutures closed, hypomeral beads present. Prosternum with sides straight at midlength in ventral view; prosternal process curved or not curved upward more than 40 degrees in lateral view. **Mesothorax.** Mesocoxal cavity open; mesoventral cavity without serration along sides. Elytra. Striae present; anterior edge outline straight to arcuate near humeri in dorsal view; integument unmarked with spots or tranverse bands; without pattern from differences in setal colour; setal vestiture even and mainly parallel. **Legs.** Metacoxal plate without elongation in mesal half, plate reaching or not reaching lateral edge; tarsal pads or membranous lobes absent; tarsal claws without setae, simple. **Ventrites.** Microserration at sides (e.g. 100 points per mm) absent, ventrite 5 apex arcuate without paired setal brushes. **Aedeagus.** Parameres with articulation at base, apical lateral expansions absent; parameres without setae, each paramere with one seta or each paramere with two setae. **Geography.** Known from Nunavut+ (NT, NU), British Columbia, Alberta, Manitoba+ (MB, SK), Ontario, Quebec, New Brunswick+ (NB, NS, PE), Newfoundland and Labrador, Alaska, Oregon+ (WA, OR, ID), California, Montana+ (MT, WY, ND), Colorado+ (NV, UT, CO), Michigan+ (MN, WI, MI), Indiana+ (IL, IN, OH), Vermont+ (ME, VT, NH), Massachusetts+ (MA, CT, RI), New York, Pennsylvania+ (PA, WV), Virginia+ (NJ, DE, MD, DC, VA) and Carolinas+ (KY, TN, NC, SC, GA, AL) ['+' refers to several states or provinces collectively].

9.2 CTENICERA Latreille, 1829

Habitus. Body Length 10-15 mm or 15-20 mm. Vestiture. Scale like setae absent. **Head.** Supra-antennal carinae fading on frons (not reaching another structure); prognathous (labrum oriented less than 90 degrees downward); frons without triangular depression. Antennae with 11 antennomeres, pectinate or bipectinate or not pectinate, sensory elements beginning on antennomere III. **Prothorax.** Pronotum wider than long at widest point (including hind angles); dorsal punctures uniform sized, all simple (floor of puncture concave), without tubercles or longitudinal carinae between punctures; pronotal lateral carina complete anteriorly, carina visible throughout length in dorsal view, meeting anterior edge of prothorax at about 90 degrees in lateral view, not serrate; bioluminescent spots absent; hind angle carinae present (single); posterior edge of pronotum with sublateral plicae and notches present, crenellations absent; hypomeron posterior edge near each hind angle with concavity, concavity arcuate; pronotosternal sutures closed, hypomeral beads present. Prosternum with sides straight at midlength in ventral view; prosternal process not curved upward more than 40 degrees in lateral view. **Mesothorax.** Mesocoxal cavity open; mesoventral cavity without serration along sides. Elytra. Striae present; anterior edge outline straight to arcuate near humeri in dorsal view; integument marked

with spot or band in apical 2/5 only; without pattern from differences in setal colour; setal vestiture even and mainly parallel. **Legs.** Metacoxal plate without elongation in mesal half, plate reaching lateral edge; tarsal pads or membranous lobes absent; tarsal claws without setae, simple. **Ventrites.** Microserration at sides (e.g. 100 points per mm) absent, ventrite 5 apex arcuate without paired setal brushes. **Aedeagus.** Parameres with articulation at base, apical lateral expansions absent; each paramere with three or more setae. **Geography.** Known from Nunavut+ (NT, NU), Yukon Territory, British Columbia, Alberta, Manitoba+ (MB, SK), Ontario, Quebec, New Brunswick+ (NB, NS, PE), Newfoundland and Labrador, Alaska, Oregon+ (WA, OR, ID), Nebraska+ (SD, NE, IA), Michigan+ (MN, WI, MI) and Vermont+ (ME, VT, NH) ['+' refers to several states or provinces collectively].

C. angularis (LeConte)

Habitus. Body Length 5-10 mm or 10-15 mm. Vestiture. Scale like setae absent. **Head.** Supra-antennal carinae fading on frons (not reaching another structure); prognathous (labrum oriented less than 90 degrees downward); frons without triangular depression. Antennae with 11 antennomeres, not pectinate, sensory elements beginning on antennomere IV. **Prothorax.** Pronotum wider than long or longer than wide at widest point (including hind angles); dorsal punctures uniform sized, all simple (floor of puncture concave), without tubercles or longitudinal carinae between punctures; pronotal lateral carina complete anteriorly, carina visible throughout length in dorsal view, meeting anterior edge of prothorax at about 90 degrees in lateral view, not serrate; bioluminescent spots absent; hind angle carinae present (single); posterior edge of pronotum with sublateral plicae and notches present, crenellations absent; hypomeron posterior edge near each hind angle with or without (by misinterpretation) concavity, concavity arcuate; pronotosternal sutures closed, hypomeral beads present. Prosternum with sides straight at midlength in ventral view; prosternal process not curved upward more than 40 degrees in lateral view. **Mesothorax.** Mesocoxal cavity open; mesoventral cavity without serration along sides. Elytra. Striae present; anterior edge outline straight to arcuate near humeri in dorsal view; integument unmarked with spots or tranverse bands; without pattern from differences in setal colour; setal vestiture even and mainly parallel. **Legs.** Metacoxal plate without elongation in mesal half, plate reaching lateral edge; tarsal pads or membranous lobes absent; tarsal claws without setae, simple. **Ventrites.** Microserration at sides (e.g. 100 points per mm) absent, ventrite 5 apex arcuate without paired setal brushes. **Aedeagus.** Parameres with articulation at base, apical lateral expansions present; each paramere with one seta or each paramere with two setae. **Geography.** Known from British Columbia, Oregon+ (WA, OR, ID) and California ['+' refers to several states or provinces collectively].

C. uliginosa (Van Dyke)

Habitus. Body Length 5-10 mm or 10-15 mm. Vestiture. Scale like setae absent. **Head.** Supra-antennal carinae fading on frons (not reaching another structure); prognathous (labrum oriented less than 90 degrees downward); frons without triangular depression. Antennae with 11 antennomeres, not pectinate, sensory elements beginning on antennomere IV. **Prothorax.** Pronotum wider than long at widest point (including hind angles); dorsal punctures uniform sized, all simple (floor of puncture concave), without tubercles or longitudinal carinae between punctures; pronotal lateral carina complete anteriorly, carina visible throughout length in dorsal view, meeting anterior edge of prothorax at about 90 degrees in lateral view, not serrate; bioluminescent spots absent; hind angle carinae present (single); posterior edge of pronotum with sublateral plicae and notches present, crenellations absent; hypomeron posterior edge near each hind angle with concavity, concavity arcuate; pronotosternal sutures closed, hypomeral beads absent. Prosternum with sides straight at midlength in ventral view;

prosternal process not curved upward more than 40 degrees in lateral view. **Mesothorax.** Mesocoxal cavity open; mesoventral cavity without serration along sides. Elytra. Striae present; anterior edge outline straight to arcuate near humeri in dorsal view; integument unmarked with spots or tranverse bands; without pattern from differences in setal colour; setal vestiture even and mainly parallel. **Legs.** Metacoxal plate without elongation in mesal half, plate reaching lateral edge; tarsal pads or membranous lobes absent; tarsal claws without setae, simple. **Ventrites.** Microserration at sides (e.g. 100 points per mm) absent, ventrite 5 apex arcuate without paired setal brushes. **Aedeagus.** Parameres with articulation at base, apical lateral expansions present; each paramere with two setae or each paramere with three or more setae. **Geography.** Known from British Columbia, Oregon+ (WA, OR, ID) and California ['+' refers to several states or provinces collectively].

9.2 DIXICOLLIS Johnson, 2021

Habitus. Body Length 5-10 mm or 10-15 mm. Vestiture. Scale like setae absent. **Head.** Supra-antennal carinae fading on frons (not reaching another structure); prognathous (labrum oriented less than 90 degrees downward); frons without triangular depression. Antennae with 11 antennomeres, not pectinate, sensory elements beginning on antennomere III. **Prothorax.** Pronotum wider than long at widest point (including hind angles); dorsal punctures uniform sized, all simple (floor of puncture concave), without tubercles or longitudinal carinae between punctures; pronotal lateral carina complete anteriorly, carina visible throughout length in dorsal view, meeting anterior edge of prothorax at about 90 degrees in lateral view, not serrate; bioluminescent spots absent; hind angle carinae present (single); posterior edge of pronotum with sublateral plicae and notches absent, crenellations absent; hypomeron posterior edge near each hind angle with concavity, concavity arcuate or angulate; pronotosternal sutures open, hypomeral beads present. Prosternum with sides straight at midlength in ventral view; prosternal process curved upward more than 40 degrees in lateral view. **Mesothorax.** Mesocoxal cavity open; mesoventral cavity without serration along sides. Elytra. Striae present; anterior edge outline straight to arcuate near humeri in dorsal view; integument unmarked with spots or tranverse bands; without pattern from differences in setal colour; setal vestiture even and mainly parallel. **Legs.** Metacoxal plate without elongation in mesal half, plate reaching lateral edge; tarsal pads or membranous lobes absent; tarsal claws without setae, simple. **Ventrites.** Microserration at sides (e.g. 100 points per mm) absent, ventrite 5 apex arcuate without paired setal brushes. **Aedeagus.** Parameres with articulation at base, apical lateral expansions present; parameres without setae. **Geography.** Known from Texas, Indiana+ (IL, IN, OH), Arkansas+ (KS, MO, OK, AR, LA, MS), Pennsylvania+ (PA, WV), Carolinas+ (KY, TN, NC, SC, GA, AL) and Florida ['+' refers to several states or provinces collectively].

9.2 EANUS LeConte, 1861

Habitus. Body Length 1-5 mm or 5-10 mm. Vestiture. Scale like setae absent. **Head.** Supra-antennal carinae fading on frons (not reaching another structure); hypognathous (labrum oriented downward 90 degrees or more) or prognathous (labrum oriented less than 90 degrees downward); frons without triangular depression. Antennae with 11 antennomeres, not pectinate, sensory elements beginning on antennomere III or IV. **Prothorax.** Pronotum wider than long at widest point (including hind angles); dorsal punctures uniform sized, all simple (floor of puncture concave), without tubercles or longitudinal carinae between punctures; pronotal lateral carina complete anteriorly or incomplete anteriorly, carina visible throughout length in dorsal view, not serrate; bioluminescent spots absent; hind angle carinae absent; posterior edge of pronotum with sublateral plicae and notches absent, crenellations absent; hypomeron posterior edge near each hind angle with concavity, concavity arcuate; pronotosternal

sutures closed, hypomeral beads present. Prosternum with sides concave at midlength in ventral view; prosternal process curved upward more than 40 degrees in lateral view. **Mesothorax.** Mesocoxal cavity open; mesoventral cavity without serration along sides. Elytra. Striae present or absent; anterior edge outline straight to arcuate near humeri in dorsal view; integument marked with spots or transverse bands, marked with spot or band in apical 2/5 only or unmarked with spots or tranverse bands; without pattern from differences in setal colour; setal vestiture even and mainly parallel. **Legs.** Metacoxal plate without elongation in mesal half, plate reaching lateral edge; tarsal pads or membranous lobes absent; tarsal claws without setae, simple. **Ventrites.** Microserration at sides (e.g. 100 points per mm) absent, ventrite 5 apex arcuate without paired setal brushes. **Aedeagus.** Parameres with articulation at base, apical lateral expansions present or absent; each paramere with one seta or each paramere with two setae. **Geography.** Known from Nunavut+ (NT, NU), Yukon Territory, British Columbia, Alberta, Manitoba+ (MB, SK), Ontario, Quebec, New Brunswick+ (NB, NS, PE), Newfoundland and Labrador, Alaska, Oregon+ (WA, OR, ID), Montana+ (MT, WY, ND), Michigan+ (MN, WI, MI), Vermont+ (ME, VT, NH) and New York ['+' refers to several states or provinces collectively].

E. striatipennis Brown

Habitus. Body Length 5-10 mm. Vestiture. Scale like setae absent. **Head.** Supra-antennal carinae fading on frons (not reaching another structure); prognathous (labrum oriented less than 90 degrees downward); frons without triangular depression. Antennae with 11 antennomeres, not pectinate, sensory elements beginning on antennomere IV. **Prothorax.** Pronotum wider than long at widest point (including hind angles); dorsal punctures uniform sized, all simple (floor of puncture concave), without tubercles or longitudinal carinae between punctures; pronotal lateral carina complete anteriorly, carina visible throughout length in dorsal view, meeting anterior edge of prothorax at about 90 degrees in lateral view, not serrate; bioluminescent spots absent; hind angle carinae absent; posterior edge of pronotum with sublateral plicae and notches absent, crenellations absent; hypomeron posterior edge near each hind angle with concavity, concavity arcuate; pronotosternal sutures closed, hypomeral beads absent. Prosternum with sides straight or concave at midlength in ventral view; prosternal process curved upward more than 40 degrees in lateral view. **Mesothorax.** Mesocoxal cavity open; mesoventral cavity without serration along sides. Elytra. Striae present; anterior edge outline straight to arcuate near humeri in dorsal view; integument with metallic reflections; without pattern from differences in setal colour; setal vestiture even and mainly parallel. **Legs.** Metacoxal plate without elongation in mesal half, plate reaching lateral edge; tarsal pads or membranous lobes absent; tarsal claws without setae, simple. **Ventrites.** Microserration at sides (e.g. 100 points per mm) absent, ventrite 5 apex arcuate without paired setal brushes. **Aedeagus.** Parameres with articulation at base, apical lateral expansions present; each paramere with three or more setae. **Geography.** Known from British Columbia, Alaska and Oregon+ (WA, OR, ID) ['+' refers to several states or provinces collectively].

9.2 HADROMORPHUS Motschulsky, 1859

Habitus. Body Length 5-10 mm or 10-15 mm. Vestiture. Scale like setae absent. **Head.** Supra-antennal carinae fading on frons (not reaching another structure); prognathous (labrum oriented less than 90 degrees downward); frons without triangular depression. Antennae with 11 antennomeres, not pectinate, sensory elements beginning on antennomere IV. **Prothorax.** Pronotum wider than long at widest point (including hind angles); dorsal punctures uniform sized, some or all punctures umbilicate (floor of puncture flat), without tubercles or longitudinal carinae between punctures; pronotal lateral carina complete anteriorly, carina visible throughout length in dorsal view, meeting anterior edge of prothorax at about 90 degrees in lateral view, not serrate; bioluminescent spots absent; hind angle

carinae present (single) (by misinterpretation) or absent; posterior edge of pronotum with sublateral plicae and notches present or absent, crenellations absent; hypomeron posterior edge near each hind angle with concavity, concavity arcuate; pronotosternal sutures open, hypomeral beads present or absent (by misinterpretation). Prosternum with sides straight at midlength in ventral view; prosternal process not curved upward more than 40 degrees in lateral view. **Mesothorax.** Mesocoxal cavity open or open to mesepimeron only; mesoventral cavity without serration along sides. Elytra. Striae present; anterior edge outline straight to arcuate near humeri in dorsal view; integument unmarked with spots or tranverse bands; without pattern from differences in setal colour; setal vestiture even and mainly parallel. **Legs.** Metacoxal plate without elongation in mesal half, plate reaching or not reaching lateral edge; tarsal pads or membranous lobes absent; tarsal claws without setae, simple. **Ventrites.** Microserration at sides (e.g. 100 points per mm) present or absent, ventrite 5 apex arcuate without paired setal brushes. **Aedeagus.** Parameres with articulation at base, apical lateral expansions present; each paramere with three or more setae. **Geography.** Known from British Columbia, Alberta, Manitoba+ (MB, SK), Ontario, Quebec, Oregon+ (WA, OR, ID), California, Montana+ (MT, WY, ND), Colorado+ (NV, UT, CO), Nebraska+ (SD, NE, IA), Indiana+ (IL, IN, OH), Arkansas+ (KS, MO, OK, AR, LA, MS), Vermont+ (ME, VT, NH), Massachusetts+ (MA, CT, RI), New York, Pennsylvania+ (PA, WV), Virginia+ (NJ, DE, MD, DC, VA) and Carolinas+ (KY, TN, NC, SC, GA, AL) ['+' refers to several states or provinces collectively].

9.2 HYPOGANUS Kiesenwetter, 1858

Habitus. Body Length 5-10 mm or 10-15 mm. Vestiture. Scale like setae absent. **Head.** Supra-antennal carinae fading on frons (not reaching another structure); prognathous (labrum oriented less than 90 degrees downward); frons without triangular depression. Antennae with 11 antennomeres, not pectinate, sensory elements beginning on antennomere IV. **Prothorax.** Pronotum wider than long at widest point (including hind angles); dorsal punctures uniform sized, all simple (floor of puncture concave), without tubercles or longitudinal carinae between punctures; pronotal lateral carina complete anteriorly, carina visible throughout length in dorsal view, meeting anterior edge of prothorax at about 90 degrees in lateral view, not serrate; bioluminescent spots absent; hind angle carinae present (single); posterior edge of pronotum with sublateral plicae and notches absent, crenellations absent; hypomeron posterior edge near each hind angle with concavity, concavity arcuate; pronotosternal sutures closed, hypomeral beads present. Prosternum with sides straight or convex at midlength in ventral view; prosternal process not curved upward more than 40 degrees in lateral view. **Mesothorax.** Mesocoxal cavity open; mesoventral cavity without serration along sides. Elytra. Striae present; anterior edge outline straight to arcuate near humeri in dorsal view; integument unmarked with spots or tranverse bands; without pattern from differences in setal colour; setal vestiture mainly absent on disk or even and mainly parallel. **Legs.** Metacoxal plate without elongation in mesal half, plate reaching lateral edge; tarsal pads or membranous lobes absent; tarsal claws without setae, simple. **Ventrites.** Microserration at sides (e.g. 100 points per mm) absent, ventrite 5 apex arcuate without paired setal brushes. **Aedeagus.** Parameres with articulation at base, apical lateral expansions present; parameres without setae. **Geography.** Known from British Columbia, Ontario, Quebec, New Brunswick+ (NB, NS, PE), Oregon+ (WA, OR, ID), California, Montana+ (MT, WY, ND), Indiana+ (IL, IN, OH), Vermont+ (ME, VT, NH), New York and Pennsylvania+ (PA, WV) ['+' refers to several states or provinces collectively].

9.2 LANEGANUS Johnson, 2001

Habitus. Body Length 5-10 mm, 10-15 mm or 15-20 mm. Vestiture. Scale like setae absent. **Head.** Supra-antennal carinae joining medially (forming shelf) or fading on frons (not reaching another structure);

nasale (head capsule below edge of frontal carina) with outline not concave in lateral view; prognathous (labrum oriented less than 90 degrees downward); frons without triangular depression. Antennae with 11 antennomeres, not pectinate, sensory elements beginning on antennomere IV. **Prothorax.** Pronotum longer than wide at widest point (including hind angles); dorsal punctures uniform sized, all simple (floor of puncture concave), without tubercles or longitudinal carinae between punctures; pronotal lateral carina complete anteriorly, carina visible throughout length in dorsal view, meeting anterior edge of prothorax at about 90 degrees in lateral view, not serrate; bioluminescent spots absent; hind angle carinae present (single); posterior edge of pronotum with sublateral plicae and notches absent, crenellations absent; hypomeron posterior edge near each hind angle with concavity, concavity arcuate; pronotosternal sutures closed, hypomeral beads present. Prosternum with sides straight or convex at midlength in ventral view; prosternal process not curved upward more than 40 degrees in lateral view. **Mesothorax.** Mesocoxal cavity open or open to mesepimeron only; mesoventral cavity without serration along sides. Elytra. Striae present; anterior edge outline straight to arcuate near humeri in dorsal view; integument marked with spot or band in apical 2/5 only or unmarked with spots or tranverse bands; without pattern from differences in setal colour; setal vestiture even and mainly parallel. **Legs.** Metacoxal plate without elongation in mesal half, plate reaching or not reaching lateral edge; tarsal pads or membranous lobes absent; tarsal claws without setae, simple. **Ventrites.** Microserration at sides (e.g. 100 points per mm) absent, ventrite 5 apex arcuate without paired setal brushes. **Aedeagus.** Parameres with articulation at base, apical lateral expansions present; parameres without setae or each paramere with three or more setae. **Geography.** Known from Manitoba+ (MB, SK), Ontario, Quebec, New Brunswick+ (NB, NS, PE), Oregon+ (WA, OR, ID), California, Montana+ (MT, WY, ND), Arizona+ (AZ, NM), Michigan+ (MN, WI, MI), Indiana+ (IL, IN, OH), Arkansas+ (KS, MO, OK, AR, LA, MS), Vermont+ (ME, VT, NH), Massachusetts+ (MA, CT, RI), New York, Pennsylvania+ (PA, WV), Virginia+ (NJ, DE, MD, DC, VA) and Carolinas+ (KY, TN, NC, SC, GA, AL) ['+' refers to several states or provinces collectively].

9.2 LIOTRICHUS kiesenwetter

Habitus. Body Length 5-10 mm or 10-15 mm. Vestiture. Scale like setae absent. **Head.** Supra-antennal carinae fading on frons (not reaching another structure); prognathous (labrum oriented less than 90 degrees downward); frons without triangular depression. Antennae with 11 antennomeres, not pectinate, sensory elements beginning on antennomere IV. **Prothorax.** Pronotum wider than long or longer than wide at widest point (including hind angles); dorsal punctures uniform sized, all simple (floor of puncture concave), without tubercles or longitudinal carinae between punctures; pronotal lateral carina complete anteriorly, carina visible throughout length in dorsal view, meeting anterior edge of prothorax at about 90 degrees in lateral view, not serrate; bioluminescent spots absent; hind angle carinae present (single) or absent; posterior edge of pronotum with sublateral plicae and notches present or absent, crenellations absent; hypomeron posterior edge near each hind angle with or without concavity, concavity arcuate or angulate; pronotosternal sutures closed, hypomeral beads present. Prosternum with sides straight or concave at midlength in ventral view; prosternal process curved or not curved upward more than 40 degrees in lateral view. **Mesothorax.** Mesocoxal cavity open; mesoventral cavity without serration along sides. Elytra. Striae present; anterior edge outline straight to arcuate near humeri in dorsal view; integument marked with spots or transverse bands or unmarked with spots or tranverse bands; without pattern from differences in setal colour; setal vestiture even and mainly parallel. **Legs.** Metacoxal plate without elongation in mesal half, plate reaching lateral edge; tarsal pads or membranous lobes absent; tarsal claws without setae, simple. **Ventrites.** Microserration at sides (e.g. 100 points per mm) absent, ventrite 5 apex arcuate without paired setal brushes. **Aedeagus.** Parameres with articulation at base, apical lateral expansions present; parameres without setae, each paramere

with one seta, each paramere with two setae or each paramere with three or more setae. **Geography.** Known from Yukon Territory, British Columbia, Ontario, Quebec, New Brunswick+ (NB, NS, PE), Oregon+ (WA, OR, ID), Montana+ (MT, WY, ND), Michigan+ (MN, WI, MI), Vermont+ (ME, VT, NH) and New York ['+' refers to several states or provinces collectively].

9.2 METANOMUS Buysson, 1887

Habitus. Body Length 5-10 mm or 10-15 mm. Vestiture. Scale like setae absent. **Head.** Supra-antennal carinae fading on frons (not reaching another structure); prognathous (labrum oriented less than 90 degrees downward); frons without triangular depression. Antennae with 11 antennomeres, not pectinate, sensory elements beginning on antennomere IV. **Prothorax.** Pronotum wider than long at widest point (including hind angles); dorsal punctures uniform sized, all simple (floor of puncture concave), without tubercles or longitudinal carinae between punctures; pronotal lateral carina complete anteriorly, carina visible throughout length in dorsal view, meeting anterior edge of prothorax at about 90 degrees in lateral view, not serrate; bioluminescent spots absent; hind angle carinae present (single); posterior edge of pronotum with sublateral plicae and notches present, crenellations absent; hypomeron posterior edge near each hind angle with concavity, concavity arcuate; pronotosternal sutures closed, hypomeral beads present or absent. Prosternum with sides straight or concave at midlength in ventral view; prosternal process curved or not curved upward more than 40 degrees in lateral view. **Mesothorax.** Mesocoxal cavity open; mesoventral cavity without serration along sides. Elytra. Striae present; anterior edge outline straight to arcuate near humeri in dorsal view; integument unmarked with spots or tranverse bands; without pattern from differences in setal colour; setal vestiture even and mainly parallel. **Legs.** Metacoxal plate without elongation in mesal half, plate reaching lateral edge; tarsal pads or membranous lobes absent; tarsal claws without setae, simple. **Ventrites.** Microserration at sides (e.g. 100 points per mm) absent, ventrite 5 apex arcuate without paired setal brushes. **Aedeagus.** Parameres with articulation at base, apical lateral expansions present; each paramere with three or more setae. **Geography.** Known from British Columbia, Alberta, Manitoba+ (MB, SK), Ontario, Quebec, New Brunswick+ (NB, NS, PE), Montana+ (MT, WY, ND), Vermont+ (ME, VT, NH), New York, Pennsylvania+ (PA, WV) and Carolinas+ (KY, TN, NC, SC, GA, AL) ['+' refers to several states or provinces collectively].

9.2 NEOPRISTILOPHUS Buysson, 1894

Habitus. Body Length 10-15 mm, 15-20 mm or 20-40 mm. Vestiture. Scale like setae absent. **Head.** Supra-antennal carinae fading on frons (not reaching another structure) or directed anteriorly (reaching anterior part of head capsule); prognathous (labrum oriented less than 90 degrees downward); frons without triangular depression. Antennae with 11 antennomeres, not pectinate, sensory elements beginning on antennomere III or IV. **Prothorax.** Pronotum wider than long or longer than wide at widest point (including hind angles); dorsal punctures uniform sized, some or all punctures umbilicate (floor of puncture flat), without tubercles or longitudinal carinae between punctures; pronotal lateral carina complete anteriorly, carina visible throughout length in dorsal view, meeting anterior edge of prothorax at about 90 degrees in lateral view, not serrate; bioluminescent spots absent; hind angle carinae present (single); posterior edge of pronotum with sublateral plicae and notches present or absent (by misinterpretation), crenellations absent; hypomeron posterior edge near each hind angle with concavity, concavity arcuate; pronotosternal sutures closed, hypomeral beads absent. Prosternum with sides straight at midlength in ventral view; prosternal process not curved upward more than 40 degrees in lateral view. **Mesothorax.** Mesocoxal cavity open; mesoventral cavity without serration along sides. Elytra. Striae present; anterior edge outline straight to arcuate near humeri in

dorsal view; integument unmarked with spots or tranverse bands; without pattern from differences in setal colour; setal vestiture mainly absent on disk (by misinterpretation) or even and mainly parallel. **Legs.** Metacoxal plate without elongation in mesal half, plate reaching lateral edge; tarsal pads or membranous lobes absent; tarsal claws without setae, simple. **Ventrites.** Microserration at sides (e.g. 100 points per mm) absent, ventrite 5 apex arcuate without paired setal brushes. **Aedeagus.** Parameres with articulation at base, apical lateral expansions absent; parameres without setae. **Geography.** Known from British Columbia, New Brunswick+ (NB, NS, PE), Oregon+ (WA, OR, ID), California, Colorado+ (NV, UT, CO), Indiana+ (IL, IN, OH), Massachusetts+ (MA, CT, RI), Pennsylvania+ (PA, WV), Virginia+ (NJ, DE, MD, DC, VA) and Carolinas+ (KY, TN, NC, SC, GA, AL) ['+' refers to several states or provinces collectively].

9.2 NITIDOLIMONIUS Johnson, 2008

Habitus. Body Length 5-10 mm or 10-15 mm. Vestiture. Scale like setae absent. **Head.** Supra-antennal carinae joining medially (forming shelf), fading on frons (not reaching another structure) or directed anteriorly (reaching anterior part of head capsule); nasale (head capsule below edge of frontal carina) with outline not concave in lateral view; prognathous (labrum oriented less than 90 degrees downward); frons without triangular depression. Antennae with 11 antennomeres, not pectinate, sensory elements beginning on antennomere IV. **Prothorax.** Pronotum wider than long at widest point (including hind angles); dorsal punctures uniform sized, all simple (floor of puncture concave) or some or all punctures umbilicate (floor of puncture flat), without tubercles or longitudinal carinae between punctures; pronotal lateral carina complete anteriorly, carina visible throughout length in dorsal view, meeting anterior edge of prothorax at about 90 degrees in lateral view, not serrate; bioluminescent spots absent; hind angle carinae present (single); posterior edge of pronotum with sublateral plicae and notches present, crenellations absent; hypomeron posterior edge near each hind angle with concavity, concavity arcuate; pronotosternal sutures open, hypomeral beads present. Prosternum with sides straight at midlength in ventral view; prosternal process curved or not curved upward more than 40 degrees in lateral view. **Mesothorax.** Mesocoxal cavity open; mesoventral cavity without serration along sides. Elytra. Striae present; anterior edge outline straight to arcuate near humeri in dorsal view; integument with metallic reflections or unmarked with spots or tranverse bands; without pattern from differences in setal colour; setal vestiture mainly absent on disk or even and mainly parallel. **Legs.** Metacoxal plate without elongation in mesal half, plate reaching lateral edge; tarsal pads or membranous lobes absent; tarsal claws without setae, simple. **Ventrites.** Microserration at sides (e.g. 100 points per mm) absent, ventrite 5 apex arcuate without paired setal brushes. **Aedeagus.** Parameres with articulation at base, apical lateral expansions present; parameres without setae or each paramere with three or more setae. **Geography.** Known from Nunavut+ (NT, NU), British Columbia, Alberta, Manitoba+ (MB, SK), Ontario, Quebec, New Brunswick+ (NB, NS, PE), Newfoundland and Labrador, Alaska, Oregon+ (WA, OR, ID), California, Montana+ (MT, WY, ND), Colorado+ (NV, UT, CO), Vermont+ (ME, VT, NH), New York, Pennsylvania+ (PA, WV) and Carolinas+ (KY, TN, NC, SC, GA, AL) ['+' refers to several states or provinces collectively].

9.2 OXYGONUS LeConte, 1863

Habitus. Body Length 1-5 mm, 5-10 mm or 10-15 mm. Vestiture. Scale like setae absent. **Head.** Supra-antennal carinae fading on frons (not reaching another structure) or directed anteriorly (reaching anterior part of head capsule); hypognathous (labrum oriented downward 90 degrees or more) or prognathous (labrum oriented less than 90 degrees downward); frons without triangular depression. Antennae with 11 antennomeres, not pectinate, sensory elements beginning on antennomere IV. **Prothorax.** Pronotum wider than long at widest point (including hind angles); dorsal punctures

uniform sized, all simple (floor of puncture concave) or some or all punctures umbilicate (floor of puncture flat), without tubercles or longitudinal carinae between punctures; pronotal lateral carina complete anteriorly, carina visible throughout length in dorsal view, meeting anterior edge of prothorax at about 90 degrees in lateral view, not serrate; bioluminescent spots absent; hind angle carinae present (single) or absent; posterior edge of pronotum with sublateral plicae and notches present or absent, crenellations absent; hypomeron posterior edge near each hind angle with concavity, concavity arcuate; pronotosternal sutures closed, hypomeral beads present. Prosternum with sides straight at midlength in ventral view; prosternal process not curved upward more than 40 degrees in lateral view. **Mesothorax.** Mesocoxal cavity open; mesoventral cavity without serration along sides. Elytra. Striae present; anterior edge outline straight to arcuate near humeri in dorsal view; integument unmarked with spots or tranverse bands; without pattern from differences in setal colour; setal vestiture even and mainly parallel or partially transverse in patches. **Legs.** Metacoxal plate without elongation in mesal half, plate reaching lateral edge; tarsal pads or membranous lobes absent; tarsal claws without setae, with two points, or appendiculate. **Ventrites.** Microserration at sides (e.g. 100 points per mm) absent, ventrite 5 apex arcuate without paired setal brushes. **Aedeagus.** Parameres with articulation at base, apical lateral expansions present; parameres without setae or each paramere with three or more setae. **Geography.** Known from Alberta, Manitoba+ (MB, SK), Ontario, Quebec, New Brunswick+ (NB, NS, PE), Oregon+ (WA, OR, ID), California, Montana+ (MT, WY, ND), Nebraska+ (SD, NE, IA), Michigan+ (MN, WI, MI), Indiana+ (IL, IN, OH), Vermont+ (ME, VT, NH), Massachusetts+ (MA, CT, RI), New York, Pennsylvania+ (PA, WV) and Carolinas+ (KY, TN, NC, SC, GA, AL) ['+' refers to several states or provinces collectively].

9.2 PARACTENICERA Johnson, 2008

Habitus. Body Length 10-15 mm or 15-20 mm. Vestiture. Scale like setae absent. **Head.** Supra-antennal carinae fading on frons (not reaching another structure); prognathous (labrum oriented less than 90 degrees downward); frons without triangular depression. Antennae with 11 antennomeres, not pectinate, sensory elements beginning on antennomere III. **Prothorax.** Pronotum longer than wide at widest point (including hind angles); dorsal punctures uniform sized, some or all punctures umbilicate (floor of puncture flat), without tubercles or longitudinal carinae between punctures; pronotal lateral carina complete anteriorly, carina visible throughout length in dorsal view, meeting anterior edge of prothorax at about 90 degrees in lateral view, not serrate; bioluminescent spots absent; hind angle carinae present (single); posterior edge of pronotum with sublateral plicae and notches present, crenellations absent; hypomeron posterior edge near each hind angle with concavity, concavity arcuate; pronotosternal sutures closed, hypomeral beads absent. Prosternum with sides straight at midlength in ventral view; prosternal process not curved upward more than 40 degrees in lateral view. **Mesothorax.** Mesocoxal cavity open; mesoventral cavity without serration along sides. Elytra. Striae present; anterior edge outline straight to arcuate near humeri in dorsal view; integument unmarked with spots or tranverse bands; without pattern from differences in setal colour; setal vestiture even and mainly parallel. **Legs.** Metacoxal plate without elongation in mesal half, plate reaching lateral edge; tarsal pads or membranous lobes absent; tarsal claws without setae, simple. **Ventrites.** Microserration at sides (e.g. 100 points per mm) absent, ventrite 5 apex arcuate without paired setal brushes. **Aedeagus.** Parameres with articulation at base, apical lateral expansions absent; each paramere with two setae. **Geography.** Known from Alberta, Ontario, Quebec, New Brunswick+ (NB, NS, PE), New York, Pennsylvania+ (PA, WV) and Virginia+ (NJ, DE, MD, DC, VA) ['+' refers to several states or provinces collectively].

9.2 PROLUDIUS Lane, 1971 (P, iaculus (LeConte), also P. anthrax, P. protractus, P. pyrros, P. tenellus)

Habitus. Body Length 10-15 mm or 15-20 mm. Vestiture. Scale like setae absent. **Head.** Supra-antennal carinae joining medially (forming shelf), fading on frons (not reaching another structure) or directed anteriorly (reaching anterior part of head capsule); nasale (head capsule below edge of frontal carina) with outline not concave in lateral view; prognathous (labrum oriented less than 90 degrees downward); frons without triangular depression. Antennae with 11 antennomeres, not pectinate, sensory elements beginning on antennomere III. **Prothorax.** Pronotum wider than long or longer than wide at widest point (including hind angles); dorsal punctures uniform sized, all simple (floor of puncture concave) or some or all punctures umbilicate (floor of puncture flat), without tubercles or longitudinal carinae between punctures; pronotal lateral carina complete anteriorly, carina visible throughout length in dorsal view, meeting anterior edge of prothorax at about 90 degrees in lateral view, not serrate; bioluminescent spots absent; hind angle carinae present (single); posterior edge of pronotum with sublateral plicae and notches present or absent, crenellations absent; hypomeron posterior edge near each hind angle with concavity, concavity arcuate or angulate; pronotosternal sutures closed, hypomeral beads present or absent. Prosternum with sides straight at midlength in ventral view; prosternal process not curved upward more than 40 degrees in lateral view. **Mesothorax.** Mesocoxal cavity open; mesoventral cavity without serration along sides. Elytra. Striae present; anterior edge outline straight to arcuate near humeri in dorsal view; integument unmarked with spots or tranverse bands; without pattern from differences in setal colour; setal vestiture even and mainly parallel. **Legs.** Metacoxal plate without elongation in mesal half, plate reaching lateral edge; tarsal pads or membranous lobes absent; tarsal claws without setae, simple. **Ventrites.** Microserration at sides (e.g. 100 points per mm) absent, ventrite 5 apex arcuate without paired setal brushes. **Aedeagus.** Parameres with articulation at base, apical lateral expansions present; parameres without setae, each paramere with one seta, each paramere with two setae or each paramere with three or more setae. **Geography.** Known from British Columbia, Ontario, Alaska, Oregon+ (WA, OR, ID), California, Montana+ (MT, WY, ND), Indiana+ (IL, IN, OH), Massachusetts+ (MA, CT, RI), New York, Pennsylvania+ (PA, WV), Virginia+ (NJ, DE, MD, DC, VA), Carolinas+ (KY, TN, NC, SC, GA, AL) and Florida ['+' refers to several states or provinces collectively].

P. angusticollis, P. captiosus, P. comes

Habitus. Body Length 10-15 mm or 15-20 mm. Vestiture. Scale like setae absent. **Head.** Supra-antennal carinae fading on frons (not reaching another structure); prognathous (labrum oriented less than 90 degrees downward); frons without triangular depression. Antennae with 11 antennomeres, not pectinate, sensory elements beginning on antennomere IV. **Prothorax.** Pronotum wider than long or longer than wide at widest point (including hind angles); dorsal punctures uniform sized, all simple (floor of puncture concave) or some or all punctures umbilicate (floor of puncture flat), without tubercles or longitudinal carinae between punctures; pronotal lateral carina complete anteriorly, carina visible throughout length in dorsal view, meeting anterior edge of prothorax at about 90 degrees in lateral view, not serrate; bioluminescent spots absent; hind angle carinae present (single); posterior edge of pronotum with sublateral plicae and notches present, crenellations absent; hypomeron posterior edge near each hind angle with concavity, concavity arcuate; pronotosternal sutures closed, hypomeral beads present or absent. Prosternum with sides straight at midlength in ventral view; prosternal process not curved upward more than 40 degrees in lateral view. **Mesothorax.** Mesocoxal cavity open; mesoventral cavity without serration along sides. Elytra. Striae present; anterior edge outline straight to arcuate near humeri in dorsal view; integument unmarked with spots or tranverse bands; without pattern from differences in setal colour; setal vestiture even and mainly parallel. **Legs.** Metacoxal plate without elongation in mesal half, plate reaching lateral edge; tarsal pads or membranous lobes absent; tarsal claws without setae, simple. **Ventrites.** Microserration at sides (e.g. 100 points per mm) absent, ventrite 5 apex arcuate without paired setal brushes. **Aedeagus.** Parameres with articulation at base, apical

lateral expansions present; parameres without setae. **Geography.** Known from British Columbia, Alaska and Oregon+ (WA, OR, ID) ['+' refers to several states or provinces collectively].

P. sylvaticus (Van Dyke, 1932)

Habitus. Body Length 10-15 mm or 15-20 mm. Vestiture. Scale like setae absent. **Head.** Supra-antennal carinae fading on frons (not reaching another structure); prognathous (labrum oriented less than 90 degrees downward); frons without triangular depression. Antennae with 11 antennomeres, not pectinate, sensory elements beginning on antennomere III. **Prothorax.** Pronotum longer than wide at widest point (including hind angles); dorsal punctures uniform sized, some or all punctures umbilicate (floor of puncture flat), without tubercles or longitudinal carinae between punctures; pronotal lateral carina complete anteriorly, carina visible throughout length in dorsal view, meeting anterior edge of prothorax at about 90 degrees in lateral view, not serrate; bioluminescent spots absent; hind angle carinae present (single); posterior edge of pronotum with sublateral plicae and notches present, crenellations absent; hypomeron posterior edge near each hind angle with concavity, concavity arcuate; pronotosternal sutures closed, hypomeral beads absent. Prosternum with sides straight at midlength in ventral view; prosternal process not curved upward more than 40 degrees in lateral view. **Mesothorax.** Mesocoxal cavity open; mesoventral cavity without serration along sides. Elytra. Striae present; anterior edge outline straight to arcuate near humeri in dorsal view; integument unmarked with spots or tranverse bands; without pattern from differences in setal colour; setal vestiture even and mainly parallel. **Legs.** Metacoxal plate without elongation in mesal half, plate reaching lateral edge; tarsal pads or membranous lobes absent; tarsal claws without setae, simple. **Ventrites.** Microserration at sides (e.g. 100 points per mm) absent, ventrite 5 apex arcuate without paired setal brushes. **Aedeagus.** Parameres with articulation at base, apical lateral expansions present; parameres without setae. **Geography.** Known from British Columbia, Alberta, Oregon+ (WA, OR, ID), California and Montana+ (MT, WY, ND) ['+' refers to several states or provinces collectively].

9.2 PROSTERNON Latreille, 1834

Habitus. Body Length 5-10 mm or 10-15 mm. Vestiture. Scale like setae absent. **Head.** Supra-antennal carinae fading on frons (not reaching another structure); prognathous (labrum oriented less than 90 degrees downward); frons without triangular depression. Antennae with 11 antennomeres, not pectinate, sensory elements beginning on antennomere IV. **Prothorax.** Pronotum wider than long at widest point (including hind angles); dorsal punctures uniform sized, all simple (floor of puncture concave) or some or all punctures umbilicate (floor of puncture flat), without tubercles or longitudinal carinae between punctures; pronotal lateral carina complete anteriorly, carina visible throughout length in dorsal view, meeting anterior edge of prothorax at about 90 degrees in lateral view, not serrate; bioluminescent spots absent; hind angle carinae present (single); posterior edge of pronotum with sublateral plicae and notches present or absent (by misinterpretation), crenellations absent; hypomeron posterior edge near each hind angle without concavity; pronotosternal sutures closed, hypomeral beads present. Prosternum with sides straight at midlength in ventral view; prosternal process not curved upward more than 40 degrees in lateral view. **Mesothorax.** Mesocoxal cavity open; mesoventral cavity without serration along sides. Elytra. Striae present; anterior edge outline straight to arcuate near humeri in dorsal view; integument unmarked with spots or tranverse bands; without pattern from differences in setal colour; setal vestiture partially transverse in patches. **Legs.** Metacoxal plate without elongation in mesal half, plate reaching lateral edge; tarsal pads or membranous lobes absent; tarsal claws without setae, simple. **Ventrites.** Microserration at sides (e.g. 100 points per mm) present, ventrite 5 apex arcuate without paired setal brushes. **Aedeagus.** Parameres with articulation at base,

apical lateral expansions present; parameres without setae, each paramere with two setae or each paramere with three or more setae. **Geography.** Known from Nunavut+ (NT, NU), British Columbia, Alberta, Manitoba+ (MB, SK), Ontario, Quebec, New Brunswick+ (NB, NS, PE), Oregon+ (WA, OR, ID), California, Montana+ (MT, WY, ND), Colorado+ (NV, UT, CO), Nebraska+ (SD, NE, IA), Michigan+ (MN, WI, MI), Vermont+ (ME, VT, NH) and Pennsylvania+ (PA, WV) ['+' refers to several states or provinces collectively].

9.2 PSEUDANOSTIRUS Dolin, 1964

P. hoppingi, P. ochreipennis, P. watsoni

Habitus. Body Length 5-10 mm or 10-15 mm. Vestiture. Scale like setae absent. **Head.** Supra-antennal carinae fading on frons (not reaching another structure); hypognathous (labrum oriented downward 90 degrees or more) or prognathous (labrum oriented less than 90 degrees downward); frons without triangular depression. Antennae with 11 antennomeres, not pectinate, sensory elements beginning on antennomere IV. **Prothorax.** Pronotum wider than long at widest point (including hind angles); dorsal punctures uniform sized, all simple (floor of puncture concave), without tubercles or longitudinal carinae between punctures; pronotal lateral carina complete anteriorly, carina visible throughout length in dorsal view, meeting anterior edge of prothorax at about 90 degrees in lateral view, not serrate; bioluminescent spots absent; hind angle carinae present (single) or absent; posterior edge of pronotum with sublateral plicae and notches present, crenellations absent; hypomeron posterior edge near each hind angle with concavity, concavity arcuate; pronotosternal sutures closed, hypomeral beads present or absent. Prosternum with sides straight at midlength in ventral view; prosternal process not curved upward more than 40 degrees in lateral view. **Mesothorax.** Mesocoxal cavity open; mesoventral cavity without serration along sides. Elytra. Striae present; anterior edge outline straight to arcuate near humeri in dorsal view; integument unmarked with spots or tranverse bands; without pattern from differences in setal colour; setal vestiture even and mainly parallel. **Legs.** Metacoxal plate without elongation in mesal half, plate reaching lateral edge; tarsal pads or membranous lobes absent; tarsal claws without setae, simple. **Ventrites.** Microserration at sides (e.g. 100 points per mm) present, ventrite 5 apex arcuate without paired setal brushes. **Aedeagus.** Parameres with articulation at base, apical lateral expansions absent; each paramere with two setae or each paramere with three or more setae. **Geography.** Known from Nunavut+ (NT, NU), Yukon Territory, British Columbia, Alberta, Manitoba+ (MB, SK), Ontario, Quebec, Newfoundland and Labrador, Alaska, Oregon+ (WA, OR, ID), California and Montana+ (MT, WY, ND) ['+' refers to several states or provinces collectively].

P. laricis (Brown)

Habitus. Body Length 5-10 mm. Vestiture. Scale like setae absent. **Head.** Supra-antennal carinae fading on frons (not reaching another structure); prognathous (labrum oriented less than 90 degrees downward); frons without triangular depression. Antennae with 11 antennomeres, not pectinate, sensory elements beginning on antennomere IV. **Prothorax.** Pronotum wider than long at widest point (including hind angles); dorsal punctures uniform sized, all simple (floor of puncture concave), without tubercles or longitudinal carinae between punctures; pronotal lateral carina complete anteriorly, carina visible throughout length in dorsal view, meeting anterior edge of prothorax at about 90 degrees in lateral view, not serrate; bioluminescent spots absent; hind angle carinae present (single) or absent; posterior edge of pronotum with sublateral plicae and notches present, crenellations absent; hypomeron posterior edge near each hind angle with concavity, concavity arcuate; pronotosternal sutures closed, hypomeral beads absent. Prosternum with sides straight at midlength in ventral view;

prosternal process not curved upward more than 40 degrees in lateral view. **Mesothorax.** Mesocoxal cavity open; mesoventral cavity without serration along sides. Elytra. Striae present; anterior edge outline straight to arcuate near humeri in dorsal view; integument unmarked with spots or tranverse bands; without pattern from differences in setal colour; setal vestiture even and mainly parallel. **Legs.** Metacoxal plate without elongation in mesal half, plate reaching lateral edge; tarsal pads or membranous lobes absent; tarsal claws without setae, simple. **Ventrites.** Microserration at sides (e.g. 100 points per mm) absent, ventrite 5 apex arcuate without paired setal brushes. **Aedeagus.** Parameres with articulation at base, apical lateral expansions present; each paramere with three or more setae. **Geography.** Known from British Columbia, Alberta and Montana+ (MT, WY, ND) ['+' refers to several states or provinces collectively].

P. nebraskensis, P. tigrinus, P. triundulatus

Habitus. Body Length 5-10 mm or 10-15 mm. Vestiture. Scale like setae absent. **Head.** Supra-antennal carinae fading on frons (not reaching another structure); prognathous (labrum oriented less than 90 degrees downward); frons without triangular depression. Antennae with 11 antennomeres, sensory elements beginning on antennomere IV. **Prothorax.** Pronotum wider than long at widest point (including hind angles); dorsal punctures uniform sized, all simple (floor of puncture concave), without tubercles or longitudinal carinae between punctures; pronotal lateral carina complete anteriorly, carina visible throughout length in dorsal view, meeting anterior edge of prothorax at about 90 degrees in lateral view, not serrate; bioluminescent spots absent; hind angle carinae absent; posterior edge of pronotum with sublateral plicae and notches absent, crenellations absent; hypomeron posterior edge near each hind angle without concavity; pronotosternal sutures closed, hypomeral beads present. Prosternum with sides straight at midlength in ventral view; prosternal process not curved upward more than 40 degrees in lateral view. **Mesothorax.** Mesocoxal cavity open; mesoventral cavity without serration along sides. Elytra. Striae present; anterior edge outline straight to arcuate near humeri in dorsal view; integument unmarked with spots or tranverse bands; with pattern from differences in setal colour; setal vestiture even and mainly parallel. **Legs.** Metacoxal plate without elongation in mesal half, plate reaching lateral edge; tarsal pads or membranous lobes absent; tarsal claws without setae, simple. **Ventrites.** Microserration at sides (e.g. 100 points per mm) absent, ventrite 5 apex arcuate without paired setal brushes. **Aedeagus.** Parameres with articulation at base, apical lateral expansions absent; parameres without setae or each paramere with one seta. **Geography.** Known from Nunavut+ (NT, NU), British Columbia, Alberta, Manitoba+ (MB, SK), Ontario, Quebec, New Brunswick+ (NB, NS, PE), Newfoundland and Labrador, Alaska, Oregon+ (WA, OR, ID), California, Montana+ (MT, WY, ND), Colorado+ (NV, UT, CO), Arizona+ (AZ, NM), Nebraska+ (SD, NE, IA), Michigan+ (MN, WI, MI), Vermont+ (ME, VT, NH), Massachusetts+ (MA, CT, RI), New York and Pennsylvania+ (PA, WV) ['+' refers to several states or provinces collectively].

9.2 SELATOSOMUS Stephens, 1830

Habitus. Body Length 5-10 mm, 10-15 mm or 15-20 mm. Vestiture. Scale like setae absent. **Head.** Supra-antennal carinae fading on frons (not reaching another structure); prognathous (labrum oriented less than 90 degrees downward); frons without triangular depression. Antennae with 11 antennomeres, not pectinate, sensory elements beginning on antennomere IV. **Prothorax.** Pronotum wider than long at widest point (including hind angles); dorsal punctures uniform sized, all simple (floor of puncture concave) or some or all punctures umbilicate (floor of puncture flat), without tubercles or longitudinal carinae between punctures; pronotal lateral carina complete anteriorly, carina visible throughout length in dorsal view, meeting anterior edge of prothorax at about 90 degrees in lateral view, not serrate;

bioluminescent spots absent; hind angle carinae present (single); posterior edge of pronotum with sublateral plicae and notches present, crenellations absent; hypomeron posterior edge near each hind angle with concavity, concavity arcuate; pronotosternal sutures closed, hypomeral beads present or absent. Prosternum with sides straight or concave at midlength in ventral view; prosternal process not curved upward more than 40 degrees in lateral view. **Mesothorax.** Mesocoxal cavity open; mesoventral cavity without serration along sides. Elytra. Striae present; anterior edge outline straight to arcuate near humeri in dorsal view; integument marked with spots or transverse bands, with metallic reflections or unmarked with spots or tranverse bands; without pattern from differences in setal colour; setal vestiture mainly absent on disk. **Legs.** Metacoxal plate without elongation in mesal half, plate reaching or not reaching lateral edge; tarsal pads or membranous lobes absent; tarsal claws without setae, simple. **Ventrites.** Microserration at sides (e.g. 100 points per mm) absent, ventrite 5 apex arcuate without paired setal brushes. **Aedeagus.** Parameres with articulation at base, apical lateral expansions present; parameres without setae, each paramere with two setae or each paramere with three or more setae. **Geography.** Known from Nunavut+ (NT, NU), Yukon Territory, British Columbia, Alberta, Manitoba+ (MB, SK), Ontario, Quebec, New Brunswick+ (NB, NS, PE), Alaska, Oregon+ (WA, OR, ID), California, Montana+ (MT, WY, ND), Colorado+ (NV, UT, CO), Arizona+ (AZ, NM), Nebraska+ (SD, NE, IA), Michigan+ (MN, WI, MI), Vermont+ (ME, VT, NH), Massachusetts+ (MA, CT, RI), New York and Pennsylvania+ (PA, WV) ['+' refers to several states or provinces collectively].

S. nigricans (Fall)

Habitus. Body Length 5-10 mm or 10-15 mm. Vestiture. Scale like setae absent. **Head.** Supra-antennal carinae fading on frons (not reaching another structure); prognathous (labrum oriented less than 90 degrees downward); frons without triangular depression. Antennae with 11 antennomeres, not pectinate, sensory elements beginning on antennomere IV. **Prothorax.** Pronotum wider than long at widest point (including hind angles); dorsal punctures uniform sized, all simple (floor of puncture concave), without tubercles or longitudinal carinae between punctures; pronotal lateral carina complete anteriorly, carina visible throughout length in dorsal view, meeting anterior edge of prothorax at about 90 degrees in lateral view, not serrate; bioluminescent spots absent; hind angle carinae present (single); posterior edge of pronotum with sublateral plicae and notches absent, crenellations absent; hypomeron posterior edge near each hind angle with concavity, concavity arcuate; pronotosternal sutures closed, hypomeral beads present. Prosternum with sides straight or convex at midlength in ventral view; prosternal process not curved upward more than 40 degrees in lateral view. **Mesothorax.** Mesocoxal cavity open; mesoventral cavity without serration along sides. Elytra. Striae present; anterior edge outline straight to arcuate near humeri in dorsal view; integument unmarked with spots or tranverse bands; without pattern from differences in setal colour; setal vestiture mainly absent on disk or even and mainly parallel. **Legs.** Metacoxal plate without elongation in mesal half, plate reaching lateral edge; tarsal pads or membranous lobes absent; tarsal claws without setae, simple. **Ventrites.** Microserration at sides (e.g. 100 points per mm) absent, ventrite 5 apex arcuate without paired setal brushes. **Aedeagus.** Parameres with articulation at base, apical lateral expansions present; parameres without setae. **Geography.** Known from British Columbia, Oregon+ (WA, OR, ID) and California ['+' refers to several states or provinces collectively].

S. pruininus (Horn)

Habitus. Body Length 5-10 mm or 10-15 mm. Vestiture. Scale like setae absent. **Head.** Supra-antennal carinae fading on frons (not reaching another structure); prognathous (labrum oriented less than 90 degrees downward); frons without triangular depression. Antennae with 11 antennomeres, not

pectinate, sensory elements beginning on antennomere IV. **Prothorax.** Pronotum wider than long at widest point (including hind angles); dorsal punctures uniform sized, all simple (floor of puncture concave) or some or all punctures umbilicate (floor of puncture flat), without tubercles or longitudinal carinae between punctures; pronotal lateral carina complete anteriorly, carina visible throughout length in dorsal view, meeting anterior edge of prothorax at about 90 degrees in lateral view, not serrate; bioluminescent spots absent; hind angle carinae present (single); posterior edge of pronotum with sublateral plicae and notches present, crenellations absent; hypomeron posterior edge near each hind angle with concavity, concavity arcuate; pronotosternal sutures closed, hypomeral beads present. Prosternum with sides straight or concave at midlength in ventral view; prosternal process not curved upward more than 40 degrees in lateral view. **Mesothorax.** Mesocoxal cavity open; mesoventral cavity without serration along sides. Elytra. Striae present; anterior edge outline straight to arcuate near humeri in dorsal view; integument unmarked with spots or tranverse bands; without pattern from differences in setal colour; setal vestiture even and mainly parallel. **Legs.** Metacoxal plate without elongation in mesal half, plate reaching lateral edge; tarsal pads or membranous lobes absent; tarsal claws without setae, simple. **Ventrites.** Microserration at sides (e.g. 100 points per mm) absent, ventrite 5 apex arcuate without paired setal brushes. **Aedeagus.** Parameres with articulation at base, apical lateral expansions present; each paramere with three or more setae. **Geography.** Known from British Columbia, Oregon+ (WA, OR, ID), California, Montana+ (MT, WY, ND), Colorado+ (NV, UT, CO) and Nebraska+ (SD, NE, IA) ['+' refers to several states or provinces collectively].

9.2 SETASOMUS Gurjeva, 1985

Habitus. Body Length 5-10 mm or 10-15 mm. Vestiture. Scale like setae absent. **Head.** Supra-antennal carinae joining medially (forming shelf) or fading on frons (not reaching another structure); nasale (head capsule below edge of frontal carina) with outline not concave in lateral view; prognathous (labrum oriented less than 90 degrees downward); frons without triangular depression. Antennae with 11 antennomeres, not pectinate, sensory elements beginning on antennomere IV. **Prothorax.** Pronotum wider than long at widest point (including hind angles); dorsal punctures uniform sized, all simple (floor of puncture concave), without tubercles or longitudinal carinae between punctures; pronotal lateral carina complete anteriorly, carina visible throughout length in dorsal view, meeting anterior edge of prothorax at about 90 degrees in lateral view, not serrate; bioluminescent spots absent; hind angle carinae present (single); posterior edge of pronotum with sublateral plicae and notches present, crenellations absent; hypomeron posterior edge near each hind angle with concavity, concavity arcuate; pronotosternal sutures closed, hypomeral beads present. Prosternum with sides straight at midlength in ventral view; prosternal process not curved upward more than 40 degrees in lateral view. **Mesothorax.** Mesocoxal cavity open or open to mesepimeron only; mesoventral cavity without serration along sides. Elytra. Striae present; anterior edge outline straight to arcuate near humeri in dorsal view; integument marked with spots or transverse bands or unmarked with spots or tranverse bands; without pattern from differences in setal colour; setal vestiture even and mainly parallel. **Legs.** Metacoxal plate without elongation in mesal half, plate reaching lateral edge; tarsal pads or membranous lobes absent; tarsal claws without setae, simple. **Ventrites.** Microserration at sides (e.g. 100 points per mm) absent, ventrite 5 apex arcuate without paired setal brushes. **Aedeagus.** Parameres with articulation at base, apical lateral expansions present or absent; parameres without setae or each paramere with three or more setae. **Geography.** Known from Nunavut+ (NT, NU), Yukon Territory, British Columbia, Alberta, Manitoba+ (MB, SK), Ontario, Quebec, New Brunswick+ (NB, NS, PE), Newfoundland and Labrador, Alaska and Vermont+ (ME, VT, NH) ['+' refers to several states or provinces collectively].

9.2 STROPENRON Johnson, 2021

Habitus. Body Length 5-10 mm or 10-15 mm. Vestiture. Scale like setae absent. **Head.** Supra-antennal carinae fading on frons (not reaching another structure); hypognathous (labrum oriented downward 90 degrees or more) or prognathous (labrum oriented less than 90 degrees downward); frons without triangular depression. Antennae with 11 antennomeres, not pectinate, sensory elements beginning on antennomere IV. **Prothorax.** Pronotum wider than long at widest point (including hind angles); dorsal punctures uniform sized, all simple (floor of puncture concave), without tubercles or longitudinal carinae between punctures; pronotal lateral carina complete anteriorly, carina visible throughout length in dorsal view, meeting anterior edge of prothorax at about 90 degrees in lateral view, not serrate; bioluminescent spots absent; hind angle carinae present (single) or absent; posterior edge of pronotum with sublateral plicae and notches present, crenellations absent; hypomeron posterior edge near each hind angle with concavity, concavity arcuate; pronotosternal sutures closed, hypomeral beads present or absent. Prosternum with sides straight at midlength in ventral view; prosternal process not curved upward more than 40 degrees in lateral view. **Mesothorax.** Mesocoxal cavity open; mesoventral cavity without serration along sides. Elytra. Striae present; anterior edge outline straight to arcuate near humeri in dorsal view; integument marked with spots or transverse bands or marked with spot or band in apical 2/5 only; without pattern from differences in setal colour; setal vestiture even and mainly parallel. **Legs.** Metacoxal plate without elongation in mesal half, plate reaching lateral edge; tarsal pads or membranous lobes absent; tarsal claws without setae, simple. **Ventrites.** Microserration at sides (e.g. 100 points per mm) present, ventrite 5 apex arcuate without paired setal brushes. **Aedeagus.** Parameres with articulation at base, apical lateral expansions present or absent; each paramere with one seta, each paramere with two setae or each paramere with three or more setae. **Geography.** Known from Nunavut+ (NT, NU), Yukon Territory, British Columbia, Alberta, Manitoba+ (MB, SK), Ontario, Quebec, New Brunswick+ (NB, NS, PE), Alaska, Oregon+ (WA, OR, ID), California, Montana+ (MT, WY, ND), Colorado+ (NV, UT, CO), Nebraska+ (SD, NE, IA), Michigan+ (MN, WI, MI), Indiana+ (IL, IN, OH), Arkansas+ (KS, MO, OK, AR, LA, MS), Vermont+ (ME, VT, NH), Massachusetts+ (MA, CT, RI), New York, Pennsylvania+ (PA, WV), Virginia+ (NJ, DE, MD, DC, VA) and Carolinas+ (KY, TN, NC, SC, GA, AL) ['+' refers to several states or provinces collectively].

9.2 SYLVANELATER Johnson, 2008

Habitus. Body Length 5-10 mm, 10-15 mm or 15-20 mm. Vestiture. Scale like setae absent. **Head.** Supra-antennal carinae fading on frons (not reaching another structure); hypognathous (labrum oriented downward 90 degrees or more) or prognathous (labrum oriented less than 90 degrees downward); frons without triangular depression. Antennae with 11 antennomeres, not pectinate, sensory elements beginning on antennomere III. **Prothorax.** Pronotum wider than long at widest point (including hind angles); dorsal punctures uniform sized, all simple (floor of puncture concave), without tubercles or longitudinal carinae between punctures; pronotal lateral carina complete anteriorly, carina visible throughout length in dorsal view, meeting anterior edge of prothorax at about 90 degrees in lateral view, not serrate; bioluminescent spots absent; hind angle carinae present (single); posterior edge of pronotum with sublateral plicae and notches present, crenellations absent; hypomeron posterior edge near each hind angle with concavity, concavity arcuate or angulate; pronotosternal sutures closed, hypomeral beads present or absent. Prosternum with sides straight at midlength in ventral view; prosternal process curved or not curved upward more than 40 degrees in lateral view. **Mesothorax.** Mesocoxal cavity open; mesoventral cavity without serration along sides. Elytra. Striae present; anterior edge outline straight to arcuate near humeri in dorsal view; integument unmarked with spots or tranverse bands; without pattern from differences in setal colour; setal

vestiture even and mainly parallel. **Legs.** Metacoxal plate without elongation in mesal half, plate reaching lateral edge; tarsal pads or membranous lobes absent; tarsal claws without setae, simple. **Ventrites.** Microserration at sides (e.g. 100 points per mm) absent, ventrite 5 apex arcuate without paired setal brushes. **Aedeagus.** Parameres with articulation at base, apical lateral expansions present; parameres without setae or each paramere with three or more setae. **Geography.** Known from Nunavut+ (NT, NU), Yukon Territory, British Columbia, Alberta, Manitoba+ (MB, SK), Ontario, Quebec, New Brunswick+ (NB, NS, PE), Newfoundland and Labrador, Alaska, Oregon+ (WA, OR, ID), California, Nebraska+ (SD, NE, IA), Michigan+ (MN, WI, MI), Arkansas+ (KS, MO, OK, AR, LA, MS), Vermont+ (ME, VT, NH), Massachusetts+ (MA, CT, RI), New York, Pennsylvania+ (PA, WV) and Virginia+ (NJ, DE, MD, DC, VA) ['+' refers to several states or provinces collectively].

S. atropurpureus (Melsheimer)

Habitus. Body Length 5-10 mm or 10-15 mm. Vestiture. Scale like setae absent. **Head.** Supra-antennal carinae fading on frons (not reaching another structure); hypognathous (labrum oriented downward 90 degrees or more); frons without triangular depression. Antennae with 11 antennomeres, not pectinate, sensory elements beginning on antennomere III. **Prothorax.** Pronotum wider than long at widest point (including hind angles); dorsal punctures uniform sized, all simple (floor of puncture concave), without tubercles or longitudinal carinae between punctures; pronotal lateral carina complete anteriorly, carina visible throughout length in dorsal view, meeting anterior edge of prothorax at about 90 degrees in lateral view, not serrate; bioluminescent spots absent; hind angle carinae present (single); posterior edge of pronotum with sublateral plicae and notches present, crenellations absent; hypomeron posterior edge near each hind angle with concavity, concavity arcuate; pronotosternal sutures open, hypomeral beads present. Prosternum with sides straight or concave at midlength in ventral view; prosternal process not curved upward more than 40 degrees in lateral view. **Mesothorax.** Mesocoxal cavity open; mesoventral cavity without serration along sides. Elytra. Striae present; anterior edge outline straight to arcuate near humeri in dorsal view; integument with metallic reflections or unmarked with spots or tranverse bands; without pattern from differences in setal colour; setal vestiture even and mainly parallel. **Legs.** Metacoxal plate without elongation in mesal half, plate reaching lateral edge; tarsal pads or membranous lobes absent; tarsal claws without setae, simple. **Ventrites.** Microserration at sides (e.g. 100 points per mm) absent, ventrite 5 apex arcuate without paired setal brushes. **Aedeagus.** Parameres with articulation at base, apical lateral expansions present; parameres without setae. **Geography.** Known from Quebec, New York and Pennsylvania+ (PA, WV) ['+' refers to several states or provinces collectively].

S. furtivus, S. limoniiformis, S. mendax

Habitus. Body Length 5-10 mm or 10-15 mm. Vestiture. Scale like setae absent. **Head.** Supra-antennal carinae joining medially (forming shelf) or fading on frons (not reaching another structure); nasale (head capsule below edge of frontal carina) with outline not concave in lateral view; hypognathous (labrum oriented downward 90 degrees or more) or prognathous (labrum oriented less than 90 degrees downward); frons without triangular depression. Antennae with 11 antennomeres, not pectinate, sensory elements beginning on antennomere IV. **Prothorax.** Pronotum wider than long at widest point (including hind angles); dorsal punctures uniform sized, all simple (floor of puncture concave), without tubercles or longitudinal carinae between punctures; pronotal lateral carina complete anteriorly, carina visible throughout length in dorsal view, meeting anterior edge of prothorax at about 90 degrees in lateral view, not serrate; bioluminescent spots absent; hind angle carinae present (single) or absent; posterior edge of pronotum with sublateral plicae and notches present, crenellations absent;

hypomeron posterior edge near each hind angle with concavity, concavity arcuate; pronotosternal sutures open, hypomeral beads present. Prosternum with sides straight or concave at midlength in ventral view; prosternal process not curved upward more than 40 degrees in lateral view. **Mesothorax.** Mesocoxal cavity open; mesoventral cavity without serration along sides. Elytra. Striae present; anterior edge outline straight to arcuate near humeri in dorsal view; integument with metallic reflections or unmarked with spots or tranverse bands; without pattern from differences in setal colour; setal vestiture even and mainly parallel. **Legs.** Metacoxal plate without elongation in mesal half, plate reaching lateral edge; tarsal pads or membranous lobes absent; tarsal claws without setae, simple. **Ventrites.** Microserration at sides (e.g. 100 points per mm) absent, ventrite 5 apex arcuate without paired setal brushes. **Aedeagus.** Parameres with articulation at base, apical lateral expansions present; parameres without setae. **Geography.** Known from British Columbia, Alberta, Manitoba+ (MB, SK), Ontario, Oregon+ (WA, OR, ID), California, Montana+ (MT, WY, ND) and Nebraska+ (SD, NE, IA) ['+' refers to several states or provinces collectively].

9.2 TESOLASOMUS Johnson, 2021

Habitus. Body Length 5-10 mm, 10-15 mm or 15-20 mm. Vestiture. Scale like setae absent. **Head.** Supra-antennal carinae fading on frons (not reaching another structure); prognathous (labrum oriented less than 90 degrees downward); frons without triangular depression. Antennae with 11 antennomeres, not pectinate, sensory elements beginning on antennomere IV. **Prothorax.** Pronotum wider than long at widest point (including hind angles); dorsal punctures uniform sized, all simple (floor of puncture concave), without tubercles or longitudinal carinae between punctures; pronotal lateral carina complete anteriorly, carina visible throughout length in dorsal view, meeting anterior edge of prothorax at about 90 degrees in lateral view, not serrate; bioluminescent spots absent; hind angle carinae present (single); posterior edge of pronotum with sublateral plicae and notches present, crenellations absent; hypomeron posterior edge near each hind angle with concavity, concavity arcuate; pronotosternal sutures closed, hypomeral beads absent. Prosternum with sides straight or concave at midlength in ventral view; prosternal process not curved upward more than 40 degrees in lateral view. **Mesothorax.** Mesocoxal cavity open; mesoventral cavity without serration along sides. Elytra. Striae present; anterior edge outline straight to arcuate near humeri in dorsal view; integument marked with spots or transverse bands or unmarked with spots or tranverse bands; without pattern from differences in setal colour; setal vestiture even and mainly parallel. **Legs.** Metacoxal plate without elongation in mesal half, plate reaching lateral edge; tarsal pads or membranous lobes absent; tarsal claws without setae, simple. **Ventrites.** Microserration at sides (e.g. 100 points per mm) absent, ventrite 5 apex arcuate without paired setal brushes. **Aedeagus.** Parameres with articulation at base, apical lateral expansions present; parameres without setae. **Geography.** Known from Newfoundland and Labrador, Oregon+ (WA, OR, ID), California, Montana+ (MT, WY, ND), Colorado+ (NV, UT, CO), Arizona+ (AZ, NM), Nebraska+ (SD, NE, IA), Arkansas+ (KS, MO, OK, AR, LA, MS), Pennsylvania+ (PA, WV), Carolinas+ (KY, TN, NC, SC, GA, AL) and Florida ['+' refers to several states or provinces collectively].

T. morulus and T. deceptor

Habitus. Body Length 5-10 mm or 10-15 mm. Vestiture. Scale like setae absent. **Head.** Supra-antennal carinae fading on frons (not reaching another structure); prognathous (labrum oriented less than 90 degrees downward); frons without triangular depression. Antennae with 11 antennomeres, not pectinate, sensory elements beginning on antennomere IV. **Prothorax.** Pronotum wider than long at widest point (including hind angles); dorsal punctures uniform sized, all simple (floor of puncture concave), without tubercles or longitudinal carinae between punctures; pronotal lateral carina complete

anteriorly, carina visible throughout length in dorsal view, meeting anterior edge of prothorax at about 90 degrees in lateral view, not serrate; bioluminescent spots absent; hind angle carinae present (single); posterior edge of pronotum with sublateral plicae and notches present, crenellations absent; hypomeron posterior edge near each hind angle with concavity; pronotosternal sutures closed, hypomeral beads present. Prosternum with sides straight or concave at midlength in ventral view; prosternal process not curved upward more than 40 degrees in lateral view. **Mesothorax.** Mesocoxal cavity open; mesoventral cavity without serration along sides. Elytra. Striae present; anterior edge outline straight to arcuate near humeri in dorsal view; integument unmarked with spots or tranverse bands; without pattern from differences in setal colour; setal vestiture mainly absent on disk or even and mainly parallel. **Legs.** Metacoxal plate without elongation in mesal half, plate reaching lateral edge; tarsal pads or membranous lobes absent; tarsal claws without setae, simple. **Ventrites.** Microserration at sides (e.g. 100 points per mm) absent, ventrite 5 apex bisinuate with paired setal brushes (larger in males). **Aedeagus.** Parameres with articulation at base, apical lateral expansions present; each paramere with three or more setae. **Geography.** Known from Nunavut+ (NT, NU), Yukon Territory, British Columbia, Alberta, Manitoba+ (MB, SK), Ontario, Quebec, Alaska, Oregon+ (WA, OR, ID), California, Montana+ (MT, WY, ND), Colorado+ (NV, UT, CO) and Nebraska+ (SD, NE, IA) ['+' refers to several states or provinces collectively].

9.3 ATHOUS Eschscholtz, 1829

ATHOUS Eschscholtz, 1829 -brightwelli group

Habitus. Body Length 5-10 mm, 10-15 mm or 15-20 mm. Vestiture. Scale like setae absent. **Head.** Supra-antennal carinae joining medially (forming shelf); nasale (head capsule below edge of frontal carina) with outline concave in lateral view; hypognathous (labrum oriented downward 90 degrees or more) or prognathous (labrum oriented less than 90 degrees downward); frons with triangular depression. Antennae with 11 antennomeres, not pectinate, sensory elements beginning on antennomere III. **Prothorax.** Pronotum longer than wide at widest point (including hind angles); dorsal punctures uniform sized, all simple (floor of puncture concave), without tubercles or longitudinal carinae between punctures; pronotal lateral carina complete anteriorly, carina visible throughout length in dorsal view, meeting anterior edge of prothorax at about 90 degrees in lateral view, microserrate at hind angles only (posterior 10%, in concavity); bioluminescent spots absent; hind angle carinae absent; posterior edge of pronotum with sublateral plicae and notches present or absent, crenellations absent; hypomeron posterior edge near each hind angle without concavity; pronotosternal sutures closed, hypomeral beads present. Prosternum with sides straight at midlength in ventral view; prosternal process not curved upward more than 40 degrees in lateral view. **Mesothorax.** Mesocoxal cavity open; mesoventral cavity without serration along sides. Elytra. Striae present; anterior edge outline straight to arcuate or sinuate (recurved) or with rectangular projection near humeri in dorsal view; integument unmarked with spots or tranverse bands; without pattern from differences in setal colour; setal vestiture even and mainly parallel. **Legs.** Metacoxal plate without elongation in mesal half, plate reaching lateral edge; tarsal pads or membranous lobes present on multiple tarsomeres, (II and III); tarsal claws without setae, simple. **Ventrites.** Microserration at sides (e.g. 100 points per mm) absent, ventrite 5 apex arcuate without paired setal brushes. **Aedeagus.** Parameres with articulation at base, apical lateral expansions present; each paramere with three or more setae. **Geography.** Known from British Columbia, Ontario, Quebec, New Brunswick+ (NB, NS, PE), Alaska, Oregon+ (WA, OR, ID), California, Montana+ (MT, WY, ND), Arizona+ (AZ, NM), Nebraska+ (SD, NE, IA), Michigan+ (MN, WI, MI), Indiana+ (IL, IN, OH), Arkansas+ (KS, MO, OK, AR, LA, MS), Vermont+ (ME, VT, NH), Massachusetts+ (MA, CT, RI), New York,

Pennsylvania+ (PA, WV), Virginia+ (NJ, DE, MD, DC, VA), Carolinas+ (KY, TN, NC, SC, GA, AL) and Florida ['+' refers to several states or provinces collectively].

ATHOUS Eschscholtz, 1829 -campyloides group

Habitus. Body Length 5-10 mm, 10-15 mm or 15-20 mm. Vestiture. Scale like setae absent. **Head.** Supra-antennal carinae joining medially (forming shelf); nasale (head capsule below edge of frontal carina) with outline concave in lateral view; prognathous (labrum oriented less than 90 degrees downward); frons with triangular depression. Antennae with 11 antennomeres, not pectinate, sensory elements beginning on antennomere IV. **Prothorax.** Pronotum longer than wide at widest point (including hind angles); dorsal punctures uniform sized, all simple (floor of puncture concave) or some or all punctures umbilicate (floor of puncture flat), without tubercles or longitudinal carinae between punctures; pronotal lateral carina complete anteriorly, carina visible throughout length in dorsal view, meeting anterior edge of prothorax at about 90 degrees in lateral view, microserrate along entire side (e.g. about 70 points per mm) or microserrate at hind angles only (posterior 10%, in concavity); bioluminescent spots absent; hind angle carinae absent; posterior edge of pronotum with sublateral plicae and notches present, crenellations absent; hypomeron posterior edge near each hind angle without concavity; pronotosternal sutures closed, hypomeral beads present. Prosternum with sides straight at midlength in ventral view; prosternal process not curved upward more than 40 degrees in lateral view. **Mesothorax.** Mesocoxal cavity open; mesoventral cavity without serration along sides. Elytra. Striae present; anterior edge outline sinuate (recurved) or with rectangular projection near humeri in dorsal view; integument unmarked with spots or tranverse bands; without pattern from differences in setal colour; setal vestiture even and mainly parallel. **Legs.** Metacoxal plate without elongation in mesal half, plate reaching or not reaching lateral edge; tarsal pads or membranous lobes present on multiple tarsomeres, (II and III) or (I, II, III, and IV or II, III, and IV); tarsal claws without setae, simple. **Ventrites.** Microserration at sides (e.g. 100 points per mm) absent, ventrite 5 apex arcuate without paired setal brushes. **Aedeagus.** Parameres with articulation at base, apical lateral expansions present; each paramere with three or more setae. **Geography.** Known from Quebec, New Brunswick+ (NB, NS, PE), Oregon+ (WA, OR, ID), California, Arizona+ (AZ, NM) and Massachusetts+ (MA, CT, RI) ['+' refers to several states or provinces collectively].

ATHOUS Eschscholtz, 1829 -cucullatus group

Habitus. Body Length 5-10 mm or 10-15 mm. Vestiture. Scale like setae absent. **Head.** Supra-antennal carinae joining medially (forming shelf); nasale (head capsule below edge of frontal carina) with outline concave in lateral view; hypognathous (labrum oriented downward 90 degrees or more) or prognathous (labrum oriented less than 90 degrees downward); frons with triangular depression. Antennae with 11 antennomeres, not pectinate, sensory elements beginning on antennomere III. **Prothorax.** Pronotum wider than long or longer than wide at widest point (including hind angles); dorsal punctures uniform sized, some or all punctures umbilicate (floor of puncture flat), without tubercles or longitudinal carinae between punctures; pronotal lateral carina complete anteriorly, carina visible throughout length in dorsal view, meeting anterior edge of prothorax at about 90 degrees in lateral view, microserrate along entire side (e.g. about 70 points per mm); bioluminescent spots absent; hind angle carinae present (single) or absent; posterior edge of pronotum with sublateral plicae and notches present or absent, crenellations absent; hypomeron posterior edge near each hind angle without concavity; pronotosternal sutures closed, hypomeral beads absent. Prosternum with sides straight or convex at midlength in ventral view; prosternal process not curved upward more than 40 degrees in lateral view. **Mesothorax.** Mesocoxal cavity open; mesoventral cavity without serration along sides. Elytra.

Striae present; anterior edge outline straight to arcuate or sinuate (recurved) or with rectangular projection near humeri in dorsal view; integument unmarked with spots or tranverse bands; without pattern from differences in setal colour; setal vestiture even and mainly parallel. **Legs.** Metacoxal plate without elongation in mesal half, plate reaching or not reaching lateral edge; tarsal pads or membranous lobes present on multiple tarsomeres, (II and III) or (I, II, III, and IV or II, III, and IV); tarsal claws without setae, simple. **Ventrites.** Microserration at sides (e.g. 100 points per mm) present or absent, ventrite 5 apex arcuate without paired setal brushes. **Aedeagus.** Parameres with articulation at base, apical lateral expansions absent; each paramere with three or more setae. **Geography.** Known from British Columbia, Alberta, Ontario, Quebec, New Brunswick+ (NB, NS, PE), Alaska, Oregon+ (WA, OR, ID), California, Montana+ (MT, WY, ND), Colorado+ (NV, UT, CO), Arizona+ (AZ, NM), Nebraska+ (SD, NE, IA), Texas, Michigan+ (MN, WI, MI), Indiana+ (IL, IN, OH), Arkansas+ (KS, MO, OK, AR, LA, MS), Vermont+ (ME, VT, NH), Massachusetts+ (MA, CT, RI), New York, Pennsylvania+ (PA, WV), Virginia+ (NJ, DE, MD, DC, VA), Carolinas+ (KY, TN, NC, SC, GA, AL) and Florida ['+' refers to several states or provinces collectively].

ATHOUS Eschscholtz, 1829 -imitans group

Habitus. Body Length 1-5 mm, 5-10 mm or 10-15 mm. Vestiture. Scale like setae absent. **Head.** Supra-antennal carinae joining medially (forming shelf); nasale (head capsule below edge of frontal carina) with outline concave in lateral view; hypognathous (labrum oriented downward 90 degrees or more) or prognathous (labrum oriented less than 90 degrees downward); frons without triangular depression. Antennae with 11 antennomeres, not pectinate, sensory elements beginning on antennomere IV. **Prothorax.** Pronotum longer than wide at widest point (including hind angles); dorsal punctures uniform sized, all simple (floor of puncture concave), without tubercles or longitudinal carinae between punctures; pronotal lateral carina complete anteriorly, carina visible throughout length in dorsal view, meeting anterior edge of prothorax at about 90 degrees in lateral view, microserrate at hind angles only (posterior 10%, in concavity) or not serrate; bioluminescent spots absent; hind angle carinae absent; posterior edge of pronotum with sublateral plicae and notches absent, crenellations absent; hypomeron posterior edge near each hind angle without concavity; pronotosternal sutures closed, hypomeral beads present or absent. Prosternum with sides straight at midlength in ventral view; prosternal process not curved upward more than 40 degrees in lateral view. **Mesothorax.** Mesocoxal cavity open to mesepimeron only; mesoventral cavity without serration along sides. Elytra. Striae present; anterior edge outline straight to arcuate near humeri in dorsal view; integument marked with spots or transverse bands or unmarked with spots or tranverse bands; without pattern from differences in setal colour; setal vestiture even and mainly parallel. **Legs.** Metacoxal plate without elongation in mesal half, plate not reaching lateral edge; tarsal pads or membranous lobes present on multiple tarsomeres, (I, II, III, and IV or II, III, and IV); tarsal claws without setae, simple. **Ventrites.** Microserration at sides (e.g. 100 points per mm) absent, ventrite 5 apex arcuate without paired setal brushes. **Aedeagus.** Parameres with articulation at base, apical lateral expansions present; each paramere with three or more setae. **Geography.** Known from British Columbia, Oregon+ (WA, OR, ID), California, Colorado+ (NV, UT, CO), Indiana+ (IL, IN, OH), Arkansas+ (KS, MO, OK, AR, LA, MS), Pennsylvania+ (PA, WV), Virginia+ (NJ, DE, MD, DC, VA) and Carolinas+ (KY, TN, NC, SC, GA, AL) ['+' refers to several states or provinces collectively].

ATHOUS Eschscholtz, 1829 -productus group

Habitus. Body Length 10-15 mm or 15-20 mm. Vestiture. Scale like setae absent. **Head.** Supra-antennal carinae joining medially (forming shelf); nasale (head capsule below edge of frontal carina) with outline concave in lateral view; hypognathous (labrum oriented downward 90 degrees or more) or prognathous

(labrum oriented less than 90 degrees downward); frons with triangular depression. Antennae with 11 antennomeres, not pectinate, sensory elements beginning on antennomere III. **Prothorax.** Pronotum wider than long at widest point (including hind angles); dorsal punctures uniform sized, some or all punctures umbilicate (floor of puncture flat), without tubercles or longitudinal carinae between punctures; pronotal lateral carina complete anteriorly, carina visible throughout length in dorsal view, meeting anterior edge of prothorax at about 90 degrees in lateral view, not serrate; bioluminescent spots absent; hind angle carinae absent; posterior edge of pronotum with sublateral plicae and notches absent, crenellations absent; hypomeron posterior edge near each hind angle with concavity, concavity arcuate; pronotosternal sutures closed, hypomeral beads present. Prosternum with sides straight or concave at midlength in ventral view; prosternal process not curved upward more than 40 degrees in lateral view. **Mesothorax.** Mesocoxal cavity open; mesoventral cavity without serration along sides. Elytra. Striae present; anterior edge outline straight to arcuate near humeri in dorsal view; integument unmarked with spots or tranverse bands; without pattern from differences in setal colour; setal vestiture even and mainly parallel. **Legs.** Metacoxal plate without elongation in mesal half, plate reaching lateral edge; tarsal pads or membranous lobes present on multiple tarsomeres, (I, II, III, and IV or II, III, and IV); tarsal claws without setae, simple. **Ventrites.** Microserration at sides (e.g. 100 points per mm) absent, ventrite 5 apex arcuate without paired setal brushes. **Aedeagus.** Parameres with articulation at base, apical lateral expansions present; each paramere with three or more setae. **Geography.** Known from Alberta, Manitoba+ (MB, SK), Ontario, Quebec, New Brunswick+ (NB, NS, PE), Newfoundland and Labrador, Michigan+ (MN, WI, MI) and Vermont+ (ME, VT, NH) ['+' refers to several states or provinces collectively].

ATHOUS Eschscholtz, 1829 -rufifrons group

Habitus. Body Length 10-15 mm or 15-20 mm. Vestiture. Scale like setae absent. **Head.** Supra-antennal carinae joining medially (forming shelf); nasale (head capsule below edge of frontal carina) with outline concave in lateral view; hypognathous (labrum oriented downward 90 degrees or more) or prognathous (labrum oriented less than 90 degrees downward); frons with triangular depression. Antennae with 11 antennomeres, not pectinate, sensory elements beginning on antennomere III. **Prothorax.** Pronotum longer than wide at widest point (including hind angles); dorsal punctures uniform sized, some or all punctures umbilicate (floor of puncture flat), without tubercles or longitudinal carinae between punctures; pronotal lateral carina complete anteriorly, carina visible throughout length in dorsal view, meeting anterior edge of prothorax at about 90 degrees in lateral view, microserrate at hind angles only (posterior 10%, in concavity); bioluminescent spots absent; hind angle carinae absent; posterior edge of pronotum with sublateral plicae and notches present, crenellations absent; hypomeron posterior edge near each hind angle without concavity; pronotosternal sutures closed, hypomeral beads present. Prosternum with sides straight at midlength in ventral view; prosternal process not curved upward more than 40 degrees in lateral view. **Mesothorax.** Mesocoxal cavity open; mesoventral cavity without serration along sides. Elytra. Striae present; anterior edge outline sinuate (recurved) or with rectangular projection near humeri in dorsal view; integument unmarked with spots or tranverse bands; without pattern from differences in setal colour; setal vestiture even and mainly parallel. **Legs.** Metacoxal plate without elongation in mesal half, plate not reaching lateral edge; tarsal pads or membranous lobes absent; tarsal claws without setae, simple. **Ventrites.** Microserration at sides (e.g. 100 points per mm) present, ventrite 5 apex arcuate without paired setal brushes. **Aedeagus.** Parameres with articulation at base, apical lateral expansions present; each paramere with three or more setae. **Geography.** Known from Manitoba+ (MB, SK), Ontario, Quebec, New Brunswick+ (NB, NS, PE), Newfoundland and Labrador, Michigan+ (MN, WI, MI), Indiana+ (IL, IN, OH), Vermont+ (ME, VT, NH), Massachusetts+ (MA, CT, RI),

New York, Pennsylvania+ (PA, WV), Virginia+ (NJ, DE, MD, DC, VA) and Carolinas+ (KY, TN, NC, SC, GA, AL) ['+' refers to several states or provinces collectively].

ATHOUS Eschscholtz, 1829 -scapularis group

Habitus. Body Length 5-10 mm, 10-15 mm or 15-20 mm. Vestiture. Scale like setae absent. **Head.** Supra-antennal carinae joining medially (forming shelf); nasale (head capsule below edge of frontal carina) with outline concave in lateral view; hypognathous (labrum oriented downward 90 degrees or more) or prognathous (labrum oriented less than 90 degrees downward); frons with triangular depression. Antennae with 11 antennomeres, not pectinate, sensory elements beginning on antennomere III or IV. **Prothorax.** Pronotum wider than long or longer than wide at widest point (including hind angles); dorsal punctures uniform sized, some or all punctures umbilicate (floor of puncture flat), without tubercles or longitudinal carinae between punctures; pronotal lateral carina complete anteriorly, carina visible throughout length in dorsal view, meeting anterior edge of prothorax at about 90 degrees in lateral view, microserrate along entire side (e.g. about 70 points per mm) or microserrate at hind angles only (posterior 10%, in concavity); bioluminescent spots absent; hind angle carinae present (single); posterior edge of pronotum with sublateral plicae and notches absent, crenellations absent; hypomeron posterior edge near each hind angle without concavity; pronotosternal sutures closed, hypomeral beads absent. Prosternum with sides straight at midlength in ventral view; prosternal process not curved upward more than 40 degrees in lateral view. **Mesothorax.** Mesocoxal cavity open; mesoventral cavity without serration along sides. Elytra. Striae present; anterior edge outline straight to arcuate near humeri in dorsal view; integument unmarked with spots or tranverse bands; without pattern from differences in setal colour; setal vestiture even and mainly parallel. **Legs.** Metacoxal plate without elongation in mesal half, plate reaching lateral edge; tarsal pads or membranous lobes present on multiple tarsomeres, (II and III); tarsal claws without setae, simple. **Ventrites.** Microserration at sides (e.g. 100 points per mm) absent, ventrite 5 apex arcuate without paired setal brushes. **Aedeagus.** Parameres with articulation at base, apical lateral expansions present; each paramere with three or more setae. **Geography.** Known from Alberta, Ontario, Quebec, New Brunswick+ (NB, NS, PE), Oregon+ (WA, OR, ID), California, Montana+ (MT, WY, ND), Michigan+ (MN, WI, MI), Indiana+ (IL, IN, OH), Arkansas+ (KS, MO, OK, AR, LA, MS), Vermont+ (ME, VT, NH), Massachusetts+ (MA, CT, RI), New York, Pennsylvania+ (PA, WV), Virginia+ (NJ, DE, MD, DC, VA) and Carolinas+ (KY, TN, NC, SC, GA, AL) ['+' refers to several states or provinces collectively].

ATHOUS Eschscholtz, 1829 -scissus group

Habitus. Body Length 10-15 mm, 15-20 mm or 20-40 mm. Vestiture. Scale like setae absent. **Head.** Supra-antennal carinae joining medially (forming shelf); nasale (head capsule below edge of frontal carina) with outline concave in lateral view; prognathous (labrum oriented less than 90 degrees downward); frons with triangular depression. Antennae with 11 antennomeres, not pectinate, sensory elements beginning on antennomere III or IV. **Prothorax.** Pronotum longer than wide at widest point (including hind angles); dorsal punctures uniform sized, some or all punctures umbilicate (floor of puncture flat), without tubercles or longitudinal carinae between punctures; pronotal lateral carina complete anteriorly, carina visible throughout length in dorsal view, meeting anterior edge of prothorax at about 90 degrees in lateral view, microserrate at hind angles only (posterior 10%, in concavity); bioluminescent spots absent; hind angle carinae absent; posterior edge of pronotum with sublateral plicae and notches present, crenellations absent; hypomeron posterior edge near each hind angle without concavity; pronotosternal sutures closed, hypomeral beads present or absent. Prosternum with sides straight at midlength in ventral view; prosternal process curved or not curved upward more than

40 degrees in lateral view. **Mesothorax.** Mesocoxal cavity open; mesoventral cavity without serration along sides. Elytra. Striae present; anterior edge outline straight to arcuate or sinuate (recurved) or with rectangular projection near humeri in dorsal view; integument unmarked with spots or tranverse bands; without pattern from differences in setal colour; setal vestiture even and mainly parallel. **Legs.** Metacoxal plate without elongation in mesal half, plate reaching or not reaching lateral edge; tarsal pads or membranous lobes present on multiple tarsomeres, (II and III) or (I, II, III, and IV or II, III, and IV); tarsal claws without setae, simple. **Ventrites.** Microserration at sides (e.g. 100 points per mm) present, ventrite 5 apex arcuate without paired setal brushes. **Aedeagus.** Parameres with articulation at base, apical lateral expansions present; each paramere with three or more setae. **Geography.** Known from British Columbia, Oregon+ (WA, OR, ID) and California ['+' refers to several states or provinces collectively].

ATHOUS Eschscholtz, 1829 -subgenus Athous

Habitus. Body Length 5-10 mm or 10-15 mm. Vestiture. Scale like setae absent. **Head.** Supra-antennal carinae joining medially (forming shelf); nasale (head capsule below edge of frontal carina) with outline concave in lateral view; prognathous (labrum oriented less than 90 degrees downward); frons without triangular depression. Antennae with 11 antennomeres, not pectinate, sensory elements beginning on antennomere IV. **Prothorax.** Pronotum longer than wide at widest point (including hind angles); dorsal punctures uniform sized, all simple (floor of puncture concave) or some or all punctures umbilicate (floor of puncture flat), without tubercles or longitudinal carinae between punctures; pronotal lateral carina complete anteriorly, carina visible throughout length in dorsal view, meeting anterior edge of prothorax at about 90 degrees in lateral view, microserrate along entire side (e.g. about 70 points per mm); bioluminescent spots absent; hind angle carinae absent; posterior edge of pronotum with sublateral plicae and notches present, crenellations absent; hypomeron posterior edge near each hind angle without concavity; pronotosternal sutures closed, hypomeral beads present or absent. Prosternum with sides straight at midlength in ventral view; prosternal process not curved upward more than 40 degrees in lateral view. **Mesothorax.** Mesocoxal cavity open; mesoventral cavity without serration along sides. Elytra. Striae present; anterior edge outline straight to arcuate near humeri in dorsal view; integument unmarked with spots or tranverse bands; without pattern from differences in setal colour; setal vestiture even and mainly parallel. **Legs.** Metacoxal plate without elongation in mesal half, plate reaching or not reaching lateral edge; tarsal pads or membranous lobes present on multiple tarsomeres, (II and III); tarsal claws without setae, simple. **Ventrites.** Microserration at sides (e.g. 100 points per mm) present, ventrite 5 apex arcuate without paired setal brushes. **Aedeagus.** Parameres with articulation at base, apical lateral expansions present; parameres without setae or each paramere with three or more setae. **Geography.** Known from Ontario and Massachusetts+ (MA, CT, RI) ['+' refers to several states or provinces collectively].

9.3 BARRELATER Johnson, 2014

Habitus. Body Length 10-15 mm or 15-20 mm. Vestiture. Scale like setae absent. **Head.** Supra-antennal carinae joining medially (forming shelf); nasale (head capsule below edge of frontal carina) with outline concave or not concave in lateral view; prognathous (labrum oriented less than 90 degrees downward); frons without triangular depression. Antennae with 11 antennomeres, not pectinate, sensory elements beginning on antennomere III. **Prothorax.** Pronotum wider than long or longer than wide at widest point (including hind angles); dorsal punctures uniform sized, some or all punctures umbilicate (floor of puncture flat), without tubercles or longitudinal carinae between punctures; pronotal lateral carina complete anteriorly, carina visible throughout length in dorsal view, meeting anterior edge of prothorax

at about 90 degrees in lateral view, microserrate at hind angles only (posterior 10%, in concavity); bioluminescent spots absent; hind angle carinae absent; posterior edge of pronotum with sublateral plicae and notches present, crenellations absent; hypomeron posterior edge near each hind angle without concavity; pronotosternal sutures closed, hypomeral beads absent. Prosternum with sides straight at midlength in ventral view; prosternal process curved upward more than 40 degrees in lateral view. **Mesothorax.** Mesocoxal cavity open; mesoventral cavity without serration along sides. Elytra. Striae present; anterior edge outline straight to arcuate near humeri in dorsal view; integument unmarked with spots or tranverse bands; without pattern from differences in setal colour; setal vestiture even and mainly parallel. **Legs.** Metacoxal plate without elongation in mesal half, plate reaching lateral edge; tarsal pads or membranous lobes present on multiple tarsomeres or absent, (I, II, III, and IV or II, III, and IV); tarsal claws without setae, simple. **Ventrites.** Microserration at sides (e.g. 100 points per mm) absent, ventrite 5 apex arcuate without paired setal brushes. **Aedeagus.** Parameres with articulation at base, apical lateral expansions present; each paramere with three or more setae. **Geography.** Known from Oregon+ (WA, OR, ID) and Colorado+ (NV, UT, CO) ['+' refers to several states or provinces collectively].

9.3 DENTICOLLIS Piller & Mitterpacher, 1783

Habitus. Body Length 5-10 mm or 10-15 mm. Vestiture. Scale like setae absent. **Head.** Supra-antennal carinae joining medially (forming shelf); nasale (head capsule below edge of frontal carina) with outline concave in lateral view; prognathous (labrum oriented less than 90 degrees downward); frons with triangular depression. Antennae with 11 antennomeres, not pectinate, sensory elements beginning on antennomere III. **Prothorax.** Pronotum wider than long at widest point (including hind angles); dorsal punctures uniform sized, all simple (floor of puncture concave) or some or all punctures umbilicate (floor of puncture flat), without tubercles or longitudinal carinae between punctures; pronotal lateral carina complete anteriorly, carina visible throughout length in dorsal view, meeting anterior edge of prothorax at about 90 degrees in lateral view, not serrate; bioluminescent spots absent; hind angle carinae absent; posterior edge of pronotum with sublateral plicae and notches absent, crenellations absent; hypomeron posterior edge near each hind angle without concavity; pronotosternal sutures closed, hypomeral beads present. Prosternum with sides straight at midlength in ventral view; prosternal process curved or not curved upward more than 40 degrees in lateral view. **Mesothorax.** Mesocoxal cavity open; mesoventral cavity without serration along sides. Elytra. Striae present or absent; anterior edge outline straight to arcuate or sinuate (recurved) or with rectangular projection near humeri in dorsal view; integument unmarked with spots or tranverse bands; without pattern from differences in setal colour; setal vestiture even and mainly parallel. **Legs.** Metacoxal plate without elongation in mesal half, plate reaching or not reaching lateral edge; tarsal pads or membranous lobes present on tarsomere IV only or absent; tarsal claws without setae, simple. **Ventrites.** Microserration at sides (e.g. 100 points per mm) absent, ventrite 5 apex arcuate without paired setal brushes. **Aedeagus.** Parameres with articulation at base, apical lateral expansions present; each paramere with three or more setae. **Geography.** Known from Nunavut+ (NT, NU), Yukon Territory, British Columbia, Alberta, Manitoba+ (MB, SK), Ontario, Quebec, New Brunswick+ (NB, NS, PE), Newfoundland and Labrador, Alaska, Michigan+ (MN, WI, MI), Vermont+ (ME, VT, NH), Massachusetts+ (MA, CT, RI), New York, Pennsylvania+ (PA, WV) and Carolinas+ (KY, TN, NC, SC, GA, AL) ['+' refers to several states or provinces collectively].

9.3 DIACANTHOUS Reitter, 1852

Habitus. Body Length 10-15 mm. Vestiture. Scale like setae absent. **Head.** Supra-antennal carinae joining medially (forming shelf); nasale (head capsule below edge of frontal carina) with outline concave in lateral view; prognathous (labrum oriented less than 90 degrees downward); frons with triangular depression. Antennae with 11 antennomeres, not pectinate, sensory elements beginning on antennomere III. **Prothorax.** Pronotum wider than long or longer than wide at widest point (including hind angles); dorsal punctures uniform sized, some or all punctures umbilicate (floor of puncture flat), without tubercles or longitudinal carinae between punctures; pronotal lateral carina complete anteriorly, carina visible throughout length in dorsal view, meeting anterior edge of prothorax at about 90 degrees in lateral view, microserrate along entire side (e.g. about 70 points per mm); bioluminescent spots absent; hind angle carinae absent; posterior edge of pronotum with sublateral plicae and notches absent, crenellations absent; hypomeron posterior edge near each hind angle with concavity, concavity arcuate; pronotosternal sutures closed, hypomeral beads absent. Prosternum with sides straight or concave at midlength in ventral view; prosternal process curved or not curved upward more than 40 degrees in lateral view. **Mesothorax.** Mesocoxal cavity open; mesoventral cavity without serration along sides. Elytra. Striae present; anterior edge outline straight to arcuate near humeri in dorsal view; integument unmarked with spots or tranverse bands; with pattern from differences in setal colour; setal vestiture even and mainly parallel. **Legs.** Metacoxal plate without elongation in mesal half, plate reaching lateral edge; tarsal pads or membranous lobes present on multiple tarsomeres, (I, II, III, and IV or II, III, and IV); tarsal claws without setae, simple. **Ventrites.** Microserration at sides (e.g. 100 points per mm) absent, ventrite 5 apex arcuate without paired setal brushes. **Aedeagus.** Parameres with articulation at base, apical lateral expansions absent; each paramere with three or more setae. **Geography.** Known from Yukon Territory, British Columbia, Alberta, Manitoba+ (MB, SK), Ontario, Quebec, Alaska, Michigan+ (MN, WI, MI) and Vermont+ (ME, VT, NH) ['+' refers to several states or provinces collectively].

9.3 ELATHOUS Reitter, 1890

Habitus. Body Length 5-10 mm or 10-15 mm. Vestiture. Scale like setae absent. **Head.** Supra-antennal carinae joining medially (forming shelf); nasale (head capsule below edge of frontal carina) with outline concave in lateral view; prognathous (labrum oriented less than 90 degrees downward), rarely hypognathous (labrum oriented downward 90 degrees or more); frons with triangular depression. Antennae with 11 antennomeres, not pectinate, sensory elements beginning on antennomere IV. **Prothorax.** Pronotum wider than long or longer than wide at widest point (including hind angles); dorsal punctures uniform sized, some or all punctures umbilicate (floor of puncture flat), without tubercles or longitudinal carinae between punctures; pronotal lateral carina complete anteriorly, carina visible throughout length in dorsal view, meeting anterior edge of prothorax at about 90 degrees in lateral view, not serrate; bioluminescent spots absent; hind angle carinae present (single); posterior edge of pronotum with sublateral plicae and notches present or absent, crenellations absent; hypomeron posterior edge near each hind angle without concavity; pronotosternal sutures open, hypomeral beads present. Prosternum with sides straight at midlength in ventral view; prosternal process curved or not curved upward more than 40 degrees in lateral view. **Mesothorax.** Mesocoxal cavity open or open to mesepimeron only; mesoventral cavity without serration along sides. Elytra. Striae present; anterior edge outline straight to arcuate near humeri in dorsal view; integument unmarked with spots or tranverse bands; without pattern from differences in setal colour; setal vestiture even and mainly parallel. **Legs.** Metacoxal plate without elongation in mesal half, plate reaching lateral edge; tarsal pads or membranous lobes absent; tarsal claws without setae, simple. **Ventrites.** Microserration at sides (e.g. 100 points per mm) present or absent, ventrite 5 apex arcuate without paired setal brushes. **Aedeagus.** Parameres with articulation at base, apical lateral

expansions present; each paramere with three or more setae. **Geography.** Known from British Columbia, Ontario, Quebec, New Brunswick+ (NB, NS, PE), Oregon+ (WA, OR, ID), California, Montana+ (MT, WY, ND), Arizona+ (AZ, NM), Michigan+ (MN, WI, MI), Indiana+ (IL, IN, OH), Arkansas+ (KS, MO, OK, AR, LA, MS), Vermont+ (ME, VT, NH), New York, Pennsylvania+ (PA, WV) and Carolinas+ (KY, TN, NC, SC, GA, AL) ['+' refers to several states or provinces collectively].

9.3 EUPLASTIUS Schwarz 1903

Habitus. Body Length 5-10 mm or 10-15 mm. Vestiture. Scale like setae absent. **Head.** Supra-antennal carinae fading on frons (not reaching another structure); nasale (head capsule below edge of frontal carina) with outline not concave in lateral view; prognathous (labrum oriented less than 90 degrees downward); frons without triangular depression. Antennae with 11 antennomeres, not pectinate, sensory elements beginning on antennomere III. **Prothorax.** Pronotum longer than wide at widest point (including hind angles); dorsal punctures uniform sized, all simple (floor of puncture concave) or some or all punctures umbilicate (floor of puncture flat), without tubercles or longitudinal carinae between punctures; pronotal lateral carina complete anteriorly, carina visible throughout length in dorsal view or carina not visible anteriorly in dorsal view, meeting anterior edge of prothorax at about 90 degrees in lateral view, not serrate; bioluminescent spots absent; hind angle carinae present (single) or absent; posterior edge of pronotum with sublateral plicae and notches absent, crenellations absent; hypomeron posterior edge near each hind angle with or without concavity, concavity arcuate; pronotosternal sutures closed, hypomeral beads present or absent. Prosternum with sides straight or convex at midlength in ventral view; prosternal process not curved upward more than 40 degrees in lateral view. **Mesothorax.** Mesocoxal cavity open; mesoventral cavity without serration along sides. Elytra. Striae present; anterior edge outline straight to arcuate or sinuate (recurved) or with rectangular projection near humeri in dorsal view; integument unmarked with spots or tranverse bands; without pattern from differences in setal colour; setal vestiture even and mainly parallel. **Legs.** Metacoxal plate without elongation in mesal half, plate reaching lateral edge; tarsal pads or membranous lobes absent; tarsal claws without setae, simple. **Ventrites.** Microserration at sides (e.g. 100 points per mm) absent, ventrite 5 apex arcuate without paired setal brushes. **Aedeagus.** Parameres with articulation at base, apical lateral expansions present; parameres without setae. **Geography.** Known from California, Texas, Michigan+ (MN, WI, MI), Indiana+ (IL, IN, OH), Arkansas+ (KS, MO, OK, AR, LA, MS), Pennsylvania+ (PA, WV), Virginia+ (NJ, DE, MD, DC, VA) and Carolinas+ (KY, TN, NC, SC, GA, AL) ['+' refers to several states or provinces collectively].

9.3 GAMBRINUS LeConte 1853

Habitus. Body Length 5-10 mm, 10-15 mm or 15-20 mm. Vestiture. Scale like setae absent. **Head.** Supra-antennal carinae joining medially (forming shelf); nasale (head capsule below edge of frontal carina) with outline concave in lateral view; prognathous (labrum oriented less than 90 degrees downward); frons without triangular depression. Antennae with 11 antennomeres, not pectinate, sensory elements beginning on antennomere IV. **Prothorax.** Pronotum wider than long or longer than wide at widest point (including hind angles); dorsal punctures uniform sized, all simple (floor of puncture concave) or some or all punctures umbilicate (floor of puncture flat), without tubercles or longitudinal carinae between punctures; pronotal lateral carina complete anteriorly, carina visible throughout length in dorsal view or carina not visible anteriorly in dorsal view, meeting anterior edge of prothorax at about 90 degrees in lateral view, not serrate; bioluminescent spots absent; hind angle carinae present (single); posterior edge of pronotum with sublateral plicae and notches absent, crenellations absent; hypomeron posterior edge near each hind angle with concavity, concavity arcuate or angulate; pronotosternal sutures open

or closed, hypomeral beads present. Prosternum with sides straight or concave at midlength in ventral view; prosternal process curved or not curved upward more than 40 degrees in lateral view. **Mesothorax.** Mesocoxal cavity open or open to mesepimeron only; mesoventral cavity without serration along sides. Elytra. Striae present; anterior edge outline straight to arcuate near humeri in dorsal view; integument marked with spots or transverse bands, marked with spot or band in apical 2/5 only or unmarked with spots or tranverse bands; without pattern from differences in setal colour; setal vestiture even and mainly parallel. **Legs.** Metacoxal plate without elongation in mesal half, plate reaching lateral edge; tarsal pads or membranous lobes absent; tarsal claws without setae, simple. **Ventrites.** Microserration at sides (e.g. 100 points per mm) present, ventrite 5 apex arcuate without paired setal brushes. **Aedeagus.** Parameres with articulation at base, apical lateral expansions present or absent; parameres without setae, each paramere with one seta, each paramere with two setae or each paramere with three or more setae. **Geography.** Known from British Columbia, Alberta, Manitoba+ (MB, SK), Ontario, Quebec, New Brunswick+ (NB, NS, PE), Oregon+ (WA, OR, ID), California, Montana+ (MT, WY, ND), Colorado+ (NV, UT, CO), Arizona+ (AZ, NM), Nebraska+ (SD, NE, IA), Texas, Michigan+ (MN, WI, MI), Indiana+ (IL, IN, OH), Arkansas+ (KS, MO, OK, AR, LA, MS), Vermont+ (ME, VT, NH), Massachusetts+ (MA, CT, RI), New York, Pennsylvania+ (PA, WV), Virginia+ (NJ, DE, MD, DC, VA), Carolinas+ (KY, TN, NC, SC, GA, AL) and Florida ['+' refers to several states or provinces collectively].

G. bicolor Van Dyke, 1932

Habitus. Body Length 5-10 mm. Vestiture. Scale like setae absent. **Head.** Supra-antennal carinae joining medially (forming shelf); nasale (head capsule below edge of frontal carina) with outline concave in lateral view; prognathous (labrum oriented less than 90 degrees downward); frons with triangular depression. Antennae with 11 antennomeres, not pectinate, sensory elements beginning on antennomere IV. **Prothorax.** Pronotum longer than wide at widest point (including hind angles); dorsal punctures uniform sized, some or all punctures umbilicate (floor of puncture flat), without tubercles or longitudinal carinae between punctures; pronotal lateral carina complete anteriorly, carina visible throughout length in dorsal view or carina not visible anteriorly in dorsal view, meeting anterior edge of prothorax at about 90 degrees in lateral view, not serrate; bioluminescent spots absent; hind angle carinae present (single); posterior edge of pronotum with sublateral plicae and notches absent, crenellations absent; hypomeron posterior edge near each hind angle with concavity, concavity arcuate; pronotosternal sutures open, hypomeral beads present. Prosternum with sides straight at midlength in ventral view; prosternal process not curved upward more than 40 degrees in lateral view. **Mesothorax.** Mesocoxal cavity open; mesoventral cavity without serration along sides. Elytra. Striae present; anterior edge outline straight to arcuate near humeri in dorsal view; integument unmarked with spots or tranverse bands; without pattern from differences in setal colour; setal vestiture even and mainly parallel. **Legs.** Metacoxal plate without elongation in mesal half, plate reaching lateral edge; tarsal pads or membranous lobes absent; tarsal claws without setae, simple. **Ventrites.** Microserration at sides (e.g. 100 points per mm) present, ventrite 5 apex arcuate without paired setal brushes. **Aedeagus.** Parameres with articulation at base, apical lateral expansions present; parameres without setae, each paramere with one seta, each paramere with two setae or each paramere with three or more setae. **Geography.** Known from British Columbia, Oregon+ (WA, OR, ID) and California ['+' refers to several states or provinces collectively].

9.3 HEMICREPIDIUS Germar, 1839

Habitus. Body Length 5-10 mm, 10-15 mm, 15-20 mm or 20-40 mm. Vestiture. Scale like setae absent. **Head.** Supra-antennal carinae joining medially (forming shelf) or directed anteriorly (reaching

anterior part of head capsule); hypognathous (labrum oriented downward 90 degrees or more) or prognathous (labrum oriented less than 90 degrees downward); frons without triangular depression. Antennae with 11 antennomeres, not pectinate, sensory elements beginning on antennomere III or IV. **Prothorax.** Pronotum wider than long at widest point (including hind angles); dorsal punctures uniform sized, all simple (floor of puncture concave), without tubercles or longitudinal carinae between punctures; pronotal lateral carina complete anteriorly, carina visible throughout length in dorsal view, meeting anterior edge of prothorax at about 90 degrees in lateral view, microserrate at hind angles only (posterior 10%, in concavity) or not serrate; bioluminescent spots absent; hind angle carinae present (single); posterior edge of pronotum with sublateral plicae and notches present or absent, crenellations absent; hypomeron posterior edge near each hind angle with or without concavity; pronotosternal sutures closed, hypomeral beads present or absent. Prosternum with sides straight at midlength in ventral view; prosternal process curved or not curved upward more than 40 degrees in lateral view. **Mesothorax.** Mesocoxal cavity open; mesoventral cavity without serration along sides. Elytra. Striae present; anterior edge outline straight to arcuate or sinuate (recurved) or with rectangular projection near humeri in dorsal view; integument unmarked with spots or tranverse bands; without pattern from differences in setal colour; setal vestiture even and mainly parallel. **Legs.** Metacoxal plate without elongation in mesal half, plate reaching or not reaching lateral edge; tarsal pads or membranous lobes present on multiple tarsomeres, (I, II, III, and IV or II, III, and IV); tarsal claws without setae, simple. **Ventrites.** Microserration at sides (e.g. 100 points per mm) present or absent, ventrite 5 apex arcuate without paired setal brushes. **Aedeagus.** Parameres with articulation at base, apical lateral expansions present; each paramere with three or more setae. **Geography.** Known from British Columbia, Alberta, Manitoba+ (MB, SK), Ontario, Quebec, New Brunswick+ (NB, NS, PE), Alaska, Oregon+ (WA, OR, ID), California, Montana+ (MT, WY, ND), Colorado+ (NV, UT, CO), Arizona+ (AZ, NM), Nebraska+ (SD, NE, IA), Michigan+ (MN, WI, MI), Indiana+ (IL, IN, OH), Arkansas+ (KS, MO, OK, AR, LA, MS), Vermont+ (ME, VT, NH), Massachusetts+ (MA, CT, RI), New York, Pennsylvania+ (PA, WV), Virginia+ (NJ, DE, MD, DC, VA), Carolinas+ (KY, TN, NC, SC, GA, AL) and Florida ['+' refers to several states or provinces collectively].

9.3 LIMONIUS Eschscholtz, 1829

Habitus. Body Length 1-5 mm, 5-10 mm or 10-15 mm. Vestiture. Scale like setae absent. **Head.** Supra-antennal carinae joining medially (forming shelf); nasale (head capsule below edge of frontal carina) with outline concave or not concave in lateral view; hypognathous (labrum oriented downward 90 degrees or more) or prognathous (labrum oriented less than 90 degrees downward); frons without triangular depression. Antennae with 11 antennomeres, not pectinate, sensory elements beginning on antennomere IV. **Prothorax.** Pronotum wider than long or longer than wide at widest point (including hind angles); dorsal punctures uniform sized, all simple (floor of puncture concave) or some or all punctures umbilicate (floor of puncture flat), without tubercles or longitudinal carinae between punctures; pronotal lateral carina complete anteriorly, carina visible throughout length in dorsal view or carina not visible anteriorly in dorsal view, meeting anterior edge of prothorax at about 90 degrees in lateral view, not serrate; bioluminescent spots absent; hind angle carinae present (single) or absent; posterior edge of pronotum with sublateral plicae and notches absent, crenellations absent; hypomeron posterior edge near each hind angle without concavity; pronotosternal sutures open or closed, hypomeral beads present. Prosternum with sides straight at midlength in ventral view; prosternal process curved or not curved upward more than 40 degrees in lateral view. **Mesothorax.** Mesocoxal cavity open or open to mesepimeron only; mesoventral cavity without serration along sides. Elytra. Striae present; anterior edge outline straight to arcuate near humeri in dorsal view; integument marked with spots or transverse bands or unmarked with spots or tranverse bands; without pattern from

differences in setal colour; setal vestiture even and mainly parallel. **Legs.** Metacoxal plate without elongation in mesal half, plate reaching or not reaching lateral edge; tarsal pads or membranous lobes absent; tarsal claws without setae, simple. **Ventrites.** Microserration at sides (e.g. 100 points per mm) present, ventrite 5 apex arcuate without paired setal brushes. **Aedeagus.** Parameres with articulation at base, apical lateral expansions present or absent; each paramere with three or more setae. **Geography.** Known from Nunavut+ (NT, NU), Yukon Territory, British Columbia, Alberta, Manitoba+ (MB, SK), Ontario, Quebec, New Brunswick+ (NB, NS, PE), Newfoundland and Labrador, Alaska, Oregon+ (WA, OR, ID), California, Montana+ (MT, WY, ND), Colorado+ (NV, UT, CO), Arizona+ (AZ, NM), Nebraska+ (SD, NE, IA), Texas, Michigan+ (MN, WI, MI), Indiana+ (IL, IN, OH), Arkansas+ (KS, MO, OK, AR, LA, MS), Vermont+ (ME, VT, NH), Massachusetts+ (MA, CT, RI), New York, Pennsylvania+ (PA, WV), Virginia+ (NJ, DE, MD, DC, VA), Carolinas+ (KY, TN, NC, SC, GA, AL) and Florida ['+' refers to several states or provinces collectively].

L. brevis Van Dyke

Habitus. Body Length 1-5 mm or 5-10 mm. Vestiture. Scale like setae absent. **Head.** Supra-antennal carinae joining medially (forming shelf); nasale (head capsule below edge of frontal carina) with outline concave in lateral view; prognathous (labrum oriented less than 90 degrees downward); frons without triangular depression. Antennae with 11 antennomeres, not pectinate, sensory elements beginning on antennomere IV. **Prothorax.** Pronotum longer than wide at widest point (including hind angles); dorsal punctures uniform sized, all simple (floor of puncture concave), with or without tubercles or longitudinal carinae between punctures; pronotal lateral carina complete anteriorly, carina visible throughout length in dorsal view or carina not visible anteriorly in dorsal view, meeting anterior edge of prothorax at about 90 degrees in lateral view, not serrate; bioluminescent spots absent; hind angle carinae present (single) or absent; posterior edge of pronotum with sublateral plicae and notches absent, crenellations absent; hypomeron posterior edge near each hind angle without concavity; pronotosternal sutures open, hypomeral beads present. Prosternum with sides straight or concave at midlength in ventral view; prosternal process curved upward more than 40 degrees in lateral view. **Mesothorax.** Mesocoxal cavity open to mesepimeron only; mesoventral cavity without serration along sides. Elytra. Striae present; anterior edge outline straight to arcuate near humeri in dorsal view; integument unmarked with spots or tranverse bands; without pattern from differences in setal colour; setal vestiture even and mainly parallel. **Legs.** Metacoxal plate without elongation in mesal half, plate reaching lateral edge; tarsal pads or membranous lobes present on multiple tarsomeres, (II and III); tarsal claws without setae, simple. **Ventrites.** Microserration at sides (e.g. 100 points per mm) present, ventrite 5 apex arcuate without paired setal brushes. **Aedeagus.** Parameres with articulation at base, apical lateral expansions present; each paramere with three or more setae. **Geography.** Known from British Columbia, Oregon+ (WA, OR, ID) and California ['+' refers to several states or provinces collectively].

9.3 PHELETES Kiesenwetter, 1858

Habitus. Body Length 1-5 mm or 5-10 mm. Vestiture. Scale like setae absent. **Head.** Supra-antennal carinae joining medially (forming shelf); frons without triangular depression. Antennae with 11 antennomeres, not pectinate, sensory elements beginning on antennomere IV. **Prothorax.** Pronotum wider than long or longer than wide at widest point (including hind angles); dorsal punctures uniform sized, all simple (floor of puncture concave), without tubercles or longitudinal carinae between punctures; pronotal lateral carina complete anteriorly, carina visible throughout length in dorsal view, meeting anterior edge of prothorax at about 90 degrees in lateral view, not serrate; bioluminescent spots absent; hind angle carinae present (single); posterior edge of pronotum with sublateral plicae and

notches absent, crenellations absent; hypomeron posterior edge near each hind angle without concavity; pronotosternal sutures open or closed, hypomeral beads present. Prosternum with sides straight at midlength in ventral view. **Mesothorax.** Mesocoxal cavity open or open to mesepimeron only; mesoventral cavity without serration along sides. Elytra. Striae present; integument unmarked with spots or tranverse bands; without pattern from differences in setal colour; setal vestiture even and mainly parallel. **Legs.** Metacoxal plate without elongation in mesal half, plate reaching lateral edge; tarsal pads or membranous lobes absent; tarsal claws without setae, simple. **Ventrites.** Microserration at sides (e.g. 100 points per mm) present, ventrite 5 apex arcuate without paired setal brushes. **Aedeagus.** Parameres with articulation at base, apical lateral expansions present; each paramere with three or more setae. **Geography.** Known from British Columbia and Oregon+ (WA, OR, ID) ['+' refers to several states or provinces collectively].

9.3 TETRALIMONIUS Etzler, 2019

Habitus. Body Length 1-5 mm or 5-10 mm. Vestiture. Scale like setae absent. **Head.** Supra-antennal carinae joining medially (forming shelf); nasale (head capsule below edge of frontal carina) with outline concave in lateral view; hypognathous (labrum oriented downward 90 degrees or more) or prognathous (labrum oriented less than 90 degrees downward); frons without triangular depression. Antennae with 11 antennomeres, not pectinate, sensory elements beginning on antennomere IV. **Prothorax.** Pronotum wider than long or longer than wide at widest point (including hind angles); dorsal punctures uniform sized, all simple (floor of puncture concave) or some or all punctures umbilicate (floor of puncture flat), without tubercles or longitudinal carinae between punctures; pronotal lateral carina complete anteriorly, carina visible throughout length in dorsal view, meeting anterior edge of prothorax at about 90 degrees in lateral view, not serrate; bioluminescent spots absent; hind angle carinae present (single) or absent; posterior edge of pronotum with sublateral plicae and notches absent, crenellations absent; hypomeron posterior edge near each hind angle without concavity; pronotosternal sutures open, hypomeral beads present. Prosternum with sides straight at midlength in ventral view; prosternal process curved or not curved upward more than 40 degrees in lateral view. **Mesothorax.** Mesocoxal cavity open or open to mesepimeron only; mesoventral cavity without serration along sides. Elytra. Striae present; anterior edge outline straight to arcuate near humeri in dorsal view; integument marked with spots or transverse bands or unmarked with spots or tranverse bands; without pattern from differences in setal colour; setal vestiture even and mainly parallel. **Legs.** Metacoxal plate without elongation in mesal half, plate reaching lateral edge; tarsal pads or membranous lobes absent; tarsal claws without setae, simple. **Ventrites.** Microserration at sides (e.g. 100 points per mm) present, ventrite 5 apex arcuate without paired setal brushes. **Aedeagus.** Parameres with articulation at base, apical lateral expansions absent; each paramere with one seta or each paramere with two setae. **Geography.** Known from British Columbia, California, Montana+ (MT, WY, ND), Indiana+ (IL, IN, OH), Arkansas+ (KS, MO, OK, AR, LA, MS), Vermont+ (ME, VT, NH), Massachusetts+ (MA, CT, RI), New York, Pennsylvania+ (PA, WV), Virginia+ (NJ, DE, MD, DC, VA), Carolinas+ (KY, TN, NC, SC, GA, AL) and Florida ['+' refers to several states or provinces collectively].

9.3 VITTATHOUS Johnson, 2021

Habitus. Body Length 10-15 mm or 15-20 mm. Vestiture. Scale like setae absent. **Head.** Supra-antennal carinae fading on frons (not reaching another structure) or directed anteriorly (reaching anterior part of head capsule); hypognathous (labrum oriented downward 90 degrees or more) or prognathous (labrum oriented less than 90 degrees downward); frons without triangular depression. Antennae with 11 antennomeres, not pectinate, sensory elements beginning on antennomere III. **Prothorax.** Pronotum

longer than wide at widest point (including hind angles); dorsal punctures uniform sized, all simple (floor of puncture concave), without tubercles or longitudinal carinae between punctures; pronotal lateral carina complete anteriorly, carina visible throughout length in dorsal view, meeting anterior edge of prothorax at about 90 degrees in lateral view, not serrate; bioluminescent spots absent; hind angle carinae present (single); posterior edge of pronotum with sublateral plicae and notches absent, crenellations absent; hypomeron posterior edge near each hind angle without concavity; pronotosternal sutures closed, hypomeral beads absent. Prosternum with sides straight at midlength in ventral view; prosternal process not curved upward more than 40 degrees in lateral view. **Mesothorax.** Mesocoxal cavity open; mesoventral cavity without serration along sides. Elytra. Striae present; anterior edge outline straight to arcuate near humeri in dorsal view; integument unmarked with spots or tranverse bands; without pattern from differences in setal colour; setal vestiture even and mainly parallel. **Legs.** Metacoxal plate without elongation in mesal half, plate reaching lateral edge; tarsal pads or membranous lobes absent; tarsal claws without setae, simple. **Ventrites.** Microserration at sides (e.g. 100 points per mm) absent, ventrite 5 apex arcuate without paired setal brushes. **Aedeagus.** Parameres with articulation at base, apical lateral expansions present; each paramere with two setae. **Geography.** Known from Oregon+ (WA, OR, ID), California and Carolinas+ (KY, TN, NC, SC, GA, AL) ['+' refers to several states or provinces collectively].
